# Built environmental correlates of older adults’ total physical activity and walking: a systematic review and meta-analysis

**DOI:** 10.1186/s12966-017-0558-z

**Published:** 2017-08-07

**Authors:** David W. Barnett, Anthony Barnett, Andrea Nathan, Jelle Van Cauwenberg, Ester Cerin, on behalf of the Council on Environment and Physical Activity (CEPA) – Older Adults working group

**Affiliations:** 1 Institute for Health and Ageing, Australian Catholic University, Level 6, 215 Spring Street, Melbourne, VIC 3000 Australia; 2 Department of Public Health, Ghent University, De Pintelaan 185, 9000 Ghent, Belgium; 3 Research Foundation Flanders, Egmontstraat 5, 1000 Brussels, Belgium; 4 School of Public Health, The University of Hong Kong, 7 Sassoon Road, Pokfulam, Hong Kong, Special Administrative Region China; 5 Baker IDI Heart and Diabetes Institute, 75 Commercial Road, Melbourne, VIC 3004 Australia

**Keywords:** Older adults, Built environment, Physical activity, Walking, Correlates, Systematic review, Meta-analysis

## Abstract

**Background:**

Identifying attributes of the built environment associated with health-enhancing levels of physical activity (PA) in older adults (≥65 years old) has the potential to inform interventions supporting healthy and active ageing. The aim of this study was to first systematically review and quantify findings on built environmental correlates of older adults’ PA, and second, investigate differences by type of PA and environmental attribute measurement.

**Methods:**

One hundred articles from peer-reviewed and grey literature examining built environmental attributes related to total PA met inclusion criteria and relevant information was extracted. Findings were meta-analysed and weighted by article quality and sample size and then stratified by PA and environmental measurement method. Associations (*p* < .05) were found in relation to 26 individual built environmental attributes across six categories (walkability, residential density/urbanisation, street connectivity, access to/availability of destinations and services, infrastructure and streetscape, and safety) and total PA and walking specifically. Reported individual- and environmental-level moderators were also examined.

**Results:**

Positive environmental correlates of PA, ranked by strength of evidence, were: walkability (*p* < .001), safety from crime (*p* < .001), overall access to destinations and services (*p* < .001), recreational facilities (*p* < .001), parks/public open space (*p* = .002) and shops/commercial destinations (*p* = .006), greenery and aesthetically pleasing scenery (*p* = .004), walk-friendly infrastructure (*p* = .009), and access to public transport (*p* = .016). There were 26 individual differences in the number of significant associations when the type of PA and environmental measurement method was considered. No consistent moderating effects on the association between built environmental attributes and PA were found.

**Conclusions:**

Safe, walkable, and aesthetically pleasing neighbourhoods, with access to overall and specific destinations and services positively influenced older adults’ PA participation. However, when considering the environmental attributes that were sufficiently studied (i.e., in ≥5 separate findings), the strength of evidence of associations of specific categories of environment attributes with PA differed across PA and environmental measurement types. Future research should be mindful of these differences in findings and identify the underlying mechanisms. Higher quality research is also needed.

**Electronic supplementary material:**

The online version of this article (doi:10.1186/s12966-017-0558-z) contains supplementary material, which is available to authorized users.

## Background

Worldwide, the proportion of older adults (65 years or older) is forecast to grow exponentially from 524 million in 2010 to approximately 1.5 billion individuals by 2050 [1]. This will pose a major economic challenge for societies globally, given the healthcare expenditure associated with individuals experiencing age-related chronic diseases [1, 2]. Evidence suggests that regular engagement in PA is particularly important for healthy ageing. For example, it reduces the risk of coronary heart disease, some cancers, type 2 diabetes, depression, cognitive impairment and social isolation [3, 4]. Older adults worldwide, however, are often inactive [4–7]. Thus, it is important to identify modifiable factors with a high level of reach that may help increase total PA in this age group. As it is ultimately the overall dose of PA that confers benefit/detriment upon health [8], irrespective of the domain/s in which it was accrued, it is important to focus on factors contributing to total PA. Furthermore, walking is the most prevalent and preferred form of PA in older adults [9], low-risk and beneficial to health and can contribute substantially to daily energy expenditure [10]. Hence, this review will focus on total PA and total walking.

Socio-ecological models posit that PA behaviour is shaped by complex and dynamic interrelations between individual, social, and environmental factors [11, 12]. The built environment offers substantial public health potential, due to people being regularly exposed to it across their life span. Understanding the impact of built environmental attributes on older adults’ PA is particularly pertinent as their diminished physical capacity makes them more vulnerable to the detrimental effects of physically challenging environments (e.g., inclines, uneven surfaces, absence of walk-friendly infrastructure) on PA [13]. In turn, this may lead to less venturing outside of the home due to fear of falls [14]. However, a previous review of the built environment and older adults’ PA identified no consistent correlates [15].

One postulated reason for the lack of consistent significant associations between environmental correlates and PA was the relative ‘western’ bias of the reviewed research – 68% of the 31 articles reviewed by Van Cauwenberg and colleagues were from North America alone [15]. This is an important limitation since homogenously low-density western cities lack environmental variability potentially resulting in the underestimation of the strength of associations between the built environment and PA. Also, western cities differ from the built environments of Africa, Asia, and South America, limiting the generalizability of findings [16–18]. In consideration of the recent growing evidence from non-western countries (e.g., [19–25]), an update of the literature was necessary.

Further reasons for inconsistent findings on the associations between the built environment and PA may relate to the methodologies of the systematic reviews and/or the studies being reviewed. For example, Gebel and colleagues [26] recommended that systematic reviews should: a) consider article/study quality in the synthesis of findings; and b) include relevant data from grey literature. Also, small sample sizes, a large variety of built environmental exposures and PA outcomes [27, 28], and the inappropriate categorisation of continuous variables [29] in examined studies may have contributed to inconsistent findings. To date, no systematic review on the built environment and total PA has considered these issues.

A synthesis of evidence would also need to distinguish between findings based on objective- and self-report measures of exposures (environmental attributes) and outcomes (PA). Self-report measures are more likely to be influenced by culture [30, 31] and, thus, yield different findings across geographical locations due to measurement rather than substantive reasons. Also, perceptions of the local environment may not accurately represent the ‘real’ environment [32, 33]. Indeed, associations of PA with objective and perceived measures of the built environment tend to differ [34, 35]. This does not necessarily mean that one type of measurement is better than the other, however, as perceived environmental measures may be more closely associated with PA than objective alternatives [27], a consideration of these differences would help better inform policies and interventions.

With regard to measurement of total PA, objective PA measures are considered to provide more valid assessments of intensity, duration, and frequency of PA than subjective alternatives [36]. They are also less likely to be influenced by cultural biases [30]. Additionally, differences in environment-PA associations between self-reported and objectively-measured PA have previously been reported in adults [37]. This indicates that there is a need for a synthesis of findings on built environment correlates of total PA by type of PA assessment (objective and self-reported).

Therefore, this systematic review aimed to provide a timely, robust overview of studies that investigated associations of built environmental attributes and estimates of total PA, including total walking. This included addressing some key methodological limitations of previous reviews by stratifying findings by measurement methods (objective and self-reported) and applying a meta-analytic procedure. The latter incorporated study quality data to more robustly quantify the direction of associations between the built environment and older adults’ PA [27] and assisted the formulation of objective conclusions based on statistical theory rather than on subjective criteria (e.g., defining >50% of significant positive associations as convincing evidence of a positive association between a specific environment characteristic and PA).

## Methods

This systematic review and meta-analysis was registered in PROSPERO (Registration no. CRD42016051227 [38]) in November 2016.

### Literature search strategy

**Fig. 1:**
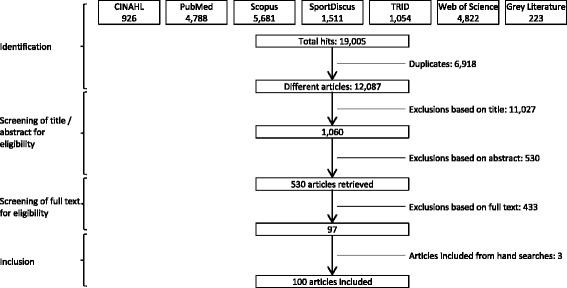
PRISMA flowchart

Our systematic literature search followed Preferred Reporting Items for Systematic Reviews and Meta-Analyses (PRISMA) guidelines [39] and was an extended update of Van Cauwenberg et al.’s 2011 review [15] including additional electronic databases and search terms to account for grey literature, experimental research, a greater variety of built environmental attributes and older adults’ PA. Systematic searches were performed in the following electronic databases (September – November 2016): Cinahl, PubMed, Scopus, SPORTDiscus, TRIS, and Web of Science. An example of the complete search terms syntax (used in PubMed) is presented in the resulting PRISMA flowchart (Fig. 1). All electronic database searches used the same search terms and syntax. However, filters varied depending on available options in each database. Manual searches were then undertaken of previously published systematic reviews, meta-analyses and authors’ personal archives. The websites of Active Living Research, SUSTRANS, the National Institute for Health and Clinical Excellence, Heart Foundation, and Open Grey were also searched for grey literature (e.g., unpublished studies, theses and reports).

### Selection criteria

Article eligibility for inclusion was based on the following criteria: (1) published in English between 1st January 2000 and 3rd September 2016; (2) quantitative study; (3) study with a cross-sectional, longitudinal or quasi-experimental design; (4) a sample with a mean age ≥ 65 years; (5) investigated the association between any objective or perceived attribute of the built environment and objectively measured or self-reported PA and/or walking that was not specific to a single PA domain only; (6) did not exclusively focus on clinical populations (e.g., overweight, disabled or institutionalised participants); and (7) did not investigate associations between PA and the built environment with an ill-defined aggregated environmental measure that, for example, combined two weakly correlated attributes such as access to/availability of recreational facilities and traffic/pedestrian safety.

Three reviewers independently screened articles for eligibility criteria by title and then by abstract. If abstracts met the inclusion criteria, the article text was independently read by two reviewers, and a decision for inclusion made accordingly. The reviewers discussed and resolved any disputed inclusions. Post article inclusion, first authors’ publication histories and eligible articles’ reference lists were screened for additional eligible articles. One hundred articles were eligible for inclusion in this review (Fig. 1).

### Data extraction

Two reviewers independently extracted all relevant information from eligible papers and cross-checked each other’s work upon completion. Any disputes were resolved in consultation with a third member of the team. A table (Additional file 1) was then constructed containing the following data: study name (if any), first author and publication year; participant information – sample size, study setting (e.g., urban or rural), mean age, percentage of females, study response rate, community dwellers or not, geographical location; study design – sampling method for clusters (areas or neighbourhoods) and individuals, stratification of recruitment site by environmental attribute/s (if any), neighbourhood definition (if any); list of covariates; PA outcome information – type of measurement (objective or self-reported), instrument or device used and whether or not it was validated, how outcome measures were operationalised (e.g., continuously or categorically); environmental exposure information – type of measurement (objective or perceived), name of measure, environmental attributes as named in study and category of environmental attribute according to the classification used in this review (see below); moderator information (if any); analytical approach information; findings; and additional comments important for the assessment or interpretation of the study (if any).

In this review, the variable “Total PA” represents all (combined) PA outcomes relevant to this review and extracted from the selected articles. These include the PA outcomes reported in the selected articles as “Total PA”, “Total MVPA” and “Total walking”. “Total PA” was then stratified into objective and self-report PA, congruent with study aims. Total walking was also considered separately for reasons aforementioned. Built environmental attributes were categorised according to an expanded list of factors included in the Neighbourhood Environment Walkability Scale (NEWS), which is the most frequently used neighbourhood environment questionnaire internationally [40, 41] (Table 1). The detailed list of measures included under each category of environmental attributes can be found in Supplementary Table S1 (Additional file 1).

**Table 1:** Characteristics of selected articles (*N* = 100)

Characteristic	Number of articles	%	Article reference
Study design^a^			
Cross-sectional	95	94	[19–25, 32, 43–50, 53, 54, 59, 60, 65, 67–80, 87, 92, 93, 100, 118–173]
Longitudinal	5	5	[61–65]
Quasi-experimental	1	1	[66]
Geographical area: continent			
Africa	1	1	[25]
Asia	16	16	[19–22, 60, 73, 75, 80, 124, 139, 142, 146, 160, 164, 169, 173]
Europe	22	22	[50, 66, 74, 79, 87, 119, 120, 123, 129, 136, 137, 147, 148, 152, 154, 156, 157, 162, 163, 165, 166, 170]
North America	46	46	[32, 43–48, 53, 54, 61–65, 67–70, 76, 78, 92, 93, 125–127, 130–135, 138, 140, 141, 143, 144, 149–151, 155, 158, 159, 161, 167, 168, 172]
Oceania	13	13	[49, 59, 71, 72, 77, 100, 118, 121, 122, 128, 145, 153, 171]
South America	2	2	[23, 24]
Geographical area: country			
Australia	13	13	[49, 59, 71, 72, 77, 100, 118, 121, 122, 128, 145, 153, 171]
Belgium	2	2	[74, 170]
Brazil	1	1	[23]
Canada	10	10	[54, 61, 62, 78, 125–127, 135, 138, 172]
China	1	1	[80]
Colombia	1	1	[24]
Czech Republic, Poland, & Slovakia (pooled analysis)	1	1	[154]
Hong Kong, China	2	2	[73, 124]
Iceland	1	1	[119]
Iran	1	1	[146]
Ireland	2	2	[147, 152]
Japan	7	7	[19–22, 160, 164, 169]
Lithuania	1	1	[120]
Malaysia	1	1	[139]
Netherlands	4	4	[123, 136, 137, 166]
Norway	1	1	[157]
Singapore	1	1	[142]
South Africa	1	1	[25]
South Korea	2	2	[75, 173]
Thailand	1	1	[60]
United Kingdom	10	10	[50, 66, 79, 87, 129, 148, 156, 162, 163, 165]
United States of America	36	36	[32, 43–48, 53, 63–65, 67–70, 76, 92, 93, 130–134, 140, 141, 143, 144, 149–151, 155, 158, 159, 161, 167, 168]
Geographical setting			
Urban	56	56	[23–25, 32, 43–48, 50, 53, 59, 61–64, 66, 71, 72, 74, 80, 87, 92, 93, 100, 120–122, 124–127, 129–131, 133, 135–138, 142, 144, 146, 149, 153, 156–158, 161, 164, 165, 167, 170, 172]
Rural	3	3	[67–69]
Mixed	32	32	[19, 21, 22, 49, 54, 60, 75, 76, 79, 118–120, 123, 128, 132, 134, 139, 143, 145, 147, 148, 150, 152, 155, 159, 160, 162, 163, 166, 169, 171, 173]
Not reported	9	9	[65, 70, 77, 78, 140, 141, 151, 154, 168]
Sample size^b^			
≤ 100	9	9	[19, 25, 53, 61, 66, 118, 127, 135, 161]
101–300	27	27	[32, 43, 53, 59, 60, 67, 69, 76, 87, 119, 120, 122, 123, 129, 132, 133, 140–142, 146, 150, 156, 162–166]
301–500	23	23	[23, 47, 63, 64, 68, 71–75, 93, 100, 121, 124, 126, 131, 134, 153, 154, 160, 167, 169, 170]
501–1000	16	16	[44–46, 48, 62, 78, 79, 92, 125, 128, 138, 144, 147, 148, 158, 159]
1001–2500	11	11	[20, 22, 24, 50, 54, 77, 130, 151, 155, 172, 173]
> 2500	16	16	[21, 49, 54, 65, 70, 80, 136, 137, 139, 143, 145, 149, 152, 157, 168, 171]
Study with multiple articles			
Active Living Study	3	3	[71, 72, 153]
BEPAS Seniors	2	2	[74, 170]
Great Britain older adults 1	2	2	[162, 163]
Health and Wellbeing Surveillance System	2	2	[49, 171]
LL-FDI study (Late-Life Function and Disability Instrument)	2	2	[132, 150]
Melbourne older adults study 1	2	2	[121, 122]
Netherlands Housing Survey (WoON)	2	2	[136, 137]
Nurses’ Health Study	2	2	[70, 168]
PACS (Physical Activity Cohort Scotland)	2	2	[79, 148]
Project OPAL (Older People Active Living)	3	3	[87, 129, 165]
SHAPE (Senior Health and Physical Exercise)	4	4	[32, 63, 92, 93]
SNQLS (Senior Neighborhood Quality of Life Study)	6	6	[43–48]
TILDA (The Irish Longitudinal Study on Ageing)	2	2	[147, 152]
VoisiNuAge	2	2	[62, 138]
Single publication (named study)	33	33	[21, 50, 54, 59, 61, 64–66, 73, 77, 78, 80, 100, 124, 128, 130, 133–135, 139, 140, 143–145, 149, 155, 157–159, 166, 169, 172, 173]
Single publication (unnamed study)	31	31	[19, 20, 22–25, 53, 60, 67–69, 75, 76, 118–120, 123, 125–127, 131, 141, 142, 146, 151, 154, 156, 160, 161, 164, 167]
Neighbourhood recruitment stratification^a^			
*Environmental attributes*	57	57	[19, 21–25, 32, 44–50, 54, 60, 63, 66, 68, 71–76, 78–80, 87, 92, 118, 119, 123, 124, 126, 129, 130, 134, 135, 139, 146–148, 151, 153, 155, 159, 160, 162–166, 168, 170–172]
Urbanisation	22	22	[19, 21, 22, 49, 54, 60, 68, 75, 78, 80, 118, 119, 123, 134, 139, 155, 159, 160, 166, 168, 171, 172]
Area-level socio-economic status	6	6	[23, 25, 87, 129, 151, 165]
Area-level socio-economic status & walkability	12	12	[44–48, 73, 74, 124, 126, 135, 146, 170]
Area-level socio-economic status & urbanisation	10	10	[50, 63, 76, 79, 92, 130, 147, 148, 162, 163]
Walkability	4	4	[32, 71, 72, 153]
Streetscape	2	2	[66, 164]
*Demographics*	31	31	[20–22, 50, 62, 64, 69, 70, 79, 119, 121, 122, 132–134, 136–138, 141–144, 148, 150, 151, 155, 156, 161–163, 173]
None	21	21	[43, 53, 59, 61, 65, 67, 77, 93, 100, 120, 125, 128, 131, 140, 145, 149, 154, 157, 158, 167, 169]
Neighbourhood definition^a^			
*Objective*			
Administrative/census area/postal code	25	25	[19, 54, 65, 68, 70, 74, 80, 118, 134, 136–139, 142, 145, 146, 148, 149, 157, 158, 160, 166, 170, 172, 173]
Buffer (crow-fly or road-network)			
≤ 250 m	3	3	[21, 64, 171]
300 m	1	1	[123]
400–500 m	16	16	[21, 24, 44, 46–49, 59, 64, 72, 73, 93, 135, 153, 159, 171]
800–1000 m	15	15	[21, 49, 59, 63, 64, 73, 92, 93, 130, 132, 135, 155, 161, 168, 171]
> 1000 m	3	3	[161, 168, 171]
Variable/not fixed	4	4	[32, 135, 151, 164]
Retirement village	3	3	[43, 72, 153]
Unknown (not reported)	3	3	[53, 62, 128]
*Perceived*			
10–20 min walk from home	24	24	[20, 22, 23, 44–46, 48, 60, 71, 72, 75, 79, 100, 121, 122, 124, 126, 131, 132, 141, 143, 150, 159, 169]
Other participant delineation	22	22	[24, 25, 32, 50, 61, 62, 67, 69, 76, 78, 87, 120, 125, 129, 133, 140, 147, 151, 152, 154, 165, 167]
Retirement village	2	2	[71, 153]
Unknown (not reported)	6	6	[63, 77, 92, 134, 144, 156]
Physical activity outcome by type of measurement and its operationalisation^a^			
Total PA (all PA outcomes from all articles)			
Continuous outcome	59	55.1	[19, 21, 43–48, 54, 59, 60, 62, 63, 65–67, 69, 71, 73, 74, 79, 80, 87, 92, 93, 118, 119, 121, 123–125, 127, 129, 131, 132, 134–138, 140–144, 147–151, 155, 160, 161, 164–166, 170, 173]
Categorical outcome	48	44.9	[20, 22–25, 32, 49, 50, 53, 54, 61, 64, 65, 68, 70, 72, 75–78, 80, 93, 100, 120, 122, 124, 126, 128, 130, 133, 135, 139, 144, 146, 152–154, 156–159, 162, 163, 167–169, 171, 172]
*Objective PA*	28	27.2	
Continuous outcome	23		[19, 43–48, 59, 73, 74, 79, 118, 127, 129, 132, 135, 141, 148, 150, 164–166, 170]
Categorical outcome	5		[25, 50, 61, 72, 135, 153]
*Self-reported PA*	75	73.8	
Continuous outcome	37		[19, 21, 54, 60, 62, 63, 65–67, 69, 71, 73, 80, 87, 92, 93, 119, 121, 123–125, 131, 134, 136–138, 140–144, 147, 149, 151, 155, 160, 161, 173]
Categorical outcome	45		[20, 22–25, 32, 49, 53, 54, 64, 65, 68, 70, 72, 75–78, 80, 93, 100, 120, 122, 124, 126, 128, 130, 133, 139, 144, 146, 152–154, 156–159, 162, 163, 167–169, 171, 172]
Total PA (as detailed in article)	31	27.2	
*Objective PA*	8	8	
Continuous outcome	8	8	[48, 79, 129, 135, 148, 150, 164, 166]
Categorical outcome	0	0	
*Self-reported PA*	23	23	
Continuous outcome	19	19	[19, 60, 65–67, 69, 73, 87, 119, 121, 125, 136, 137, 141, 143, 147, 151, 160, 161]
Categorical outcome	4	4	[65, 70, 126, 157]
Total walking (as detailed in article)	55	48.2	
*Objective PA*	9	9	
Continuous outcome	8	8	[19, 118, 127, 129, 132, 135, 141, 165]
Categorical outcome	2	2	[61, 135]
*Self-reported PA*	47	47	
Continuous outcome	19	19	[21, 62, 63, 65, 67, 71, 80, 92, 93, 123, 124, 131, 134, 138, 142, 144, 149, 155, 173]
Categorical outcome	32	32	[20, 22–24, 32, 49, 53, 64, 65, 70, 72, 75, 76, 80, 93, 100, 122, 124, 130, 144, 146, 153, 154, 158, 159, 162, 163, 167–169, 171, 172]
Total MVPA^c^ (as detailed in article)	28	24.6	
*Objective PA*	15	15	
Continuous outcome	11	11	[43–48, 73, 74, 129, 166, 170]
Categorical outcome	4	4	[25, 50, 72, 153]
*Self-reported PA*	14	14	
Continuous outcome	3	3	[54, 140, 173]
Categorical outcome	12	12	[25, 54, 68, 77, 78, 120, 128, 130, 133, 139, 152, 156]
Environmental attribute by type of measurement^a^			
*Objective environment*	49	48	
*Perceived environment*	53	52	
Walkability	13	13	
*Objective*	11	11	[46, 48, 72–74, 146, 153, 155, 167, 171, 172]
*Perceived*	2	2	[126, 141]
Residential density/urbanisation^d^	35	35	
*Objective*	21	21	[19, 21, 47, 54, 65, 70, 73, 80, 92, 118, 119, 123, 128, 134, 139, 148, 158–160, 168, 173]
*Perceived*	15	15	[20, 22, 25, 48, 76, 132, 140, 145, 147, 150, 152, 154, 159, 162, 169]
Street connectivity^d^	24	24	
*Objective*	10	10	[21, 24, 43, 53, 73, 93, 159, 161, 166, 168]
*Perceived*	16	16	[20, 23, 25, 48, 53, 71, 76, 79, 127, 132, 140, 142, 150, 154, 159, 170]
Access to/availability of destinations & services^a^	65	65	
*Objective*	29	29	
*Perceived*	45	45	
Overall access to destinations & services^d^	24	24	
*Objective*	6	6	[21, 53, 62, 93, 135, 168]
*Perceived*	21	21	[20, 23, 25, 48, 50, 53, 62, 67, 71, 76, 87, 100, 126, 127, 131, 132, 140, 141, 150, 154, 159]
Land-use mix—destination diversity^d^	16	16	
*Objective*	8	8	[49, 72, 80, 123, 135, 153, 159, 161]
*Perceived*	9	9	[25, 48, 67, 76, 125, 132, 150, 159, 170]
Shops/commercial destinations^d^	26	26	
*Objective*	17	17	[32, 43, 47, 49, 53, 72, 73, 80, 93, 135, 148, 158, 159, 161, 166, 168]
*Perceived*	10	10	[22, 23, 32, 75, 129, 141, 142, 156, 165, 169]
Food outlets	11	11	
*Objective*	8	8	[43, 47, 49, 53, 73, 135, 166, 168]
*Perceived*	3	3	[23, 141, 142]
Government/finance services	8	8	
*Objective*	7	7	[43, 47, 49, 53, 73, 135, 158]
*Perceived*	1	1	[23]
Education	7	7	
*Objective*	6	6	[21, 132, 135, 158, 161, 168]
*Perceived*	1	1	[141]
Health & aged care	10	10	
*Objective*	8	8	[43, 49, 53, 72, 135, 153, 158, 166]
*Perceived*	2	2	[23, 156]
Religious	5	5	
*Objective*	3	3	[43, 135, 161]
*Perceived*	2	2	[23, 141]
Public transport	18	18	
*Objective*	8	8	[24, 47, 72, 73, 80, 93, 158, 166]
*Perceived*	10	10	[22, 23, 48, 50, 62, 68, 100, 141, 156, 169]
Parks/public open space^d^	30	30	
*Objective*	17	17	[21, 24, 32, 43, 44, 48, 53, 64, 73, 92, 93, 124, 132, 135, 151, 158, 161]
*Perceived*	15	15	[23, 32, 46, 66, 68, 69, 75, 100, 125, 141, 142, 144, 151, 156, 163]
Recreational facilities	25	25	
*Objective*	10	10	[43, 47, 64, 72, 73, 124, 132, 135, 153, 168]
*Perceived*	15	15	[22, 23, 46, 60, 63, 68, 77, 78, 92, 120, 125, 142, 156, 169, 170]
Social recreational facilities	13	13	
*Objective*	6	6	[43, 49, 73, 135, 138, 166]
*Perceived*	7	7	[23, 50, 71, 141, 142, 153, 156]
Other destinations	1	1	
*Objective*	1	1	[43]
*Perceived*	0	0	
Infrastructure & streetscape^a^	43	43	
*Objective*	12	12	
*Perceived*	34	34	
Overall cycle/walk-friendly infrastructure	8	8	
*Objective*	0	0	
*Perceived*	9	9	[25, 48, 66, 76, 127, 132, 150, 154, 159]
Walk-friendly infrastructure	21	21	
*Objective*	7	7	[43, 47, 80, 93, 124, 132, 161]
*Perceived*	14	14	[22, 44, 53, 61, 62, 68, 69, 71, 100, 121, 125, 142, 169, 170]
Cycle-friendly infrastructure	4	4	
*Objective*	1	1	[47]
*Perceived*	3	3	[22, 125, 169]
No physical environmental barriers (e.g., hills)	16	16	
*Objective*	8	8	[21, 24, 32, 43, 47, 72, 124, 164]
*Perceived*	9	9	[23, 32, 66, 71, 100, 125, 142, 169, 170]
Pavement/footpath quality	8	8	
*Objective*	2	2	[47, 124]
*Perceived*	6	6	[23, 24, 66, 121, 142, 163]
Street lighting	6	6	
*Objective*	1	1	[124]
*Perceived*	5	5	[23, 53, 69, 75, 122]
Greenery & aesthetically pleasing scenery^d^	33	33	
*Objective*	6	6	[32, 43, 47, 80, 124, 161]
*Perceived*	28	28	[20, 22, 23, 25, 32, 44, 48, 53, 61, 71, 75, 76, 79, 100, 122, 125, 127, 132, 137, 142, 147, 150, 154, 156, 159, 163, 169, 170]
Pollution (air)	3	3	
*Objective*	1	1	[124]
*Perceived*	2	2	[23, 156]
Safety	46	46	
*Objective*	7	7	
*Perceived*	40	40	
Traffic/pedestrian safety	28	28	
*Objective*	4	4	[47, 72, 93, 124]
*Perceived*	24	24	[22–25, 45, 48, 53, 61, 69, 71, 75, 76, 79, 92, 100, 125, 132, 136, 142, 150, 156, 159, 169, 170]
Crime/personal safety^d^	41	41	
*Objective*	5	5	[43, 93, 124, 149, 157]
*Perceived*	37	37	[22, 23, 25, 45, 48, 50, 53, 61, 63, 66, 68, 69, 71, 75, 76, 79, 100, 121, 122, 125, 132–134, 137, 140, 142–145, 150, 156, 157, 159, 163, 167, 169, 170]
Moderator of environmental attribute-PA association^a^	39	39	
*Individual factors*	24	24	[20–23, 44, 45, 48–50, 59, 69, 70, 73, 77, 79, 100, 122, 125, 131, 144, 152, 155, 157, 159]
Socio-demographics	16	16	[20–23, 45, 49, 50, 69, 70, 73, 122, 125, 144, 152, 155, 157]
Health status/functionality	7	7	[59, 73, 77, 100, 131, 155, 159]
Psychosocial factors	3	3	[44, 79, 100]
Driving status/car ownership	2	2	[48, 73]
Duration of residency	1	1	[21]
*Environmental factors*	18	18	[19, 21, 25, 45, 46, 64, 66, 74–76, 92, 100, 141, 146, 155, 160, 162, 168]
Socioeconomic status/area-level income	5	5	[25, 45, 64, 74, 146]
Residential density/urbanisation	6	6	[19, 75, 76, 160, 162, 168]
Infrastructure and streetscape aspects (e.g., walkability)	3	3	[46, 92, 100]
Intervention	2	2	[66, 100]
Geographical scale (e.g., 400 m buffer)	6	6	[21, 64, 73, 93, 135, 161]
Tested, but unknown	1	1	[24]
None	61	61	[32, 43, 47, 53, 54, 60–63, 65, 67, 68, 71, 72, 78, 80, 87, 93, 118–124, 126–130, 132–143, 145, 147–151, 153, 154, 156, 158, 163–167, 169–173]

*Notes*:

^a^Multiple options allowed in single articles

^b^Two articles had instances where environmental attributes were associated with different sample sizes ([53, 54]). Hence, the total number of articles added up separately is 2 units more than the total number of articles. Notably, this was adjusted for in our analysis

^c^One article ([25]) had both objective and self-reported physical activity measures. Hence, the total number of articles is 1 unit smaller than the sum of the articles in these cases

^d^Multiple articles had both objective and perceived environmental measures. Hence, the total number of articles is 1, 2, or 3 unit/s smaller than the sum of the articles in these cases

### Coding and quantification of associations

Following the protocol used by Van Cauwenberg and colleagues in their earlier systematic review [15], associations between built environmental attributes and PA outcomes were categorised as statistically significantly positive (P), significantly negative (N), or not statistically significant (Ø). When available, preference was given to findings within articles that adjusted for socio-demographic covariates and self-selection (i.e., factors that may affect neighbourhood choice and subsequent PA level, for example, preference for PA resources [42]). Often, one article reported multiple environmental attributes that were coded under the same environmental category (e.g., ‘absence of litter’ and ‘presence of trees’ would both fall under the common category of ‘greenery and aesthetically pleasing scenery’). Similarly, one article may have reported multiple PA outcomes related to the same environmental attribute (e.g., ‘total walking’ and ‘total PA’ with ‘crime/personal safety’). Four distinct findings would be extracted from these two examples. We then cross-examined other articles from the same dataset (e.g., Senior Neighborhood Quality of Life Study (SNQLS) [43–48]) to avoid duplication of extracted data.

### Quantification of buffer effects and/or moderators

Findings from studies reporting associations for a given combination of environmental attribute and PA outcome for multiple environmental buffer sizes or values of a moderator were assigned fractional weights totalling 1.0 (per study). Associations from studies reporting on multiple buffer sizes were each assigned a fractional weight equal to 1 divided by the number of buffer sizes. For example, Nathan and colleagues examined associations for two buffer sizes (400 m and 800 m). They found a nil association for 400 m buffers around the home between access to social infrastructure and total walking and a positive one for 800 m buffers [49]. In this case, a resulting score of 0.5 nil (400 m buffer) and 0.5 positive associations (800 m buffer) was recorded. For studies that examined moderator effects, associations were reported as main effects only if the findings across all examined values of the moderator were consistent in direction and statistical significance. When this did not occur, weights were assigned for each examined value of the moderator dependent upon the (approximate) proportion of the total sample represented by the subgroup of participants. For example, Jefferis and colleagues found a positive association between access to social and leisure activities for males and a nil association for females [50]. Since males represented 65% of the total sample, a fractional weight of 0.65 was assigned to the positive association and a 0.35 weight for the nil association (females). For studies that used a continuous measure of moderators, associations recorded at the average value of the moderator were assigned a weight of 0.60, while those above and below the mean were each assigned a weight of 0.20 (i.e., ±1 standard deviation (SD)). This follows the logic that, if data are normally distributed, ≈20% of values would fall below and 20% above 1SD (accounting for some uncertainty around the value of the moderator at ±1 SD). When an association was moderated by numerous factors, weights were assigned according to the aforementioned procedure and, again, with all fractional weights totalling 1.0.

### Article quality and sample size assessment

A checklist for article quality assessment was developed based on ten set criteria [with a maximum total quality score of 9] (Additional file 2): (1) study design [score: cross-sectional = 0, longitudinal = 1, quasi-experimental = 2]; (2) study areas or participant recruitment stratified by key environmental attributes [yes = 1, no = 0]; (3) adequate participant response rate (≥60%) [51] or evidence of a representative sample [yes = 1, no = 0]; (4) outcome measures valid and reliable based on published metric properties of the instrument used [52], or outcome measures well-established in the field [yes = 1, no = 0]; (5) adjustment for key socio-demographic covariates (at least age, sex, and education considered) [yes = 1, no = 0]; (6) adjustment for self-selection [yes = 1, no = 0]; (7) appropriate analytical approach – adjustment for clustering (if needed) [yes = 1/3, no = 0]; (8) appropriate analytical approach – accounting for distributional assumptions [yes = 1/3, no = 0]; (9) appropriate analytical approach – analyses conducted and presented correctly [yes = 1/3, no = 0]; and (10) did not inappropriately categorise continuous environmental exposures [yes = 1, no = 0]. Higher scores reflect higher quality: 0–3.5 (low quality), 3.6–5.9 (moderate quality), and ≥6 (high quality). Apart from providing descriptive information on article quality, these scores were also used as weights in the meta-analyses described below so that higher quality articles had a greater contribution to the synthesis of findings.

To account for sample size in the meta-analyses, the following weights were assigned to findings: 0.25 (≤100 participants), 0.50 (101–300 participants), 1.00 (301–500 participants), 1.25 (501–1000 participants), 1.50 (1001–2500 participants), and 1.75 (>2500 participants). In two instances, different sample weights were applied to findings derived from the same study because of differences in sample size between objectively assessed and perceived environmental exposures [53] and between data collection periods [54]. A thorough rationale for these weights can be found elsewhere [27].

### Data synthesis

Each separate positive, negative, and nil association between a built environmental attribute and PA outcome (either total PA, total MVPA, or total walking) was tallied [27] and, where appropriate, multiplied by a buffer-size or moderator-related fractional weight (see section above on ‘Quantification of buffer effects and/or moderators’). All of these findings were then included into a “Total PA” outcome, which was then stratified by PA measurement type (objective or self-report). “Total walking” was considered separately and was based on findings from self-report walking only, as the majority of associations between built environmental attributes and objectively assessed walking was insufficiently examined (i.e., <5 findings) (Additional file 3). The findings related to both “Total PA” and “Total walking” were then stratified by environmental measurement type (objective or perceived).

Due to the large range of different environmental and PA measures reported in the selected articles, it was not possible to conduct a ‘traditional’ meta-analysis involving the estimation of pooled effect sizes and their 95% confidence intervals. Hence, to quantitatively synthesise the findings, a meta-analytic approach was applied to define conservative estimates of *p*-values for each examined combination of environmental attribute and PA outcome [27]. These *p*-values represented the probability of observing a certain distribution of findings (e.g., 4 positive, 2 nil, and 0 negative associations) under a null hypothesis of no association. These computations were undertaken accounting for: (1) sample size and quality scores of included articles (see previous section); (2) sample size score only; (3) article quality score only; and (4) neither – with 2–4 providing a sensitivity analysis on how each of these influenced meta-analytical findings. Associations of specific built environmental attributes with PA outcomes were examined by type of PA and environmental measurement (objective and perceived) only when ≥3 articles provided data on each type of measure, consistent with recommendations for meta-analyses from Cochrane’s database of systematic reviews [55]. Environment-PA outcome associations totalling <5 separate findings were deemed as insufficiently studied to reach a robust conclusion [56].

To perform the meta-analyses, we first assigned a z-value to each separate built environmental attribute and PA finding, specifically: 1.96 for a positive; −1.96 for a negative; and 0 for a nil finding [27]. We then calculated a summary two-tailed *p*-value using Rosenthal’s approach [57], reporting a summary weighted z-value and its associated two-tailed probability value as detailed in Cerin et al.’s recent systematic review and meta-analysis of built environmental correlates of active transport [27]. For the sensitivity analysis aforementioned, we also calculated non-weighted z values and their associated two-tailed *p*-values. All computations were performed in a Microsoft Excel spreadsheet using algorithms developed by the authors. *P*-values were interpreted as follows: .05 to .01 – evidence of an association; <.01 – strong evidence of an association; and <.001 – very strong evidence of an association [58].

All examined moderators of environment-PA associations were counted and summarised by the category of moderator tested. This included multiple factor (i.e., higher-order) moderating effects, tested formally (e.g., [59]) or not (e.g., [20]). Reporting higher-order moderating effects by moderator category resulted in findings being counted multiple times, i.e., once for each moderator category. For example, Chen and colleagues reported sex by employment status interactions, so the findings were reported twice, once under sex and once under employment status as moderators [20]. For this reason, the total number of moderating effects reported in this review is greater than the number of moderating effects reported in the articles.

## Results

Of 19,005 potential articles, we fully read 530 and included 100 in our analysis – two of which were PhD theses [60, 61] (Fig. 1). From the 100 articles, 1553 individual associations were extracted.

### Characteristics of included articles

Details of article characteristics can be found in Table 1. Cross-sectional studies accounted for 94% of articles, with five papers reporting longitudinal [61–65] and one quasi-experimental [66] findings. Almost half of all studies (46%) were based in North America, followed by Europe (22%), Asia (16%), Oceania (13%), two studies from South America [23, 24], and one pilot study from South Africa [25]. Regarding countries, the USA (36%), Australia (13%), the UK (10%), and Canada (10%) conducted the most research. Only 3% of articles specifically studied rural participants [67–69], about half researched those from urban settings (56%), and 32% from a mixture of both.

Sample sizes ranged from 44 [25] to 69,253 [70], with over a third of samples (36%) regarded as small (i.e., ≤300 participants). Recruitment of neighbourhoods was stratified by key built environmental attributes in 57% of articles; urbanisation being the most prevalent (22%), followed by area-level socio-economic status (SES) and walkability combined (12%), and area-level SES and urbanisation combined (10%). Neighbourhoods were most frequently defined objectively by administrative and census areas. When buffers were applied to define neighbourhoods, a 400-500 m radius was the most frequently used buffer size. A 10–20 min’ walk from home was the most commonly reported perceived neighbourhood definition.

Overall, 66% of articles used a validated or established PA measure. Almost three quarters of articles reported findings based on older adults’ self-reported PA (74%). The most commonly reported PA outcome was total walking (55%). Approximately 55% of PA outcomes were operationalised as continuous measures (e.g., minutes/day) and 56% of self-report PA outcomes were measures of total walking. The most commonly reported objective PA outcome was total MVPA (47% of objective PA articles).

Overall, the most researched attributes were crime-related personal safety (41%), residential density/urbanisation (35%), greenery and aesthetically pleasing scenery (33%), access to/availability of parks/public open space (30%), pedestrian-related traffic safety (28%), and access to/availability of shops/commercial destinations (26%). Similarly, the most investigated perceived attributes were crime-related personal safety (38%), greenery and aesthetically pleasing scenery (28%), pedestrian-related traffic safety (25%), access to/availability of parks/public open space and recreational facilities (both 15%). The most commonly evaluated objectively assessed environmental attributes were urbanisation/residential density (21%), and access to/availability of shops/commercial destinations and of parks/public open space (17% each).

Thirty-nine percent of articles investigated moderating effects on associations between built environmental attributes and total PA. At the individual level, socio-demographics were the most frequently examined moderator, and at the environmental level, residential density/urbanisation was the most frequently reported moderator. (Note: Full details of article characteristics can be found in Additional file 1).

### Article quality

Only 9% of articles were deemed of high quality, 55% of moderate quality, and the remaining 36% of low quality. Key socio-demographic covariates (i.e., at least age, sex, and education) were adjusted for in 66% of articles; much fewer analyses adjusted for self-selection (12%) (Table 2) [62, 69, 71–80]. Approximately three quarters of the included articles conducted appropriate analyses (76%). (Note: Full details of article quality can be found in Additional file 3).

**Table 2:** Summary of article quality assessment (*N* = 100)

Quality-assessment item [score]	%
1. Study design [cross-sectional: 0; longitudinal: 1; quasi-experimental: 2]	
cross-sectional	94
longitudinal	5
quasi-experimental	1
2. Study areas or participant recruitment stratified by key environmental attributes [1]	56
3. Response rate ≥ 60% or sample representative of the population [1]	32
4. Physical activity measures (outcomes) valid, or well-established in the field [1]	66
5. Analyses adjusted for key socio-demographic covariates (at least age, sex, and education considered) [1]	66
6. Analysis adjusted for self-selection [1]	12
7. Analytical approach – adjustment for clustering (if needed) [1/3]	58
8. Analytical approach – accounting for distributional assumptions [1/3]	84
9. Analytical approach – analyses conducted and presented correctly [1/3]	76
10. Did not, inappropriately, categorise continuous environmental exposure/s [1]	74
Total quality score [theoretical range: 0–9]; mean ± SD	3.9 ± 1.3

*Notes: SD* Standard deviation

### Built environmental correlates of older adults’ PA

#### Total PA

There was very strong evidence that neighbourhood walkability (*p* < .001), overall access to destinations and services (*p* < .001) and recreational facilities (*p* < .001), and crime-related personal safety (*p* < .001) were positively associated with older adults’ total PA (Table 3). Moreover, there was strong evidence of positive associations between total PA and access to both parks/public open space (*p* = .002) and shops/commercial destinations (*p* = .006), the presence of greenery and aesthetically pleasing scenery (*p* = .004), and walk-friendly infrastructure (*p* = .009). In addition, there was evidence that access to public transport was positively associated with total PA (*p* = .016). No other significant associations were found in relation to built environmental attributes and total PA.

**Table 3:** Associations of environmental attributes/correlates with older adults’ physical activity by physical activity outcomes

Environmental attribute	Total PA^1^					Total walking only				
	P	Ø	N	p_a_	D_a_	P	Ø	N	p_a_	D_a_
Walkability	12.33	6.67	0	**<.001**	**P**	4.37	3.63	0	**.001**	**P**
Residential density/urbanisation	11.53	33.50	12.97	.394	Ø	8	14.50	3.50	**.036**	**P**
Street connectivity	8.80	26.06	2.14	.094	Ø	5.80	13.20	2	.185	Ø
*Access to/availability of destinations & services*										
Overall access to destinations & services	12.55	38.15	0.50	**<.001**	**P**	6.93	25.57	0.50	**.009**	**P**
Land-use mix—destination diversity	5.68	19.32	2	.148	Ø	1	8	2	.439	Ø
Shops/commercial	9.96	57.04	0	**.006**	**P**	8.58	23.42	0	**.001**	**P**
Food outlets	0.72	21.28	1	.932	Ø	0.72	6.28	1	.873	Ø
Government/finance services	0.33	11.67	0	.834	Ø	0	6	0	1.00	Ø
Education	0.31	11.69	0	.765	Ø	0.14	2.85	0	.826	Ø
Health & aged care	4.61	26.39	1	.275	Ø	3.61	7.39	1	.191	Ø
Religious	0	8	0	1.00	Ø	0	1	0	1.00	Ø
Public transport	7.50	25.50	1	**.016**	**P**	5.50	11.50	1	**.011**	**P**
Parks/public open space	11.29	47.54	0.17	**.002**	**P**	6.05	23.78	0.17	**.014**	**P**
Recreational facilities	13.34	39.66	0	**<.001**	**P**	3.07	15.93	0	.135	Ø
Social recreational facilities	4.15	25.95	0	.105	Ø	1.50	10.50	0	.413	Ø
Other destinations	0	3	0	1.00	Ø	-	-	-	-	-
*Infrastructure & streetscape*										
Overall access to cycle/walk-friendly infrastructure	1	9	0	.612	Ø	0	3	0	1.00	Ø
Walk-friendly infrastructure	9	21.49	1.51	**.009**	**P**	5	15	0	**.042**	**P**
Cycle-friendly infrastructure	0	5	0	1.00	Ø	0	3	0	1.00	Ø
No physical barriers (e.g., hills)	5	20.40	2.60	.208	Ø	2	14.40	0.60	.384	Ø
Pavement/footpath quality	3	6	1	.155	Ø	2	5	0	.169	Ø
Street lighting	3	6	0	.059	Ø	3	4	0	.042	P
Greenery & aesthetically pleasing scenery	13.01	45.49	0.5	**.004**	**P**	10.51	19.49	0	**.002**	**P**
Pollution (air)	0	5	0	1.00	Ø	0	4	0	1.00	Ø
*Safety*										
Traffic/pedestrian safety	7	47	3	.463	Ø	5	25	3	.705	Ø
Crime/personal safety	20.52	50.48	4	**<.001**	**P**	10.49	28.01	2.50	**.027**	**P**

*Notes:* ^1^Objective and self-report total PA (including total walking) combined. P = positive association; Ø = nil association; N = negative association; *p* = *p*-value; D = direction of association supported by the data; subscript “a” = fully adjusted (for sample size and article quality). In bold font: statistically significant evidence of a directional association that has been sufficiently studied (i.e., ≥5 findings reported on specific combinations of environmental exposure and PA variables)

#### Total walking

We found strong evidence for positive associations between older adults’ total walking and neighbourhood walkability (*p* = .001), access to/availability of shops/commercial destinations (*p* = .001) and overall destinations and services (*p* = .009) and more greenery and aesthetically pleasing scenery (*p* = .002) (Table 3). We also found evidence that access to/availability of neighbourhood public transport (*p* = .011) and parks/public open space (*p* = .014), crime/personal safety (*p* = .027), residential density (*p* = .036), walk-friendly infrastructure (*p* = .042), and street lighting (*p* = .042) were positively associated with total walking. No significant associations were found for the remaining 15 built environmental attributes (Table 3).

### Built environmental correlates of older adults’ PA by measurement method

#### PA measurement type (objective and self-report)

Irrespective of the PA measurement type used, neighbourhood walkability (both *p* < .001) and overall access to destinations and services (both *p* < .01) were positively associated with older adults’ total PA (Table 4). Seven other positive associations between attributes of the built environment and PA were PA-measurement dependent. Five positive associations were specific to self-reported total PA, namely: greenery and aesthetically pleasing scenery (*p* = .001), access to shops/commercial destinations (*p* = .002), parks/public open space (*p* = .002), recreational facilities (*p* = .002) and public transport (*p* = .006). Two remaining positive associations were in relation to objectively assessed total PA only, specifically: walk-friendly infrastructure (*p* = .031) and destination diversity (land use mix) (*p* = .019).

**Table 4:** Associations of environmental attributes/correlates with older adults’ physical activity by physical activity measurement method (objective and self-report)

Environmental attribute	Total PA									
	Objective					Self-report				
	P	Ø	N	p_a_	D_a_	P	Ø	N	p_a_	D_a_
Walkability	5.96	2.04	0	**<.001**	**P**	6.37	4.63	0	**<.001**	**P**
Residential density/urbanisation	1	7	0	.377	Ø	10.53	26.50	12.97	.240	Ø
Street connectivity	3	7.86	0.14	.262	Ø	5.71	18.20	2	.215	Ø
*Access to/availability of destinations & services*										
Overall access to destinations & services	3.89	8.31	0	**.005**	**P**	8.66	29.84	0.50	**.004**	**P**
Land-use mix—destination diversity	3.17	8.83	0	**.019**	**P**	2.51	12.49	2	.884	Ø
Shops/commercial	1.38	26.62	0	.507	Ø	8.58	29.42	0	**.002**	**P**
Food outlets	0	13	0	1.00	Ø	0.72	8.28	1	.884	Ø
Government/finance services	0.34	5.66	0	.377	Ø	0	6	0	1.00	Ø
Education	0.17	6.83	0	.818	Ø	0.14	4.86	0	.845	Ø
Health & aged care	1	18	0	.612	Ø	3.61	8.39	1	.206	Ø
Religious	0	5	0	1.00	Ø	0	3	0	1.00	Ø
Public transport	1	12	0	.520	Ø	6.50	13.50	1	**.006**	**P**
Parks/public open space	1.75	14.25	0	.296	Ø	9.54	33.29	0.17	**.002**	**P**
Recreational facilities	4.29	16.71	0	.056	Ø	9.05	22.95	0	**.002**	**P**
Social recreational facilities	2.65	12.35	0	.118	Ø	1.50	13.50	0	.432	Ø
*Infrastructure & streetscape*										
Overall access to cycle/walk-friendly infrastructure	1	5	0	.529	Ø	0	4	0	1.00	Ø
Walk-friendly infrastructure	3	3	0	**.031**	**P**	6	18.49	1.51	.059	Ø
No physical barriers (e.g., hills)	3	5	1	.135	Ø	2	15.40	1.60	.631	Ø
Greenery & aesthetically pleasing scenery	1.50	15	0.50	.741	Ø	11.51	30.49	0	**.001**	**P**
*Safety*										
Traffic/pedestrian safety	2	14	0	.408	Ø	5	33	3	.737	Ø
Crime/personal safety	3	8	0	.063	Ø	17.52	42.48	4	**.001**	**P**

*Notes:* P = positive association; Ø = nil association; N = negative association; *p* = *p*-value; D = direction of association supported by the data; subscript “a” = fully adjusted (for sample size and article quality). In bold font: statistically significant evidence of a directional association that has been sufficiently studied (i.e., ≥5 findings reported on specific combinations of environmental exposure and PA variables)

### Environmental attribute measurement type (objective and perceived)

#### Total PA

For nine out of 18 environmental exposures, associations with total PA differed by environmental measurement type (Table 5). For five environmental attributes, positive associations with total PA were found with perceived but not objectively assessed measures. Perceptions of crime-related personal safety (*p* < .001), access to/availability of recreational facilities (*p* < .001), access to/availability of parks/public open space (*p* = .003), greenery and aesthetically pleasing scenery (*p* = .003), and destination diversity (land-use mix) (*p* = .002) were all positively associated with total PA. Objectively assessed access to/availability of shops/commercial destinations (*p* = .006) and public transport (*p* = .004), presence of walk-friendly infrastructure (*p* = .028), and absence of physical environmental barriers (e.g., hills) (*p* = .048) were all positively associated with total PA, whereas associations with these attributes were non-significant when using perceived measures.

**Table 5:** Associations of environmental attributes/correlates with older adults’ physical activity by physical activity and environmental measures (objective and perceived)

Environmental attribute	Total PA^1^					Total walking only				
	P	Ø	N	p_a_	D_a_	P	Ø	N	p_a_	D_a_
Walkability	12.33	6.67	0	**<.001**	**P**	4.37	3.63	0	.001	P
*Objective*	9.05	6.95	0	<.001	P	-	-	-	-	-
*Perceived*	3	0	0	.003	P	-	-	-	-	-
Residential density/urbanisation	11.53	33.5	12.97	.394	Ø	8	14.5	3.5	.036	P
*Objective*	10	18.50	11.50	.388	Ø	7	6.50	3.50	**.032**	**P**
*Perceived*	1.53	15	1.47	.855	Ø	1	8	0	**.652**	**Ø**
Street connectivity	8.71	26.06	2.14	.094	Ø	5.71	13.20	2	.185	Ø
*Objective*	2.80	14.20	1	.366	Ø	1.80	9.20	1	.543	Ø
*Perceived*	6	11.86	1.14	.076	Ø	4	4	1	.210	Ø
*Access to/availability of destinations & services*										
Overall access to destinations & services	12.55	38.15	0.5	**<.001**	**P**	6.93	25.57	0.5	**.009**	**P**
*Objective*	3.76	12.24	0	.003	P	3.43	9.57	0	**.004**	**P**
*Perceived*	8.79	25.91	0.50	.008	P	3.50	16	0.50	**.277**	**Ø**
Land-use mix—destination diversity	5.68	19.32	2	.148	Ø	1	8	2	.439	Ø
*Objective*	1.17	10.83	2	**.504**	**Ø**	-	-	-	-	-
*Perceived*	4.51	8.49	0	**.002**	**P**	-	-	-	-	-
Shops/commercial	9.96	57.21	0	**.006**	**P**	8.58	23.42	0	.001	P
*Objective*	8.25	34.75	0	**.006**	**P**	7.08	12.92	0	**<.001**	**P**
*Perceived*	1.71	21.29	0	**.475**	**Ø**	1.50	10.50	0	**.422**	**Ø**
Food outlets	0.72	21.28	1	.932	Ø	0.72	6.28	1	.873	Ø
*Objective*	0.72	14.28	0	.685	Ø	-	-	-	-	-
*Perceived*	0	7	1	.521	Ø	-	-	-	-	-
Education	0.31	11.69	0	.765	Ø	0.14	2.85	0	.826	Ø
*Objective*	0.31	8.69	0	.727	Ø	-	-	-	-	-
*Perceived*	0	3	0	1.00	Ø	-	-	-	-	-
Health & aged care	4.61	26.39	1	.275	Ø	3.61	7.39	1	.191	Ø
*Objective*	4	24	1	.382	Ø	-	-	-	-	-
*Perceived*	0.61	2.39	0	.290	Ø	-	-	-	-	-
Public transport	7.5	25.6	1	.013	P	5.5	11.5	1	.011	P
*Objective*	6.50	12.50	0	**.004**	**P**	5.50	5.50	0	**<.001**	**P**
*Perceived*	1	13	1	**.918**	**Ø**	0	6	1	**.501**	**Ø**
Parks/public open space	11.29	47.54	0.17	.002	P	6.05	23.78	0.17	.014	P
*Objective*	4.42	28.58	0	**.083**	**Ø**	4.42	13.58	0	**.035**	**P**
*Perceived*	6.87	18.96	0.17	**.003**	**P**	1.63	10.20	0.17	**.201**	**Ø**
Recreational facilities	13.34	39.66	0	<.001	P	3.07	15.93	0	.135	Ø
*Objective*	4.58	21.42	0	**.092**	**Ø**	0.29	6.71	0	**.848**	**Ø**
*Perceived*	8.76	18.24	0	**<.001**	**P**	2.78	9.22	0	**.050**	**P**
Social recreational facilities	4.15	25.95	0	.105	Ø	1.5	10.5	0	.413	Ø
*Objective*	3.50	14.50	0	.094	Ø	1.50	4.50	0	.291	Ø
*Perceived*	0.65	11.45	0	.687	Ø	0	6	0	1.00	Ø
*Infrastructure & streetscape*										
Walk-friendly infrastructure	9	21.49	1.51	.009	P	5	15	0	.042	P
*Objective*	5	9	0	**.028**	**P**	3	7	0	.103	Ø
*Perceived*	4	12.49	1.51	**.137**	**Ø**	2	8	0	.222	Ø
No physical barriers (e.g., hills)	5	20.40	2.61	.208	Ø	2	14.40	0.61	.384	Ø
*Objective*	5	8.40	1.60	**.048**	**P**	2	4.40	0.60	.227	Ø
*Perceived*	0	12	1	**.629**	**Ø**	0	10	0	1.00	Ø
Greenery & aesthetically pleasing scenery	13.01	45.49	0.5	.004	P	10.51	19.49	0	.002	P
*Objective*	3	18	0	**.252**	**Ø**	3	9	0	**.199**	**Ø**
*Perceived*	10.01	27.49	0.50	**.003**	**P**	7.51	10.49	0	**<.001**	**P**
*Safety*										
Traffic/pedestrian safety	7	47	3	.463	Ø	5	25	3	.705	Ø
*Objective*	1	13	3	.407	Ø	0	11	3	**.150**	**Ø**
*Perceived*	6	34	0	.126	Ø	5	14	0	**.043**	**P**
Crime/personal safety	20.63	50.58	3.99	**<.001**	**P**	10.49	28.01	2.5	**.027**	**P**
*Objective*	4	5.50	2.50	**.510**	**Ø**	3	5	2	**.627**	**Ø**
*Perceived*	16.52	44.98	1.50	**<.001**	**P**	7.49	23.01	0.50	**.012**	**P**

*Notes:* ^1^Objective and self-report total PA (including total walking) combined. P = positive association; Ø = nil association; N = negative association; p = *p*-value; D = direction of association supported by the data; subscript “a” = fully adjusted (for sample size and article quality). In bold font: evidence of a difference between environmental measures of an association between a sufficiently studied exposure and PA variable (i.e., ≥3 articles’ reported findings on specific combinations of environmental exposure and physical activity variables)

#### Total walking

There were five positive PA associations with objectively measured environment variables only and four others with measures based on perceptions only. Evidence of a positive association with total walking was found for perceived measures of neighbourhood greenery and aesthetically pleasing scenery (*p* < .001), crime/personal safety (*p* = .012), traffic-safety (*p* = .043), and access to/availability of recreational facilities (*p* = .050). Regarding objectively measured environmental attributes, access to/availability of shops/commercial destinations (*p* < .001), public transport (*p* < .001), overall destinations and services (*p* = .004), parks/public open space (*p* = .035), and residential density/urbanisation (*p* = .032) were all positively related to total walking.

### Sensitivity analyses

None of the significant correlates of total PA, objective total PA or self-report total PA differed based on any adjustment (partial or none) (Additional file 4). Regarding total walking, only two significant correlates differed based on adjustment, namely: residential density/urbanisation (fully-adjusted: *p* = .036; unadjusted: *p* = .055) and walk-friendly infrastructure (fully-adjusted: *p* = .042; article quality-adjusted: *p* = .057). In addition, some built environmental attributes were significant when unadjusted, but not when taking into account sample size and/or article quality. These were street lighting (total PA and self-report total PA), street connectivity (total walking), and crime/personal safety (objective total PA).

### Moderators of environment-PA associations

Sixteen moderators of built environmental attribute-PA associations were examined in 39 articles (Additional file 5). The most frequently examined moderators by number of articles were sex (7 articles), health status/functionality (7 articles), residential density/urbanisation (6 articles), and SES/area-level income (5 articles). Buffer size (121 findings) and sex (83 findings) were the most frequently examined by estimating regression interaction terms. The direction of effects for all significant interaction terms was inconsistent (Additional file 5).

## Discussion

In the last decade, world bodies have been advocating the importance of healthy ageing and the enabling role played by PA and built environments (e.g., [81]). As a result, the number of published articles on the associations between built environmental attributes and older adults’ PA increased over three times since the last systematic review in 2011 [15]. Moreover, there was a greater percentage of articles from outside of North America, with notable increases in research conducted in Asia and Europe, which expanded the range of examined geographical settings and cultures. We undertook a systematic review and applied a meta-analytic procedure to statistically identify built environmental attributes related to total PA and total walking, stratifying by measurement method.

In general, while the findings from Van Cauwenberg and colleagues’ systematic review published in 2011 were inconclusive [15], we found strong evidence of positive associations between walkability, access to destinations and services, personal safety from crime and PA. Also, while the relatively small number of articles included in Van Cauwenberg and colleagues’ review [15] precluded the examination of differences in findings by measurement method, this review and meta-analysis revealed important differences in associations when using objective versus perceived measures of environmental attributes and when using self-report versus objective measures of PA. These new findings and their implications are discussed in detail below.

### Built environmental correlates of older adults’ PA

#### Walkability and access to destinations/services

We found strong to very strong evidence supporting the benefits of neighbourhood walkability on total PA and walking, regardless of measurement method. Two of the three components comprising walkability were found to individually relate to PA: strong evidence supported the association between access to destinations and services and total PA, for both objective and perceived environmental and PA measurement types. Evidence was also found supporting the impact of access to destinations and services and residential density on total walking, particularly when these attributes were measured objectively. Our findings highlight the importance of having local neighbourhood destinations for older adults to not only walk to and walk around, but to also engage in other types of PA. Furthermore, local destinations may provide a location for social activity and engagement, potentially reducing risk of social isolation and loneliness [82].

Although easier access to destinations and services tend to be highly correlated with greater residential density [83], our findings suggest that walking may be the only type of PA positively related to residential density. High levels of residential density may not be conducive to other forms of active transport, such as cycling. One study has found that Flemish older adults living in urban areas were less likely to cycle everyday than those living in semi-urban (i.e., less dense) areas [84]. While walking is the most popular type of PA that older adults participate in [9], cycling is also a popular PA mode in European countries such as the Netherlands, Denmark, and Germany, where cycling levels remain high even among older people [85]. Future research examining the differential influence of residential density on different types of PA as well as identifying the optimal threshold of density for supporting all types of PA will be important for informing planning policy and practice [86].

In terms of access to specific types of destinations in the neighbourhood, we found evidence supporting shops and commercial destinations, public transport, parks and public open space, and recreational facilities as possible facilitators of PA. No evidence was found for the seven other destination types examined. Overall, this is in line with the work of others who highlight that certain types of destinations may be more conducive to higher PA levels than other destination types [15, 27].

Shops/commercial destinations and public transport, particularly for objectively assessed measurement types, were positively associated with total walking and total PA, specifically self-reported measures of total PA. The importance of shops and commercial destinations for PA is consistent with findings highlighting that shopping is the most prevalent reason for older adults leaving their homes [87], and thus an important part of daily life. Therefore, ensuring neighbourhoods have ease of access to shops means that health-enhancing levels of PA can be incorporated into daily living. Availability and access to public transport not only facilitates PA levels but has the potential to also reduce car dependence [88] with co-benefits of environmental sustainability [89]. For older adults especially, access to public transport enables those who are not confident with driving or no longer able to drive to still travel outside of home, thus maintaining their mobility and reducing risk of loneliness [90].

We found strong to very strong evidence for parks and public open space and recreational facilities as correlates of total PA, particularly for self-reported types of measurement. This is consistent with findings in adults [91]. When comparing environmental measurement methods, evidence was found for positive associations between total PA and perceived, but not objectively assessed, access to parks, public open space and recreational facilities. Access to parks and public open space was also found to be positively associated with total walking. However, when comparing the environmental measurement type, it was the objectively assessed measures showing a positive association with total walking, not perceptions. Here, it should be noted that most of the objectively assessed positive findings were from studies based in Portland, USA [64, 92, 93], a city renowned for its walk-friendliness and management of parks in the presence of urban growth [94, 95]. Having accessible parks and public open space and recreational facilities in local areas may be beneficial beyond PA, as green spaces and visual cues of nature in parks may impart further psychological benefits on individuals [96, 97]. Moreover, both destinations provide an opportunity for fostering social connectedness/activities (e.g., a walk with friends in a park and a game of squash at a recreational facility).

#### Infrastructure and streetscape

Pedestrian-friendly infrastructure, particularly when measured objectively, was found to be positively associated with both total PA and total walking. This reflects qualitative [98] and experimental research [99] findings highlighting the importance of pavements/footpaths and other infrastructure, such as benches for resting, for older adults’ PA. Ensuring the provision of walk-friendly infrastructure, especially along routes to destinations within the neighbourhood, may be particularly pertinent. It is plausible that the relationship between walk-friendly infrastructure and PA may differ based on physical functionality. However, we identified only one study that had examined this, finding no difference in the associations [100].

We found evidence supporting a positive association between street lighting and total walking only. This highlights the importance of providing street lighting along pedestrian infrastructure so that its use is not dependent on the time of day. For older adults especially, ensuring neighbourhoods are well-lit at night may also contribute to a heightened sense of safety from crime [98]. This is because street lighting helps contribute to natural surveillance by allowing pedestrians to be seen.

We found no evidence of an association between pavement/footpath quality and PA. This is in contrast to qualitative research findings indicating quality of infrastructure to be particularly pertinent in facilitating PA among older adults [98, 99, 101]. Our findings may be explained by the diversity of measures used and/or lack of clear definition of pavement/footpath quality. For example, what constituted ‘footpath quality’ ranged from “curb quality” (objectively assessed) [47] to “quality and maintenance of sidewalks” (as perceived by study participants) [24].

Strong evidence supported the role of greenery and aesthetically pleasing scenery on levels of total PA (especially self-reported measurement types) and total walking. These findings are in line with recent research highlighting the importance of green, clean, and attractive neighbourhoods and streetscapes in facilitating PA [98, 102]. When stratifying by environmental measurement type, only perceived measures were found to be significant. Beyond facilitating PA, it is plausible that there are synergistic benefits of streetscape trees and vegetation, for example, in reducing urban heat island effect [103] and air pollution [104] – environmental factors linked with premature mortality [105] and global disease burden [106]. Following design principles of ‘tactical urbanism’, which are low cost interventions to make areas more attractive and pedestrian-friendly [107], environmental modifications such as planting trees and flora are micro-scale interventions that can be more easily implemented than macro-level interventions to street design and layout.

#### Safety

Safety from crime, especially when perceived measures were used, was found to be positively associated with total PA (primarily self-reported measures) and total walking. This adds to the evidence base as previous research in older adults has mostly shown inconsistent findings [31, 108]. Our findings are in line with the notion that perceptions of crime have more influence on behaviour (e.g., leaving the home) than objective crime rates [109]. This speaks in favour of interventions aimed at positively changing perceptions of safety (when appropriate) and encouraging older people to get out of home. This may be particularly important as the frequency of daily out of home trips is predictive of PA participation in this demographic [87].

Overall, we found no evidence to support the relationship between traffic-related safety and total PA and total walking. However, when only perceived measures of traffic safety were considered, there was evidence of a positive association with total walking only. It is possible that older adults may have no choice other than participating in walking near heavy neighbourhood traffic because they do not own a car and/or have limited access to public transport [73]. It may be that a substantial amount of walking and PA in older adults comes from actively travelling to and from destination-rich areas where traffic is typically heavy [27].

### Differences in built environmental correlates by type of PA measurement method

We found more significant environmental correlates for self-reported PA than for objectively measured PA. One reason for this may relate to common method bias associated with self-reported PA and environmental attributes – systematic error variance introduced by measurement methods that do not accurately assess the constructs they represent and may be due to factors such as social desirability [110]. Another reason may be that the environmental attributes measured in these studies primarily influence walking behaviours that may be more easily measured by self-reports than by accelerometry. An additional reason may relate to issues with the accelerometry-based operationalisation of older adults’ MVPA. Sixteen of the 28 reviewed articles reporting objective PA findings used accelerometer cut-points and half of those applied an MVPA cut-point of 1952 accelerometer counts per min derived for adults [111]. As older adults have a lower MVPA cut-point due to lower resting metabolic rates [112], using the adult accelerometer cut-point likely resulted in lower estimates of MVPA, potentially masking associations. To accurately classify different intensities of older adults’ PA, future research using objectively assessed PA should be underpinned by appropriate cut-points.

### Differences in built environmental correlates by type of environmental measurement method

Overall, there were numerous differences in the associations between built environmental attributes and total PA and walking, based on type of environmental measure. Attributes that can be classed within the functional (e.g., pedestrian infrastructure) and destination domains in Pikora’s framework tended to be significantly related to PA when objectively assessed [113]. In contrast, those attributes that fall within the safety and aesthetics domains were associated with PA when perceived measures were used. This may be explained by attributes within safety and aesthetics domains being more subjective in their interpretation and thus depend on perceptions that may vary greatly between individuals. Attributes related to function and destinations are more objective and, hence, are associated with lower levels of interpersonal differences in perceptions (e.g., a pavement is either present or it is not).

Effects were generally stronger for associations between the perceived environment and PA, which is consistent with previous research [114]. Unlike the objective environment, perceptions of the same neighbourhood environment can greatly differ across individuals due to differences in socio-demographics (e.g., socioeconomic status), preference, experience, culture and/or amount of walking in the neighbourhood [30]. Regular walkers may have more accurate perceptions of their local environments. Moreover, perceived measures often define neighbourhood in terms of time to reach a destination (e.g., 10–20 min’ walk from home) [40, 41], rather than set distances (e.g., objective 400 m home-centred buffers), and therefore may be more closely aligned with the individual and their own definition of ‘neighbourhood’.

### Implications for future research and planning policy/practice

Socio-ecological models of health behaviour underpin the majority of research undertaken in the built environment and PA field. One of the key tenets of this approach is its emphasis on the importance of behaviour specificity [12], and for PA this means considering the domain in which PA was accrued. While taking a behavioural perspective allows for the pathways or mechanisms through which the built environment influences PA to be understood, it is possible that built environmental attributes may relate differently to different behaviours [115]. Instead, a public health perspective examining built environmental attributes associated with total PA focuses on the identifications of environmental attributes enabling health-enhancing levels of PA, which is accrued across all domains. Notably, nearly all built environmental correlates of older adults’ total PA were also identified as being environmental correlates of either active transport [27] and/or leisure-time PA [116], thus explaining the behavioural pathways through which the built form impacts on total levels of PA. There was one exception, however, with crime/personal safety being positively associated with total PA, but no evidence found for a relationship with either active transport [27] or leisure-time PA [116]. Other behavioural or psychosocial factors may explain the associations between crime/personal safety and total PA. Given the medium to strong evidence of these associations, it is especially important for future research to unpack the mechanisms through which crime/personal safety relates to total health-enhancing PA in order to better inform the implementation of suitable interventions. For example, a better understanding of fear of crime and assessment of the emotional rather than cognitive response to crime may be warranted [108]. Moderators of the relationship between safety and PA that warrant further consideration may include self-efficacy and physical functioning/capacity [117].

### Research design issues

Longitudinal and quasi-experimental studies are needed to establish causal relationships between the built environment and PA. Insofar as possible, future research designs would also benefit from assessing and adjusting for residential self-selection to account for biases at the individual level (e.g., an older adult who enjoys PA or chooses to live near a park) and thus enabling, to a certain extent, the controlling of reverse causation. The findings of this review may help inform researchers involved with natural experiments on what environmental attributes to measure, given the environmental manipulation itself will be out of their control.

Better quality research may also come from conceptually-driven choices of built environmental attributes and validated PA measures. Where accelerometer cut-points and the classification of older adults’ PA intensities are concerned, it is important that the thresholds for moderate intensity activity are appropriate (e.g., 1013 counts per min [112]). Applying suggestions such as these also allows for the possibility of pooling data across countries. For example, there has been evidence of curvilinearity related to perceived access to destinations and services and objectively assessed MVPA in a multi-country study of adults [31]. This finding was only possible because of the use of comparable environmental exposure and PA outcome measures across a large range of diverse geographical locations combined with a high variability in exposures across countries (another issue that future research may care to address). Thus, the multi-country pooling of data based on valid, comparable measures are needed to address issues surrounding limited variability in environmental exposures and non-linear associations between exposures and PA outcomes. Other statistical analysis decisions such as adjusting for key socio-demographic covariates (i.e., age, sex, and education), and not categorising continuous environmental measures would contribute to improving the quality of future research designs.

### Strengths and limitations of this review and meta-analysis

This systematic review and meta-analysis has several strengths. It addressed publication bias by including both peer-reviewed scientific articles and grey literature. It provided a quantitative synthesis of associations based on non-standardised environmental and PA measurement instruments and stratified findings by measurement types. It incorporated an extensive article quality assessment into the meta-analytical procedure and, therefore, adjusted the synthesis of evidence for study methodology quality. Limitations include: (1) not accounting for potentially correlated findings from the same article; (2) an inability to account for potential moderating effects of neighbourhood size and definition; (3) using a meta-analytic method that relied on statistical significance testing rather than effect size estimates and, thus, likely underestimating the evidence of environment-PA associations; and (4) including only articles published in English.

## Conclusions

Safe, walkable, and aesthetically pleasing neighbourhoods, with access to destinations and services, specifically, recreational facilities, parks/public open space, shops/commercial destinations and public transport facilitated older adults’ participation in PA, beyond domain-specificity. However, PA correlates were not consistent across different PA and environmental measurement types. Future research should consider these differences in findings and identify the mechanisms underlying them. Future studies should also strive to undertake higher quality research by implementing longitudinal research designs, adjusting for residential self-selection, conceptually-driven choosing of built environmental attributes, using validated PA measures (including, where necessary, appropriate accelerometer cut-points), pooling data from different countries based on valid standardised measures, adjusting for key socio-demographic covariates, and not inappropriately categorising continuous environmental measures.

## Additional files

Additional file 1: Table S1. Reviewed total physical activity articles (*N* = 100) – Information. (DOCX 286 kb)

Additional file 2: Table S3. Reviewed total physical activity articles (*N* = 100) – Quality assessment. (DOCX 106 kb)

Additional file 3: Table S2. Associations between built environmental attributes and older adults’ objectively measured walking. (DOCX 16 kb)

Additional file 4: Table S4. Meta-analytic results of significance of associations between built environmental correlates of older adults’ PA by outcome and type of adjustment for article characteristics. (DOCX 19 kb)

Additional file 5: Table S5. Overview of moderating effects examined in the association between environmental attributes and older adults’ total PA. (DOCX 214 kb)

## Abbreviations

MVPA: Moderate- to vigorous-intensity PA
PA: Physical activity
PhD: Doctor of Philosophy
PRISMA: Preferred Reporting Items for Systematic Reviews and Meta-Analyses
SD: Standard deviation
UK: United Kingdom of Great Britain and Northern Ireland
USA: United States of America
