# Supplementary material for: Built environmental correlates of older adults’ total physical activity and walking: a systematic review and meta-analysis
Source: Int J Behav Nutr Phys Act. 2017 Aug 7;14:103. doi: 10.1186/s12966-017-0558-z (PMC5547528; doi:10.1186/s12966-017-0558-z)
Supplement: Supplementary file 1 — Reviewed total physical activity articles (N = 100) – Information. (DOCX 286 kb) [file 12966_2017_558_MOESM1_ESM.docx]

**Table S1. Reviewed total physical activity articles (N=100) – Information**

| **#** | **Name & author, article reference in review**  **[Name of study, first author, publication year]** | **Participants**  **[Total sample size; urban, rural, or mixed sample; response rate or proof of representativeness of sample; community dwellers or not; geographical location]** | **Study design**  **[Cross-sectional, longitudinal, or experimental; sampling method for clusters and individuals; stratification used by environment attributes; neighbourhood definition]** | **Covariates**  **[Covariates included in the analyses]** | **Outcome measures**  **[PA outcome measure; instrument; validity]** | **Environmental exposure variables**  **[Environmental variables, their type (objective vs perceived) and their classification into environmental categories (to assist compilation of summary table)]** | **Moderators**  **[Moderators examined and breakdown of sample size by qualitative moderator (e.g., sex; educational attainment)]** | **Analytical approach**  **[Analytical approach; adjustment for clustering; appropriateness (distributional assumptions; moderation analyses) and presentation]** | **Findings**  **Main effects or moderating effects (conclusion in red)** | **Comments**  **[Notes important for the assessment or interpretation of the study (if any)]** |
| --- | --- | --- | --- | --- | --- | --- | --- | --- | --- | --- |
| 1 | Active Living Study  Nathan et al., 2014 [71] | N=323 (urban)  Mean age: 77 years  68% female  49% response rate (village)  46% response rate (person)  Retirement village dwellers  Perth, Australia | Cross-sectional  Cluster: purposive  Individuals: random and convenience  Stratification: walkability  Neighbourhood definition: retirement village, 10-15mins walk from village | Age, sex, education, physical functioning, neighbourhood walkability, sampling method, other significant environmental predictors, self-selection | *Self-report [Community Healthy Activities Model Program for Seniors (CHAMPS) questionnaire; validated]:*  Brisk walking (150+mins/wk; Yes/No) 🡪 Total walking *TotalWalking(150+ mins/wk; Yes/No)* | *Perceived [Neighborhood Environment Walkability Scale (NEWS)—Abbreviated; validated]:*  1. Access to activity centre 🡪 Social recreational facilities access/availability  2. Access to services – neighbourhood 🡪 Destinations/services (overall/unspecific) access/availability  3. Proximate destinations – village + neighbourhood 🡪 Destinations/services (overall/unspecific) access/availability  4. Infrastructure for walking – village + neighbourhood 🡪 Walk-friendly infrastructure  5. Aesthetics – village + neighbourhood 🡪 Greenery and aesthetically pleasing scenery  6. Safety from crime – village + neighbourhood 🡪 Crime/personal safety  7. Safety from traffic – village + neighbourhood 🡪 Traffic/pedestrian safety  8. Even gradient – village 🡪 No physical barriers to walking  9. Street connectivity – village 🡪 Street connectivity  10. Fewer physical barriers – neighbourhood 🡪 No physical barriers to walking  11. Orderliness – neighbourhood 🡪 Crime/personal safety  12. Age-appropriate infrastructure for walking – neighbourhood 🡪 Walk-friendly infrastructure  13. Traffic signal transition – neighbourhood 🡪 Traffic/pedestrian safety) | None | Generalized Estimating Equations with exchangeable correlation matrix to account for clustering | ***Main effects with TotalWalking(150+ mins/wk; Yes/No)***  1. Access to activity centre – village:  OR=not reported, p>.05  (Social recreational facilities access/availability **0**)  2. Access to services – neighbourhood:  OR=not reported, p>.05  (Destinations/services (overall/unspecific) access/availability **0**) 3. Proximate destinations – village + neighbourhood:  Village: OR=0.80 (0.66; 0.98) p<.05 (Destinations/services (overall/unspecific) access/availability **-*0.5**)  Neighbourhood: OR=not reported, p>.05 (Destinations/services (overall/unspecific) access/availability **0 *0.5**) 4. Infrastructure for walking – village + neighbourhood:  Village: OR=not reported, p>.05  Neighbourhood: OR=1.61 (0.76-3.45), p>.05 (Walk-friendly infrastructure **0*0.5; 0*0.5**)  5. Aesthetics – village + neighbourhood:  Village: OR=0.86 (0.49; 1.50) p>.05  Neighbourhood: OR=not reported p>.05  (Greenery and aesthetically pleasing scenery **0*0.5; 0*0.5**)  6. Safety from crime – village + neighbourhood:  Village: OR=0.43 (0.21; 0.88), p<.05 (Crime/personal safety **-*0.5**) Neighbourhood: OR=not reported, p>.05  (Crime/personal safety **0*0.5**)  7. Safety from traffic – village + neighbourhood:  ORs=not reported, p>.05 (Traffic/pedestrian safety **0*0.5; 0*0.5**)  8. Even gradient – village:  OR=not reported, p>.05 (No physical barriers to walking **0**)  9. Street connectivity – village:  OR=0.71 (0.51; 0.98), p<.05 (Street connectivity **-**)  10. Fewer physical barriers – neighbourhood:  OR=not reported, p>.05 (No physical barriers to walking **0**)  11. Orderliness – neighbourhood:  OR=not reported, p>.05  (Crime/personal safety **0**)  12. Age-appropriate infrastructure for walking – neighbourhood:  OR=1.51 (0.81; 2.82), p>.05 (Walk-friendly infrastructure **0**)  13. Traffic signal transition – neighbourhood:  OR=0.99 (0.66; 1.48), p>.05 (Traffic/pedestrian safety **0**) | Treat similar measures on village and neighbourhood environment as two buffers (assign fractional weights). Note that there are multiple measures per environmental construct that need to be summed.  Reporting fully adjusted models (with multiple environmental predictors) as these were adjusted for self-selection. |
| 2 | Active Living Study  Nathan et al., 2014 [153] | N=323 (urban)  Mean age: 77 years;  68% female  49% response rate (village)  46% response rate (person)  Retirement village dwellers  Perth, Australia | Cross-sectional  Cluster: purposive  Individuals: random and convenience  Stratification: walkability  Neighbourhood definition: retirement village, 400m street network buffer | Age, sex, physical functionality, sampling method, education | *Self-report [CHAMPS questionnaire; validated]:*  Brisk walking (any participation; Yes/No) 🡪 Total walking *TotalWalking(Yes/No)*  *Objective [ActiGraph accelerometer—validated; Freedson MVPA cutoff point—validated]:*  MVPA (150+ mins/wk; Yes/No) 🡪 Total MVPA *TotalMVPAFreedson(150+ mins/wk; Yes/No)* | *Objective [site manager’s questionnaire, GIS; unvalidated]:*  1. Age care facility 🡪 Health and aged care access/availability 2. Clubhouse 🡪 Social recreational facilities access/availability  3. Amenities 🡪 Land use mix—destination diversity  4. Recreational facilities 🡪 Recreational facilities access/availability  5. Neighbourhood walkability 🡪 Walkability | None | Generalized Estimating Equations with exchangeable correlation matrix to account for clustering | ***Main effects with TotalWalking(Yes/No)***  1. Age care facility:  OR=not reported, p>.05 (Health and aged care access/availability **0**)  2. Clubhouse:  OR=not reported, p>.05 (Social recreational facilities access/availability **0**)  3. Amenities:  OR=not reported, p>.05 (Land use mix—destination diversity **0**)  4. Recreational facilities:  OR=not reported, p>.05  (Recreational facilities access/availability **0**)  5. Neighbourhood walkability:  OR=not reported, p>.05 (Walkability **0**)  ***Main effects with TotalMVPAFreedson(150+ mins/wk; Yes/No)***  1. Age care facility:  OR=not reported, p>.05 (Health and aged care access/availability **0**)  2. Clubhouse:  OR=not reported, p>.05 (Social recreational facilities access/availability **0**)  3. Amenities:  OR=not reported, p>.05  (Land use mix–destination diversity **0**)  4. Recreational facilities:  OR=not reported, p>.05 (Recreational facilities access/availability **0**)  5. Neighbourhood walkability:  OR=not reported, p>.05 (Walkability **0**) | Report single attribute rather than fully-adjusted models because results not adjusted for self-selection. |
| 3 | Active Living Study  Nathan et al., 2014 [72] | N=323 (urban)  Mean age: 77 years;  68% female  49% response rate (village)  46% response rate (person)  Retirement village dwellers  Perth, Australia | Cross-sectional  Cluster: purposive  Individuals: random and convenience  Stratification: walkability  Neighbourhood definition: 10-15mins walk from home; 400m street network buffer | Age, sex, physical functioning, education, sampling method, other significant environmental predictors, self-selection | *Self-report [CHAMPS questionnaire; validated]:*  Brisk walking (150+mins/wk; Yes/No) 🡪 Total walking *TotalWalking(150+ mins/wk; Yes/No)*  *Objective [ActiGraph accelerometer + Freedson MVPA cutoff point; validated]:*  MVPA (150+ mins/wk; Yes/No) 🡪 Total MVPA *TotalMVPA(150+ mins/wk; Yes/No)* | *Objective [GIS, site manager’s questionnaire; unvalidated]:*  1. Distance to local shop 🡪 Shops/commercial access/availability  2. Distance to supermarket 🡪 Shops/commercial access/availability  3. Distance to health service 🡪 Health and aged care access/availability  4. Distance to entertainment facility 🡪 Social recreational facilities access/availability  5. Distance to public transport 🡪 Public transport access/availability  6. Distance to public recreation area 🡪 Recreational facilities access/availability  7. Traffic-volume exposure 🡪 Traffic/pedestrian safety  8. Slope 🡪 No physical barriers to walking 9. Age care facility – village 🡪 Health and aged care access/availability 10. Clubhouse – village 🡪 Social recreational facilities access/availability  11. Amenities – village 🡪 Land-use mix—destination diversity 12. Recreational facilities access/availability – village 🡪 Recreational facilities access/availability  13. Walkability – neighbourhood 🡪 Walkability  *Perceived [Neighborhood Environment Walkability Scale (NEWS)—Abbreviated; validated]:*  *Note:* Attributes were included but are not reported here because they were reported in Nathan et al., 2014 (Enviro&Behav) without adjustment for objective environment. | None | Generalized Estimating Equations with exchangeable correlation matrix to account for clustering | ***Main effects with TotalWalking(150+ mins/wk; Yes/No):***  1. Distance to local shop—OR (95% CIs):  OR=not reported, p>.05 in single attribute models  (Shops/commercial access/availability **0**)  2. Distance to supermarket:  OR=not reported, p>.05 in single-attributes models  (Shops/commercial access/availability **0**)  3. Distance to health service:  OR=negative, p<.05 in single-attributes models  (Health and aged care access/availability **+**)  4. Distance to entertainment facility:  OR=not reported, p>.05 (Social recreational facilities access/availability 0)  5. Distance to public transport:  OR=0.81 (0.70; 0.94), p=.007 in single-attributes models (Public transport **+**)  6. Distance to public recreation area:  OR=not reported, p>.05 (Recreational facilities access/availability **0**)  7. Traffic-volume exposure:  OR=not reported, p>.05 (Traffic/pedestrian safety **0**)  8. Slope:  OR=not reported, p>.05 (No physical barriers to walking **0**) 9. Age care facility:  OR=not reported, p>.05 (Health and aged care access/availability **0**) 10. Clubhouse:  OR=not reported, p>.05 (Social recreational facilities access/availability **0**) 11. Amenities--village:  OR=not reported, p>.05 (Land-use mix—destination diversity **0**) 12. Recreational facilities access/availability:  OR=not reported, p>.05 (Recreational facilities access/availability **0**) 13. Walkability:  OR=not reported, p>.05 (Walkability **0**)  ***Main effects with TotalMVPAFreedson(150+ mins/wk; Yes/No):***  1. Distance to local shop:  OR=0.67 (0.50; 0.90), p=.007 in single attribute models (Shops/commercial access/availability **+**)  2. Distance to supermarket:  OR=not reported, p>.05 in single-attributes models  (Shops/commercial access/availability **0**)  3. Distance to health service:  OR=negative, p<. 05 in single-attributes models (Health and aged care access/availability **+**)  4. Distance to entertainment facility:  OR=negative, p<.05 (Social recreational facilities access/availability **+**)  5. Distance to public transport:  OR=negative, p<.05 in single-attributes models  (Public transport **+**)  6. Distance to public recreation area:  OR=negative, p<.05 (Recreational facilities access/availability **+**)  7. Traffic-volume exposure:  OR=not reported, p>.05 (Traffic/pedestrian safety **0**)  8. Slope:  OR=not reported, p>.05 (No physical barriers to walking **0**) | Note that there are multiple measures per environmental construct that need to be summed.  Do not report perceived measures as they were reported in Nathan et al., 2014 (E&B) not adjusted for objective measures.  Reporting single-attribute models as self-selection shown not to be related to walking. |
| 4 | AGES (Aichi Gerontological Evaluation Study) Hanibuchi et al., 2011 [21] | N=9414 (mixed) 65+ years 52% female 48.7% response rate Community-dwellers Chita Peninsula, Japan | Cross-sectional Cluster: purposive Individuals: random Stratification: age and urbanisation Neighbourhood definition: 250m, 500m, and 1000m buffers | Age, sex, education, marital status, household income, having paid work, self-rated health, depression, instrumental activities of daily living (IADL) (physical function) | *Self-report [unnamed questionnaire; validated]:*  Walking (mins/d) 🡪 Total walking *TotalWalking(mins/d)* | *Objective [ArcGIS, census data, Geospatial Information Authority; unvalidated]:*  1. Population density 🡪 Residential density 2. Number of intersections 🡪 Street connectivity 3. Number of dead-ends 🡪 Street connectivity 4. Number of destinations 🡪 Destinations/services (overall/unspecific) access/availability 5. Parks or green spaces 🡪 Parks/public open space access/availability  6. Schools 🡪 Education facilities access/availability 7. Land slope 🡪 No physical barriers to walking | Sex:  Male (n=4519), Female (n=4895)  Urbanisation:  North (urban; n=3856),  South (rural; n=5558)  Length of neighbourhood residency: <50 years (n=4819), ≥50 years (n=4138) | Multivariate linear regression | ***Main and moderated effects with TotalWalking(mins/d):*** 1. Population density—OR (95% CIs): Inc. Males, Females, North, South,< and ≥50y neighbourhood residency + 250, 500 and 1000m buffers: (Residential density **0*0.055; 0*0.055; 0*0.055; 0*0.055; 0*0.055; 0*0.055; 0*0.055; 0*0.055; 0*0.055; 0*0.055; 0*0.055; 0*0.055; 0*0.055; 0*0.055; 0*0.055; 0*0.055; 0*0.055; 0*0.065**) 2. Number of intersections:  *Males + 250m:* OR=0.999 (0.993; 1.004),  p>.05 (Street connectivity **0*0.055**) *Males + 500m:* OR=0.999 (0.993; 1.002),  p>.05 (Street connectivity **0*0.055**) *Males + 1000m:* OR=1.000 (1.000; 1.001),  p<.05 (Street connectivity **+*0.055**) *Females + 250m:* OR=0.995 (0.990; 1.001),  p>.05 (Street connectivity **0*0.055**) *Females + 500m:* OR=0.998 (0.996; 1.000),  p>.05 (Street connectivity **0*0.055**) *Females + 1000m:* OR=1.000 (0.999; 1.000),  p>.05 (Street connectivity **0*0.055**) *North + 250m:* OR=0.999 (0.993; 1.005),  p>.05 (Street connectivity **0*0.055**) *North + 500m:* OR=0.999 (0.997; 1.001),  p>.05 (Street connectivity **0*0.055**) *North + 1000m:* OR=1.000 (0.999; 1.001),  p>.05 (Street connectivity **0*0.055**) *South + 250m:* OR=0.995 (0.989; 1.000),  p>.05 (Street connectivity **0*0.055**) *South + 500m:* OR=0.999 (0.997; 1.001),  p>.05 (Street connectivity **0*0.055**) *South + 1000m:* OR=1.000 (0.999; 1.000),  p>.05 (Street connectivity **0*0.055**) *<50 years + 250m:* OR=1.001 (0.996; 1.007),  p>.05 (Street connectivity **0*0.055**) *<50 years + 500m:* OR=1.000 (0.998; 1.002),  p>.05 (Street connectivity **0*0.055**) *<50 years + 1000m:* OR=1.000 (1.000; 1.001),  p<.05 (Street connectivity **+*0.055**) *≥50 years + 250m:* OR=0.992 (0.986; 0.997),  p<.05 (Street connectivity **-*0.055**) *≥50 years + 500m:* OR=0.998 (0.996; 1.000),  p>.05 (Street connectivity **0*0.055**) *≥50 years + 1000m:* OR=1.000 (0.999; 1.001),  p>.05 (Street connectivity **0*0.065**) 3. Number of dead-ends: Inc. Males, Females, North, South, < and ≥50y neighbourhood residency + 250, 500 and 1000m buffers: (Street connectivity **0*0.055; 0*0.055; 0*0.055; 0*0.055; 0*0.055; 0*0.055; 0*0.055; 0*0.055; 0*0.055; 0*0.055; 0*0.055; 0*0.055; 0*0.055; 0*0.055; 0*0.055; 0*0.055; 0*0.055; 0*0.065**) 4. Number of destinations: Inc. Males, Females, North, South, < and ≥50y neighbourhood residency + 250, 500 and 1000m buffers: (Destinations/services (overall/unspecific) access/availability **0*0.055; 0*0.055; 0*0.055; 0*0.055; 0*0.055; 0*0.055; 0*0.055; 0*0.055; 0*0.055; 0*0.055; 0*0.055; 0*0.055; 0*0.055; 0*0.055; 0*0.055; 0*0.055; 0*0.055; 0*0.065**) 5. Parks or green spaces: Inc. Males, Females, North, South, < and ≥50y neighbourhood residency + 250, 500 and 1000m buffers: (Parks/public open space **0*0.055; 0*0.055; 0*0.055; 0*0.055; 0*0.055; 0*0.055; 0*0.055; 0*0.055; 0*0.055; 0*0.055; 0*0.055; 0*0.055; 0*0.055; 0*0.055; 0*0.055; 0*0.055; 0*0.055; 0*0.065**) 6. Schools: Inc. Males, Females, North, South,< and ≥50y neighbourhood residency + 250, 500 and 1000m buffers: (Education facilities access/availability **0*0.055; 0*0.055; 0*0.055; 0*0.055; 0*0.055; 0*0.055; 0*0.055; 0*0.055; 0*0.055; 0*0.055; 0*0.055; 0*0.055; 0*0.055; 0*0.055; 0*0.055; 0*0.055; 0*0.055; 0*0.065**) 7. Land slope: *Males+250m:* OR=1.028 (1.000; 1.056),  p<.05 (No physical barriers to walking **-*0.055**) *Males + 500m:* OR=1.038 (1.002; 1.074),  p<.05  (No physical barriers to walking **-*0.055**) *Males + 1000m:* OR=1.021 (0.991; 1.053),  p>.05 (No physical barriers to walking **0*0.055**) *Females + 250m:* OR=1.045 (1.019; 1.072),  p<.05 (No physical barriers to walking **-*0.055**) *Females + 500m:* OR=1.058 (1.022; 1.094),  p<.05 (No physical barriers to walking **-*0.055**) *Females + 1000m:* OR=1.048 (1.019; 1.078),  p<.05  (No physical barriers to walking **-*0.055**) *North + 250m:* OR=0.997 (0.950; 1.047),  p>.05 (No physical barriers to walking **0*0.055**) *North + 500m:* OR=1.004 (0.947; 1.065),  p>.05 (No physical barriers to walking **0*0.055**) *North + 1000m:* OR=1.019 (0.948; 1.096),  p>.05  (No physical barriers to walking **0*0.055**) *South + 250m:* OR=1.043 (1.022; 1.065),  p<.05 (No physical barriers to walking **-*0.055**) *South + 500m:* OR=1.056 (1.028; 1.085),  p<.05 (No physical barriers to walking **-*0.055**) *South + 1000m:* OR=1.042 (1.019; 1.066),  p<.05 (No physical barriers to walking **-*0.055**) *<50 years + 250m:* OR=1.019 (0.986; 1.054),  p>.05 (No physical barriers to walking **0*0.055**) *<50 years + 500m:* OR=1.034 (0.992; 1.077),  p>.05 (No physical barriers to walking **0*0.055**) *<50 years + 1000m:* OR=1.026 (0.988; 1.065), p>.05 (No physical barriers to walking **0*0.065**) *≥50 years + 250m:* OR=1.044 (1.020; 1.069),  p<.05 (No physical barriers to walking **-*0.055**) *≥50 years + 500m:* OR=1.056 (1.023; 1.090),  p<.05 (No physical barriers to walking **-*0.055**) *≥50 years + 1000m:* OR=1.035 (1.008; 1.062),  p<.05 (No physical barriers to walking **-*0.055**) | Buffer and stratification effects. Table 8. |
| 5 | AIBL (Australian Imaging, Biomarkers & Lifestyle) study Cerin et al., 2016 [59] | N=146 (urban) Mean age=74.8-75.0 years 55.9-57.5% female 12.5% response rate Community-dwellers Melbourne, Australia | Cross-sectional Cluster: purposive Individual: convenience Stratification: none Neighbourhood definition: 0.5km and 1km buffers | Age, sex, education, median weekly household income, *APOE* ε4 status, time of assessment, *APOE* ε4 status by the time of assessment interaction | *Self-report [IPAQ—Long form; validated]:*  Total PA (MET mins/wk) 🡪 Total PA *TotalPA(MET mins/wk)* | *Objective [ArcGIS; unvalidated]:*  1. Walkability (inc., residential density, street connectivity, & land-use mix) 🡪 Walkability | Brain imaging outcomes: Right hippocampal volume (n=127);  Left hippocampal volume (n=127); Gray matter (n=127); Ventricle volume (n=127); Amyloid β burden (n=143) | Linear mixed regression models | ***PA-mediated indirect effects with TotalPA(MET mins/wk):*** 1. Walkability—b (95% CIs): *0.5km:* Right hippocampal volume:  b=0.008 (-0.001; 0.019) [390: 2.0∙10^-5^], p<.001 *1km:* Right hippocampal volume:  b=0.008 (-0.001; 0.019) [362: 2.0∙10^-5^], p<.001 *0.5km:* *Left hippocampal volume:* b=0.006 (-0.001; 0.017) [382: 1.7∙10^-5^], p<.001 *1km:* Left hippocampal volume:  b=0.007 (-0.001; 0.017) [353: 1.8∙10^-5^], p<.001 *0.5km:* Gray matter volume:  b=0.42 (-0.21; 1.21) [390: 1.1∙10^-3^], p<.001 *1km:* Gray matter volume:  b=0.47 (-0.10; 1.21) [361: 1.3∙10^-3^], p<.001 *0.5km:* Ventricle volume:  b=-0.16 (-0.67; 0.26) [382: -4.2∙10^-4^], p<.001 *1km:* Ventricle volume:  b=-0.13 (-0.61; 0.26) [352: -3.8∙10^-4^], p<.001 *0.5km:* Amyloid β burden:  b=-0.011 (-0.030; 0.001) [342: -3.3∙10^-5^], p<.001 *1km:* Amyloid β burden:  b=-0.009 (-0.029; 0.002) [309: -2.8∙10^-5^], p<.001 (Walkability **+*1**) | Table 2. |
| 6 | ALECS study Cerin et al., 2016 [73] | N=402 (urban) 65+ years 69% female 71% response rate Community-dwellers Hong Kong, China | Cross-sectional Cluster: purposive Individuals: random Stratification: walkability and SES Neighbourhood definition: 400m and 1km buffers | Age, sex, education, household car, marital status, housing type, area-level SES, number of diagnosed health problems, Short Physical Performance Battery Score, type of recruitment centre (Elderly Health Centre vs. community centre), accelerometer wear time | *Objective [ActiGraph accelerometer—Freedson cut-points (≥1952 counts/min); validated]:*  Total MVPA (min/d) 🡪 Total MVPA *TotalMVPAFreedson(mins/d)* | *Objective [GIS, census data, Lands Department of Hong Kong data; unvalidated]:*  1. Residential density 🡪 Residential density 2. Intersection density 🡪 Street connectivity 3. Retail density 🡪 Shops/commercial access/availability 4. Civic destination density 🡪 Government/finance services access/availability 5. Entertainment density 🡪 Social recreational facilities access/availability 6. Food outlet density 🡪 Food outlets access/availability 7. Recreation density 🡪 Recreational facilities access/availability 8. Public transit density 🡪 Public transport access/availability 9. Public park area 🡪 Parks/public open space access/availability  10. Nearest recreation destination 🡪 Recreational facilities access/availability  11. Nearest public park 🡪 Parks/public open space access/availability  12. Nearest trail 🡪 Parks/public open space access/availability  13. Nearest transit stop 🡪 Public transport access/availability | Intersection density*education; Entertainment density*number of diagnosed chronic health problems; Recreation density*education, Recreation density*household with car, recreation density*age; Public transit density*household with car; Nearest park*number of diagnosed chronic health problems | Generalised additive mixed models | ***Moderating effects with TotalMVPAFreedson(mins/d):*** Intersection density (400m)*education—e (95% CIs): Up to primary: e=1.033 (0.857; 1.246), p>.05 Secondary of higher: e=1.295 (1.031; 1.627), p<.05 Entertainment density (400m)*number of diagnosed chronic health problems: 1 SD below average (1.0 problems): e=0.994 (0.987; 1.002), p>.05 Average (3.1 problems): e=1.001 (0.996; 1.006), p>.05 1 SD above average (5.1 problems): e=1.007 (1.000; 1.015), p<.05 Recreation density (400m)*education: Up to primary: e=1.009 (1.000; 1.017), p<.05 Secondary of higher: e=1.001 (0.992; 1.010), p>.05 Recreation density (400m)*household with car: No: e=1.009 (1.001; 1.015), p<.05 Yes: e=0.999 (0.987; 1.010), p>.05 Recreation density (400m)*age: 1 SD below average (69.3 y): e=0.997 (0.992; 1.003), p>.05 Average (75.5 y): e=1.002 (1.001; 1.003), p<.001 1 SD above average (81.7 y): e=1.003 (1.001; 1.012), p<.05 Public transit density (1km)*household with car: No: e=0.989 (0.984; 0.994), p<.001 Yes: e=1.012 (1.002; 1.021), p<.05 Nearest park (100m)*number of diagnosed chronic health problems: 1 SD below average (1.0 problem): e=1.027 (1.003; 1.050), p<.05 Average (3.1 problems): e=1.010 (0.994; 1.027), p>.05 1 SD above average (5.1 problems): e=0.994 (0.974; 1.015), p>.05  ***Main and moderated effects with TotalMVPAFreedson(mins/d):*** 1. Residential density: *1km:* e=1.000 (0.996; 1.004), p=.979 *400m:* e=1.000 (0.999; 1.001), p=.815  (Residential density **0*0.5; 0*0.5**) 2. Intersection density: *1km:* e=1.206 (0.911; 1.594), p=.300 (Street connectivity **0*0.5**) *400m:* e=1.138 (0.980; 1.321), p=.090  (Street connectivity **0*0.25; 0*0.25**) 3. Retail density: *1km:* e=0.999 (0.997; 1.001), p=.294  *400m:* e=0.999 (0.996; 1.002), p=.444  (Shops/commercial access/availability **0*0.5; 0*0.5**) 4. Civic destination density: *1km:* e (95% CIs)=1.001 (0.997; 1.005), p=.553  *400m:* e (95% CIs)=0.999 (0.998; 1.001), p=.334  (Government/finance services access/availability **0*0.5; 0*0.5**) 5. Entertainment density: *1km:* e=0.991 (0.968; 1.001), p=.483  (Social recreational facilities access/availability **0*0.5**) *400m:* e=1.001 (0.995; 1.006), p=.800  (Social recreational facilities access/availability **0*0.17; 0*0.33**) 6. Food outlet density: *1km:* e=0.998 (0.992; 1.004), p=.564  *400m:* e=0.999 (0.993; 1.005), p=.732  (Food outlets access/availability **0*0.5; 0*0.5**) 7. Recreation density: *1km:* e=1.005 (0.998; 1.012), p=.154  (Recreational facilities access/availability **0*0.5**) *400m:* e=1.003 (1.000; 1.007), p=.040  (Recreational facilities access/availability **+*0.29; 0*0.21**) 8. Public transit density: *1km:* e=0.995 (0.991; 1.000), p=.052  (Public transport access/availability **0*0.25; 0*0.25**) *400m:* e=0.995 (0.990; 1.001), p=.058  (Public transport access/availability **0*0.5**) 9. Public park area: *1km:* e=1.000 (0.998; 1.002), p=.919  *400m:* e=0.995 (0.990; 1.001), p=.058  (Parks/public open space access/availability **0*0.5; 0*0.5**) 10. Nearest recreation destination: e=1.000 (0.999; 1.000), p=.888 (Recreational facilities access/availability **0**) 11. Nearest public park: e=1.000 (0.999; 1.001), p=.120  (Parks/public open space access/availability **0*0.33; 0*0.67**) 12. Nearest trail: e=1.000 (0.999; 1.000), p=.294  (Parks/public open space access/availability **0**) 13. Nearest transit stop: e=1.000 (0.999; 1.001), p=.512  (Public transport access/availability **0**) | Moderating effects (Table 4). Table 3. |
| 7 | Australian Time Use Survey 2006 Espinel et al., 2015 [128] | N=992 (mixed) 65+ years 56% female 82.5% response rate Community-dwellers *All states,* Australia | Cross-sectional Cluster: none Individuals: random Stratification: none Neighbourhood definition: not reported | Age, sex, education, SES, geographic remoteness, marital status, living alone, self-rated health, disability or long-term health condition | *Self-report [PA diary; unvalidated]:*  MVPA (30+ mins/d; Yes/No) 🡪 Total MVPA *TotalMVPA(30+ mins/d; Yes/No)* | *Objective [Accessibility/Remoteness Index of Australia; unvalidated]:*  1. Geographic remoteness 🡪 Urbanisation | None | Multivariate logistic regression | ***Main effects with TotalMVPA(30+ mins/d; Yes/No):*** 1. Geographic remoteness (Ref: Major cities)—OR (95% CIs): Inner regional or other: OR=1.03 (0.68; 1.55), p>.05 (Urbanisation **0**) | Table 3. |
| 8 | Behavior Change Consortium Initiative – Rhode Island Trial King et al., 2006 [140] | N=109 (not reported)  Mean age=75 years 65% women  36.9% response rate  Community-dwellers  Rhode Island, USA | Cross-sectional Cluster: none  Individual: convenience  Stratification: none  Neighbourhood definition: participant delimitation | Age, education | *Self-report [CHAMPS questionnaire; validated]:*  Total MPA or VPA (mins/wk) 🡪 Total MVPA *TotalMVPA(mins/wk)* | *Perceived [NEWS questionnaire; validated]:*  1. Residential density 🡪 Residential density  2. Land use mix: access 🡪 Destinations/services (overall/unspecific) access/availability  3. Street connectivity 🡪 Street connectivity  4. Seeing and speaking with others when walking in the neighbourhood 🡪 Crime/personal safety  5. Loose or unattended dogs 🡪 Crime/personal safety | None | Multivariate linear regression | ***Main effects with TotalMVPA(mins/wk):***  1. Residential density:  b=not reported, p>.05 (Residential density **0**)  2. Land use mix—access:  b=not reported, p>.05 (Destinations/services (overall/unspecific) access/availability **0**)  3. Street connectivity:  b=not reported, p>.05 (Street connectivity **0**)  4. Seeing and speaking with others when walking in the neighbourhood:  b=not reported, p>.05 (Crime/personal safety **0**)  5. Loose or unattended dogs:  b=not reported, p>.05 (Crime/personal safety **0**) | Note that there are multiple measures per environmental construct that need to be summed. |
| 9 | BEPAS Seniors Van Cauwenberg et al., 2016 [74] | N=391 (urban)  65+ years; 54% women  45% response rate  Community-dwellers  Ghent, Belgium | Cross-sectional  Cluster: purposive  Individual: random  Stratification: walkability and median household income  Neighbourhood definition: statistical sectors (administrative/census area) | Age, sex, marital status, education, physical functioning, number of motorised vehicles in household, residential self-selection | *Objective [ActiGraph—validated; Freedson and Copeland MVPA cutoff points—validated]:*  Freedson MVPA (mins/d) 🡪 Total MVPA *TotalMVPAFreedson(mins/d)*  Copeland MVPA (mins/d) 🡪 Total MVPA *TotalMVPACopeland(mins/d)* | *Objective [GIS; unvalidated]:*  1. Walkability 🡪 Walkability | Area-level income: Low (n=189), High (n=202) | Multilevel linear regression accounting for clustering and moderating effects | ***Moderating effect with TotalMVPAFreedson(mins/d):*** 1. Walkability*income (ref.=low)—OR (90% CIs): OR=-5.0 (-10.5; 0.6) p<.10  ***Moderating effect with Total MVPACopeland(mins/d):*** 1. Walkability*income (ref.=low): OR=-27.9 (-61.5; 5.7) p<.10  ***Moderated effect with TotalMVPAFreedson (mins/d):*** 1. Walkability—OR (95% CIs) (ref=high income): *Low income:* OR=5.6 (1.6; 9.6) p<.01  (Walkability **+*0.48; 0*0.52**)  ***Moderated effect with TotalMVPACopeland(mins/d):*** 1. Walkability: *Low income:* OR=27.2 (2.4; 52.0) p<.05  (Walkability **+*0.48; 0*0.52**) | Income effects. |
| 10 | BEPAS Seniors Van Holle et al., 2016 [170] | N=438 (urban)  65+ years; 54% women  45% response rate  Community-dwellers  Ghent, Belgium | Cross-sectional  Cluster: purposive  Individual: random  Stratification: walkability and median household income  Neighbourhood definition: statistical sectors (administrative/census area) | Age, sex, living situation, education, neighbourhood income | *Objective [ActiGraph—validated; Freedson MVPA cutoff point; validated]:*  Freedson MVPA (mins/wk) 🡪 Total MVPA *TotalMVPAFreedson(mins/wk)* | *Perceived [NEWS; validated]:*  1. Land use mix—diversity 🡪 Land-use mix—destination diversity  2. Access to recreational facilities 🡪 Recreational facilities access/availability  3. Connectivity 🡪 Street connectivity  4. Physical barriers to walking 🡪 No physical barriers to walking  5. Walking infrastructure 🡪 Walk-friendly infrastructure  6. Aesthetics 🡪 Greenery and aesthetically pleasing scenery  7. Safety from crime 🡪 Crime/personal safety  8. Safety traffic speeding 🡪 Traffic/pedestrian safety  *Objective [GIS; unvalidated]:*  *Note:* Walkability not extracted as reported in Van Holle et al., 2014. | None | Multilevel linear regression accounting for clustering - squared root of outcome | ***Main effects with TotalMVPAFreedson(mins/wk):*** 1. Land-use mix—diversity: b=1.38 (SE=0.45), p<.05 (Land-use mix—destination diversity **+**) 2. Access to recreational facilities: b=0.33 (SE=0.20), p>.05 (Recreational facilities access/availability **0**) 3. Connectivity: b=0.29 (SE=0.29), p>.05 (Street connectivity **0**) 4. Physical barriers to walking: b=0.18 (SE=0.5), p>.05 (No physical barriers to walking **0**) 5. Walking infrastructure: b=0.97 (SE=0.45), p<.05 (Walk-friendly infrastructure **+**) 6. Aesthetics: b=0.33 (SE=0.37), p>.05 (Greenery and aesthetically pleasing scenery **0**) 7. Safety from crime: b=0.37 (SE=0.34), p>.05 (Crime/personal safety **0**) 8. Safety traffic speeding: b=0.23 (SE=0.23), p>.05 (Traffic/pedestrian safety **0**) | Supplementary Table 3. Walkability not extracted as reported in Van Holle et al., 2014. |
| 11 | British Regional Heart Study & British Women’s Heart Health Study Jefferis et al., 2014 [50] | N=2426 (urban) 70+ years 35% female 29-51% response rate Community-dwellers *24 British towns,* United Kingdom | Cross-sectional Cluster: purposive Individuals: random Stratification: population size, representative of the region in terms of: cardiovascular mortality rates, water quality, socio-economic activity, high mobility towns (Lawlor, Bedford, Taylor, & Ebrahim, 2003; Walker, Whincup, & Shaper, 2004)  Neighbourhood definition: participant delineation | Age, season, region, average monitor wear time | *Objective [ActiGraph accelerometer; validated; Copeland & Esliger MVPA cutoff point (>1040 counts/min; validated]:*  MVPA (150+ mins/wk of 10+ min/bout; Yes/No) 🡪 Total MVPA *TotalMVPACopeland(150+ mins/wk; Yes/No)* | *Perceived [unnamed questionnaire; unvalidated]:*  1. Social and leisure activities 🡪 Social recreational facilities access/availability  2. Facilities for people your age 🡪 Destinations/services (overall/unspecific) access/availability 3. Local transport 🡪 Public transport access/availability 4. Somewhere nice to go for a walk 🡪 Destinations/services (overall/unspecific) access/availability 5. Feel safe walking alone in the daytime 🡪 Crime/personal safety 6. Feel safe walking alone after dark 🡪 Crime/personal safety | Sex:  Males (n=1577), Female (n=849) | Multivariate logistic regression | ***Main and moderated effects with TotalMVPACopeland(150+ mins/wk; Yes/No):*** Males: 1. Social and leisure activities—OR (95% CIs): *Males:* OR=1.48 (1.11; 1.97), p<.05 (Social recreational facilities access/availability **+*0.65**) *Females:* OR=1.54 (0.94; 2.52), p>.05 (Social recreational facilities access/availability **0*0.35**) 2. Facilities for people your age: *Males:* OR=1.25 (0.94; 1.66), p>.05 (Destinations/services (overall/unspecific) access/availability **0*0.65**) *Females:* OR=1.70 (1.01; 2.87), p>.05 (Destinations/services (overall/unspecific) access/availability **+*0.35**) 3. Local transport: *Males:* OR=1.04 (0.76; 1.42), p>.05 *Females:* OR=0.83 (0.49; 1.41), p>.05 (Public transport access/availability **0*0.65; 0*0.35**) 4. Somewhere nice to go for a walk: *Males:* OR=1.90 (1.20; 3.02), p<.05 (Destinations/services (overall/unspecific) access/availability **+*0.65**) *Females:* OR=1.91 (0.92; 3.98), p>.05 (Destinations/services (overall/unspecific) access/availability **0*0.35**) 5. Feel safe walking alone in the daytime: *Males:* OR=2.15 (0.76; 6.10), p>.05 *Females:* OR=2.50 (0.33; 19.07), p>.05 (Crime/personal safety **0*0.65; 0*0.35**) 6. Feel safe walking alone after dark: *Males:* OR=2.59 (1.77; 3.81), p<.05 *Females:* OR=2.81 (1.73; 4.54), p<.05 (Crime/personal safety **+*0.65; +*0.35**) | Sex effects.  Table 3 – Model 1. |
| 12 | Canada’s General Social Survey Time Use Spinney & Millward 2014 [54] | 1992: N=1992 (mixed) 1998: N=1889 (mixed) 2005: N=3589 (mixed) 2010: N=3639 (mixed) 65+ years 1992: 66% female 1998: 63% female 2005: 60% female 2010: 59% female Response rates not reported Community-dwellers *10 provinces,* Canada | Cross-sectional at four time points Cluster: purposive Individuals: random Stratification: urbanisation Neighbourhood definition: census level | Age, sex, education, household income, activity limitation, living situation, season (logistic regression only) | *Self-report [General Social Survey Time Use questionnaire; unvalidated]:*  MVPA (21+ mins/d; Yes/No) 🡪 Total MVPA *TotalMVPA(21+ mins/d; Yes/No) TotalMVPA(median mins/d)*  MVPA (median mins/d) 🡪 Total MVPA *TotalMVPA(median mins/d)* | *Objective [census data; unvalidated]:*  1. Urbanisation 🡪 Urbanisation | None | Mann Whitney *U* test, multivariate logistic regression | ***Main effects with TotalMVPA(21+ mins/d; Yes/No):*** 1. Urbanisation—OR (95% CIs): 1992: OR=1.452 (1.050; 2.009), p<.01 (Urbanisation **-**) 1998: OR=1.529 (1.088; 2.149), p<.05 (Urbanisation **-**) 2005: OR=1.091 (0.880; 1.352), p>.05 (Urbanisation **0**) 2010: OR=1.326 (1.091; 1.612), p<.01 (Urbanisation **-**)  ***Main effects with TotalMVPA(median mins/d):*** 1. Urbanisation (urban vs. rural): 1992: 75 mins vs. 120 mins, p=.001 (Urbanisation **-**) 1998: 90 mins vs. 120 mins, p=.175 (Urbanisation **0**) 2005: 105 mins vs. 120 mins, p=.042 (Urbanisation **-**) 2010: 100 mins vs. 120 mins, p=.001 (Urbanisation **-**) | Table 2 and 3. |
| 13 | CCHS (Canadian Community Health Survey) 2008/2009 Winters et al., 2015 [172] | N=1309 (urban) Mean age: 75 years 55% female 74% response rate Community-dwellers British Columbia, Canada | Cross-sectional Cluster: purposive Individuals: random Stratification: urbanisation (urban only)  Neighbourhood definition: census metropolitan area | Age, sex, education, country of birth, mobility, fear of falls | *Self-report [CCHS questionnaire; unvalidated]:*  Outdoor walking (150+ mins/wk; Yes/No) 🡪 Total walking *TotalWalking(150+ mins/wk; Yes/No* | *Objective [Street Smart Walk Score; validated]:*  1. Walkability 🡪 Walkability | None | Multivariate logistic regression | ***Main effects with TotalWalking(150+ mins/wk; Yes/No):*** 1. Walkability—OR (95% CIs): OR=1.17 (1.07; 1.27), p<.05 (Walkability **+**) | Table 2—Adjusted. |
| 14 | CHIS (California Health Interview Survey) 2003 data Li et al., 2015 [144] | N=965 (sub-sample; urban) 65+ years 56% female 60% response rate Community-dwellers California, USA | Cross-sectional Cluster: purposive Individuals: random Stratification: ethnicity (Asian) Neighbourhood definition: not defined | Age, sex, education, immigration status, marital status, poverty level, employment status, health conditions (asthma and heart disease), instrumental activities of daily living, BMI | *Self-report [CHIS questionnaire; unvalidated]:*  Total walking (mins/wk) 🡪 Total walking *TotalWalking(mins/wk)* Total walking (non-walkers) 🡪 Total walking *TotalWalking(non-walkers; Yes/No)* | *Perceived [CHIS questionnaire; unvalidated]:*  1. Nearby park/playground 🡪 Parks/public open space access/availability  2. Safe neighbourhood 🡪 Crime/personal safety | Ethnicity: Chinese (n=355), Filipino (n=173), Japanese (n=164), Korean (n=140), Vietnamese (n=133) | Zero-inflated negative binomial regression | ***Main and moderated effects with TotalWalking(mins/wk):*** 1. Nearby park/playground (Ref: no)—IRR (95% CIs): *Chinese:* IRR=1.25 (1.03; 1.51), p<.05 (Parks/public open space access/availability **+*0.367**) *Filipino:* IRR=1.17 (0.74; 1.83), p>.05 (Parks/public open space access/availability **0*0.179**) *Japanese:* IRR=0.54 (0.31; 0.96), p<.05 (Parks/public open space access/availability **-*0.170**) *Korean:* IRR=1.99 (1.05; 2.77), p<.05 (Parks/public open space access/availability **+*0.145**) *Vietnamese:* IRR=1.39 (0.85; 2.28), p>.05 (Parks/public open space access/availability 0***0.139**) 2. Safe neighbourhood (Ref: no): *Chinese:* IRR=1.13 (0.94; 1.36), p>.05 (Crime/personal safety **0*0.367**) *Filipino:* IRR=1.99 (1.27; 3.12), p<.01 (Crime/personal safety **+*0.179**) *Japanese:* IRR=0.91 (0.51; 1.63), p>.05 (Crime/personal safety **0*0.170**) *Korean:* IRR=0.77 (0.49; 1.23), p>.05 (Crime/personal safety **0*0.145**) *Vietnamese:* IRR=0.78 (0.47; 1.30), p>.05 (Crime/personal safety **0*0.139**)  ***Main effects with TotalWalking(non-walkers; Yes/No):*** 1. Nearby park/playground (Ref: no): *Chinese:* OR=1.15 (95% CI=0.46; 2.88), p>.05 *Filipino:* OR=1.56 (95% CI=0.41; 5.90), p>.05 *Japanese:* IRR=0.96 (95% CI=0.30; 3.09), p>.05 *Korean:* OR=1.65 (95% CI=0.30; 9.16), p>.05 *Vietnamese:* OR=3.00 (95% CI=0.81; 11.19), p>.05 (Parks/public open space access/availability **0*0.367; 0*0.179; 0*0.170; 0*0.145; 0*0.139**) 2. Safe neighbourhood (Ref: no): *Chinese:* OR=0.95 (95% CI=0.46; 1.94), p>.05 *Filipino:* OR=0.60 (95% CI=0.17; 2.11), p>.05 *Japanese:* IRR=0.37 (95% CI=0.12; 1.13), p>.05 *Korean:* OR=3.59 (95% CI=0.55; 23.66), p>.05 *Vietnamese:* IRR=2.52 (95% CI=0.55; 11.60), p>.05 (Crime/personal safety **0*0.367; 0*0.179; 0*0.170; 0*0.145; 0*0.139**) | Ethnicity effects. Table 3. |
| 15 | CNDS (Chicago Neighborhood and Disability Study) data Mendes de Leon et al., 2009 [149] | N=4317 (urban) Mean age: 75 years 61% female 78.9% response rate Community-dwellers Chicago, USA | Cross-sectional Cluster: convenience Individuals: all invited to participate Stratification: none Neighbourhood definition: census block | Age, sex, education, income, marital status, years of residence in the neighbourhood, medical condition, season | *Self-report [Health Interview Survey 1985; unvalidated]:*  Total walking (mins/2 wks) 🡪 Total walking *TotalWalking(mins/2 wks)* | *Objective [unnamed questionnaire; unvalidated]:*  1. Neighbourhood-level disorder 🡪 Crime/personal safety | None | Multilevel linear regression accounting for clustering, square root transformation of outcome variable | ***Main effects with TotalWalking(mins/2 wks):*** 1. Neighbourhood-level disorder: b=-2.78, p<.01 (Crime/personal safety **+**) | Table 2—Model 2. |
| 16 | DIY (Do-It-Yourself) Streets Thompson et al., 2012 [66] | Pre-intervention: N=96 (urban) Post-intervention: N=61 (urban) Mean age: 74-77 years 51-65% female Response rate not reported England, Scotland, and Wales, United Kingdom | Intervention study Cluster: purposive  Individuals: convenience Stratification: street wide enough + not a major route; comparison streets matched by housing type, street layout and SES Neighbourhood definition: not defined | Age, instrumental activities of daily living | *Self-report [unnamed questionnaire; unvalidated]:*  Time spent outdoors 🡪 Total PA *TotalPA(duration)* | *Perceived [unnamed questionnaire; unvalidated]:*  1. Pleasant local open space 🡪 Parks/public open space 2. Barriers/nuisance in local open space and neighbourhood 🡪 Crime/personal safety 3. Bad footways/paths 🡪 Pavement/footpath quality 4. Easy to get out and about 🡪 No physical barriers to walking 5. Good paths and cycleways 🡪 Cycle/walk-friendly infrastructure | Intervention | Multivariate linear regression | ***Main and intervention effects with TotalPA(duration):*** 1. Pleasant local open space: *Pre-intervention:* b=not reported, p>.05 *Post-intervention:* b=not reported, p>.05 (Parks/public open space access/availability **0*1**) 2. Barriers/nuisance in local open space and neighbourhood: *Pre-intervention:* b=not reported, p>.05 *Post-intervention:* b=-0.309, p<.05 (Crime/personal safety **+*1**) 3. Bad footways/paths: *Pre-intervention:* b=not reported, p>.05 *Post-intervention:* b=not reported, p>.05 (Pavement/footpath quality **0*1**) 4. Easy to get out and about: *Pre-intervention:* b=.228 (standardised b=.236), p<.05 *Post-intervention:* b=not reported, p>.05 (No physical barriers to walking **-*1**) 5. Good paths and cycleways: *Pre-intervention:* b=not reported, p>.05 *Post-intervention:* b=0.293, p<.05 (Cycle/Walk-friendly infrastructure **+*1**) | Intervention effects. Table 4. |
| 17 | Easy Steps to Health Merom et al., 2015 [100] | N=301 (urban) 65+ years 73% female Response rate not reported  Community-dwellers Sydney, Australia | Cross-sectional (not an environmental intervention) Cluster: convenience Individuals: convenience Stratification: none Neighbourhood definition: 15-20mins walk from home | Age, sex, education, intervention, recurrent fallers, self-rated health, self-efficacy, fear of falling, unable to walk 30 min, *interaction term: pedestrian-orientated neighbourhood (walkability)*intervention* | *Self-report [Incidental and Planned Exercise Questionnaire; validated]:*  Planned walking (2.5+ hr/wk; Yes/No) 🡪 Total walking *TotalWalking(2.5+ hr/wk; Yes/No)* | *Perceived [NEWS-AUS questionnaire; validated]:*  1. Many places to go within easy walking distance 🡪 Destinations/services (overall/unspecific) access/availability 2. Easy to walk to a public transport stop 🡪 Public transport access/availability 3. Footpaths on most of the streets 🡪 Walk-friendly infrastructure 4. Crosswalks and pedestrian signals 🡪 Traffic/pedestrian safety 5. Neighbourhood streets non-hilly 🡪 No physical barriers to walking 6. Walkers in neighbourhood easily seen 🡪 Crime/personal safety 7. Lots of greenery in local area 🡪 Greenery and aesthetically pleasing scenery 8. Many interesting things to look at 🡪 Greenery and aesthetically pleasing scenery 9. Not much traffic along nearby streets 🡪 Traffic/pedestrian safety 10. Local parks and walking trails 🡪 Parks/public open space access/availability 11. Crime rate 🡪 Crime/personal safety | Fear of falling (n=310),  Health status (n=315), Intervention group (n=145), Pedestrian-orientated neighbourhood (n=314) | Generalised linear models with binominal distribution | *No significant moderating effects.*  ***Main effects with TotalWalking(2.5+ h/wk; Yes/No):*** 1. Many places to go within easy walking distance—OR (95% CIs): OR=0.65 (0.30; 1.40), p>.05 (Destinations/services (overall/unspecific) access/availability **0**) 2. Easy to walk to a public transport spot: OR=1.90 (0.63; 5.72), p>.05 (Public transport access/availability **0**) 3. Footpaths on most of the streets: OR=1.65 (0.74; 3.65), p>.05 (Walk-friendly infrastructure **0**) 4. Crosswalks and pedestrian signals: OR=0.93 (0.43; 2.04), p>.05 (Traffic/pedestrian safety **0**) 5. Neighbourhood streets non-hilly: OR=0.94 (0.38; 2.33), p>.05 (No physical barriers to walking **0**) 6. Walkers in neighbourhood easily seen: OR=1.03 (0.45; 2.31), p>.05 (Crime/personal safety **0**) 7. Lots of greenery in local area: OR=3.33 (1.11; 9.98), p<.05 (Greenery and aesthetically pleasing scenery **+**) 8. Many interesting things to look at: OR=0.55 (0.21; 1.45), p>.05 (Greenery and aesthetically pleasing scenery **0**) 9. Not much traffic along nearby streets: OR=1.98 (1.00; 3.91), p<.05 (Traffic/pedestrian safety **+**) 10. Local parks and walking trails: OR=0.55 (0.22; 1.39), p>.05 (Parks/public open space access/availability **0**) 11. Crime rate: OR=0.30 (0.09; 1.05), p>.05 (Crime/personal safety **0**) | Table 2–adjusted. |
| 18 | EPOSA (European Project on OSteoArthritis –Dutch trial) Timmermans et al., 2016 [166] | N=247 (mixed) Mean age: 75 years 50% female 84.1% response rate Dwelling not reported Amsterdam, Netherlands | Cross-sectional Cluster: purposive Individuals: random (original study); convenience (data drawn from larger study) Stratification: region and urbanisation Neighbourhood definition: postal code | Age, sex, education, partner status, urbanisation, body mass index, no. of chronic diseases other than lower limb osteoarthritis (LLOA), anxiety, depression, functional limitations, wear time of accelerometer, LLOA (Note. LLOA as a covariate was not included in models for general practice, pharmacy, and supermarket due to an interaction effect between those destinations and PA) | *Objective [ActiGraph accelerometer—validated; Matthews MVPA cutoff point≥2020 cpm and LPA cutoff point>100 cpm—validated]:*  Total PA mins/d 🡪 Total PA *TotalPA(mins/d)* MVPA mins/d 🡪 Total MVPA *TotalMVPAMatthews(mins/d)* | *Objective [ArcGIS, Statistics Netherlands, The Netherlands’ Cadastre, Land Registry, Mapping Agency; unvalidated]:*  1. Street connectivity 🡪 Street connectivity 2. Distance to general practice 🡪 Health and aged care access/availability 3. Distance to general practice centre 🡪 Health and aged care access/availability 4. Distance to pharmacy 🡪 Health and aged care access/availability 5. Distance to hospital, with outside clinic 🡪 Access to/availability of Health and aged care access/availability 6. Distance to hospital, without outside clinic 🡪 Health and aged care access/availability 7. Distance to physiotherapist 🡪 Health and aged care access/availability 8. Distance to supermarket 🡪 Shops/commercial access/availability 9. Distance to grocery store 🡪 Shops/commercial access/availability 10. Distance to department store 🡪 Shops/commercial access/availability 11. Distance to pub 🡪 Social recreational facilities access/availability  12. Distance to cafeteria 🡪 Food outlets access/availability 13. Distance to restaurant 🡪 Food outlets access/availability 14. Distance to train station 🡪 Public transport access/availability 15. Distance to important transfer station 🡪 Public transport access/availability | None | Multivariate linear regression | ***Main effects with TotalPA(mins/d):*** 1. Street connectivity:  b=0.08 (SE=0.05) p>.05 (Street connectivity **0**) 2. Distance to general practice: b=1.75 (SE=4.92) p>.05 (Health and aged care access/availability **0**) 3. Distance to general practice centre: b=0.49 (SE=1.15) p>.05 (Health and aged care access/availability **0**) 4. Distance to pharmacy: b=0.22 (SE=4.27) p>.05 (Health and aged care access/availability **0**) 5. Distance to hospital with outside clinic: b=-0.66 (SE=1.46) p>.05 (Health and aged care access/availability **0**) 6. Distance to hospital without outside clinic: b=0.47 (SE=1.13) p>.05 (Health and aged care access/availability **0**) 7. Distance to physiotherapist: b=0.52 (SE=6.27) p>.05 (Health and aged care access/availability **0**) 8. Distance to supermarket: b=1.60 (SE=4.66) p>.05 (Shops/commercial access/availability **0**) 9. Distance to grocery store: b=-0.95 (SE=3.26) p>.05 (Shops/commercial access/availability **0**) 10. Distance to department store: b=1.54 (SE=1.49) p>.05 (Shops/commercial access/availability **0**) 11. Distance to pub: b=-2.00 (SE=3.11) p>.05 (Social recreational facilities access/availability **0**) 12. Distance to cafeteria: b=0.28 (SE=5.46) p>.05 (Food outlets access/availability **0**) 13. Distance to restaurant: b=-8.71 (SE=7.57) p>.05 (Food outlets access/availability **0**) 14. Distance to public transport: b=-0.64 (SE=0.89) p>.05 (Public transport access/availability **0**) 15. Distance to important transfer station: b=0.62 (SE=0.59) p>.05 (Public transport access/availability **0**)  ***Main effects with TotalMVPAMatthews(mins/d):*** 1. Street connectivity:  b=0.01 (SE=0.02) p>.05 (Street connectivity **0**) 2. Distance to general practice: b=1.75 (SE=4.92) p>.05 (Health and aged care access/availability **0**) 3. Distance to general practice centre: b=-0.10 (SE=0.35) p>.05 (Health and aged care access/availability **0**) 4. Distance to pharmacy: b=0.51 (SE=1.30) p>.05 (Health and aged care access/availability **0**) 5. Distance to hospital with outside clinic: b=0.28 (SE=0.44) p>.05 (Health and aged care access/availability **0**) 6. Distance to hospital without outside clinic: b=-0.11 (SE=0.34) p>.05 (Health and aged care access/availability **0**) 7. Distance to physiotherapist: b=-3.32 (SE=1.90) p>.05 (Health and aged care access/availability **0**) 8. Distance to supermarket: b=0.87 (SE=1.42) p>.05 (Shops/commercial access/availability **0**) 9. Distance to grocery store: b=1.18 (SE=0.98) p>.05 (Shops/commercial access/availability **0**) 10. Distance to department store: b=-0.19 (SE=0.45) p>.05 (Shops/commercial access/availability **0**) 11. Distance to pub: b=0.48 (SE=0.95) p>.05 (Social recreational facilities access/availability **0**) 12. Distance to cafeteria: b=-0.87 (SE=1.66) p>.05 (Food outlets access/availability **0**) 13. Distance to restaurant: b=-4.96 (SE=2.29) p>.05 (Food outlets access/availability **0**) 14. Distance to public transport: b=0.30 (SE=0.27) p>.05 (Public transport access/availability **0**) 15. Distance to important transfer station: b=0.62 (SE=0.59) p>.05 (Public transport access/availability **0**) | Table 4—Model 2. |
| 19 | Great Britain older adults 1 (name assigned) Sugiyama & Ward Thompson 2007 [162] | N=264 (mixed) 65+ years 57% female 10% response rate England, Scotland, and Wales, United Kingdom | Cross-sectional Cluster: purposive Individuals: random Stratification: urbanisation, SES, functional status, living arrangements and cultural background Neighbourhood definition: local authority | None | *Self-report [unnamed questionnaire; unvalidated]:*  Summer and Winter outdoor walking (2.5+ hrs/wk; Yes/No) 🡪 Total Walking *TotalWalking(2.5+ hrs/wk; Yes/No)* | *Perceived [unnamed questionnaire; unvalidated]:*  1. Living in an urban/rural area 🡪 Urbanisation | Urbanisation:  Urban (n=205), Rural (n=59) | Chi-square test | ***Main effect with TotalWalking(2.5+ hrs/wk; Yes/No):*** 1. Living in an urban/rural area: Χ^2^=not reported, p>.05 (Urbanisation **0**) | In-text, Section 2.3: sentence beginning “Ethnicity and living in urban or rural…” |
| 20 | Great Britain older adults 1 (name assigned) Sugiyama et al., 2009 [163] | N=271 (mixed) 65+ years 60% female 14% response rate England, Scotland, and Wales, United Kingdom | Cross-sectional Cluster: purposive Individuals: random Stratification: urbanisation, SES, functional status, living arrangements and cultural background Neighbourhood definition: participant delineation | Age, education, physical function | *Self-report [unnamed questionnaire; unvalidated]:*  Summer and Winter outdoor walking (2.5+ hrs/wk; Yes/No) 🡪 Total Walking *TotalWalking(2.5+ hrs/wk; Yes/No)* | *Perceived [unnamed questionnaire; unvalidated]:*  1. Pleasantness of neighbourhood open space 🡪 Greenery and aesthetically pleasing scenery 2. Nuisance in neighbourhood open space 🡪 Crime/personal safety 3. Quality of paths to neighbourhood open space 🡪 Pavement/footpath quality 4. Distance to neighbourhood open space 🡪 Parks/public open space access/availability | None | Multivariate logistic regression | ***Main effects with TotalWalking(2.5+ hrs/wk; Yes/No):*** 1. Pleasantness of neighbourhood open space (Ref: Low): High: OR=1.68 (95% CI=0.87; 3.23), p>.05 (Greenery and aesthetically pleasing scenery **0**) 2. Nuisance in neighbourhood open space (Ref: High): Low: OR=1.18 (95% CI=0.62; 2.24), p>.05 (Crime/personal safety **0**) 3. Quality of paths to neighbourhood open space (Ref: Low): High: OR=1.96 (95% CI=1.03; 3.74), p<.05 (Pavement/footpath quality **+**) 4. Distance to neighbourhood open space (Ref:>10 mins walk away): Within 10 mins walk: OR=1.61 (95% CI=0.85; 3.07), p>.05 (Parks/public open space access/availability **0**) | Table 3. |
| 21 | HAN (Healthy Aging Research Network) Walking study Satariano et al., 2010 [159] | N=884 (mixed) 65+ years 77% female Response rate not reported Community-dwellers California, Pennsylvania, Illinois, and North Carolina, USA | Cross-sectional Cluster: purposive Individuals: random Stratification: residential density Neighbourhood definition: 15-20mins walk from home (perceived); 400m buffer (objective) | Age, sex, education, study site, race, income, lower-body function, and drive/access to a driver | *Self-report [unnamed questionnaire; unvalidated]:*  Total walking (<150 mins/wk; Yes/No) 🡪 Total walking *TotalWalking(<150 mins/wk; Yes/No)* | *Objective [GIS—census data, RAND Center for Population Health and Health Disparities; unvalidated]:*  1. Street connectivity 🡪 Street connectivity 2. Housing unit density 🡪 Residential density 3. Number of retail businesses within the buffer 🡪 Shops/commercial access/availability 4. Primary type of buildings in neighbourhood 🡪 Land-use mix—destination diversity  *Perceived [NEWS questionnaire; validated]:* 5. Residential density 🡪 Residential density 6. Land-use mix: access 🡪 Destinations/services (overall/unspecific) access/availability 7. Land-use mix: diversity 🡪 Land-use mix—destination diversity 8. Street connectivity 🡪 Street connectivity 9. Walking/cycling facilities 🡪 Cycle/walk-friendly infrastructure 10. Aesthetics 🡪 Greenery and aesthetically pleasing scenery 11. Pedestrian/automobile traffic safety 🡪 Traffic/pedestrian safety 12. Crime safety 🡪 Crime/personal safety | Physical function**all environmental exposure variables* | Generalised logistic estimating equation | *No significant moderating effect for* physical function*crime safety:  OR=2.31 (95% CI=1.22; 4.38), p=.12 Significant moderating effect for physical function*housing unit density:  OR=2.35 (95% CI=1.06; 5.21), p=.06  ***Main and moderated effects with TotalWalking(<150 mins/wk; Yes/No):***  1. Street connectivity + 8. Street connectivity: *Objective (#1):* OR=not reported, p>.05 *Perceived (#8):* OR=not reported, p>.05 (Street connectivity **0*1; 0*1**) 2. Housing unit density + 5. Residential density: *Objective (#2):* OR=not reported, p>.05 (Residential density **0*0.25; 0*0.75**) *Perceived (#5):* OR=not reported, p>.05 (Residential density **0*1**) 3. Number of retail businesses: *Objective:* OR=not reported, p>.05 (Shops/commercial access/availability **0**) 4. Primary type of buildings in neighbourhood (Ref: Commercial or a mix of residential and commercial) + 7. Land-use mix—destination diversity: *Objective (#4):* Residential: OR=1.54 (95% CI=1.05; 2.25), p=.03 (Land-use mix—destination diversity **+**) *Perceived (#7):* OR=not reported, p>.05 (Land-use mix—destination diversity **0**) 6. Land-use mix: access: *Perceived:* OR=not reported, p>.05 (Destinations/services (overall/unspecific) access/availability **0**) 9. Walking/cycling facilities: *Perceived:* OR=not reported, p>.05 (Cycle/Walk-friendly infrastructure **0**) 10. Aesthetics: *Perceived:* OR=not reported, p>.05 (Greenery and aesthetically pleasing scenery **0**) 11. Pedestrian/automobile traffic safety: *Perceived:* OR=not reported, p>.05 (Traffic/pedestrian safety **0**) 12. Crime safety (Ref: Feel safe from crime in neighbourhood): *Perceived:* Feel somewhat safe: OR=1.53 (95% CI=1.04; 2.25), Feel unsafe: OR=1.51 (95% CI=1.04; 2.25), p=.04 (Crime/personal safety **+**) | Moderating effects. Table 2–adjusted models. |
| 22 | Harvard Alumni Health Study (HAHS) Lee et al., 2009 [65] | Cross-sectional: 1988: N=4918 1993: N=4997 (likely mixed/not reported) Longitudinal: (N=3448) (likely mixed/not reported) Mean age: 70 years 0% female 71% response rate Community-dwellers *Multiple locations,* USA | Cross-sectional at two time-points and longitudinal Cluster: convenience Individuals: not reported Stratification: none Neighbourhood definition: county level | Age, smoking | *Self-report [HAHS questionnaire; validated]:*  Total PA (30+ mins/d*5 d/wk; Yes/No) 🡪 Total PA *TotalPA(30+ mins/d*5d/wk; Yes/No)*  Total walking (30+ mins/d*5 d/wk; Yes/No) 🡪 Total walking *TotalWalking(30+ mins/d*5 d/wk; Yes/No)* Total PA change (kcal/wk) (longitudinal) 🡪 Total PA *TotalPAChange(kcal/wk)* Mean distance walked (miles/wk) (longitudinal) 🡪 Total walking *TotalWalkingChange(kcal/wk)* | *Objective [Sprawl Index; unvalidated]:*  1. Urban sprawl 🡪 Urbanisation | None | Multivariate logistic regression | ***Moderated effects with TotalPA(30+ mins/d*5d/wk; Yes/No):*** Cross-sectional: 1. 1988: Urban sprawl (Ref: high sprawl, n=497)—OR (95% CIs): Medium sprawl (n=3042): OR=0.93 (0.75; 1.14),  Low sprawl (n=1379): OR=1.15 (0.92; 1.45), p=.01 (Urbanisation **0*0.10; 0*0.62; 0*0.28**)  1. 1993: Urban sprawl (Ref: high sprawl, n=551): Medium sprawl (n=3126): OR=1.02 (95% CI=0.84; 1.25), Low sprawl (n=1320): OR=1.10 (95% CI=0.88; 1.37), p=.30 (Urbanisation **0*0.11; 0*0.63; 0*0.26**)  ***Main effects with TotalWalking(30+ mins/d*5d/wk; Yes/No):*** 1. 1988: Urban sprawl (Ref: high sprawl, n=497)—OR (95% CIs): Medium sprawl (n=3042): OR=1.01 (0.80; 1.28),  Low sprawl (n=1379): OR=1.53 (1.19; 1.96), p<.001 (Urbanisation **+*0.10; +*0.62; +*0.28**)  1. 1993: Urban sprawl (Ref: high sprawl, n=551): Medium sprawl (n=3126): OR=1.02 (0.81; 1.27),  Low sprawl (n=1320): OR=1.38 (1.09; 1.76), p<.001 (Urbanisation **+*0.11; +*0.63; +*0.26**)  ***Main effects with TotalPAChange(kcal/wk):*** Longitudinal: 1. Urban sprawl: Moved to more sprawling country (n=135): 230 (208), P>.05 (Urbanisation **0*0.04**) Remained at same sprawl (n=3240): -76 (42), P>.05 (Urbanisation **0*0.94**) Remained at same sprawl (n=73): -157 (283), P>.05 (Urbanisation **0*0.02**)  ***Main effects with TotalWalkingChange(miles/wk):*** Longitudinal: 1. Urban sprawl: Moved to more sprawling country (n=135): 0.08 (0.5), P>.05 (Urbanisation **0*0.04**) Remained at same sprawl (n=3240): 0.03 (0.1), P>.05 (Urbanisation **0*0.94**) Remained at same sprawl (n=73): -0.6 (0.7), P>.05 (Urbanisation **0*0.02**) | Cross-sectional results: Table 2. Longitudinal results: Table 3. |
| 23 | Health and Retirement Study Latham et al., 2015 [143] | N=5922 (mixed) Mean age: 70 years 67% female 71% response rate Community-dwellers *Multiple locations,* USA | Cross-sectional Cluster: purposive Individuals: random (original study); convenience Stratification: age Neighbourhood definition: 20mins walk from home | Age, sex, socioeconomic status (included education), race/ethnicity, married/partner status | *Self-report [unnamed questionnaire; unvalidated]:*  Frequency of PA (score index) 🡪 Total PA *TotalPA(score)* | *Perceived [adapted from Project on Human Development in Chicago Neighbourhoods; reliable]:*  1. Physical disorder, comprising a) vandalism/graffiti, b) rubbish/litter, c) vacant/deserted homes, and d) crime 🡪 Crime/personal safety | None | Ordinary least squares regression  *Note. PA outcome given intensity weights, however, these produced small estimates and increased skewness – therefore, scaled weights were used which improved model fit (specific data not reported).* | ***Main effect with TotalPA(score):*** 1. Physical disorder: b=-0.07 (SE=0.03), p>.05 (Crime/personal safety **0**) | Table 4—Model 7. |
| 24 | Health and Wellbeing Surveillance System data Nathan et al., 2012 [49] | N=2918 (mixed) 65+ years 56% female 80-84% response rates Community-dwellers Western Australia, Australia | Cross-sectional Cluster: none Individuals: random Stratification: urbanisation and health service area Neighbourhood definition: 400m and 800m buffers | Age, sex, education, marital status, self-rated health, use of assistive equipment | *Self-report [Active Australia Survey; validated]:*  Walking (150+ mins/wk; Yes/No) 🡪 Total walking *TotalWalking(150+ mins/wk; Yes/No)* Walking (some; Yes/No) 🡪 Total walking *TotalWalking(some; Yes/No)* | *Objective [GIS; Sensis Pty. Ltd. Data—Australian Yellow Pages; unvalidated]:*  1. Food retail 🡪 Food outlets access/availability 2. General retail 🡪 Shops/commercial access/availability 3. Medical care services 🡪 Health and aged care access/availability 4. Financial services 🡪 Government/finance services access/availability 5. General services 🡪 General services access/availability 6. Social infrastructure 🡪 Social recreational facilities access/availability  7. Destination mix 🡪 Land-use mix—destination diversity | Sex:  Male (n=1287), Female (n=1631) | Multivariate logistic regression | *No significant moderating effects.* ***Main effects with TotalWalking(some; Yes/No):*** 1. Food retail—OR (95% CIs): *400m:* OR=1.05 (0.84; 1.30), p=.676 *800m:* OR=0.98 (0.83; 1.15), p=.767 (Food outlets access/availability **0*0.5; 0*0.5**) 2. General retail: *400m:* OR=1.00 (0.77; 1.29), p=.979 *800m:* OR=1.00 (0.85; 1.18), p=.996 (Shops/commercial access/availability **0*0.5; 0*0.5**) 3. Medical care services: *400m:* OR=0.96 (0.80; 1.15), p=.653 *800m:* OR=1.01 (0.85; 1.20), p=.893 (Health and aged care access/availability **0*0.5; 0*0.5**) 4. Financial services: *400m:* OR=1.10 (0.71; 1.70), p=.675 *800m:* OR=0.93 (0.75; 1.15), p=.507 (Government/finance services access/availability **0*0.5; 0*0.5**) 5. General services: *400m:* OR=1.33 (1.07; 1.66), p=.011 *800m:* OR=1.20 (1.02; 1.42), p=.027 (Destinations/services (overall/unspecific) access/availability **+*0.5; +*0.5**) 6. Social infrastructure: *400m:* OR=1.02 (0.83; 1.24), p=.884 (Social recreational access/availability **0*0.5**) *800m:* OR=1.19 (1.01; 1.40), p=.043 (Social recreational access/availability **+*0.5**) 7. Destination mix: *400m:* OR=1.02 (0.97; 1.08), p=.473 *800m:* OR=1.02 (0.98; 1.06), p=.473 (Land-use mix—destination diversity **0*0.5; 0*0.5**)  ***Main effects with TotalWalking(150+ mins/wk; Yes/No):*** 1. Food retail: *400m:* OR=0.86 (0.69; 1.08), p=.188 *800m:* OR=0.87 (0.74; 1.03), p=.109 (Food outlets access/availability **0*0.5; 0*0.5**) 2. General retail: *400m:* OR=0.80 (0.62; 1.05), p=.107 *800m:* OR=0.90 (0.76; 1.07), p=.248 (Shops/commercial access/availability **0*0.5; 0*0.5**) 3. Medical care services: *400m:* OR=0.76 (0.63; 0.92), p=.005 *800m:* OR=0.83 (0.70; 0.99), p=.036 (Health and aged care access/availability **-*0.5; -*0.5**) 4. Financial services: *400m:* OR=0.85 (0.55; 1.33), p=.480 *800m:* OR=0.96 (0.77; 1.20), p=.738 (Government/finance services access/availability **0*0.5; 0*0.5**) 5. General services: *400m:* OR=1.00 (0.81; 1.24), p=.971 *800m:* OR=1.06 (0.89; 1.25), p=.526  (General services access/availability **0*0.5; 0*0.5**) 6. Social infrastructure: *400m:* OR=1.02 (0.83; 1.24), p=.861 *800m:* OR=0.90 (0.76; 1.06), p=.194 (Social recreational access/availability **0*0.5; 0*0.5**) 7. Destination mix: *400m:* OR=0.96 (0.90; 1.01), p=.124 *800m:* OR=0.97 (0.94; 1.01), p=.178 (Land-use mix—destination diversity **0*0.5; 0*0.5**) | Buffer effects. Table 3 and 4. |
| 25 | Health and Wellbeing Surveillance System data Villanueva et al., 2014 [171] | n=3611 (sub-sample; mixed) 65+ years 56% female 80-84% response rates Community-dwellers Western Australia, Australia | Cross-sectional Cluster: none Individuals: random Stratification: urbanisation and health service area Neighbourhood definition: 200m, 400m, 800m, and 1600m buffers | Age, sex, education, area-level SES | *Self-report [Active Australia Survey; validated]:*  Walking (>0 mins/wk; Yes/No) 🡪 Total walking *TotalWalking(>0 mins/wk; Yes/No)* | *Objective [ArcGIS; adapted version of Frank et al.’s walkability index; unvalidated]:*  1. Walkability 🡪 Walkability | None | Binary logistic regression | ***Main effects with TotalWalking(>0mins/wk; Yes/No):*** 1. Walkability—OR (95% CIs): 200m: OR=1.06 (1.02; 1.11), p=.008 400m: OR=1.08 (1.03; 1.13), p=.001 800m: OR=1.07 (1.02; 1.11), p=.003 1600m: OR=1.08 (1.04; 1.13), p<.001 (Walkability **+*0.25; +*0.25; +*0.25; +*0.25**) | Table 2. |
| 26 | Hong Kong Elderly Study (name assigned) Cerin et al., 2013 [124] | N=484 (urban) 65+ years 58% female 78% response rate Community-dwellers Hong Kong, China | Cross-sectional Cluster: purposive Individuals: random Stratification: walkability and SES Neighbourhood definition: 10-15mins walk from home | Age, sex, education | *Self-report [Neighbourhood Walkability Questionnaire-Chinese Seniors version; validated]:*  Within-neighbourhood walking (mins/wk) 🡪 Total walking *TotalWalking(mins/wk)* Within-neighbourhood walking (mins/wk; odds of being a walker) 🡪 Total walking *TotalWalking(Non-WalkerOdds; Yes/No)* | *Objective [Environment in Asia Scan Tool—Hong Kong version; reliability-tested]:*  1. Stray animals 🡪 Crime/personal safety 2. Street lights 🡪 Street lighting 3. Signs of crime 🡪 Crime/personal safety 4. Pedestrian safety 🡪 Traffic/pedestrian safety 5. Traffic hazards 🡪 Traffic/pedestrian safety 6. Good path conditions 🡪 Pavement/footpath quality 7. Physical barriers to walking 🡪 No physical barriers to walking 8. Public facilities 🡪 Walk-friendly infrastructure 9. Indoor covered places for walking 🡪 Walk-friendly infrastructure 10. Natural sites 🡪 Greenery and aesthetically pleasing scenery  11. Trees 🡪 Greenery and aesthetically pleasing scenery 12. Park 🡪 Parks/public open space access/availability 13. Building attractiveness 🡪 Greenery and aesthetically pleasing scenery 14. Litter 🡪 Greenery and aesthetically pleasing scenery 15. Recreational facilities other than parks 🡪 Recreational facilities access/availability  16. Perceptible pollution 🡪 Pollution | None | Zero-inflated negative binomial regression models accounting for clustering and positive skewness of within-neighbourhood walking | ***Main effects with TotalWalking(mins/wk):*** 1. Stray animals—e (95% CIs): e=0.985 (0.970; 0.999), p<.05 (Crime/personal safety **+**) 2. Street lights: e=1.005 (1.002; 1.008), p<.001 (Street lighting **+**) 3. Signs of crime: e=1.040 (1.017; 1.063), p<.001 (Crime/personal safety **-**) 4. Pedestrian safety: e=not reported, p>.05 (Traffic/pedestrian safety **0**) 5. Traffic hazards: e=not reported, p>.05 (Traffic/pedestrian safety **0**) 6. Good path conditions: e=not reported, p>.05 (Pavement/footpath quality **0**) 7. Physical barriers to walking: e=not reported, p>.05 (No physical barriers to walking **0**) 8. Public facilities: e=not reported, p>.05 (Walk-friendly infrastructure **0**) 9. Indoor covered places for walking: e=not reported, p>.05 (Walk-friendly infrastructure **0**) 10. Natural sites: e=not reported, p>.05 (Greenery and aesthetically pleasing scenery **0**) 11. Trees: e=not reported, p>.05 (Greenery and aesthetically pleasing scenery **0**) 12. Park: e=not reported, p>.05 (Parks/public open space access/availability **0**) 13. Building attractiveness: e=not reported, p>.05 (Greenery and aesthetically pleasing scenery **0**) 14. Litter: e=not reported, p>.05 (Greenery and aesthetically pleasing scenery **0**) 15. Recreational facilities other than parks: e=not reported, p>.05 (Recreational facilities access/availability **0**) 16. Perceptible pollution: e=not reported, p>.05 (Pollution **0**)  ***Main effects with TotalWalking(Non-WalkerOdds; Yes/No):*** 1. Stray animals: e=1.055 (1.000; 1.067), p<.05 (Crime/personal safety **+**) 2. Street lights: e=not reported, p>.05 (Street lighting **0**) 3. Signs of crime: e=0.774 (0.682; 0.878), p<.001 (Crime/personal safety **-**) 4. Pedestrian safety: e=not reported, p>.05 (Traffic/pedestrian safety **0**) 5. Traffic hazards: e=not reported, p>.05 (Traffic/pedestrian safety **0**) 6. Good path conditions: e=0.940 (0.894; 0.987), p<.05 (Pavement/footpath quality **+**) 7. Physical barriers to walking: e=1.037 (1.010; 1.064), p<.01 (No physical barriers to walking **+**) 8. Public facilities: e=not reported, p>.05 (Walk-friendly infrastructure **0**) 9. Indoor covered places for walking: e=not reported, p>.05 (Walk-friendly infrastructure **0**) 10. Natural sites: e=not reported, p>.05 (Greenery and aesthetically pleasing scenery **0**) 11. Trees: e=not reported, p>.05 (Greenery and aesthetically pleasing scenery **0**) 12. Park: e=not reported, p>.05 (Parks/public open space access/availability **0**) 13. Building attractiveness: e=not reported, p>.05 (Greenery and aesthetically pleasing scenery **0**)  14. Litter: e=not reported, p>.05 (Greenery and aesthetically pleasing scenery **0**) 15. Recreational facilities other than parks: e=not reported, p>.05 (Recreational facilities access/availability **0**) 16. Perceptible pollution: e=not reported, p>.05 (Pollution **0**) | Table 3. |
| 27 | Kasama study Tsunoda et al., 2012 [169] | N=421 (mixed) 65+ years 52.5% female 20.9% response rate Community-dwellers Kasama City and a rural region in Ibaraki Prefecture, Japan | Cross-sectional Cluster: none Individuals: random Stratification: none Neighbourhood definition: 10-15mins walk from home | Age, sex, education, work status, clinical histories: heart disease, stroke, low back disease, knee disease, hip disease | *Self-report [PASE questionnaire—Japanese version; validated]:*  Walking (60+ mins/wk; Yes/No) 🡪 Total walking *TotalWalking(60+ mins/wk; Yes/No)* Walking (150+ mins/wk; Yes/No) 🡪 Total walking *TotalWalking(150+ mins/wk; Yes/No)* | *Perceived [IPAQ—Japanese version; validated]:*  1. Residential density 🡪 Residential density 2. Access to shops 🡪 Shops/commercial access/availability 3. Access to public transport 🡪 Public transport access/availability 4. Access to recreational facilities 🡪 Recreational facilities access/availability  5. Presence of sidewalks 🡪 Walk-friendly infrastructure 6. Presence of bike lanes 🡪 Cycle-friendly infrastructure 7. Traffic safety 🡪 Traffic/pedestrian safety 8. Crime safety 🡪 Crime/personal safety 9. Presence of hills 🡪 No physical barriers to walking 10. Seeing people exercise 🡪 Crime/personal safety 11. Aesthetics 🡪 Greenery and aesthetically pleasing scenery | None | Multivariate logistic regression. Continuous environmental exposure variables categorised. | ***Main effects with TotalWalking(60+ mins/wk; Yes/No):*** 1. Residential density (Reference: high)—OR (95% CIs): Low: OR=0.96 (0.43; 2.16), p>.05 (Residential density **0**) 2. Access to shops (Reference: poor): Good: OR=1.02 (0.65; 1.60), p>.05 (Shops/commercial access/availability **0**) 3. Access to public transport (Reference: poor): Good: OR=0.78 (0.48; 1.24), p>.05 (Public transport access/availability **0**) 4. Access to recreational facilities (Reference: poor): Good: OR=1.15 (0.74; 1.81), p>.05 (Recreational facilities access/availability **0**) 5. Presence of sidewalks (Reference: no): Yes: OR=0.72 (0.44; 1.18), p>.05 (Walk-friendly infrastructure **0**) 6. Presence of bike lanes (Reference: no): Yes: OR=0.97 (0.62; 1.53), p>.05 (Cycle-friendly infrastructure **0**) 7. Traffic safety (Reference: not safe): Safe: OR=1.64 (1.03; 2.60), p<.05 (Traffic/pedestrian safety **+**) 8. Crime safety (Reference: not safe): Safe: OR=0.69 (0.41; 1.15), p>.05 (Crime/personal safety **0**) 9. Presence of hills (Reference: yes): No: OR=1.44 (0.77; 2.69), p>.05 (No physical barriers to walking **0**) 10. Seeing people exercise (Reference: no): Yes: OR=0.89 (0.49; 1.60), p>.05 (Crime/personal safety **0**) 11. Aesthetics (Reference: poor): Good: OR=2.12 (1.34; 3.36), p<.05 (Greenery and aesthetically pleasing scenery **+**)  ***Main effects with TotalWalking(150+ mins/wk; Yes/No):*** 1. Residential density (Reference: high): Low: OR=0.82 (0.39; 1.72), p>.05 (Residential density **0**) 2. Access to shops (Reference: poor): Good: OR=0.67 (0.45; 1.02), p>.05 (Shops/commercial access/availability **0**) 3. Access to public transport (Reference: poor): Good: OR=0.64 (0.42; 0.98), p<.05 (Public transport access/availability **-**) 4. Access to recreational facilities (Reference: poor): Good: OR=1.20 (0.80; 1.80), p>.05 (Recreational facilities access/availability **0**) 5. Presence of sidewalks (Reference: no): Yes: OR=0.73 (0.48; 1.13), p>.05 (Walk-friendly infrastructure **0**) 6. Presence of bike lanes (Reference: no): Yes: OR=0.95 (0.63; 1.43), p>.05 (Cycle-friendly infrastructure **0**) 7. Traffic safety (Reference: not safe): Safe: OR=1.46 (0.96; 2.21), p>.05 (Traffic/pedestrian safety **0**) 8. Crime safety (Reference: not safe): Safe: OR=0.94 (0.60; 1.48), p>.05 (Crime/personal safety **0**) 9. Presence of hills (Reference: yes): No: OR=1.12 (0.63; 2.02), p>.05 (No physical barriers to walking **0**) 10. Seeing people exercise (Reference: no): Yes: OR = 1.04 (0.62; 1.76), p>.05 (Crime/personal safety **0**) 11. Aesthetics (Reference: poor): Good: OR=2.00 (1.33; 3.02), p<.05 (Greenery and aesthetically pleasing scenery **+**) | Table 3. |
| 28 | KNHANES (Korean National Health & Nutrition Examination Survey) 2007/2008 Yeom et al., 2011 [173] | N=2241 (mixed) 65+ years 60% female 97.7% response rate Community-dwellers *Multiple locations,* South Korea | Cross-sectional at two time points Cluster: purposive Individuals: purposive Stratification: age and 20 households from 192 regions selected Neighbourhood definition: province level | None | *Self-report [World Health Organisation’s IPAQ; validated]:*  MPA (mins/wk) 🡪 Total MVPA *TotalMVPA(mins/wk)*  Walking (mins/wk) 🡪 Total walking *TotalWalking(mins/wk)* | *Objective [GIS—Korean Government data; unvalidated]:*  1. Living in metropolitan cities 🡪 Urbanisation | None | Chi-square test | ***Main effect with TotalMVPA(mins/wk):*** 1. Urbanisation: Χ^2^=7.8, p=.005 (Urbanisation **-**)  ***Main effect with TotalWalking(mins/wk):*** 1. Urbanisation: Χ^2^=0.9, p=.346 (Urbanisation **0**) | Table 3. |
| 29 | LL-FDI (Late-Life Function and Disability Instrument) validation study Morris et al., 2008 [150] | n=136 (sub-sample) (mixed) Mean age: 69.6 years 100% female 51.4% response rate Community-dwellers *Undisclosed location,* USA | Cross-sectional Cluster: convenience Individuals: purposive Stratification: sex and multiple sclerosis diagnosis (related to a different sub-sample than this older women cohort)  Neighbourhood definition: 10-15mins walk from home | None | *Objective [ActiGraph accelerometer; validated]:*  Mean accelerometer counts/d 🡪 Total PA *TotalPA(counts/d)* | *Perceived [NEWS questionnaire; validated]:*  1. Residential density 🡪 Residential density 2. Land-use mix—diversity 🡪 Land-use mix—diversity 3. Access to services 🡪 Destinations/services (overall/unspecific) access/availability 4. Street connectivity 🡪 Street connectivity 5. Walking/cycling facilities 🡪 Cycle/walk-friendly infrastructure 6. Aesthetics 🡪 Greenery and aesthetically pleasing scenery 7. Safety from traffic 🡪 Traffic/pedestrian safety 8. Safety from crime safety 🡪 Crime/personal safety | None | Pearson’s correlation | ***Main effects with TotalPA(counts/d):*** 1. Residential density: r=.05, p>.05 (Residential density **0**) 2. Land-use mix—diversity: r=.04, p>.05 (Land-use mix—destination diversity **0**) 3. Access to services: r=.08, p>.05  (Destinations/services (overall/unspecific) access/availability **0**) 4. Street connectivity: r=.25, p<.01  (Street connectivity **+**) 5. Walking/cycling facilities: r=.21, p<.05  (Cycling/Walk-friendly infrastructure **+**) 6. Aesthetics: r=.21, p<.05  (Greenery and aesthetically pleasing scenery **+**) 7. Safety from traffic: r=.11, p>.05  (Traffic/pedestrian safety **0**) 8. Safety from crime: r=.00, p>.05  (Crime/personal safety **0**) | Table 3. *Note.* Pearson’s correlation results were retained vs. multiple regression because the latter did not adjust for key sociodemographics |
| 30 | LL-FDI study Hall & McAuley, 2010 [132] | n=128 (sub-sample) (mixed) Mean age: 70 years 100% female 51.4% response rate Community-dwellers *Undisclosed location,* USA | Cross-sectional Cluster: none Individuals: convenience  Stratification: sex and multiple sclerosis diagnosis (related to a different sub-sample than this older women cohort)  Neighbourhood definition: 10-15mins walk from home (perceived); 1km buffer (objective) | None | *Objective [ActiGraph accelerometer; validated]:*  Steps/d 🡪 Total walking *TotalWalking(steps/d)* | *Objective [GIS; unvalidated]:*  1. Number of schools 🡪 Education facilities access/availability 2. Number of parks 🡪 Parks/public open space access/availability 3. Number of walking paths 🡪 Walk-friendly infrastructure 4. Number of exercise/gym facilities 🡪 Recreational facilities access/availability  5. Number of recreation areas 🡪 Recreational facilities access/availability  *Perceived [NEWS questionnaire; validated]:*  6. Residential density 🡪 Residential density 7. Land-use mix: access 🡪 Destinations/services (overall/unspecific) access/availability 8. Land-use mix: diversity 🡪 Land-use mix—destination diversity 9. Street connectivity 🡪 Street connectivity 10. Walking/cycling facilities 🡪 Cycle/walk facilities/infrastructure 11. Aesthetics 🡪 Greenery and aesthetically pleasing scenery 12. Pedestrian safety from traffic 🡪 Traffic/pedestrian safety 13. Safety from crime 🡪 Crime/personal safety | None | MANOVA | ***Main effects with TotalWalking(steps/d):*** Objective environment: 1. Number of schools: <10,000 steps/d (1.57±2.00) vs. 10,000+ steps/d (1.66±1.95), f=0.05, p=.83 (Education facilities access/availability **0**) 2. Number of parks: <10,000 steps/d (2.53±2.55) vs. 10,000+ steps/d (3.29±2.81), f=2.13, p=.15 (Parks/public open space access/availability **0**) 3. Number of walking paths: <10,000 steps/d (1.18±1.40) vs. 10,000+ steps/d (1.89±1.62), f=5.88, p=.02 (Walk-friendly infrastructure **+**) 4. Number of exercise/gym facilities: <10,000 steps/d (0.35±0.67) vs. 10,000+ steps/d (0.43±0.74), f=0.29, p=.59 (Recreational facilities access/availability **0**) 5. Number of exercise/gym facilities: <10,000 steps/d (0.35±0.67) vs. 10,000+ steps/d (0.43±0.74), f=0.0029, p=.90 (Recreational facilities access/availability **0**)  Perceived environment: 6. Residential density: <10,000 steps/d (187.48±22.33) vs. 10,000+ steps/d (193.06±39.22), f=1.01, p=.32 (Residential density **0**) 7. Land-use mix – access: <10,000 steps/d (2.53±0.59) vs. 10,000+ steps/d (2.66±0.63), f=1.18, p=.28 (Destinations/services (overall/unspecific) access/availability **0**) 8. Land-use mix – diversity: <10,000 steps/d (2.84±1.08) vs. 10,000+ steps/d (2.82±0.99), f=0.01, p=.93 (Land-use mix—destination diversity **0**) 9. Street connectivity: <10,000 steps/d (2.45±0.65) vs. 10,000+ steps/d (2.77±0.66), f=6.04, p=.02 (Street connectivity **+**) 10. Walking/cycling facilities: <10,000 steps/d (2.30±0.98) vs. 10,000+ steps/d (2.66±0.92), f=3.53, p=.06 (Cycling/Walk-friendly infrastructure **0**) 11. Aesthetics: <10,000 steps/d (3.11±0.60) vs. 10,000+ steps/d (3.26±0.64), f=1.41, p=.24 (Greenery and aesthetically pleasing scenery **0**) 12. Pedestrian safety from traffic: <10,000 steps/d (2.77±0.60) vs. 10,000+ steps/d (3.04±0.78), f=4.39, p=.04 (Traffic/pedestrian safety **+**) 13. Safety from crime: <10,000 steps/d (3.26±0.49) vs. 10,000+ steps/d (3.33±0.55), f=0.50, p=.48 (Crime/personal safety **0**) | Table 2. |
| 31 | Malaysian National Health and Morbidity Survey III 2006 data Kaur et al., 2015 [139] | N=4831(mixed) 60-80+ years 53% female 97.5% response rate Community-dwellers *Multiple locations,* Malaysia | Cross-sectional Cluster: purposive Individuals: purposive Stratification: urbanisation Neighbourhood definition: enumeration block (80-120 living quarters with ≈600 individuals) | Age, sex, education, ethnicity, marital status, household income | *Self-report [World Health Organisation STEPS questionnaire; validated]:*  Inactivity (<150 mins/wk) 🡪 Total MVPA *TotalMVPA(<150 mins/wk; Yes/No)* | *Objective [Malaysian Department of Statistics; unvalidated]:*  1. Locality 🡪 Urbanisation | None | Multivariate logistic regression | ***Main effects with TotalMVPA(<150 mins/wk; Yes/No):*** 1. Locality (Ref: Rural)—OR (95% CIs): Urban: OR=1.318 (1.025; 1.696), p=.031 (Urbanisation **-**) | Table 3. |
| 32 | Melbourne older adults study 1 (name assigned) Bird et al., 2009 [121] | N=333 (urban) Mean age: 72 years  59% female Response rate not reported Community-dwellers apart from n=1 (nursing home) Melbourne, Australia | Cross-sectional Cluster: purposive Individuals: convenience Stratification: cultural group Neighbourhood definition: 15-20mins walk from home | Age, number of years in Australia, self-reported health, cultural group (Anglo vs. non-Anglo) | *Self-report [International Physical Activity Questionnaire (IPAQ); validated]:*  Total PA (mins/wk) 🡪 Total PA  *TotalPA(mins/wk)* | *Perceived [NEWS questionnaire—modified version—unvalidated; St. Louis Scale—modified version—unvalidated]:* NEWS items:  1. Well maintained footpaths 🡪 Pavement/footpath quality 2. Trees shading footpaths 🡪 Walk-friendly infrastructure 3. Seeing and speaking to others when neighbourhood walking 🡪 Crime/personal safety  St. Louis item: 4. No safe place to exercise 🡪 Crime/personal safety | None | Hierarchical regression. Outcome square root transformed as not normally distributed. No adjustment for LGA cluster | ***Main effects with TotalPA(mins/wk):*** 1. Well maintained footpaths—b (95% CIs): b=-0.15 (-6.81; -1.25), p<.001 (Pavement/footpath quality **-**) 2. Trees shading footpaths: b=-0.15 (-6.81; -1.25), p=.01 (Walk-friendly infrastructure **-**) 3. Seeing and speaking to others when neighbourhood walking: b=0.04 (-1.99; 4.52), p=.45 (Crime/personal safety **0**) 4. No safe place to exercise: b=-0.18 (-7.33; -1.74), p<.001 (Crime/personal safety **+**) | Table 7—model 3. |
| 33 | Melbourne older adults study 1 (name assigned) Bird et al., 2010 [122] | N=268 (urban) Mean age: 72 years  % female: not reported Community-dwellers Melbourne, Australia | Cross-sectional; Cluster: convenience Individuals: convenience Stratification: cultural group Neighbourhood definition: 15-20mins walk from home | Age, number of years in Australia, self-reported health, cultural group (Anglo vs. non-Anglo) | *Self-report [IPAQ—translated version; validated]:*  Total walking (150+ mins/wk; Yes/No) 🡪 Total walking *TotalWalking(150+ mins/wk; Yes/No)* | *Perceived [NEWS questionnaire—validated; St. Louis Scale—validated]:* NEWS items:  1. Unsafe to walk at night 🡪 Crime/personal safety 2. Safe to walk during day 🡪 Crime/personal safety 3. Crime rate 🡪 Crime/personal safety 4. Attractive natural sights 🡪 Greenery and aesthetically pleasing scenery 5. Streets well lit at night 🡪 Street lighting | None | Chi-square test, Mann-Whitney test, Kruskal-Wallis test  *Note: Walk mins/week non-normally distributed, therefore data described as medians and interquartile ranges.* *Environmental data also non-normally distributed (fails to explicitly mention which attributes…)* | ***Main effects with TotalWalking(150+ mins; Yes/No):*** 1. Unsafe to walk at night: Χ^2^=4.75, p=.093 (Crime/personal safety **0**) 2. Safe to walk during day: Χ^2^=3.19, p=.074 (Crime/personal safety **0**) 3. Crime rate: Χ^2^=5.07, p=.079 (Crime/personal safety **0**) 4. Attractive natural sights: <150 min: U=157 vs. ≥150 min: U=177, p=.048 (Greenery and aesthetically pleasing scenery **+**) 5. Streets well lit at night: ≥10 min <150 min: H=125 vs. ≥150 min: H=140, p=.095  (Street lighting **0**) | *Two paragraphs above Discussion sub-heading.* |
| 34 | MOBILIZE (Maintenance of Balance, Independent Living, Intellect and Zest in the Elderly) Boston study Procter-Gray et al., 2015 [158] | N=745 (urban) Mean age: 78 years 64% female 68% response rate  Community-dwellers Boston, USA | Cross-sectional Cluster: convenience (8km radius from research institute) Individuals: random Stratification: none Neighbourhood definition: census block | Age, sex, ethnicity, BMI alcohol use, balance, unable to do the chair-stand test without arms, gait speed, activities of daily living, short performance battery score, illness, bodily pain, comorbidities, self-rated health, peripheral neuropathy, foot pain, daily medications, impaired cognition, falls efficacy score | *Self-report [unnamed questionnaire; unvalidated]:*  Habitual walking (5+ d/wk; Yes/No) 🡪 Total walking *TotalWalking(5+ d/wk; Yes/No)* | *Objective [GIS—Massachusetts GIS, InfoUSA, US Post Office; unvalidated]:*  1. Bus stop 🡪 Public transport access/availability 2. Subway 🡪 Public transport access/availability 3. Hospital 🡪 Health and aged care access/availability 4. Shopping centre or mall 🡪 Shops/commercial access/availability 5. Post office 🡪 Shops/commercial access/availability 6. Public park (1+ acre) 🡪 Parks/public open space access/availability 7. Grocery/convenience store 🡪 Shops/commercial access/availability 8. Town hall 🡪 Government/finance services access/availability 9. Public library 🡪 Education facilities access/availability 10. % housing units vacant 🡪 Residential density | None | Multivariate logistic regression | ***Main effects with TotalWalking(5+ d/wk; Yes/No):*** 1. Nearest bus stop—OR (95% CIs): OR=0.74 (0.52; 1.04), p>.05 (Public transport **0**) 2. Nearest subway: OR=0.88 (0.79; 0.97), p<.01 (Public transport **+**) 3. Nearest hospital: OR=0.84 (0.76; 0.94), p<.01 (Health and aged care access/availability **+**) 4. Nearest shopping centre or mall: OR=0.93 (0.85; 1.02), p>.05 (Shops/commercial access/availability **0**) 5. Nearest post office: OR=0.82 (0.73; 0.92), p<.01 (Shops/commercial access/availability **+**) 6. Nearest public park (1+ acre): OR=0.77 (0.53; 1.13), p>.05 (Parks/public open space access/availability **0**) 7. Nearest grocery/convenience store: OR=0.77 (0.61; 0.99), p<.05 (Shops/commercial access/availability **+**) 8. Nearest town hall: OR=0.93 (0.86; 1.02), p>.05 (Government/finance services access/availability **0**) 9. Nearest public library: OR=0.95 (0.76; 1.18), p>.05 (Education facilities access/availability **0**) 10. % housing units vacant (Ref: <5%): *5-10%:* OR=1.59 (1.11; 2.29), p<.05 (Residential density **-*0.5**) *>10%:* OR=1.31 (0.83; 2.07), p>.05 (Residential density **0*0.5**) | Table 2 |
| 35 | Neighbourhoods and Physical Activity in Elderly Men Michael et al., 2010 [64] | N=422 (urban) 65+ years 0% female 10-15% response rate Community-dwellers Portland, USA | Longitudinal Cluster: convenience Individuals: purposive Stratification: clinical site (Portland only) and ethnicity (minorities represented) Neighbourhood definition: 0.125mile, 0.25mile, and 0.5mile buffers | Age, education, race, occupation, marital status, self-rated health, BMI, smoking, alcohol/wk, chronic conditions, physical function *Note. Following variables were not explicitly reported, authors’ reported adjusting for covariate sets including demographic and socioeconomic characteristics, health behaviours, chronic conditions and self-reported health, and physical function.* | *Self-report [2 questions from PASE questionnaire; validated]:*  Change in total walking (30+ mins/d; Yes/No) 🡪 Total walking *TotalWalking(change; 30+ mins/d; Yes/No)* | *Objective [GIS, Regional Land Scale Information Database; unvalidated]:*  1. Number of recreational facilities 🡪 Recreational facilities access/availability  2. Park proximity 🡪 Parks/public open space access/availability 3. Trail proximity 🡪 Parks/public open space access/availability | SES:  Low (n=211), High (n=211)  Park proximity*SES Trail proximity*SES | Log-binomial regression | *Significant moderating effects for: Park proximity (0.125 mile)*SES:* p<.10 *Trail proximity (0.5 mile)*SES:* p<.10  ***Main and moderated effects with TotalWalking(change; 30+ mins/d; Yes/No):*** 1. Number of Recreational facilities—RR (95% CIs): *Total participants + 0.25 mile:* RR=not reported, p>.05 *Total participants + 0.5 mile:* RR=not reported, p>.05 (Recreational facilities access/availability **0*0.5; 0*0.5**) 2. Park proximity: *High SES + 0.125 mile:* RR=1.22 (1.01; 1.47), p<.05 (Parks/public open space access/availability **+*0.167**) *Low SES + 0.125 mile:* RR=0.89 (0.70; 1.13), p>.05 *Total participants + 0.25 mile:* RR=not reported, p>.05 *Total participants + 0.5 mile:* RR=not reported, p>.05 (Parks/public open space access/availability **0*0.833**) 3. Trail proximity: *Total participants + 0.25 mile:* RR=not reported, p>.05 *High SES + 0.5 mile:* RR=1.34 (1.16; 1.55), p<.05 (Parks/public open space access/availability **+*0.25**) *Low SES + 0.5 mile:* RR=0.93 (0.71; 1.23), p>.05 (Parks/public open space access/availability **0*0.75**) | Moderating and buffer effects. In-text, pp. 657. |
| 36 | Netherlands Housing Survey (WoON) data Jongeneel-Grimen et al., 2013 [136] | n=6830 (2006; sub-sample)), n=8994 (2009; sub-sample) (urban) 60-84 years 53-56% female 62.6-70.9% response rate Community-dwellers *Multiple municipalities,* Netherlands | Cross-sectional measured at two time-points Cluster: purposive Individuals: random Stratification: age, gender, country of birth and municipality Neighbourhood definition: postal code | Age, sex education, employment status, household income | *Self-report (WoON questionnaire; unvalidated):*  Total PA (hrs/wk) 🡪 Total PA *TotalPA(hrs/wk—prevalence) TotalPA(hrs/wk—frequency)* | *Perceived [WoON questionnaire; unvalidated]:*  1. Traffic safety 🡪 Traffic/pedestrian safety 2. Change in traffic safety 🡪 Traffic/pedestrian safety | None.  *Notes:* Age:  *In 2006:*  18-34 y (n≈7408), 35-59 y (n≈11,070),  60-84 y (n≈6830)  *In 2009:*  18-34 y (n≈9134), 35-59 y (n≈13,648),  60-84 y (n≈8994) | Multilevel logistic regression, multilevel zero-truncated negative binomial regression | ***Main effect with TotalPA(hrs/wk—prevalence):*** 1. Traffic safety—OR (95% CIs): OR=1.028 (0.953; 1.107), p>.05 (Traffic/pedestrian safety **0**)  *Main effect with TotalPA(hrs/wk—frequency):* 1. Traffic safety: Activity intensity ratio=1.017 (0.983; 1.052), p>.05 (Traffic/pedestrian safety **0**)  *Main effects with TotalPA(hrs/wk—prevalence):* 2. Change in traffic safety: OR=1.042 (0.964; 1.125), p>.05 (Traffic/pedestrian safety **0**)  *Main effects with TotalPA(hrs/wk—frequency):* 2. Change in traffic safety: Activity intensity ratio=1.013 (0.978; 1.049), p>.05 (Traffic/pedestrian safety **0**) | Table 5. |
| 37 | Netherlands Housing Survey (WoON) data Jongeneel-Grimen et al., 2014 [137] | n=6830 (2006; sub-sample), n=8994 (2009; sub-sample) (urban) 60-84 years  53-56% female 62.6-70.9% response rate Community-dwellers *Multiple municipalities,* Netherlands | Cross-sectional measured at two time-points Cluster: purposive Individuals: random Stratification: age, gender, country of birth and municipality Neighbourhood definition: postal code | Age, sex education, employment status, household income, urbanisation of municipality | *Self-report (WoON questionnaire; unvalidated):*  Total PA (hrs/wk) 🡪 Total PA *TotalPA(hrs/wk—prevalence)* | *Perceived [WoON questionnaire; unvalidated]:*  1. No fear of crime 🡪 Crime/personal safety 2. Change in no fear of crime 🡪 Crime/personal safety 3. Absence of physical disorder 🡪 Greenery and aesthetically pleasing scenery 4. Change in absence of physical disorder 🡪 Greenery and aesthetically pleasing scenery 5. Absence of social disorder 🡪 Crime/personal safety 6. Change in absence of social disorder 🡪 Crime/personal safety | None.  *Notes:* Age:  *In 2006:*  18-34 y (n≈7408), 35-59 y (n≈11,070), 60-84 y (n≈6830)  *In 2009:*  18-34 y (n≈9134), 35-59 y (n≈13,648), 60-84 y (n≈8994) | Multilevel multivariate logistic regression | ***Main effects with TotalPA(hrs/wk—prevalence):*** 1. No fear of crime—OR (95% CIs): OR=1.32 (1.16; 1.50), p≤.05 (Crime/personal safety **+**) 2. Change in no fear of crime: OR=1.02 (0.87; 1.20), p>.05 (Crime/personal safety **0**) 3. Absence of physical disorder: OR=1.11 (1.01; 1.23), p≤.05 (Greenery and aesthetically pleasing scenery **+**) 4. Change in absence of physical disorder: OR=1.02 (0.91; 1.15), p>.05 (Greenery and aesthetically pleasing scenery **0**) 5. Absence of social disorder: OR=1.26 (1.11; 1.42), p ≤ .05 (Crime/personal safety **+**) 6. Change in absence of social disorder: OR=1.06 (0.94; 1.21), p>.05 (Crime/personal safety **0**) | Table 4. |
| 38 | *No study name*  Aird et al., 2015 [118] | N=48 (mixed)  Mean age: 72 years  50% female  Response rate not reported  Community-dwellers  Queensland, Australia | Cross-sectional  Cluster: purposive  Individuals: convenience  Stratification: population density/urbanisation Neighbourhood definition: statistical local areas | None | *Objective [GPS in conjunction with travel diary; unvalidated]:*  Total walking (mins/d) 🡪 Total walking *TotalWalking(mins/d)* | *Objective [used statistical information based on population density; unvalidated]:*  1. Urbanisation 🡪 Urbanisation | None | Kruskal-Wallis test | ***Main effect with TotalWalking(mins/d)*:**  1. Urbanisation:  Inner city=26.38, City suburban=21.92, Regional city=23.46, Rural town=26.25  (Urbanisation **0**) | None |
| 39 | *No study name* Arnadottir et al., 2009 [119] | N=186 (mixed) 65+ years 48% female 78-80% response rates Community-dwellers *Multiple locations,* Iceland | Cross-sectional Cluster: purposive Individuals: random Stratification: population density and occupation (farming) Neighbourhood definition: *macro level—not defined* | Education, depression, physical function (timed up and go test) | *Self-report [PASE questionnaire; validated]:*  Total PA (score) 🡪 Total PA *TotalPA(score)* | *Objective [National Registry data; unvalidated]:*  1. Residency 🡪 Urbanisation | None | Analysis of covariance  *Note. Positively-skewed Total PASE data—log transformation undertaken* | ***Main effects with TotalPA(score):*** 1. Urbanisation: f=0.93, p=.336 (Urbanisation **0**) | Table 2. |
| 40 | *No study name* Asawachaisuwikrom 2001 [60] | N=112 (mixed) Mean age: 71 years  50% female Response rate not reported Community-dwellers Chonburi Province, Thailand | Cross-sectional Cluster: random Individuals: random Stratification: urbanisation Neighbourhood definition: 15-20mins walk from home | Gender, education, income, perceived benefits, perceived barriers, self-efficacy, family support | *Self-report [Physical Activity Questionnaire (PAQ); validated]:*  Total PA (score) 🡪 Total PA *TotalPA(score)* | *Perceived [Convenient Facilities Scale; validated]:*  1. Convenient Facilities Score: scale comprised of a list of facilities that can be utilised for PA 🡪 Recreational facilities access/availability | None | Hierarchical regression. No adjustment for village cluster | ***Main effects with TotalPA(score):*** 1. Convenient facilities: t=2.93, p=.00  (Recreational facilities access/availability **+**) | Results from Table 13 used because it is the only model to include gender and education as covariates. |
| 41 | *No study name* Baceviciene & Alisauskas, 2013 [120] | N=160 (urban) Mean age: 71 years 61% female Response rate not reported Community-dwellers Kaunas City, Lithuania | Cross-sectional Cluster: none Individuals: random Stratification: none Neighbourhood definition: participant delineation | None | *Self-report [IPAQ; validated]:* Active (30+ MVPA mins/d*4+ d/wk) 🡪 Total MVPA  *TotalMVPA(30+ mins/d*4+ d/wk; Yes/No)* | *Perceived [unnamed questionnaire; validated]:*  1. Unsuitable environment/exercise facilities are too far away 🡪 Recreational facilities access/availability | None | Chi-square test | ***Main effects with TotalMVPA(30+ mins/d*4+ d/wk; Yes/No):*** 1. Exercise facilities are too far away: Active (n=3) vs. Inactive (n=6): 3.9% vs. 7.2%, p>.05 (Recreational facilities access/availability **0**) | Table 2. |
| 42 | *No study name* Bocker et al., 2016 [123] | N=147 (mixed) 65+ years 63% female Response rate not reported Dwelling not reported Rotterdam, Netherlands | Cross-sectional Cluster: none Individuals: random Stratification: urbanisation Neighbourhood definition: 300m buffer | Age, sex, education, ethnicity, household income, household size, # of cars, bicycle availability, public transport card owner, obesity, disability, trip distance, trip motive, travel company, weekend day, daily air temperature, daily precipitation level, daily wind speed, snow cover | *Self-report [travel diary; unvalidated]:*  Walking (trips/d) 🡪 Total walking *TotalWalking(trips/d)* | *Objective [GIS; unvalidated]:*  1. Address density 🡪 Residential density 2. Building diversity 🡪 Land-use mix 3. Surface % green space 🡪 Access to/availability of parks/public space | None | Zero-inflated negative binomial regression, with robust standard errors to adjust for within-cluster correlation | ***Main effects with TotalWalking(trips/d):*** 1. Address density: b=-0.003, z=-0.03, p>.05 (Residential density **0**) 2. Building diversity: b=1.749, z=1.79, p<.10 (Land-use mix—destination diversity **0**) 3. Surface % green space: b=0.004, z=0.33, p>.05 (Parks/public open space access/availability **0**) | Table 3. |
| 43 | *No study name* Carvalho Sampaio et al., 2012 [19] | N=95 (mixed) Mean age: 73.7 years 100% female Response rate not reported Community-dwellers Kyoto, Japan | Cross-sectional Cluster: none Individuals: convenience Stratification: urbanisation Neighbourhood definition: rural (<9000/15.2km^2^), urban (Kyoto City—not defined) | None | *Objective [Yamax Pedometer; validated]:*  Total steps 🡪 Total walking *TotalWalking(steps/d)  Self-report [unnamed questionnaire; unvalidated]:*  Total PA (no; almost everyday; 2-3/wk; 1-2/month) 🡪 Total PA *TotalPA(frequency)* | *Objective [classification following Bibby & Shephard, 2005; unvalidated]:*  1. Urbanisation 🡪 Urbanisation | Urbanisation: Rural (n=54), Urban (n=41) | Independent t-test, Mann-Whitney U test | ***Main effects with TotalWalking(steps/d):*** 1. Urbanisation: Urban: 5791 (3992-7634) vs. Rural: 6734 (5447-7794), p=.07 (Urbanisation **0*0.568; 0*0.432**)  ***Main effects with TotalPA(frequency):*** 1. Urbanisation: *Urban:* No: 17.9% vs. Almost everyday: 20.5%, 2-3/wk: 46.2%, 1-2/month: 15.4% vs. *Rural:* No: 35.4% vs. Almost everyday: 6.3%, 2-3/wk: 52.1%, 1-2/month: 6.3%, p=.05 (Urbanisation **0*0.568; 0*0.432**) | Table 3. |
| 44 | *No study name* Chad et al., 2005 [125] | N=658 (urban) Mean age: 77 years 80% female 73% response rate (community level) Community-dwellers *Mid-sized city,* Canada | Cross-sectional Cluster: none Individuals: convenience Stratification: none Neighbourhood definition: participant delineation | None | *Self-report [Physical Activity Scale for the Elderly (PASE) questionnaire; validated]:*  Total PA (score) 🡪 Total PA *TotalPA(score)* | *Perceived [unnamed questionnaire; reliable]:*  1. Hills 🡪 No physical barriers to walking 2. Enjoyable scenery 🡪 Greenery and aesthetically pleasing scenery 3. Sidewalks 🡪 Walk-friendly infrastructure 4. Biking lanes or trails 🡪 Cycle-friendly infrastructure 5. Walking/hiking trails 🡪 Parks/public open space access/availability 6. Water fountains 🡪 Greenery and aesthetically pleasing scenery 7. Benches to sit on 🡪 Walk-friendly infrastructure 8. Street lights 🡪 Crime/personal safety 9. Golf course 🡪 Recreational facilities access/availability  10. Public park 🡪 Parks/public open space access/availability 11. Skating rink 🡪 Recreational facilities access/availability  12. Swimming pool 🡪 Recreational facilities access/availability  13. Tennis courts 🡪 Recreational facilities access/availability  14. Dance studio 🡪 Recreational facilities access/availability  15. Public recreation centre 🡪 Recreational facilities access/availability  16. Heavy traffic 🡪 Traffic/pedestrian safety 17. Dogs that are unattended 🡪 Crime/personal safety  18. Frequently see active people 🡪 Crime/personal safety 19. High crime 🡪 Crime/personal safety 20. Type of neighbourhood 🡪 Land-use mix—destination diversity | Age:  65-79 y (n=324), 80+ y (n=311) | Independent t-test/one-way ANOVA (when assumptions met) or Mann-Whitney U test/Kruskal-Wallis test (when assumptions not met). No formal testing of moderating effects. | ***Main and moderated effects with TotalPA(score):***  1. Hills—Absent vs. Present (applies to all exposures): *65-79 years (n=324):* 127.6±66.9 vs. 134.1±62.2, p>.05 *80+ years (n=311):*  81.2±49.5 vs. 89.3±50.3, p>.05 (No physical barriers to walking **0*0.51; 0*0.49**) 2. Enjoyable scenery: *65-79 years (n=324):* 124.6±64.8 vs. 130.8±65.6, p>.05 *80+ years (n=310):* 77.5±53.5 vs. 84.8±48.5, p>.05  (Greenery and aesthetically pleasing scenery **0*0.51; 0*0.49**) 3. Sidewalks: *65-79 years (n=324):* 122.5±59.2 vs. 130.2±65.9, p>.05 *80+ years (n=311):* 91.2±70.8 vs. 83.0±47.2, p>.05 (Walk-friendly infrastructure **0*0.51; 0*0.49**) 4. Biking lanes or trails: *65-79 years (n=324):* 124.7±62.6 vs. 137.7±69.2, p>.05 *80+ years (n=311):* 82.4±50.6 vs. 87.7±47.6, p>.05 (Cycle-friendly infrastructure **0*0.51; 0*0.49**) 5. Walking/hiking trails: *65-79 years (n=323):* 122.2±59.2 vs. 130.2±65.9, p>.05 (Parks/public open space access/availability **0*0.51**) *80+ years (n=311):* 74.8±46.1 vs. 95.3±52.0, p<.05 (Parks/public open space access/availability **+*0.49**) 6. Water fountains: *65-79 years (n=324):* 129.4±64.2 vs. 132.0±74.8, p>.05 *80+ years (n=310):* 85.9±50.3 vs. 70.3±44.8, p>.05 (Greenery and aesthetically pleasing scenery **0*0.51; 0*0.49**) 7. Benches to sit on: *65-79 years (n=324):* 150.3±74.2 vs. 122.4±60.5, p<.05 (Walk-friendly infrastructure **-*0.51**) *80+ years (n=311):* 82.5±52.6 vs. 84.1±49.0, p>.05 (Walk-friendly infrastructure **0*0.49**) 8. Street lights: *65-79 years (n=324):* 116.8±58.6 vs. 131.5±66.2, p>.05 *80+ years (n=311):* 82.3±53.6 vs. 84.1±49.0, p>.05 (Street lighting **0*0.51; 0*0.49**) 9. Golf course: *65-79 years (n=324):* 126.4±126.4 vs. 144.5±144.5, p>.05 *80+ years (n=311):* 82.1±49.1 vs. 101.1±54.5, p>.05 (Recreational facilities access/availability **0*0.51; 0*0.49**) 10. Public park: *65-79 years (n=324):* 112.6±54.1 vs. 134.3±67.5, p<.05 *80+ years (n=311):* 72.6±49.2 vs. 90.7±49.0, p<.05  (Parks/public open space access/availability **+*0.51; +*0.49**) 11. Skating rink: *65-79 years (n=324):* 125.2±63.3 vs. 147.4±70.6, p<.05 (Recreational facilities access/availability **+*0.51**) *80+ years (n=310):* 81.2±47.3 vs. 99.8±60.7, p>.05 (Recreational facilities access/availability **0*0.49**) 12. Swimming pool: *65-79 years (n=324):* 132.1±67.1 vs. 125.9±62.6, p>.05 (Recreational facilities access/availability **0*0.51**) *80+ years (n=310):* 78.8±49.0 vs. 96.1±50.1, p<.05 (Recreational facilities access/availability **+*0.49**) 13. Tennis courts: *65-79 years (n=324):* 126.1±58.5 vs. 139.8±81.0, p>.05 (Recreational facilities access/availability **0*0.51**) *80+ years (n=311):* 80.4±48.0 vs. 98.0±54.8, p<.05 (Recreational facilities access/availability **+*0.49**) 14. Dance studio: *65-79 years (n=324):* 129.8±65.7 vs. 129.4±63.5, p>.05  (Recreational facilities access/availability **0*0.51**) *80+ years (n=311):* 80.8±48.4 vs. 111.2±55.0, p<.05  (Recreational facilities access/availability **+*0.49**) 15. Public recreation centre: *65-79 years (n=324):* 129.3±64.7 vs. 130.5±67.0, p>.05 *80+ years (n=311):* 81.7±51.2 vs. 89.1±46.1, p>.05  (Recreation facilities access/availability **0*0.51**; **0*0.49**) 16. Heavy traffic: *65-79 years (n=324):* 129.9±70.2 vs. 129.6±61.3, p>.05 *80+ years (n=311):* 80.1±48.8 vs. 86.9±50.5, p>.05  (Traffic/pedestrian safety **0*0.51; 0*0.49**) 17. Dogs that are unattended: *65-79 years (n=324):* 128.3±65.3 vs. 136.5±66.3, p>.05  (Crime/personal safety **0*0.51**) *80+ years (n=310):* 79.8±45.4 vs. 104.9±65.2, p<.05  (Crime/personal safety **-*0.49**) 18. Frequently see active people: *65-79 years (n=324):* 117.8±58.7 vs. 132.6±66.7, p>.05  (Crime/personal safety **0*0.51**) *80+ years (n=311):* 71.8±45.7 vs. 87.6±50.5, p<.05  (Crime/personal safety **+*0.49**) 19. High crime: *65-79 years (n=324):* 130.7±65.7 vs. 122.5±63.9, p>.05 *80+ years (n=311):* 83.6±49.4 vs. 85.2±53.2, p>.05 (Crime/personal safety **0*0.51; 0*0.49**) 20. Type of neighbourhood: *65-79 years:* Residential (n=208): 137.2±67.7 vs. commercial (n=3): 151.9±57.4 vs. mixed (n=107): 116.5±91.5, p<.05 (Land-use mix—destination diversity **+*0.51**) *80+ years:* Residential (n=208): 87.4±51.4 vs. commercial (n=3): 61.6±43.6 vs. mixed (n=107): 75.3±41.3, p>.05 (Land-use mix—destination diversity **0*0.49**) | Moderating effects. Table 6. |
| 45 | *No study name* Chaudhury et al., 2016 [126] | N=434 (urban) 60+ years (no mean age reported) 64% female 6% response rate Dwelling not reported Portland, USA, and Vancouver, Canada | Cross-sectional Cluster: purposive Individuals: random Stratification: population density (proxy for walkability *in article*) and median household income Neighbourhood definition: 15-20mins walk from home | Age, education, marital status, household income, self-rated health, physical functioning limitations, membership in a sports group or recreational organisation, walking with a neighbour | *Self-report [unnamed questionnaire; unvalidated]:*  Total PA (5+ hr/wk; Yes/No) 🡪 Total PA *TotalPA(5+ hr/wk; Yes/No)* | *Perceived [NEWS questionnaire; validated]:*   1. Neighbourhood walkability 🡪 Walkability 2. Neighbourhood amenities and accessibility 🡪 Destinations/services (overall/unspecific) access/availability | None | Multivariate logistic regression | ***Main effects with TotalPA(5+ hr/wk; Yes/No):*** 1. Neighbourhood walkability: b=not reported, p>.05 (Walkability **0**) 2. Neighbourhood amenities and accessibility: b=not reported, p>.05 (Destinations/services (overall/unspecific) access/availability **0**) | Table 4. |
| 46 | *No study name* Chen et al., 2013 [20] | n=1701 (sub-sample; urban) Mean age: 70 years 50% female Response rate not reported Community-dwellers Tsuruoka City, Japan | Cross-sectional Cluster: purposive Individuals: random Stratification: age and sex Neighbourhood definition: 10mins walk from home | Age, automobile commuting | *Self-report [unnamed questionnaire; validated]:*  Daily walking (mins/d) 🡪 Total walking *TotalWalking(60+ mins/d; Yes/No)* | *Perceived [NEWS questionnaire—Japanese version; validated]:*  1. Density of dwelling 🡪 Residential density 2. Proximity of services facilities 🡪 Destinations/services (overall/unspecific) access/availability 3. No. of service facilities within 10 mins 🡪 Destinations/services (overall/unspecific) access/availability  4. Street connectivity 🡪 Street connectivity 5. Places for walking 🡪 Destinations/services (overall/unspecific) access/availability 6. Good view 🡪 Greenery and aesthetically pleasing scenery | Sex:  Male (n=846), Female (n=855)  Age: 20-39 y (n=633), 40-59 y (n=1472), 60-79 y (n=1701)  Employment status: Employed (n=604), Unemployed (n=1097) | Multivariate logistic analysis | ***Main effects with TotalWalking(60+ mins/d; Yes/No):*** 1. Density of dwelling (Ref: Low)—OR (95% CIs):  *Males + employed (60-79 y):* High OR=1.27 (0.52; 3.09), p>.05 *Males + unemployed:* High OR=0.89 (0.35; 2.25), p>.05 *Females + employed (60-79 y):* High OR=0.85 (0.26; 2.81), p>.05 *Females + unemployed:* High OR=0.99 (0.46; 2.17), p>.05 (Residential density **0*0.244; 0*0.253; 0*0.133; 0*0.370**) 2. Proximity to service facilities (Ref: Low): *Males + employed (60-79 y):* High OR=0.69 (0.34; 1.39), p>.05 *Males + unemployed:* High OR=1.63 (0.69; 3.85), p>.05  *Females + employed (60-79 y):* High OR=1.07 (0.41; 2.80), p>.05 *Females + unemployed:* High OR=0.87 (0.43; 1.75), p>.05 (Destinations/services (overall/unspecific) access/availability **0*0.244; 0*0.253; 0*0.133; 0*0.370**) 3. Number of service facilities within 10 mins (Ref: Low): *Males + employed (60-79 y):* High OR=0.66 (0.33; 1.36), p>.05 *Males + unemployed:* High OR=0.93 (0.39; 2.24), p>.05 *Females + employed (60-79 y):* High OR=0.98 (0.38; 2.53), p>.05 *Females + unemployed:* High OR=1.06 (0.53; 2.13), p>.05 (Destinations/services (overall/unspecific) access/availability **0*0.244; 0*0.253; 0*0.133; 0*0.370**) 4. Street connectivity (Ref: Low): *Males + employed (60-79 y):* High OR=0.73 (0.36; 1.50), p>.05 *Males + unemployed:* High OR=2.22 (0.93; 5.32), p>.05 *Females + employed (60-79 y):* High OR=1.50 (0.57; 3.95), p>.05 *Females + unemployed:* High OR=0.74 (0.35; 1.54), p>.05 (Street connectivity **0*0.244; 0*0.253; 0*0.133; 0*0.370**) 5. Places for walking (Ref: Low): *Males + employed (60-79 y):* High OR=0.90 (0.44; 1.85), p>.05 *Males + unemployed:* High OR=0.47 (0.20; 1.10), p>.05 *Females + employed (60-79 y):* High OR=0.88 (0.33; 2.31), p>.05 *Females + unemployed:* High OR=1.15 (0.56; 2.35), p>.05 (Destinations/services (overall/unspecific) access/availability **0*0.244; 0*0.253; 0*0.133; 0*0.370**) 6. Good view (Ref: Low): *Males + employed (60-79 y):* High OR=0.78 (0.37; 1.62), p>.05 *Males + unemployed:* High OR=1.01 (0.44; 2.30), p>.05 *Females + employed (60-79 y):* High OR=0.92 (0.35; 2.40), p>.05 *Females + unemployed:* High OR=0.92 (0.35; 2.40), p>.05 (Greenery and aesthetically pleasing scenery **0*0.244; 0*0.253; 0*0.133; 0*0.370**) | Table 2. |
| 47 | *No study name*  de Melo et al., 2010 [127] | N=60 (urban) 65+ years 75% female Response rate not reported Community-dwellers *Midsized city,* Canada | Cross-sectional Cluster: convenience Individuals: convenience Stratification: none Neighbourhood definition: 15-20mins walk from home | Age, income, physical function | *Objective [StepsCount—01 pedometer; reliable]:*  Steps/d 🡪 Total walking *TotalWalking(steps/d)* | *Perceived [NEWS-A questionnaire; validated]:*  1. Access to services 🡪 Destinations/services (overall/unspecific) access/availability 2. Street connectivity 🡪 Street connectivity 3. Infrastructure for walking and cycling 🡪 Cycle/walk-friendly infrastructure 4. Aesthetics 🡪 Greenery and aesthetically pleasing scenery | None | Hierarchical negative binomial regression due to skewed and overdispersion. Pearson correlation | ***Main effects with TotalWalking(steps/d):*** 1. Access to services: RR=1.05 (95% CI=0.99; 1.13), p=.07  (Destinations/services (overall/unspecific) access/availability **0**) 2. Street connectivity: r=not reported, p>.05 (Street connectivity **0**) 3. Infrastructure for walking and cycling: r=not reported, p>.05 (Cycling/Walk-friendly infrastructure **0**) 4. Aesthetics: r=not reported, p>.05 (Greenery and aesthetically pleasing scenery **0**) | #1: Table 4. #2-5: Pearson correlations. |
| 48 | *No study name*  Gallagher et al., 2012 [131] | N=326 (urban) Mean age: 76 years;  67% female 81.5% response rate Community-dwellers Michigan, USA | Cross-sectional Cluster: none Individuals: convenience Stratification: none Neighbourhood definition: 15min/0.5mile walk from home | Age, sex, education, race, outcome expectations, self-efficacy | *Self-report [Neighbourhood Physical Activity Questionnaire (NPAQ); reliable]:*  Neighbourhood walking (mins/wk) 🡪 Total walking  *TotalWalking(mins/wk)* | *Perceived [NEWS questionnaire; validated]:*  1. Neighbourhood density 🡪 Residential density 2. Neighbourhood destinations 🡪 Destinations/services (overall/unspecific) access/availability | Mobility impairment: Mobility limited (n=163),  Non-mobility limited (n=163) | Pearson correlation; multiple linear regression | ***Main effects with TotalWalking(mins/wk):*** 1. Neighbourhood density: *All participants:* r=0.106, p>.05  (Residential density **0*1**)  ***Moderated effects with TotalWalking(mins/wk):*** 2. Neighbourhood destinations: *Mobility-limited participants:* b=0.318, p<.001 (Destinations/services (overall/unspecific) access/availability **+*0.5**) *Non-mobility-limited participants:* b=0.318, p>.05 (Destinations/services (overall/unspecific) access/availability **0*0.5**) | Moderating effects. Table 2 and 4. |
| 49 | *No study name* Gomez et al., 2010 [24] | N=1966 (urban) Mean age: 71 years 63% females 67.8% response rate Community-dwellers Bogotá, Colombia | Cross-sectional Cluster: purposive Individuals: random Stratification: area-level SES Neighbourhood definition: participant delineation (perceived); 500m buffer (objective) | Age, sex, education, limitation to engage in PA, slope, SES | *Self-report [IPAQ—modified version; unvalidated]:*  Total walking (60+ mins/wk; Yes/No) 🡪 Total walking *TotalWalking(60+ mins/wk; Yes/No)* Total walking (150+ mins/wk; Yes/No) 🡪 Total walking *TotalWalking(150+ mins/wk; Yes/No)* | *Objective [GIS; unvalidated]:*  1. Slope 🡪 No physical barriers to walking 2. Connectivity index 🡪 Street connectivity 3. Public park density 🡪 Parks/public open space access/availability 4. TransMilenio stations 🡪 Public transport access/availability  *Perceived [unnamed questionnaire; unvalidated]:* 5. Traffic safety 🡪 Traffic/pedestrian safety 6. Quality and maintenance of sidewalks 🡪 Pavement/footpath quality | *Note. Cross-level interactions were explored but none found.* | Multilevel models with random intercept to adjusting for clustering. | ***Main effects with TotalWalking(60+ mins/wk; Yes/No):*** Objective environment: 1. Slope (Ref:<5%)—OR (95% CIs): 5+% OR=0.61 (0.38; 0.97), p=.038 (No physical barriers to walking **+**) 2. Connectivity index (Ref: 1.46-1.74): 1.75-1.80 OR=0.74 (0.50; 1.10), p=0.143 1.81-1.99 OR=0.64 (0.44; 0.93), p=.021  (Street connectivity **-)** 3. Public park density (Ref: 0.01-4.14): 4.53-7.98 OR=1.42 (1.02; 1.98), p=.039 8.11-35.21 OR=1.06 (0.74; 1.53), p=.726 (Parks/public open space access/availability **+**) 4. TransMilenio stations (Ref: 0): 1+ OR=0.75 (0.52; 1.08), p=.127 (Public transport access/availability **0**)  Perceived environment: 5. Traffic safety (Ref: Very unsafe, unsafe, neither): Very safe, safe OR=1.50 (1.11; 2.03), p=.007 (Traffic/pedestrian safety **+**) 6. Quality and maintenance (Ref: Very dissatisfied, dissatisfied, neither): Satisfied, very satisfied OR=0.82 (0.61; 1.12), p=.225  (Pavement/footpath quality **0**)  ***Main effects with TotalWalking(150+ mins/wk; Yes/No):*** Objective environment: 1. Slope (Ref:<5%): 5+% OR=0.80 (0.55; 1.16), p=.254 (No physical barriers to walking **0**) 2. Connectivity index (Ref: 1.46-1.74): 1.75-1.80 OR=0.79 (0.58; 1.09), p=0.163 1.81-1.99 OR=0.90 (0.67; 1.23), p=.540 (Street connectivity **0**) 3. Public park density (Ref: 0.01-4.14): 4.53-7.98 OR=1.08 (0.81; 1.43), p=.574 8.11-35.21 OR=0.95 (0.70; 1.29), p=.757 (Parks/public open space access/availability **0**) 4. TransMilenio stations (Ref: 0): 1+ OR=0.78 (0.59; 1.04), p=.101 (Public transport access/availability **0**)  Perceived environment: 5. Traffic safety (Ref: Very unsafe, unsafe, neither): Very safe, safe OR=1.19 (0.97; 1.47), p=.088 (Traffic/pedestrian safety **0**) 6. Quality and maintenance (Ref: Very dissatisfied, dissatisfied, neither): Satisfied, very satisfied OR=0.95 (0.76; 1.19), p=.712 (Pavement/footpath quality **0**) | Table 2. |
| 50 | *No study name* Grant-Savela et al., 2009 [67] | N=197 (rural) Mean age: 72 years 55% female 50.8% response rate Community-dwellers *Midwest,* USA | Cross-sectional Cluster: none Individuals: convenience Stratification: none Neighbourhood definition: Naturally occurring retirement community (NORC) itself (variable/not fixed) | None | *Self-report [adapted version of PASE; unvalidated]:*  Total PASE score 🡪 Total PA *TotalPA(score)*  Walking frequency (d/wk) 🡪  Total walking  *TotalWalking(d/wk)*  Walking duration (hr/d) 🡪 Total walking *TotalWalking(hr/d)* | *Perceived [unnamed questionnaire; unvalidated]:*  1. Convenience of destinations from participant’s home 🡪 Destinations/services (overall/unspecific) access/availability 2. Location of walking 🡪 Land-use mix—destination diversity | None | Spearman’s Rank-Order Correlation | ***Main effects with TotalPA(score):*** 1. Convenience of destinations: r=0.12, p>.05 (Destinations/services (overall/unspecific) access/availability **0**) 2. Location of walking: r=0.09, p>.05 (Land-use mix—destination diversity **0**)  *Main effects with TotalWalking(d/wk):* 1. Convenience of destinations: r=0.08, p>.05 (Destinations/services (overall/unspecific) access/availability **0**) 2. Location of walking: r=0.12, p>.05 (Land-use mix—destination diversity **0**)  ***Main effects with TotalWalking(hr/d):*** 1. Convenience of destinations: r=0.18, p<.05 (Destinations/services (overall/unspecific) access/availability **+**) 2. Location of walking: r=0.17, p<.05 (Land-use mix—destination diversity **+**) | Table 3. |
| 51 | *No study name* Inoue et al.,2011 [22] | N=1921 (mixed) 65+ years 49% female  72.8% response rate Community-dwellers Bunkyo ward in Tokyo, Fuchu in Tokyo, and Oyama in Shizuoka prefecture, Japan | Cross-sectional Cluster: purposive Individuals: random Stratification: age, sex, urbanisation and neighbourhood Neighbourhood definition: cho-cho (smallest administrative unit for area in Japan), 10-15mins walk from residence | Age, education, city of residence, employment status, BMI, self-rated health | *Self-report [modified version of a previously unnamed questionnaire; unvalidated]:*  Total neighbourhood walking (150+ mins/wk) 🡪 Total walking *TotalWalking(150+ min/wk; Yes/No)* | *Perceived [IPAQ Environmental Module—modified version; validated]:*  1. Residential density 🡪 Residential density 2. Access to shops 🡪 Shops/commercial access/availability 3. Public transport 🡪 Public transport access/availability 4. Sidewalks 🡪 Walk-friendly infrastructure 5. Bicycle lanes 🡪 Cycle-friendly infrastructure 6. Access to exercise facilities 🡪 Recreational facilities access/availability  7. Crime safety 🡪 Crime/personal safety 8. Traffic safety 🡪 Traffic/pedestrian safety 9. Social environment (seeing people being active) 🡪 Crime/personal safety 10. Aesthetics 🡪 Greenery and aesthetically pleasing scenery | Sex:  Male (n=846), Female (n=855) | Multilevel logistic regression accounting for clustering. | ***Main and moderated effects with TotalWalking(150+ mins/wk; Yes/No):*** 1. Residential density—OR (95% CIs): *Males:* High (n=210) vs. Low (n=320):  OR=0.93 (0.69; 1.26), p=.650 *Females:* High (n=217) vs. Low (n=286):  OR=1.09 (0.80; 1.49), p=.578 (Residential density **0*0.5; 0*0.5**) 2. Access to shops: *Males:* Good (n=385) vs. Poor (n=142):  OR=1.02 (0.75; 1.39), p=.913 (Shops/commercial access/availability **0*0.5**) *Females:* Good (n=362) vs. Poor (n=139): OR=1.44 (1.07; 1.94), p=.017 (Shops/commercial access/availability **+*0.5**) 3. Public transport: *Males:* Good (n=482) vs. Poor (n=45):  OR=0.94 (0.59; 1.49), p=.799 *Females:* Good (n=473) vs. Poor (n=32):  OR=1.08 (0.65; 1.80), p=.776 (Public transport access/availability **0*0.5; 0*0.5**) 4. Sidewalks: *Males:* Good (n=438) vs. Poor (n=89): OR=1.22 (0.87; 1.72), p=.255 *Females:* Good (n=430) vs. Poor (n=74): OR=1.30 (0.91; 1.85), p=.152 (Walk-friendly infrastructure **0*0.5; 0*0.5**) 5. Bicycle lanes: *Males:* Good (n=200) vs. Poor (n=327):  OR=0.98 (0.74; 1.31), p=.904 *Females:* Good (n=203) vs. Poor (n=301):  OR=1.18 (0.88; 1.57), p=.271 (Cycle-friendly infrastructure **0*0.5; 0*0.5**) 6. Access to exercise facilities: *Males:* Good (n=354) vs. Poor (n=174): OR=1.19 (0.90; 1.59), p=.221 *Females:* Good (n=346) vs. Poor (n=158): OR=1.31 (0.98; 1.76), p=.221 (Recreational facilities access/availability **0*0.5; 0*0.5**) 7. Crime safety: *Males:* Good (n=388) vs. Poor (n=141):  OR=0.78 (0.57; 1.05), p=.106 *Females:* Good (n=329) vs. Poor (n=174):  OR=1.16 (0.87; 1.53), p=.308 (Crime/personal safety **0*0.5; 0*0.5**) 8. Traffic safety: *Males:* Good (n=372) vs. Poor (n=156):  OR=1.08 (0.81; 1.43), p=.606 *Females:* Good (n=343) vs. Poor (n=162):  OR=1.24 (0.94; 1.65), p=.130 (Traffic/pedestrian safety **0*0.5; 0*0.5**) 9. Social environment: *Males:* Good (n=395) vs. Poor (n=134):  OR=1.57 (1.18; 2.09), p=.002 (Crime/personal safety **+*0.5**) *Females:* Good (n=357) vs. Poor (n=145):  OR=1.24 (0.93; 1.66), p=.145 (Crime/personal safety **0*0.5**) 10. Aesthetics: *Males:* Good (n=376) vs. Poor (n=153):  OR=1.56 (1.18; 2.07), p=.002 *Females:* Good (n=367) vs. Poor (n=137):  OR=1.38 (1.03; 1.85), p=.030 (Greenery and aesthetically pleasing scenery **+*0.5; +*0.5**) | Moderating effects. Table 2. |
| 52 | *No study name* King et al., 2003 [141] | N=149 (not reported) Mean age: 74 years 100% female 79-82% response rate Community-dwellers Pennsylvania, USA | Cross-sectional Cluster: purposive Individuals: convenience Stratification: sex Neighbourhood definition: 20mins walk from home | None | *Self-report [Paffenbarger Activity Questionnaire; validated]:*  Total PA (kcal/wk) 🡪 Total PA *TotalPA(median kcal/wk)*  *Objective [Yamax Digiwalker pedometer; validated]:*  Steps/d (kcal/wk) 🡪 Total walking *TotalWalking(median kcal/wk); TotalSteps/d (median)* | *Perceived [unnamed questionnaire; unvalidated]:*  1. Biking or walking trail 🡪 Parks/public open space access/availability 2. Bus stop 🡪 Public transport access/availability 3. Café or coffee shop 🡪 Food outlets access/availability 4. Church, synagogue, or religious institution 🡪 Religious institution access/availability 5. Community centre 🡪 Social recreational facilities access/availability 6. Convenience, deli, or grocery store 🡪 Shops/commercial access/availability 7. Department, discount, or hardware 🡪 Shops/commercial access/availability 8. Library 🡪 Education facilities access/availability 9. Park 🡪 Parks/public open space access/availability 10. Post office 🡪 Shops/commercial access/availability 11. Restaurant, bar, or pub 🡪 Food outlets access/availability 12. No. of destinations 🡪 Destinations/services (overall/unspecific) access/availability 13. Walkability 🡪 Walkability | Walkability*no. of destinations within walking distance  Walkability: Poor (n=16), Fair (n=24), Good (n=62), Excellent (n=46) | Wilcoxon rank sum test; Jonckheere-Terpstra trend test | *Significant moderating effects for: Walkability*no. of destinations within walking distance, p=.0005* ***Main and moderated effects with TotalPA(median kcal/wk; 25^th^; 75^th^ %tile):*** 1. Biking or walking trail: Yes (n=38)=1517 (785; 2285) vs. no (n=103)=1246 (554; 2354), p=.2636 (Parks/public open space access/availability **0**) 2. Bus stop: Yes (n=116)=1246 (640; 2389) vs. no (n=27)=1344 (646; 2492), p>.05 (Public transport access/availability **0**) 3. Café or coffee shop: Yes (n=82)=1281 (554; 2492) vs. no (n=59)=1246 (658; 2215), p>.05 (Food outlets access/availability **0**) 4. Church, synagogue, or religious institution: Yes (n=68)=1240 (692; 2279) vs. no (n=74)=1341 (623; 2469), p>.05 (Religious institution access/availability **0**) 5. Community centre: Yes (n=46)=1454 (692; 2492) vs. no (n=93)=1235 (623; 2327), p>.05 (Social recreational facilities access/availability **0**) 6. Convenience, deli, grocery store: Yes (n=89)=1442 (692; 2354) vs. no (n=54)=1050 (623; 2469), p>.05 (Shops/commercial access/availability **0**) 7. Department, discount, or hardware store: Yes (n=30)=1794 (739; 3000) vs. no (n=111)=1168 (623; 2215), p>.05 (Shops/commercial access/availability **0**) 8. Library: Yes (n=45)=1339 (785; 2539) vs. no (n=97)=1062 (623; 2273), p>.05 (Education facilities access/availability **0**) 9. Park: Yes (n=69)=1344 (692; 2539) vs. no (n=75)=1235 (531; 2273), p>.05 (Parks/public open space access/availability **0**) 10. Post office: Yes (n=50)=1292 (692; 2723) vs. no (n=92)=1246 (635; 2244), p>.05 (Shops/commercial access/availability **0**) 11. Restaurant, bar, or pub: Yes (n=78)=1390 (623; 2469) vs. no (n=64)=1246 (652; 2215), p>.05 (Food outlets access/availability **0**) 12. No. of destinations: 0 (n=14) 52 (0; 415) vs. 1 (n=15) 139 (0; 831) vs. 2-11 (n=120) 623 (196; 315), p=.0005 (Destinations/services (overall/unspecific) access/availability **+*0.73; 0*0.27**) 13. Walkability: Poor (n=16) 612 (65; 1015) vs. fair (n=24) 1396 (640; 2308) vs. good (n=62) 1246 (415; 2327) vs. excellent (n=46) 1399 (854; 2908), p=.0016 (Walkability **+**)  ***Main effects with TotalWalking(median kcal/wk; 25^th^; 75^th^ %tile):*** 1. Biking or walking trail: Yes (n=38)=692 (215; 1246) vs. no (n=103)=519 (104; 1246), p>.05 (Parks/public open space access/availability **0**) 2. Bus stop: Yes (n=116)=623 (139; 1240) vs. no (n=27)=623 (138; 1246), p>.05 (Public transport access/availability **0**) 3. Café or coffee shop: Yes (n=82)=623 (116; 1246) vs. no (n=59)=519 (138; 1246), p>.05 (Food outlets access/availability **0**) 4. Church, synagogue, or religious institution: Yes (n=68)=669 (199; 1240) vs. no (n=74)=512 (138; 1246), p>.05 (Religious institution access/availability **0**) 5. Community centre: Yes (n=46)=623 (138; 1168) vs. no (n=93)=554 (138; 2356), p>.05 (Social recreational facilities access/availability **0**) 6. Convenience, deli, grocery store: Yes (n=89)=692 (215; 1246) vs. no (n=54)=467 (69; 865), p>.05 (Shops/commercial access/availability **0**) 7. Department, discount, or hardware store: Yes (n=30)=658 (277; 1454) vs. no (n=111)=623 (138; 1246), p>.05 (Shops/commercial access/availability **0**) 8. Library: Yes (n=45)=880 (277; 1246) vs. no (n=97)=623 (138; 1246), p>.05 (Education facilities access/availability **0**) 9. Park: Yes (n=69)=692 (173; 1454) vs. no (n=75)=519 (139; 1235), p>.05 (Parks/public open space access/availability **0**) 10. Post office: Yes (n=50)=623 (242; 1246) vs. no (n=92)=614 (138; 1246), p>.05 (Shops/commercial access/availability **0**) 11. Restaurant, bar, or pub: Yes (n=78)=623 (92; 1050) vs. no (n=64)=623 (179; 1246), p>.05 (Food outlets access/availability **0**) 12. No. of destinations: 0 (n=14) 52 (0; 415) vs. 1 (n=15) 139 (0; 831) vs. 2-11 (n=120) 623 (196; 315), p=.0005 (Destinations/services (overall/unspecific) access/availability **+*0.73; 0*0.27**) 13. Walkability: Poor (n=16) 139 (0; 519) vs. fair (n=24) 364 (0; 1000) vs. good (n=62) 623 (176; 1246) vs. excellent (n=46) 692 (415; 1454), p=.0077 (Walkability **+**)  ***Main effects with TotalSteps/d(median; 25^th^; 75^th^ %tile):*** 1. Biking or walking trail: Yes (n=38)=6797 (515; 8331) vs. no (n=103)=4908 (3060; 6728), p=.0018 (Parks/public open space access/availability **+*0.270; 0*0.730**) 2. Bus stop: Yes (n=116)=5494 (3436; 7265) vs. no (n=27)=5105 (3610; 7786), p>.05 (Public transport access/availability **0**) 3. Café or coffee shop: Yes (n=82)=5657 (4021; 7588) vs. no (n=59)=5105 (2858; 7319), p>.05 (Food outlets access/availability **0**) 4. Church, synagogue, or religious institution: Yes (n=68)=5134 (3724; 6921) vs. no (n=74)=5695 (3244; 7747), p>.05 (Religious institution access/availability **0**) 5. Community centre: Yes (n=46)=5148 (3449; 7660) vs. no (n=93)=5694 (3677, 7319), p>.05 (Social recreational facilities access/availability **0**) 6. Convenience, deli, grocery store: Yes (n=89)=5732 (3859; 7212) vs. no (n=54)=5084 (2865; 7576), p>.05 (Shops/commercial access/availability **0**) 7. Department, discount, or hardware store: Yes (n=30)=6808 (5871; 8420) vs. no (n=111)=5015 (3060; 7021), p=.0022 (Shops/commercial access/availability **+*0.213; 0*0.787**) 8. Library: Yes (n=45)=5908 (4230; 8150) vs. no (n=97)=5116 (3281; 6889), p>.05 (Education facilities access/availability **0**) 9. Park: Yes (n=69)=6075 (4594; 8150) vs. no (n=75)=4802 (2908; 6305), p=.0044 (Parks/public open space access/availability **+*0.479; 0*0.521**) 10. Post office: Yes (n=50)=5899 (3755; 8034) vs. no (n=92)=5132 (3225; 7051), p>.05 (Shops/commercial access/availability **0**) 11. Restaurant, bar, or pub: Yes (n=78)=5287 (3616; 7114) vs. no (n=64)=5580 (3243; 7592), p>.05 (Food outlets access/availability **0**) 12. No. of destinations: 0 (n=14) 2745 (1038; 4382) vs. 1 (n=15) 3281 (2449; 6801) vs. 2-11 (n=120) 5714 (3940; 7703), p<.0001 (Destinations/services (overall/unspecific) access/availability **+*0.73; 0*0.27**) 13. Walkability: Poor (n=16) 3376 (2449; 4961) vs. fair (n=24) 4258 (2532; 6812) vs. good (n=62) 5377 (3449; 6801) vs. excellent (n=46) 6349 (4877; 8749), p=.0008 (Walkability **+**) | Table 4, 5, and 6. |
| 53 | *No study name* Koh et al., 2015 [142] | N=168 (urban) 60-85+ years % female not reported Response rate not reported Community-dwellers Singapore | Cross-sectional Cluster: purposive Individuals: convenience Stratification: % of older adults/population density Neighbourhood definition: region | Age, gender, cycling status, exercise duration, medical conditions, assistance from caregiver, working status, fall incidences | *Self-report [unnamed questionnaire; unvalidated]:*  Neighbourhood walking (mins/wk) 🡪 Total walking *TotalWalking(mins/wk)* | *Perceived [unnamed questionnaire; unvalidated]:*  1. SecurityAM 🡪 Crime/personal safety 2. SecurityPM 🡪 Crime/personal safety 3. Detour (reach destinations without barriers) 🡪 No physical barriers to walking 4. Road crossing delay 🡪 Street connectivity 5. Directional sign (adequate amount of street signage) 🡪 Traffic/pedestrian safety 6. Comfort1 (good walkway condition) 🡪 Pavement/footpath quality 7. Comfort3 (no obstacles along the walkway) 🡪 No physical barriers to walking 8. Stairs/slope 🡪 No physical barriers to walking 9. Traffic accident risk 🡪 Traffic/pedestrian safety 10. Shops1 (shops within walking distance) 🡪 Shops/commercial access/availability 11. Shops2 (eateries within walking distance) 🡪 Food outlets access/availability 12. Scenery1 (many trees) 🡪 Greenery and aesthetically pleasing scenery 13. Scenery2 (e.g., reservoir, park, lake etc.) 🡪 Parks/public open space access/availability 14. Recreational facilities 1 (activity centre) 🡪 Social recreational facilities access/availability  15. Recreational facilities 2 (fitness corner) 🡪 Recreational facilities access/availability  16. Recreational facilities 3 (sheltered social spaces) 🡪 Social recreational facilities access/availability  17. Weather protection (e.g., covered walkways) 🡪 Walk-friendly infrastructure | None | General linear model | ***Main effects with TotalWalking(mins/wk):*** 1. Daytime security: b=not reported, p>.05 (Crime/personal safety **0**) 2. Night-time security: b=not reported, p>.05 (Crime/personal safety **0**) 3. Detour: b=not reported, p>.05 (No physical barriers to walking **0**) 4. Road crossing delay: b=-409.60 (SE=187.52), p=.0307 (Street connectivity **+**) 5. Adequate amount of street signage: b=not reported, p>.05 (Traffic/pedestrian safety **0**) 6. Comfort1 (good walkway condition): b=not reported, p>.05 (Pavement/footpath quality **0**) 7. Comfort3 (no obstacles in the way): b=not reported, p>.05 (No physical barriers to walking **0**) 8. Stairs/slope: b=not reported, p>.05 (No physical barriers to walking **0**) 9. Traffic accident risk: b=not reported, p>.05 (Traffic/pedestrian safety **0**) 10. Shops1: b=1036.49 (SE=354.43), p=.0040 (Shops/commercial access/availability **+)** 11. Shops2 (eateries): b=-1143.06 (SE=406.81), p=.0057  (Food outlets access/availability **-**) 12. Scenery1: b=not reported, p>.05 (Greenery and aesthetically pleasing scenery **0**) 13. Scenery2: b=not reported, p>.05 (Parks/public open space access/availability **0**) 14. Recreational facilities 1 (activity centre): b=not reported, p>.05 (Social recreational facilities access/availability **0**) 15. Recreational facilities 2 (fitness corner): b=not reported, p>.05 (Recreational facilities access/availability **0**) 16. Recreational facilities 3 (sheltered social space): b=331.53 (SE=179.85), p=.0675 (Social recreational facilities access/availability **0**) 17. Weather protection: b=not reported, p>.05 (Walk-friendly infrastructure **0**) | Table 5. |
| 54 | *No study name* Kolbe-Alexander et al., 2015 [25] | N=44 (urban) Mean age: 65 years 78% female Response rate not reported Community-dwellers Cape Town, South Africa | Cross-sectional Cluster: purposive Individuals: convenience Stratification: area-level SES Neighbourhood definition: participant delineation | None | *Self-report [Global Physical Activity Questionnaire; validated]:*  Total MVPA (150+ mins/wk; Yes/No) 🡪 Total MVPA *TotalMVPA(150+ mins/wk; Yes/No)  Objective [ActiGraph accelerometer; validated; Matthews MVPA cutoff point≥2020 cpm; validated]:*  Total MVPA (150+ mins/wk; Yes/No) 🡪 Total MVPA *TotalMVPAMatthews(150+ mins/wk; Yes/No)* | *Perceived [NEWS questionnaire; validated]:*  1. Residential density 🡪 Residential density 2. Land-use mix: diversity 🡪 Land-use mix—destination diversity 3. Land-use mix: access 🡪 Destinations/services (overall/unspecific) access/availability 4. Street connectivity 🡪 Street connectivity 5. Walk/cycle infrastructure 🡪 Cycle/walk-friendly infrastructure 6. Aesthetics 🡪 Greenery and aesthetically pleasing scenery 7. Safety from traffic 🡪 Traffic/pedestrian safety 8. Safety from crime 🡪 Crime/personal safety | Area-level SES:  Low SES (n=24), High SES (n=20) | Spearman’s correlation coefficient. No testing of interaction terms. No adjustment for suburb cluster | ***Main effects with self-reported TotalMVPA(150+ mins/wk; Yes/No):*** 1. Residential density: *Low SES:* *R^2^=*not reported, p>.05 *High SES: R^2^=*not reported, p>.05 (Residential density **0*0.55; 0*0.45**) 2. Land-use mix: diversity: *Low SES:* *R^2^=*not reported, p>.05 *High SES: R^2^=*not reported, p>.05 (Land-use mix—destination diversity **0*0.55; 0*0.45**) 3. Land-use mix: access: *Low SES:* *R^2^=*not reported, p>.05 *High SES: R^2^=*not reported, p>.05 (Destinations/services (overall/unspecific) access/availability **0*0.55; 0*0.45**) 4. Street connectivity: *Low SES:* *R^2^=*not reported, p>.05 *High SES: R^2^=*not reported, p>.05 (Street connectivity **0*0.55; 0*0.45**) 5. Walk/cycle infrastructure: *Low SES:* *R^2^=*not reported, p>.05 *High SES: R^2^=*not reported, p>.05 (Cycle/Walk-friendly infrastructure **0*0.55; 0*0.45**) 6. Aesthetics: *Low SES:* *R^2^=*not reported, p>.05 *High SES: R^2^=*not reported, p>.05 (Greenery and aesthetically pleasing scenery **0*0.55; 0*0.45**) 7. Safety from traffic: *Low SES:* *R^2^=*not reported, p>.05 *High SES: R^2^=*not reported, p>.05 (Traffic/pedestrian safety **0*0.55; 0*0.45**) 8. Safety from crime: *Low SES:* *R^2^=*0.41, p=.04 (Crime/personal safety **+*0.55**) *High SES: R^2^=*not reported, p>.05 (Crime/personal safety **0*0.45**)  ***Main effects with objectively measured TotalMVPAMatthews(150+ mins/wk; Yes/No):*** 1. Residential density: *Low SES:* *R^2^=*not reported, p>.05 *High SES: R^2^=*not reported, p>.05 (Residential density **0*0.55; 0*0.45**) 2. Land-use mix: diversity: *Low SES:* *R^2^=*not reported, p>.05 *High SES: R^2^=*not reported, p>.05 (Land-use mix—destination diversity **0*0.55; 0*0.45**) 3. Land-use mix: access: *Low SES:* *R^2^=*not reported, p>.05 *High SES: R^2^=*not reported, p>.05 (Destinations/services (unspecified) **0*0.55; 0*0.45**) 4. Street connectivity: *Low SES:* *R^2^=*not reported, p>.05 *High SES: R^2^=*not reported, p>.05 (Street connectivity **0*0.55; 0*0.45**) 5. Walk/cycle infrastructure: *Low SES:* *R^2^=*not reported, p>.05 *High SES: R^2^=*not reported, p>.05 (Cycle/Walk-friendly infrastructure **0*0.55; 0*0.45**) 6. Aesthetics: *Low SES:* *R^2^=*not reported, p>.05 *High SES: R^2^=*not reported, p>.05 (Greenery and aesthetically pleasing scenery **0*0.55; 0*0.45**) 7. Safety from traffic: *Low SES:* *R^2^=*not reported, p>.05 *High SES: R^2^=*not reported, p>.05 (Traffic/pedestrian safety **0*0.55; 0*0.45**) 8. Safety from crime: *Low SES:* *R^2^=*not reported, p>.05 *High SES: R^2^=*not reported, p>.05 (Crime/personal safety **0*0.55; 0*0.45**) | SES effects. Table 5. |
| 55 | *No study name* Lee & Park, 2015 [75] | N=437 (mixed) 65+ years 100% female Response rate not reported Dwelling not reported Seongnam City and South Chungnam Province, South Korea | Cross-sectional Cluster: purposive Individuals: convenience Stratification: population size, population density (urbanisation) and importance for primary industry Neighbourhood definition: 15-20mins walk from home | Age, income, education, social support, attitude, intention, self-efficacy | *Self-report [unnamed questionnaire; unvalidated]:*  Total walking (mins/wk) 🡪 Total walking *TotalWalking(150+ mins; Yes/No)* | *Perceived: [NEWS questionnaire; validated]:*  1. Proximity to parks 🡪 Parks/public open space access/availability 2. Proximity to stores 🡪 Shops/commercial access/availability 3. Street trees 🡪 Greenery and aesthetically pleasing scenery 4. Traffic safety 🡪 Traffic/pedestrian safety 5. Street lights 🡪 Street lighting 6. Crime safety 🡪 Crime/personal safety | Urbanisation:  Rural (n=221), Urban (n=216) | Logistic regression | ***Main effects with TotalWalking(150+ mins/wk; Yes/No):*** 1. Proximity to parks—OR (95% CIs): *Urban:* OR=0.68 (0.24; 1.94) p=.47 (Parks/public open space access/availability **0*0.49**) *Rural:* OR=3.02 (1.07; 8.56) p=.04 (Parks/public open space access/availability **+*0.51**) 2. Proximity to stores: *Urban:* OR=1.69 (0.63; 4.59) p=.30 *Rural:* OR=0.43 (0.12; 1.54) p=.20 (Shops/commercial access/availability **0*0.49; 0*0.51**) 3. Street trees: *Urban:* OR=2.07 (0.76; 5.68) p=.16 (Greenery and aesthetically pleasing scenery **0*0.49**) *Rural:* OR=2.73 (1.02; 7.34) p=.04 (Greenery and aesthetically pleasing scenery **+*0.51**) 4. Traffic safety: *Urban:* OR=1.05 (0.49; 2.24) p=.90 *Rural:* OR=1.00 (0.40; 2.47) p=1.0 (Traffic/pedestrian safety **0*0.49; 0*0.51**) 5. Street lights:  *Urban:* OR=0.61 (0.25; 1.51) p=.28 *Rural:* OR=1.73 (0.63; 4.79) p=.29 (Street lighting **0*0.49; 0*0.51**) 6. Crime safety: *Urban:* OR=2.68 (1.06; 6.77) p=.04 (Crime/personal safety **+*0.49**) *Rural:* OR=1.15 (0.51; 2.56) p=.74 (Crime/personal safety **0*0.51**) | Urbanisation effects. Table 4. |
| 56 | *No study name* Lotfi & Koohsari, 2011 [146] | N=238 (urban) 65+ years % female not reported Response rate not reported Dwelling not reported Tehran, Iran | Cross-sectional Cluster: purposive Individuals: random Stratification: walkability and social vulnerability (proxy for SES) Neighbourhood definition: administrative boundary | None | *Self-report [unnamed questionnaire; unvalidated]:*  Total walking (10+ mins/occurrence) 🡪 Total walking *TotalWalking(10+ mins/occurrence; Yes/No)* | *Objective [Tehran Traffic Control Centre; Statistical Centre of Iran; unvalidated]:*  1. Walkability 🡪 Walkability | Walkability*SES:  High + Low (n=48), Low + Low (n=40), High + High (n=65), Low + High (n=85) | *No formal analysis conducted—percentages reported* | ***Association with TotalWalking(10+ min/occurrence; Yes/No):*** 1. Walkability:  High walkability + low social vulnerability: Less than 2 times/wk: 10% 2-5 times/wk: 30% 6-10 times/wk: 36% ≥10 times/wk: 24% High walkability + high social vulnerability: Less than 2 times/wk: 12% 2-5 times/wk: 24% 6-10 times/wk: 42% ≥10 times/wk: 22% Low walkability + low social vulnerability: Less than 2 times/wk: 21% 2-5 times/wk: 47% 6-10 times/wk: 28% ≥10 times/wk: 4%  Low walkability + high social vulnerability: Less than 2 times/wk: 23% 2-5 times/wk: 54% 6-10 times/wk: 17% ≥10 times/wk: 6% (Walkability **+*0.37; 0*0.63**) | Moderating effects. Table 4. |
| 57 | *No study name* Maisel et al., 2016 [76] | N=121 (mixed) 65+ years 74% female Response rate not reported Community-dwellers Erie County NY, USA | Cross-sectional Cluster: purposive Individuals: convenience Stratification: area-level SES and urbanisation Neighbourhood definition: participant delineation | Age, sex, household income | *Self-report [IPAQ; validated]:*  Total neighbourhood walking (>0 mins/wk; Yes/No) 🡪 Total walking *TotalWalking(>0 mins/wk; Yes/No)* | *Perceived [NEWS questionnaire; validated]:*  1. Residential density 🡪 Residential density 2. Street connectivity 🡪 Street connectivity 3. Land-use mix: access 🡪 Destinations/services (overall/unspecific) access/availability 4. Land-use mix: diversity 🡪 Land-use mix—destination diversity 5. Aesthetics 🡪 Greenery and aesthetically pleasing scenery 6. Walking/cycling facilities 🡪 Cycle/walk-friendly infrastructure 7. Crime safety 🡪 Crime/personal safety 8. Traffic safety 🡪 Traffic/pedestrian safety | Urbanisation: Rural (n=39), Suburban (n=50), Urban (n=32) | Logistic regression. No testing of interaction terms. No adjustment for senior centre cluster. Continuous environmental exposure variables categorised. | ***Main and moderated effects with TotalWalking(>0 mins/wk; Yes/No):*** 1. Residential density—OR (95% CIs): *Overall participants:* OR=not reported, p>.05 (Residential density **0*1**) 2. Street connectivity: *Overall participants:* OR=2.34 (1.07; 5.10), p<.05 (Street connectivity **+*1**) 3. Land-use mix: access: *Rural participants:* OR=not reported, p>.05 *Suburban participants:* OR=not reported, p=.009 *Urban participants:* OR=not reported, p>.05 (Destinations/services (unspecified access/availability **0*0.32; 0*0.41; 0*0.27**) 4. Land-use mix: diversity: *Overall participants:* OR=not reported, p>.05 (Land-use mix—destination diversity **0*1**) 5. Aesthetics: *Overall participants:* OR=not reported, p>.05  (Greenery and aesthetically pleasing scenery **0*1**) 6. Walking/cycling facilities: *Overall participants:* OR=not reported, p>.05 (Cycling/Walk-friendly infrastructure **0*1**) 7. Crime safety: *Rural participants:* OR=11.23 (1.67; 77.56), p<.05 (Crime/personal safety **+*0.32**) *Suburban participants:* OR=not reported, p>.05 (Crime/personal safety **0*0.41**) *Urban participants:* OR=not reported, p>.05 (Crime/personal safety **0*0.27**) 8. Traffic safety: *Overall participants:* OR=not reported, p>.05 (Traffic/pedestrian safety **0*1**) | Moderating effects. |
| 58 | *No study name* Mowen et al., 2007 [151] | N=1515 (not reported) Mean age: 67 years 66% female 45% response rate Community-dwellers Ohio, USA | Cross-sectional Cluster: purposive Individuals: random Stratification: SES and sociodemographics (unspecified) Neighbourhood definition: participant delineation – “walking distance from home” (perceived), nearest park (objective) | None | *Self-report [unnamed questionnaire; unvalidated]:*  Total PA (ordinal score) 🡪 Total PA *TotalPA(score)* | *Perceived [unnamed questionnaire; unvalidated]:*  1. Park within walking distance 🡪 Parks/public open space access/availability  *Objective [GIS; unvalidated]:*  2. Distance to closest park 🡪 Parks/public open space access/availability | None | Univariate linear regression | ***Main effects with TotalPA(score):*** Perceived environment: 1. Park within walking distance: b=0.025, p=.581 (Parks/public open space access/availability **0**)  Objective environment: 2. Distance to closest park: b=-0.042, p=.354 (Parks/public open space access/availability **0**) | Table 1. |
| 59 | *No study name* Pelclova et al., 2012 [154] | N=456 (not reported) Mean age: ≈65 years 88% female Response rate not reported Community-dwellers Zlin, Olomouc, Usit, Labem and Brno, Czech Republic; Katowice, Poland; Presov, Slovakia | Cross-sectional Cluster: none Individuals: all eligible participants enrolled in University of Third Age invited Stratification: none Neighbourhood definition: participant delineation | None (no socio-demographics). Perceived environmental variables were entered as covariates | *Self-report [IPAQ—Polish and Slovakian translation; validated]:*  Total walking (30+ mins/d*5 d/wk) 🡪 Total walking *TotalWalking(30+ mins/d*5d/wk; Yes/No)* | *Perceived [NEWS questionnaire—modified version; unvalidated]:*  1. Residential density 🡪 Residential density 2. Land-use mix: proximity 🡪 Destinations/services (overall/unspecific) access/availability 3. Accessibility 🡪 Destinations/services (overall/unspecific) access/availability 4. Street connectivity 🡪 Street connectivity 5. Infrastructure for walking/cycling 🡪 Cycle/walk-friendly infrastructure 6. Neighbourhood aesthetics 🡪 Greenery and aesthetically pleasing scenery | None | Multivariate logistic regression (study site not accounted for). | ***Main effects with TotalWalking(30+ mins/d*5 d/wk; Yes/No):*** 1. Residential density—OR (95% CIs): OR=1.81 (1.13; 2.89), p<.05 (Residential density **+**) 2. Land-use mix: proximity: OR=0.92 (0.60; 1.42), p>.05 (Destinations/services (overall/unspecific) access/availability **0**) 3. Accessibility: OR=1.16 (0.46; 2.94), p>.05 (Destinations/services (overall/unspecific) access/availability **0**) 4. Street connectivity: OR=0.75 (0.20; 2.80), p>.05 (Street connectivity **0**) 5. Infrastructure for walking/cycling: OR=1.17 (0.54; 2.54), p>.05 (Cycling/Walk-friendly infrastructure **0**) 6. Neighbourhood aesthetics: OR=1.61 (0.92; 2.81), p>.05 (Greenery and aesthetically pleasing scenery **0**) | Table 2. |
| 60 | *No study name* Persson et al., 2012 [156] | N=225 (urban) Mean age: 75 years 77% female  88.6% response rate Community-dwellers London, United Kingdom | Cross-sectional Cluster: purposive Individuals: convenience Stratification: Boroughs with high percentage of older adults and health inequalities Neighbourhood definition: not defined | Age, sex, education, borough lived in, living with someone, social class, ethnicity, health status, health problems, smoking status, BMI | *Self-report [unnamed questionnaire; unvalidated]:*  Sufficient PA (30+ mins/d*5 d/wk) 🡪 Total MVPA *TotalMVPA(30+ mins/d*5d/wk; Yes/No)* | *Perceived [unnamed questionnaire; unvalidated]:*  1. Good facilities for exercising 🡪 Recreational facilities access/availability  2. Access to public transport 🡪 Public transport access/availability 3. Feel safe walking outside when dark 🡪 Crime/personal safety 4. Good leisure and social facilities in general 🡪 Social recreational facilities access/availability  5. Good facilities for people aged 60+ years 🡪 Social recreational facilities access/availability  6. Graffiti and vandalism 🡪 Greenery and aesthetically pleasing scenery 7. Access to public outdoor areas 🡪 Parks/public open space access/availability 8. Proximity to shops 🡪 Shops/commercial access/availability 9. Proximity to health services 🡪 Health and aged care access/availability 10. Litter and rubbish collection 🡪 Greenery and aesthetically pleasing scenery 11. Speed and volume of traffic 🡪 Traffic/pedestrian safety 12. Crime 🡪 Crime/personal safety 13. Air pollution 🡪 Pollution | None | Forward stepwise logistic regression | ***Main effects with TotalMVPA(30+ mins/d*5d/wk; Yes/No):*** 1. Good facilities for exercising—OR (95% CIs): OR=not reported, p=.047 (Recreational facilities access/availability **+**) 2. Access to public transport: OR=2.04 (not reported), p>.05 (Public transport access/availability **0**) 3. Feel safe walking outside when dark: OR=1.33 (not reported), p>.05 (Crime/personal safety **0**) 4. Good leisure and social facilities: OR=1.88 (0.42; 8.53), p>.05 (Social recreational facilities access/availability **0**) 5. Good facilities for people aged 60+ years: OR=1.26 (0.39; 4.07), p>.05 (Social recreational facilities access/availability **0**) 6. Graffiti and vandalism: OR=0.57 (0.22; 1.45), p>.05 (Greenery and aesthetically pleasing scenery **0**) 7. Access to public outdoor areas: OR=not reported, p>.05 (Parks/public open space access/availability **0**) 8. Proximity to shops: OR=not reported, p>.05 (Shops/commercial access/availability **0**) 9. Proximity to health services: OR=not reported, p>.05 (Health and aged care access/availability **0**) 10. Litter and rubbish collection: OR=not reported, p>.05 (Greenery and aesthetically pleasing scenery **0**) 11. Speed and volume of traffic: OR=not reported, p>.05 (Traffic/pedestrian safety **0**) 12. Crime: OR=not reported, p>.05 (Crime/personal safety **0**) 13. Air pollution: OR=not reported, p>.05 (Pollution **0**) | In-text. |
| 61 | *No study name* Salvador et al., 2010 [23] | N=385 (urban) 60-74 years 61% female 72.6% response rate Community-dwellers Sao Paulo, Brazil | Cross-sectional Cluster: purposive Individuals: random Stratification: census tracts (SES) Neighbourhood definition: 15-20mins walk from home | Age, education | *Self-report [IPAQ; validated]:*  Walking (150+ mins/wk; Yes/No) 🡪 Total walking *TotalWalking(150+ mins/wk; Yes/No)* | *Perceived [NEWS questionnaire—Brazilian version; unvalidated]:*  1. Presence of [meeting] square 🡪 Parks/public open space access/availability 2. Presence of social places (bars) 🡪 Social Recreational facilities access/availability  3. Walking time to parks 🡪 Parks/public open space access/availability 4. Walking time to public squares 🡪 Parks/public open space access/availability 5. Walking time to places to walk 🡪 Destinations/services (overall/unspecific) access/availability 6. Walking time to gyms 🡪 Recreational facilities access/availability  7. Walking time to clubs 🡪 Recreational facilities access/availability  8. Walking time to sport courts 🡪 Recreational facilities access/availability  9. Walking time to soccer fields 🡪 Recreational facilities access/availability  10. Walking time to bus stops 🡪 Public transport access/availability 11. Walking time to train stations 🡪 Public transport access/availability  12. Walking time to health clinics 🡪 Health and aged care access/availability 13. Walking time to pharmacies 🡪 Health and aged care access/availability 14. Walking time to churches or religious temples 🡪 Religious institution access/availability  15. Walking time to bakeries 🡪 Food outlets access/availability 16. Walking time to bank branches 🡪 Government/finance access/availability 17. Walking time to bars 🡪 Social recreational facilities access/availability  18. Walking time to street fairs 🡪 Social recreational facilities access/availability 19. Walking time to stores 🡪 Shops/commercial access/availability 20. Walking time to markets 🡪 Shops/commercial access/availability 21. Walking time to supermarkets 🡪 Shops/commercial access/availability 22. Presence and quality of pavements/sidewalks 🡪 Pavement/footpath quality 23. Presence and quality of green areas 🡪 Parks/public open space 24. Streets steepness 🡪 No physical barriers to walking 25. Presence of litter 🡪 Greenery and aesthetically pleasing scenery 26. Absence of open-air sewers 🡪 Pollution 27. Heavy traffic 🡪 Traffic/pedestrian safety 28. Pedestrian crossing close to home 🡪 Street connectivity 29. Drivers usually respect pedestrians on crossing 🡪 Traffic/pedestrian safety 30. Street lights close to home, well lit at night 🡪 Street lighting 31. Safe to walk during day 🡪 Crime/personal safety 32. Safe to walk during night 🡪 Crime/personal safety 33. Presence of soccer fields 🡪 Recreational facilities access/availability  34. Presence of places to walk in the district 🡪 Destinations/services (overall/unspecific) access/availability  35. Smoke pollution close to home 🡪 Pollution | Sex:  Male (n=152), Female (n=233) | Multivariate logistic regression | ***Main effects TotalWalking(150+ mins/wk; Yes/No):*** 1. Presence of [meeting] square—OR (95% CIs): *Males:* OR=not reported, p>.05 (Parks/public open space access/availability **0*0.39**) *Females:* OR=4.70 (95% CI=1.43; 15.43), p=.012 (Parks/public open space access/availability **+*0.61**) 2. Presence of social places (bar): *Males:* OR=not reported, p>.05 *Females:* OR=2.28 (0.13; 41.25), p=.566 (Social recreational facilities access/availability **0*0.39; 0*0.61**) 3. Walking time to parks: *Males:* OR=not reported, p>.05 *Females:* OR=not reported, p>.05 (Parks/public open space access/availability **0*0.39; 0*0.61**) 4. Walking time to public squares: *Males:* OR=not reported, p>.05 *Females:* OR=not reported, p>.05 (Parks/public open space access/availability **0*0.39; 0*0.61**) 5. Walking time to places to walk: *Males:* OR=2.23 (0.67; 7.40), p=.181  *Females:* OR=2.23 (0.67; 7.40), p=.181 (Destinations/services (overall/unspecific) access/availability **0*0.39; 0*0.61**) 6. Walking time to gyms: *Males:* OR=not reported, p>.05 *Females:* OR=not reported, p>.05 (Recreational facilities access/availability **0*0.39; 0*0.61**) 7. Walking time to clubs: *Males:* OR=not reported, p>.05 *Females:* OR=not reported, p>.05 (Recreational facilities access/availability **0*0.39; 0*0.61**) 8. Walking time to sports courts: *Males:* OR=not reported, p>.05 *Females:* OR=not reported, p>.05 (Recreational facilities access/availability **0*0.39; 0*0.61**) 9. Walking time to soccer fields: *Males:* OR=3.43 (1.46; 8.10), p=.006 (Recreational facilities access/availability **+*0.39**) *Females:* OR=1.58 (0.64; 3.90), p=.307 (Recreational facilities access/availability **0*0.61**) 10. Walking time to bus stops: *Males:* OR=not reported, p>.05 *Females:* OR=not reported, p>.05 (Public transport access/availability **0*0.39; 0*0.61**) 11. Walking time to train stations: *Males:* OR=not reported, p>.05 *Females:* OR=not reported, p>.05 (Public transport access/availability **0*0.39; 0*0.61**) 12. Walking time to health clinics: *Males:* OR=not reported, p>.05 (Health and aged care access/availability **0*0.39**) *Females:* OR=3.71 (1.19; 11.54), p=.025 (Health and aged care access/availability **+*0.61**) 13. Walking time to pharmacies: *Males:* OR=not reported, p>.05 *Females:* OR=2.45 (0.88; 6.81), p=.084 (Health and aged care access/availability **0*0.39; 0*0.61**) 14. Walking time to churches or religious temples: *Males:* OR=not reported, p>.05 *Females:* OR=not reported, p>.05 (Religious institution access/availability **0*0.39; 0*0.61**) 15. Walking time to bakeries: *Males:* OR=not reported, p>.05 *Females:* OR=not reported, p>.05 (Food outlets access/availability **0*0.39; 0*0.61**) 16. Walking time to bank branches: *Males:* OR=not reported, p>.05 *Females:* OR=not reported, p>.05 (Government/finance services access/availability **0*0.39; 0*0.61**) 17. Walking time to bars: *Males:* OR=not reported, p>.05 *Females:* OR=not reported, p>.05 (Social recreational facilities access/availability **0*0.39; 0*0.61**) 18. Walking time to street fairs: *Males:* OR=not reported, p>.05 *Females:* OR=not reported, p>.05 (Social recreational facilities access/availability **0*0.39; 0*0.61**) 19. Walking time to stores: *Males:* OR=not reported, p>.05 *Females:* OR=not reported, p>.05 (Shops/commercial access/availability **0*0.39; 0*0.61**) 20. Walking time to markets: *Males:* OR=not reported, p>.05 *Females:* OR=not reported, p>.05 (Shops/commercial access/availability **0*0.39; 0*0.61**) 21. Walking time to supermarkets: *Males:* OR=not reported, p>.05 *Females:* OR=not reported, p>.05 (Shops/commercial access/availability **0*0.39; 0*0.61**) 22. Presence and quality of pavements/sidewalks: *Males:* OR=not reported, p>.05 *Females:* OR=not reported, p>.05 (Pavement/footpath quality **0*0.39; 0*0.61**) 23. Presence and quality of green areas: *Males:* OR=not reported, p>.05 *Females:* OR=not reported, p>.05 (Parks/public open space access/availability **0*0.39; 0*0.61**) 24. Streets steepness: *Males:* OR=not reported, p>.05 *Females:* OR=not reported, p>.05 (No physical barriers to walking **0*0.39; 0*0.61**) 25. Presence of litter: *Males:* OR=not reported, p>.05 *Females:* OR=not reported, p>.05 (Greenery and aesthetically pleasing scenery **0*0.39; 0*0.61**) 26. Absence of open-air sewers: *Males:* OR=2.18 (0.56; 8.52), p=.253 *Females:* OR=not reported, p>.05 (Pollution **0*0.39; 0*0.61**) 27. Heavy traffic: *Males:* OR=not reported, p>.05 *Females:* OR=2.88 (0.99; 8.41), p=.052 (Traffic/pedestrian safety **0*0.39; 0*0.61**) 28. Pedestrian crossing close to home: *Males:* OR=not reported, p>.05 *Females:* OR=not reported, p>.05 (Street connectivity **0*0.39; 0*0.61**) 29. Drivers usually respect pedestrians on crossing: *Males:* OR=not reported, p>.05 *Females:* OR=5.29 (0.90; 31.10), p=.064 (Traffic/pedestrian safety **0*0.39; 0*0.61**) 30. Street lights close to home, well lit at night: *Males:* OR=2.00 (0.45; 8.90), p=.353 *Females:* OR=not reported, p>.05 (Street lighting **0*0.39; 0*0.61**) 31. Safe to walk during the day: *Males:* OR=not reported, p>.05 *Females:* OR=not reported, p>.05 (Crime/personal safety **0*0.39; 0*0.61**) 32. Safe to walk during the night: *Males:* OR=1.53 (0.41; 5.61) p=.514 *Females:* OR=not reported, p>.05 (Crime/personal safety **0*0.39; 0*0.61**) 33. Presence of soccer fields: *Males:* OR=4.12 (1.41; 12.02), p=.011 (Recreational facilities access/availability **+*0.39**) *Females:* OR=not reported, p>.05 (Recreational facilities access/availability **0*0.61**) 34. Presence of places to walk in the district: *Males:* OR=2.23 (0.67; 7.40), p=.181  *Females:* OR=not reported, p>.05 (Destinations/services (overall/unspecific) access/availability **0*0.39; 0*0.61**) 35. Smoke pollution close to home: *Males:* OR=2.23 (0.67; 7.40), p=.181  *Females:* OR=not reported, p>.05 (Pollution **0*0.39; 0*0.61**) | Sex effects. Tables 3 and 4. |
| 62 | *No study name* Sewo Sampaio et al., 2013 [160] | N=465 (mixed) Mean age: 69.0 years 48.7% female 56% response rate Community-dwellers *Multiple locations,* Japan | Cross-sectional Cluster: purposive Individuals: convenience Stratification: urbanisation Neighbourhood definition: urban and rural | None | *Self-report [unnamed questionnaire; unvalidated]:*  Physical activity times/wk (e.g., walking, sports etc.) 🡪 Total PA *TotalPA(median times/wk)* | *Objective [census data; unvalidated]:*  1. Urbanisation 🡪 Urbanisation | Urbanisation: Rural (n=178) Urban (n=287) | Chi-square test | ***Main effects with TotalPA(median times/wk):*** 1. Urbanisation: Rural vs. Urban: 3 vs. 4, p<.01 (Urbanisation **+**) | Table 3. |
| 63 | *No study name* Shin et al., 2011 [161] | N=80 (urban) Mean age: 66.8 years 100% female 33.9% response rate Community-dwellers Texas, USA | Cross-sectional Cluster: purposive Individuals: purposive and convenient (2 distinct stages) Stratification: sex and ethnicity (≥80% African-American/census block) Neighbourhood definition: 0.5mile, and 1mile buffer | Age, employment status, household members, self-rated health | *Self-report [CHAMPS questionnaire—modified version; unvalidated]:*  Total PA (caloric expenditure/wk/kg 🡪 TotalPA *TotalPA(cal/wk/kg)* | *Objective [GIS, ENVI, FRAGSTATS; unvalidated]:*  1. Density of green spaces 🡪 Parks/public open space access/availability 2. Number of accessible green spaces 🡪 Parks/public open space access/availability 3. Greenery density 🡪 Greenery and aesthetically pleasing scenery 4. Street greenery density 🡪 Aesthetics and greenspace 5. Land-use mix 🡪 Land-use mix—destination diversity 6. Intersection density 🡪 Street connectivity 7. Cul-de-sac density 🡪 Street connectivity 8. Street density 🡪 Street connectivity 9. Commercial density 🡪 Shops/commercial access/availability 10. Sidewalk connectivity 🡪 Walk-friendly infrastructure 11. Distance to closest green space 🡪 Parks/public open space access/availability 12. Distance to closest commercial area 🡪 Shops/commercial access/availability 13. Distance to closest school 🡪 Education facilities access/availability 14. Distance to closest church 🡪 Religious institution access/availability | None | Path analyses | ***Main effects with TotalPA(cal/wk/kg):*** 1. Density of green spaces: *0.5 mile:* b=not reported, p>.05 *1 mile:* b=not reported, p>.05 (Parks/public open space access/availability **0*0.5; 0*0.5**) 2. Number of accessible green spaces: *0.5 mile:* b=not reported, p>.05 *1 mile:* b=not reported, p>.05 (Parks/public open space access/availability **0*0.5; 0*0.5**) 3. Greenery density: *0.5 mile:* b=4.99, p=.07) *1 mile:* b=not reported, p>.05 (Greenery and aesthetically pleasing scenery 0***0.5; 0*0.5**) 4. Street greenery density: *0.5 mile:* b=not reported, p>.05 *1 mile:* b=not reported, p>.05 (Greenery and aesthetically pleasing scenery **0*0.5; 0*0.5**)  5. Land-use mix: *0.5 mile:* b=not reported, p>.05 *1 mile:* b=not reported, p>.05 (Land-use mix—destination diversity **0*0.5; 0*0.5**) 6. Intersection density: *0.5 mile:* b=not reported, p>.05 *1 mile:* b=0.24, p=.10 (Street connectivity **0*0.5; 0*0.5**) 7. Cul-de-sac density: *0.5 mile:* b=not reported, p>.05 *1 mile:* b=not reported, p>.05 (Street connectivity **0*0.5; 0*0.5**) 8. Street density: *0.5 mile:* b=not reported, p>.05 *1 mile:* b=not reported, p>.05 (Street connectivity **0*0.5; 0*0.5**) 9. Commercial density: *0.5 mile:* b=not reported, p>.05 *1 mile:* b=not reported, p>.05 (Shops/commercial access/availability **0*0.5; 0*0.5**) 10. Sidewalk connectivity: *0.5 mile:* b=not reported, p>.05 *1 mile:* b=not reported, p>.05 (Walk-friendly infrastructure **0*0.5; 0*0.5**) 11. Distance to closest green space: *0.5 mile:* b=not reported, p>.05 *1 mile:* b=not reported, p>.05 (Parks/public open space access/availability **0*0.5; 0*0.5**) 12. Distance to closest commercial area: *0.5 mile:* b=not reported, p>.05 *1 mile:* b=not reported, p>.05 (Shops/commercial access/availability **0*0.5; 0*0.5**) 13. Distance to closest school: *0.5 mile:* b=not reported, p>.05 *1 mile:* b=not reported, p>.05 (Education facilities access/availability **0*0.5; 0*0.5**) 14. Distance to closest church: *0.5 mile:* b=not reported, p>.05 *1 mile:* b=not reported, p>.05 (Religious institution access/availability **0*0.5; 0*0.5**) | Table 6. |
| 64 | *No study name* Shores et al., 2009 [68] | N=449 (rural) 65+ years 46.8% female 38% response rate Community-dwellers Western North Carolina, USA | Cross-sectional Cluster: purposive Individuals: random Stratification: population density (urbanisation) Neighbourhood definition: county level | Age, sex, BMI, income | *Self-report [unnamed questionnaire; unvalidated]:*  Achievers (MPA/VPA: 20+ mins/d*5 d/wk) 🡪 Total MVPA *TotalMVPA(20+ mins/d*5 d/wk; Yes/No)* | *Perceived [unnamed questionnaire; unvalidated]:*  1. Concerned about safety in recreation areas 🡪 Crime/personal safety 2. Walking distance to a park 🡪 Parks/public open space access/availability 3. Recreational facilities close to home 🡪 Recreational facilities access/availability  4. Access to recreational facilities via [public] transport 🡪 Public transport access/availability  *Perceived [NEWS questionnaire; validated]:* 5. “Walkability”: Sidewalk quality, presence of a sidewalk, separation from traffic 🡪 Walk-friendly infrastructure | None | ANCOVA | ***Main effects with TotalMVPA(20+ mins/d*5+ d/wk; Yes/No):***  1. Concerned about safety in recreation areas: f=12.6, p=.001 (Crime/personal safety **+**) 2. Walking distance to a park: f=3.94, p=.049 (Parks/public open space access/availability **+**) 3. Recreational facilities close to home: f=7.05, p=.009 (Recreational facilities access/availability **+**) 4. Access to recreational facilities via [public] transport: f=1.55, p=.215 (Public transport **0**) 5.“Walkability”: f=2.09, p=.063 (Walk-friendly infrastructure **0**) | Table 2. |
| 65 | *No study name* Tanaka et al., 2016 [164] | N=108 (urban) 65+ years 100% female Response rate not reported Community-dwellers Nagasaki City, Japan | Cross-sectional Cluster: purposive Individuals: convenience Stratification: land slope Neighbourhood definition: a place where the ground rises 20m above sea level or ≥5° angle | Comorbidities, pulmonary function, muscle force, depressive symptoms | *Objective [Lifecorder accelerometer; validated]:*  Total PA (mean counts/d) 🡪 Total PA *TotalPA(counts/d)* Total PA (activity times/d) 🡪 Total PA *TotalPA(activity times/d)* | *Objective [Public Health Nursing Care Insurance index; unvalidated]:*  1. Slope 🡪 No physical barriers to walking | None | Multivariate logistic regression | ***Main effects with TotalPA(counts/d):*** 1. Slope—b; OR (95% CIs): b=-2.001; OR=0.779 (0.715; 0.841), p=.002 (No physical barriers to walking **+**)  ***Main effects with TotalPA(activity times/d):*** 1. Slope: b=-.189; OR=0.821 (0.801; 0.913), p=.004 (No physical barriers to walking **+**) | Table 3. |
| 66 | *No study name* Towne Jr. et al., 2016 [167] | N=394 (urban) Mean age: 65 years 54% female 6.8% response rate Community-dwelling Texas, USA | Cross-sectional Cluster: convenience Individuals: convenience Stratification: none Neighbourhood definition: ≤30mins from home | Age, sex, race, household income, someone to walk with, marital status, physical function (difficulty with walking), median income (census level) | *Self-report [unnamed questionnaire; unvalidated]:*  Total walking (any purpose-- 150+ mins/wk; Yes/No) 🡪 Total walking *TotalWalking(150+ mins/wk; Yes/No)* | *Objective [Walk Score™; unvalidated]:*  1. Walk Score™ 🡪 Walkability  *Perceived [unnamed questionnaire; unvalidated]:*  2. *Aggregated variable:* Perceived neighbourhood environment 🡪 Crime/personal safety | None | Multivariate logistic regression | ***Main effects with TotalWalking(150+ mins/wk; Yes/No):*** 1. Walk Score™ (Ref:<50)—OR (90% CIs): 50+: OR =3.171 (1.480; 6.797), p=.0128 (Walkability **+**) 2. Perceived neighbourhood environment (Ref: Low): Medium: OR =1.862 (1.059; 3.275)  High: OR=2.671 (1.404; 5.082), p=.0406 (Crime/personal safety **+**) | Table 3. |
| 67 | *No study name* Wang & Lee 2010 [53] | N=114 and n=61 (urban) Mean age: 84 years 81.6% female 20-45% response rates Assisted-living facility residents Texas, USA | Cross-sectional Cluster: convenience Individuals: convenience Stratification: none Neighbourhood definition: not defined | None | *Self-report [unnamed questionnaire; unvalidated]:*  Neighbourhood walking at previous address (when community-dwelling) (1+ walks/d) 🡪 Total walking *TotalWalking(1+ walks/d; Yes/No)* Neighbourhood walking at previous address (when community-dwelling) (10+ mins/walking occurrence) 🡪 Total walking *TotalWalking(10+ mins/occurrence; Yes/No)* | *Perceived [unnamed questionnaire; unvalidated]:* Neighbourhood environment:  1. Number of walking destinations 🡪 Destinations/services (overall/unspecific) access/availability 2. Walking route choices 🡪 Street connectivity 3. Safety from crime 🡪 Crime/personal safety 4. Lighting conditions 🡪 Street lighting 5. Safety from traffic 🡪 Traffic/pedestrian safety 6. Visual interest 🡪 Greenery and aesthetically pleasing scenery 7. Usable sidewalks (sidewalks present) 🡪 Walk-friendly infrastructure  *Objective [GIS; unvalidated]:* Neighbourhood environment:  8. Distance to nearest drug store 🡪 Health and aged care access/availability 9. Distance to nearest general life facilities 🡪 Destinations/services (overall/unspecific) access/availability 10. Distance to nearest health care facilities 🡪 Health and aged care access/availability  11. Number of general daily life facilities within ¼ mile 🡪 Destinations/services (overall/unspecific) access/availability 12. Number of general life facilities within ½ mile 🡪 Destinations/services (overall/unspecific) access/availability 13. Number of general daily life facilities within 1 mile 🡪 Destinations/services (overall/unspecific) access/availability 14. Number of general daily life facilities within 2 miles 🡪 Destinations/services (overall/unspecific) access/availability 15. Distance to nearest park 🡪 Parks/public open space access/availability 16. Distance to bank 🡪 Government/finance access/availability 17. Distance to post office 🡪 Shops/commercial access/availability 18. Distance to food facility/establishments 🡪 Food outlets access/availability 19. Total amount of paved roads in the area within ¼ mile 🡪 Street connectivity 20. Total amount of paved roads in the area within ½ mile 🡪 Street connectivity 21. Total amount of paved roads in the area within 1 mile 🡪 Street connectivity 22. Total amount of paved roads in the area within 2 miles 🡪 Street connectivity | None | Chi-square test | ***Main effects with TotalWalking(1+ walks/d):*** Perceived environment: 1. Number of walking destinations: Χ^2^=13.81, p<.001 (Destinations/services (overall/unspecific) access/availability **+**) 2. Walking route choices: Χ^2^=28.47, p<.001 (Street connectivity +) 3. Safety from crime: Χ^2^=6.98, p=.008 (Crime/personal safety +) 4. Lighting conditions: Χ^2^=9.23, p=.002 (Street lighting **+**) 5. Safety from traffic: Χ^2^=4.04, p=.044 (Traffic/pedestrian safety **+**) 6. Visual interest: Χ^2^=12.62, p=.002 (Greenery and aesthetically pleasing scenery **+**) 7. Usable sidewalks: Χ^2^=11.30, p=.004  (Walk-friendly infrastructure **+**)  Objective environment: 8. Distance to nearest drug store: Χ^2^=11.89, p=.036 (Health and aged care access/availability **+**) 9. Distance to nearest general life facility: Χ^2^=not reported, p>.05 (Destinations/services (overall/unspecific) access/availability **0**) 10. Distance to nearest healthcare facility: Χ^2^=not reported, p>.05 (Health and aged care access/availability **0**) 11. Number of general life facilities within ¼ mile: Χ^2^=not reported, p>.05 (Destinations/services (overall/unspecific) access/availability **0*0.25**) 12. Number of general life facilities within ½ mile: Χ^2^=not reported, p>.05 (Destinations/services (overall/unspecific) access/availability **0*0.25**) 13. Number of general life facilities within 1 mile: Χ^2^=not reported, p>.05 (Destinations/services (overall/unspecific) access/availability **0*0.25**) 14. Number of general life facilities within 2 miles: Χ^2^=not reported, p>.05 (Destinations/services (overall/unspecific) access/availability **0*0.25**) 15. Distance to nearest park: Χ^2^=not reported, p>.05 (Parks/public open space access/availability **0**) 16. Distance to bank: Χ^2^=not reported, p>.05 (Government/finance services access/availability **0**) 17. Distance to post-office: Χ^2^=not reported, p>.05 (Shops/commercial access/availability **0**) 18. Distance to food facility/establishment: Χ^2^=not reported, p>.05 (Food outlets access/availability **0**) 19. Total paved roads within ¼ mile: Χ^2^=not reported, p>.05  (Street connectivity **0*0.25**) 20. Total paved roads within ½ mile: Χ^2^=not reported, p>.05  (Street connectivity **0*0.25**) 21. Total paved roads within 1 mile: Χ^2^=not reported, p>.05  (Street connectivity **0*0.25**) 22. Total paved roads within 2 miles: Χ^2^=not reported, p>.05  (Street connectivity **0*0.25**)  ***Main effects with TotalWalking(10+ mins/occurrence):*** Perceived environment: 1. Number of walking destinations: Χ^2^=11.97, p=.001 (Destinations/services (overall/unspecific) access/availability **+**) 2. Walking route choices: Χ^2^=21.78, p<.001  (Street connectivity **+**) 3. Safety from crime: Χ^2^=15.55, p<.001  (Crime/personal safety **+**) 4. Lighting conditions: Χ^2^=8.54, p=.003  (Street lighting **+**) 5. Safety from traffic: Χ^2^=6.72, p=.010  (Traffic/pedestrian safety **+**) 6. Visual interest: Χ^2^=7.07, p=.029  (Greenery and aesthetically pleasing scenery **+**) 7. Usable sidewalks: Χ^2^=not reported, p>.05  (Walk-friendly facilities/infrastructure **0**)  Objective environment: 8. Distance to drugstore: Χ^2^=not reported, p>.05 (Health and aged care access/availability **0**) 9. Distance to general life facility: Χ^2^=not reported, p>.05 (Destinations/services (overall/unspecific) access/availability **0**) 10. Distance to healthcare facility: Χ^2^=not reported, p>.05 (Health and aged care access/availability **0**) 11. Number of general life facilities within ¼ mile: Χ^2^=not reported, p>.05 (Destinations/services (overall/unspecific) access/availability **0*0.25**) 12. Number of general life facilities within ½ mile: Χ^2^=not reported, p>.05 (Destinations/services (overall/unspecific) access/availability **0*0.25**) 13. Number of general life facilities within 1 mile: Χ^2^=not reported, p>.05 (Destinations/services (overall/unspecific) access/availability **0*0.25**) 14. Number of general life facilities within 2 miles: Χ^2^=not reported, p>.05 (Destinations/services (overall/unspecific) access/availability **0*0.25**) 15. Distance to park: Χ^2^=not reported, p>.05 (Parks/public open space access/availability **0**) 16. Distance to bank: Χ^2^=not reported, p>.05 (Government/finance access/availability **0**) 17. Distance to post-office: Χ^2^=not reported, p>.05 (Shops/commercial access/availability **0**) 18. Distance to food facility/establishment: Χ^2^=not reported, p>.05  (Food outlets access/availability **0**) 19. Total paved roads within ¼ mile: Χ^2^=not reported, p>.05  (Street connectivity **0*0.25**) 20. Total paved roads within ½ mile: Χ^2^=13.75, p=.017  (Street connectivity **+*0.25**) 21. Total paved roads within 1 mile: Χ^2^=not reported, p>.05  (Street connectivity **0*0.25**) 22. Total paved roads within 2 miles: Χ^2^=not reported, p>.05  (Street connectivity **0*0.25**) | Buffer effects. Table 1. *Note. Perceived environment data (n=114); objectively assessed environmental data (n=61).* |
| 68 | *No study name* Wilcox et al., 2003 [69] | N=102 (rural) Mean age: 70.6 years (African-Americans), 71 years (White) 100% female Response rate not reported Dwelling not reported South Carolina, USA | Cross-sectional Cluster: purposive Individuals: convenience Stratification: ethnicity (African-American, White) Neighbourhood definition: participant delineation | Age, education, race, marital status, self-efficacy, decisional balance, depression, stress, social support, healthcare provider | *Self-report [PASE questionnaire; validated]:*  Total PA (score) 🡪 Total PA *TotalPA(score)* | *Perceived [unnamed questionnaire; unvalidated]:*  1. Perceived neighbourhood safety 🡪 Crime/personal safety 2. Traffic volume 🡪 Traffic/pedestrian safety 3. Street lighting 🡪 Street lighting 4. Unattended dogs 🡪 Crime/personal safety 5. Walking distance to a park 🡪 Parks/public open space access/availability 6. Absence of sidewalks 🡪 Walk-friendly infrastructure | Race:  African-American (n≈42), White (n≈60) | Hierarchical linear regression (trimmed model) | *Significant moderating effects for race*nearby park: r=<.01  No moderating effect for race*all other environmental exposure variables.* ***Main effects with TotalPA(score):*** 1. Perceived neighbourhood safety: b=0.20, p=.03 (Crime/personal safety **+**) 2. Traffic volume: b=not reported, p>.05 (Traffic/pedestrian safety **0**) 3. Street lighting: b=not reported, p>.05 (Street lighting **0**) 4. Unattended dogs: b=not reported, p>.05 (Crime/personal safety **0**) 5. Walking distance to park: b=not reported, p>.05 (Parks/public open space access/availability **0*0.40; 0*0.60**) 6. Absence of sidewalks: b=-0.21, p=.02 (Walk-friendly infrastructure **+**) | In-text, below Table 3. |
| 69 | NSW (New South Wales) Falls Prevention Baseline Survey 2009 data Macniven et al., 2014 [77] | N=1822 (not reported) 65+ years 58% female 60.8% response rate Community-dwellers New South Wales, Australia | Cross-sectional Cluster: purposive Individuals: random Stratification: health service area (none) Neighbourhood definition: not defined | Age, sex, education, BMI, SES (Socio-Economic Indexes for Areas (SEIFA)) | *Self-report [NSW Falls Prevention Survey questionnaire; unvalidated]:*  Total MVPA (150+ mins/wk; Yes/No) 🡪 Total MVPA *TotalMVPA(150+ mins/wk; Yes/No)* | *Perceived [unnamed questionnaire; unvalidated]:*  1. Availability of sports or gym facilities 🡪 Recreational facilities access/availability | Health status:  Good (n=1260), Bad (n=552) | Binary logistic regression | *No significant moderating effects.* ***Main effects with TotalMVPA(150+ mins/wk; Yes/No):*** 1. Availability of sports or gym facilities—OR (95% CIs): *Good health:* Yes (n=99) vs. No (n=1161) (Ref):  OR=1.04 (0.69; 1.57), p>.05 *Poor health:* Yes (n=40) vs. No (n=512) (Ref):  OR=1.56 (0.79; 3.06), p>.05 (Recreational facilities access/availability **0*0.696; 0*0.304**) | Table 2. |
| 70 | NSW Older People’s Health Survey (OPHS) Lim & Taylor 2005 [145] | N=4419 (mixed) 65+ years 47% female 70.7% response rate Community-dwelling New South Wales, Australia | Cross-sectional Cluster: purposive Individuals: random Stratification: health area (500 residents/area) (none) Neighbourhood definition: health area (administrative) | Age, sex, education (age left school), physical functioning, diabetes, eyesight, fruit and vegetables intake, caregiving, language spoken, living arrangements, employment, fear of falling, can travel independently | *Self-report [NSW OPHS questionnaire; unvalidated]:*  Adequate PA (30+ mins/d, 5 d/wk; Yes/No) 🡪 Total PA *TotalPA(30+ mins/d, 5 d/wk; Yes/No)* | *Perceived [OPHS questionnaire; unvalidated]:*  1. Area of residence 🡪 Urbanisation 2. Feel safe in neighbourhood 🡪 Crime/personal safety | None | Cox’s proportional hazards model | ***Main effects with TotalPA(30+ mins/d, 5 d/wk; Yes/No):*** 1. Area of residence (Ref: rural)—RR (95% CIs): Urban: RR=0.911 (0.871; 0.954), p<.001 (Urbanisation **-**) 2. Feel safe in the neighbourhood (Ref: some/none of the time): All/most of the time: RR=0.941 (0.854; 1.038), p>.05 (Crime/personal safety **0**) | Table 2—adjusted model. |
| 71 | Nurses’ Health Study James et al., 2013 [70] | N=34,952 (likely mixed/not reported) 66+ years 100% female 90% response rate Community-dwellers *48 states,* USA | Cross-sectional Cluster: purposive Individuals: convenience Stratification: sex and occupation Neighbourhood definition: postal code | Age, smoking, race, husband’s education | *Self-report [Nurses’ Health study questionnaire; validated]:*  Total PA (500+ MET mins/wk) 🡪 Total PA *TotalPA(500+ MET mins/wk; Yes/No)*  Total walking (>3 METs; 500+ MET mins/wk) 🡪 Total walking *TotalWalking(500+ MET mins/wk; Yes/No)* | *Objective [county sprawl index developed by Smart Growth America; unvalidated]:*  1. Urban sprawl 🡪 Urbanisation | Age:  66.2-72.5 y (n=17,633),  72.6-81.3 y (n=17,319) | Multilevel logistic regression accounting for clustering | ***Main effects with TotalPA(500+ MET mins/wk; Yes/No):*** 1. Urban sprawl—b (95% CIs): *66.2-72.5 years:* b=-0.12 (-0.54; 0.30), p>.05 72.6-81.3 years: b=-0.23 (-0.57; 0.11), p>.05 (Urbanisation **0*0.5; 0*0.5**)  ***Main effects with TotalWalking(500+ MET mins/wk; Yes/No):*** 1. Urban sprawl: *66.2-72.5 years:* b=0.23 (-0.07; 0.39), p<.05 *72.6-81.3 years:* b=0.17 (0.02; 0.31), p<.05 (Urbanisation **+*0.5; +*0.5**) | Table 3. |
| 72 | Nurses’ Health Study Troped et al., 2014 [168] | N=23,434 (likely mixed/not reported) Mean age: 70 years 100% female 90% response rate Community-dwellers California, Massachusetts, and Pennsylvania, USA | Cross-sectional Cluster: purposive Individuals: convenience Stratification: population density (urbanisation) and state (highest populations) Neighbourhood definition: 94.5% street number level; 800m, and 1200m buffers | Age, number of years at address, race, ethnicity, husband’s and nurse’s education, BMI, walking limitations, more relaxed indoors, smoking status | *Self-report [Nurses’ Health study questionnaire; validated]:*  Total walking (500+ MET mins/wk) 🡪 Total walking *TotalWalking(500+ MET mins/wk; Yes/No)* | *Objective [GIS, LandScan; unvalidated]:*  1. Population density 🡪 Residential density 2. Intersection density 🡪 Street connectivity 3. Total facilities 🡪 Destinations/services (overall/unspecific) access/availability 4. Retail/stores 🡪 Shops/commercial access/availability 5. Services 🡪 Destinations/services (overall/unspecific) access/availability 6. Cultural/Education 🡪 Education facilities access/availability 7. Physical activity 🡪 Recreational facilities access/availability  8. Restaurants 🡪 Food outlets access/availability 9. Fast food restaurants 🡪 Food outlets access/availability 10. Grocery stores 🡪 Shops/commercial access/availability 11. Convenience stores 🡪 Shops/commercial access/availability | Population density*facilities (n=cases): 0-20.0^th^ (n=1053), 20.1-40.0^th^ (n=1102), 40.1-60.0^th^ (n=1072), 60.1-80.0^th^ (n=1064), 80.1-90.0^th^ (n=511), 90.1-95.1^th^ (n=272), 95.1-100^th^ (n=292) | Multivariate logistic regression | ***Main and moderated effects with TotalWalking(500+ MET mins/wk; Yes/No):*** 1. Population density—OR (95% CIs): OR=1.04 (1.02; 1.07), p<.05 (Residential density **+**) 2. Intersection density (Ref: <2.00 ≥3-way intersections/km): 2.00-3.99: OR=1.18 (1.05; 1.34), p<.05  4.00-5.99: OR=1.28 (1.13; 1.44), p<.05 6.00-11.00: OR=1.23 (1.03; 1.48), p<.05 (Street connectivity **+*0.092; +*0.384; +*0.470; +*0.054**) 3. Total facilities: OR=1.04 (1.02; 1.07), p<.05 (Destinations/services (overall/unspecific) access/availability **+*0.29; 0*0.71**) 4. Retail/stores: OR=1.10 (1.05; 1.15), p<.05 (Shops/commercial access/availability **+*0.29; 0*0.71**) 5. Services: OR=1.53 (1.20; 1.95), p<.05 (Destinations/services (overall/unspecific) access/availability **+*0.14; 0*0.86**) 6. Cultural/Education: OR=1.15 (1.03; 1.28), p<.05 (Education facilities access/availability **+*0.14; 0*0.86**) 7. Physical activity facilities: OR=1.45 (0.98; 2.15), p>.05 (Recreational facilities access/availability **+*0.29; 0*0.71**) 8. Restaurants: OR=1.04 (1.01; 1.06), p<.05 (Food outlets access/availability **+*0.29; 0*0.86**) 9. Fast food restaurants: OR=1.01 (1.01; 1.02), p<.05 (Food outlets access/availability **+*0.43; 0*0.57**) 10. Grocery stores: OR=1.06 (1.00; 1.12), p<.05 (Shops/commercial access/availability **+*1**) 11. Convenience stores: OR=1.09 (1.04; 1.15), p<.05 (Shops/commercial access/availability **+*0.29; 0*0.71**) | Moderating effects. Table 2. |
| 73 | Oslo Health Study Piro et al., 2006 [157] | N=3499 (urban) 74/75 years 57% female 53.2% response rate Community-dwellers (apart from n=35, whom resided in an institution) Oslo, Norway | Cross-sectional Clusters: purposive Individuals: convenience  Stratification: none Neighbourhood definition: administrative borough | Education, medical conditions, marital status, income, fortune, time in current residence | *Self-report [Oslo Health Study questionnaire; unvalidated]:* Total PA (1+ hr/wk; Yes/No) 🡪 Total PA *TotalPA(1+ hr/wk; Yes/No)* | *Perceived [Oslo Health Study questionnaire; unvalidated]:*  1. Feeling safe walking alone in the evening 🡪 Crime/personal safety  *Objective [Oslo City Council data; unvalidated]:*  2. Neighbourhood violence (violent cases/1000 inhabitants) 🡪 Crime/personal safety | Sex:  Male (n=1409), Female (n=1864) | Multilevel logistic regression accounting for clustering | ***Moderated effects with TotalPA(1+ hr/wk; Yes/No):*** 1. Feeling safe walking alone at night (Ref: high)—OR (95% CIs): *Males:* Low: OR=0.68 (0.45; 1.02), p>.05 (Crime/personal safety **0*0.5**) *Females:* Low: OR=0.64 (0.47; 0.87), p<.05 (Crime/personal safety **-*0.5**)  2. Neighbourhood violence (Ref: low): *Males:* High: OR=0.54 (0.36; 0.81), p<.05 (Crime/personal safety **-*0.5**) *Females:* High: OR=1.08 (0.82; 1.42), p>.05 (Crime/personal safety **0*0.5**) | Moderating effects. Table 3 (male); Table 4 (female). |
| 74 | Physical Activity Monitor 2002 Pan et al., 2009 [78] | N=637 (not reported) Subsample: 60-79 years % female not reported 51% response rate Community-dwellers *All provinces,* Canada | Cross-sectional Cluster: none Individuals: random Stratification: population density (urbanisation) Neighbourhood definition: “local community” (variable/not fixed) | Age, education, self-rated health, family income, intention, self-efficacy, perceived barriers, perceived health benefits, social support, facility availability | *Self-report [IPAQ; validated]:* Sufficient PA (Yes/No) 🡪 Total MVPA *TotalMVPA(MPA: 30+ mins/d*7 d/wk; VPA: 20+ mins/d*3 d/wk; 840 MET mins/wk; Yes/No)* | *Perceived [Physical Activity Monitor questionnaire; unvalidated]:*  1. Availability of PA facilities – aggregated measure of number of: places to walk safely (inc., walking trails), places to ride a bike safely, publicly-owned multi-purpose recreation trails, facilities, places, recreation facilities, and other places (e.g., school gyms, skate parks) 🡪 Recreational facilities access/availability | None.  *Notes:* Age:  15-24 y (n=645),  25-39 y (n=1589), 40-64 y (n=2296), 65-79 y (n=637) | Multivariate logistic regression | ***Main effects with TotalMVPA(30+mins/d*7 d/wk; 20+mins/d*3 d/wk; 840 MET mins/wk; Yes/No):*** 1. Facility availability—OR (95% CIs): OR=0.94 (0.61; 1.44) (Recreational facilities access/availability **0**) | Table 3. |
| 75 | PACS (Physical Activity Cohort Scotland) McMurdo et al., 2012 [148] | N=547 (mixed) 65+ years 54% female 17% response rate Community-dwellers Scotland, United Kingdom | Cross-sectional Cluster: purposive Individuals: random Stratification: age, SES (index of deprivation) and urbanisation Neighbourhood definition: postal code | Age, perceived behavioural control, physical functioning, social cohesion (number of people can turn to nearby) | *Objective [RT3 accelerometer; validated]:*  Counts/min 🡪 Total PA *TotalPA(cpm)* | *Objective [GIS; yell.com ltd.; unvalidated]:*  1. Urbanisation 🡪 Urbanisation 2. % greenspace in the residential ward 🡪 Aesthetics and greenspace 3. Road distance to grocery shop or supermarket 🡪 Shops/commercial access/availability  *Perceived [Project OPAL questionnaire; unvalidated]:*  *Notes:* Local area surroundings, streets in your area, and personal safety not extracted as reported in Sniehotta et al. (2013). | None | Stepwise multiple linear regression | ***Main effects with TotalPA(cpm):*** 1. Urbanisation: b=not reported, p>.05  (Urbanisation **0**) 2. % greenspace in the residential ward: b=not reported, p>.05 (Greenery and aesthetically pleasing scenery **0**) 3. Road distance to grocery shop or supermarket: b=not reported, p>.05 (Shops/commercial access/availability **0**) | Table 2. Perceived environmental variables reported in Sniehotta et al. 2013 accounting for self-selection. |
| 76 | PACS Sniehotta et al., 2013 [79] | N=547 (mixed) 65+ years 54% female 17.5% response rate Community-dwellers Scotland, United Kingdom | Cross-sectional Cluster: purposive Individuals: random Stratification: age, SES (index of deprivation) and urbanisation Neighbourhood definition: 15-20mins walk from home | Age, sex, deprivation, intention, self-efficacy, physical functioning, bodily pain, general health, vitality, mental health, psychosocial health, depression, anxiety, need for support, received support, loneliness, neighbourhoodliness, sun, minimum temperature, maximum temperature, rainfall | *Objective [RT3 accelerometer; validated]:*  Counts/min 🡪 Total PA *TotalPA(cpm)* | *Perceived [Project OPAL questionnaire; unvalidated];*  1. Local area surroundings 🡪 Greenery and aesthetically pleasing scenery 2. Streets in your area 🡪 Street connectivity 3. Traffic 🡪 Traffic/pedestrian safety 4. Pedestrian safety 🡪 Traffic/pedestrian safety 5. Personal safety 🡪 Crime/personal safety | Social cognitions: Intention, Self-efficacy | Stepwise multiple linear regression | *No significant moderating effects.* ***Main effects with TotalPA(cpm):*** 1. Local area surroundings: b=0.02, p>.05 (Greenery and aesthetically pleasing scenery **0**) 2. Streets in your local area: b=-0.02, p>.05 (Street connectivity **0**) 3. Traffic: b=-0.05, p>.05 (Traffic/pedestrian safety **0**) 4. Pedestrian safety: b=-0.06, p>.05 (Traffic/pedestrian safety **0**) 5. Personal safety: b=0.20, p<.001 (Crime/personal safety **+**) | Table 4. |
| 77 | Project OPAL (Older People Active Living) Davis et al., 2011 [87] | N=214 (urban) Mean age: 78.1 years 49% female 20.8% response rate Dwelling not reported Bristol, United Kingdom | Cross-sectional Cluster: purposive Individuals: random Stratification: amenity access and SES (Index of Multiple Deprivation) Neighbourhood definition: 5min walk from home | None | *Self-report [daily trip log; unvalidated]:*  Total trips/wk 🡪 Total PA *TotalPA(trips/wk)* | *Perceived [OPAL questionnaire; unvalidated]:*  1. Amenities 🡪 Destinations/services (overall/unspecific) access/availability | None | One-way ANOVA | ***Main effects with TotalPA(trips/wk):*** 1. Amenities: None (n=18): 5.8±4.0, 1 (n=26): 8.7±4.3, 2-3 (n=56): 9.7±4.6, 4-7 (n=59): 9.8±5.1, 8+ (n=55): 11.1±4.9, F=4.5, p=.002 (Destinations/services (overall/unspecific) access/availability **+**) | Table 1. |
| 78 | Project OPAL Fox et al., 2011 [129] | N=240 (urban) 70+ years 48% female 20.4% response rate Dwelling not reported Bristol, United Kingdom | Cross-sectional Cluster: purposive Individuals: random Stratification: amenity access and SES (Index of Multiple Deprivation) Neighbourhood definition: distance to nearest shop | None | *Objective[ActiGraph accelerometer; validated; Freedson MVPA cutoff point (>1952 cpm); validated]:*  Counts/min 🡪 Total PA *TotalPA(cpm—logged)* Steps/d 🡪 Total walking *TotalWalking(steps/d)*  MVPA 🡪 Total MVPA *TotalMVPA(mins/d—logged)* | *Perceived [OPAL questionnaire; unvalidated]:*  1. Distance to nearest shop 🡪 Shops/commercial access/availability | None | One-way ANOVA  *Note.* MVPA mins/d and cpm were log-transformed) | ***Main effect with TotalPA(cpm—logged):*** 1. Distance to nearest shop: 0.27-0.63km: 4.9±0.8, 0.64-1.07km: 5.0±0.5, 1.08-6.52km: 5.1±0.7, p=.35 (Shops/commercial access/availability **0**)  ***Main effect with TotalMVPAFreedson(mins/d—logged):*** 1. Distance to nearest shop: 0.27-0.63km: 2.2±1.3, 0.64-1.07km: 2.4±1.0, 1.08-6.52km: 2.4±1.3, p=.53 (Shops/commercial access/availability **0**)  ***Main effect with TotalWalking(steps/d):*** 1. Distance to nearest shop: 0.27-0.63km: 4286±2899, 0.64-1.07km: 4472±2010, 1.08-6.52km: 4616±2472, p=.72 (Shops/commercial access/availability **0**) | Table 3. |
| 79 | Project OPAL Thompson et al., 2011 [165] | N=240 (urban) Mean age: 78.1 years 48% female 20.4% response rate Dwelling not reported Bristol, United Kingdom | Cross-sectional Cluster: purposive Individuals: random Stratification: amenity access and SES (Index of Multiple Deprivation) Neighbourhood definition: distance to nearest shop | None | *Objective[ActiGraph accelerometer; validated]:*  Steps/d 🡪 Total walking *TotalWalking(steps/d)* | *Perceived [Project OPAL questionnaire; unvalidated]:*  1. Minutes taken to reach the main food shopping venue 🡪 Shops/commercial access/availability | None | Spearman’s rank order correlations | ***Main effects with TotalWalking(steps/d):*** 1. Minutes to reach main food shopping venue: r=-0.100, p>.05 (Shops/commercial access/availability **0**) | Table 4. |
| 80 | Project RICE (Reaching Immigrants through Community Empowerment) pilot Han et al., 2016 [133] | n=106 (sub-sample) (urban) 65+ years 56% female Response rate not reported New York City, USA | Cross-sectional Cluster: none Individuals: convenient Stratification: ethnicity (Korean) Neighbourhood definition: participant delineation | Age, sex, years lived in USA, marital status, health insurance, BMI | *Self-report [Behavioral Risk Factor Surveillance System—Korean version; unvalidated]:* MVPA (min/d) 🡪 Total MVPA *TotalMVPA(150+ min/wk; Yes/No)* | *Perceived [Exercise Benefits and Barriers Scale—Korean version; unvalidated]:* 1. No safe place to exercise 🡪 Crime/personal safety | None | Multiple logistic regression analysis | ***Main effects with TotalMVPA(150+ min/wk; Yes/No):*** 1. No safe place to exercise—OR (95% CIs): OR=1.7 (0.5; 6.2), p=.410 (Crime/personal safety **0**) | Table 2. |
| 81 | SHAPE (Senior Health and Physical Exercise) Li et al., 2005a [92] | N=577 (urban) 65+ years  64% female 31% response rate Community-dwellers Portland, USA | Cross-sectional Cluster: purposive Individuals: random Stratification: population density, residential density, commercial density (urbanisation) and income Neighbourhood definition: street level (objective); 0.5mile buffer (objective); not defined (perceived) | None | *Self-report [SHAPE questionnaire; reliable]:*  Total neighbourhood walking (score) 🡪 Total walking *TotalWalking(score)* | *Objective [ArcGIS, Regional Land Information System data; unvalidated]:*  Neighbourhood level: 1. Employment density 🡪 Urbanisation 2. Household density 🡪 Residential density 3. Area of green and open space for recreation 🡪 Parks/public open space access/availability  Resident level: 4. Area of green and open space for recreation 🡪 Parks/public open space access/availability  *Perceived [unnamed questionnaire; reliable]:*  5. Number of Recreational facilities 🡪 Recreational facilities access/availability 6. Safe from traffic 🡪 Traffic/pedestrian safety  *Notes:* Number of street intersections not reported because they were reported in Li et al. (2005b). In addition, access/proximity to recreational facilities access/availability, and safe to walk not reported because they were reported in Nagel et al. (2008). | Area of green and open space*access to recreational facilities  Number of street intersections*safe from traffic | Multilevel linear regression accounting for clustering | *No significant moderating effects between* area of green and open space for recreation*access to recreational facilities: b=-0.022 (95% CI=-0.060; 0.016) (SE=0.019), p=.13  Significant moderating effects for number of street intersections*safe from traffic: b=0.019 (95% CI=0.007; 0.016) (SE=0.032), p=.05  ***Main effects with TotalWalking(score):*** Objective environment: Neighbourhood level: 1. Employment density—b (unstandardised) (95% CIs): b=0.187 (0.061; 0.313) (SE=0.06), p=.05 (Urbanisation **+**) 2. Household density: b=0.047 (0.001; 0.094) (SE=0.024), p=.05 (Residential density **+**) 3. Area of green and open space for recreation: b=0.074 (0.009; 0.140) (SE=0.033), p=.05 (Parks/public open space access/availability **+**)  Resident level: 4. Area of green and open space for recreation: b=-0.056 (-0.103; -0.009) (SE=0.024), p=.05 (Parks/public open space access/availability **0**) Perceived environment: 5. Number of Recreational facilities: b=0.077 (0.052; 0.103) (SE=0.013), p=.001 (Recreational facilities access/availability **+**) 6. Safe from traffic: b=0.152 (-0.016; 0.321) (SE=0.086), p=.06 (Traffic/pedestrian safety **0**) | *Resident and neighbourhood like a buffer, as resident level findings were ‘aggregated up’ to determine neighbourhood level findings.* Table 2. Findings regarding ‘Access/proximity to Recreational facilities access/availability’ and ‘Safe to walk’ were not reported because they are reported in Li et al. (2005b) adjusted for some covariates (e.g., education). Findings regarding ‘Number of street intersections’ was not reported because they are reported in Nagel et al. (2008) adjusted for age, sex, education etc. In addition, the outcome variable was assessed using a validated measure. |
| 82 | SHAPE Li et al., 2005b [63] | N=303 (urban) 65+ years 64% female 31% response rate Community-dwellers Portland, USA | Cross-sectional and longitudinal Cluster: purposive Individuals: random Stratification: population density, residential density, commercial density (urbanisation) and income Neighbourhood definition: street level (objective); 0.5mile buffer (objective); not defined (perceived) | Education, health status, household income, walking self-efficacy | *Self-report [SHAPE questionnaire; reliable]:* Total neighbourhood walking (score) 🡪 Total walking *TotalWalking(score)*  Total neighbourhood walking—rate of change (score) 🡪 Total walking *TotalWalking(score—rate of change)* | *Perceived [unnamed questionnaire; reliable]:*  Initial status: 1. Safe to walk 🡪 Crime/personal safety 2. Access to Recreational facilities 🡪 Recreational facilities access/availability   Rate of change: 1. Safe to walk 🡪 Crime/personal safety 2. Access/proximity to Recreational facilities access/availability 🡪 Recreational facilities access/availability | None | Multilevel growth model | ***Main effects with TotalWalking(score):*** Cross-sectional: 1. Safe to walk: b (unstandardised)=0.25 (SE=0.56), t=0.45, p>.05 (Crime/personal safety **0**) 2. Access to Recreational facilities: b (unstandardised)=-0.16 (SE=0.35), t=-0.46 p>.05  (Recreational facilities access/availability **0**)  ***Main effects with TotalWalking(score—rate of change):*** Longitudinal: 1. Safe to walk: b (unstandardised)=0.44 (SE=0.15), t=2.92, p<.05 (Crime/personal safety **+**) 2. Access/proximity to Recreational facilities access/availability : b (unstandardised)=0.30 (SE=0.10), t=2.96, p<.05 (Recreational facilities access/availability **+**) | Table 2. |
| 83 | SHAPE Michael et al., 2006 [32] | N=105 (urban) 65+ years 67% female 31% response rate Community-dwellers Portland, USA | Cross-sectional  Cluster: purposive Individuals: random Stratification: walkability Neighbourhood definition: street level (objective); “near home” (perceived) | Age, sex, education, race, income | *Self-report [SHAPE questionnaire; reliable]:*  Total neighbourhood walking (low walkers: not at all, a little bit, moderate amount; high walkers: quite a bit, a great deal; Yes/No) 🡪 Total walking *TotalWalking(High walker; Yes/No)* | *Objective [ArcGIS, Regional Land Information System data; unvalidated];*  1. Graffiti and vandalism 🡪 Greenery and aesthetically pleasing scenery 2. Sidewalk obstruction 🡪 No physical barriers to walking 3. Presence of parks 🡪 Parks/public open space access/availability 4. Presence of malls 🡪 Shops/commercial access/availability 5. Presence of trails 🡪 Parks/public open space access/availability *Perceived [unnamed questionnaire; reliable]:*  6. Graffiti and vandalism 🡪 Greenery and aesthetically pleasing scenery 7. Sidewalk obstruction 🡪 No physical barriers to walking 8. Presence of parks 🡪 Parks/public open space access/availability 9. Presence of malls 🡪 Shops/commercial access/availability 10. Presence of trails 🡪 Parks/public open space access/availability | None | Multivariate logistic regression  *Note. Several [unnamed] variables transformed.* | ***Main effects with TotalWalking(High walker; Yes/No):*** Objective environment: 1. Graffiti and vandalism: OR=0.57, p=.28  (Greenery and aesthetically pleasing scenery **0**) 2. Sidewalk obstruction: OR=not reported, p>.05 (No physical barriers to walking **0**) 3. Presence of parks: OR=not reported, p>.05 (Parks/public open space access/availability **0**) 4. Presence of malls: OR=4.12, p=.147 (Shops/commercial access/availability **0**) 5. Presence of trails: OR=not reported, p>.05 (Parks/public open space access/availability **0**)  Perceived environment: 6. Graffiti and vandalism: OR=not reported, p>.05 (Greenery and aesthetically pleasing scenery **0**) 7. Sidewalk obstruction: OR=not reported, p>.05 (No physical barriers to walking **0**) 8. Presence of parks: OR=not reported, p>.05 (Parks/public open space access/availability **0**) 9. Presence of malls: OR=2.10, p=.108 (Shops/commercial access/availability **0**) 10. Presence of trails: OR=not reported, p>.05 (Parks/public open space access/availability **0**) | In-text, below Table 2. |
| 84 | SHAPE Nagel et al., 2008 [93] | N=426 (urban) 65+ years 70% female Response rate not reported Community-dwellers Portland, USA | Cross-sectional  Cluster: purposive Individuals: random Stratification: none Neighbourhood definition: 0.25mile and 0.5mile buffers | Age, sex, education, race, income, health status, walking self-efficacy | *Self-report [Yale Physical Activity Scale questionnaire; validated]:*  Total walking (mins/wk) 🡪 Total walking *TotalWalking(mins/wk)*  Likelihood of walking (0 mins/wk vs.>0 mins/wk; Yes/No) 🡪 Total walking *TotalWalking(likelihood>0 mins/wk; Yes/No)*  Brisk walking time (mins/wk) 🡪 Total walking *BriskTotalWalking(mins/wk)* | *Objective [ArcGIS, Regional Land Information System database; unvalidated]:*  1. % of high volume streets 🡪 Traffic/pedestrian safety  2. % of medium-volume streets 🡪 Traffic/pedestrian safety  3. % of low-volume streets 🡪 Traffic/pedestrian safety 4. Sidewalk coverage 🡪 Walk-friendly infrastructure 5. Number of intersections 🡪 Street connectivity 6. Number of bus lines 🡪 Public transport access/availability 7. Number of commercial establishments 🡪 Shops/commercial access/availability 8. Number of select establishments 🡪 Destinations/services (overall/unspecific) access/availability 9. Distance to nearest park 🡪 Parks/public open space access/availability  10. Neighbourhood-level problems 🡪 Crime/personal safety  11. Neighbourhood-level walking safety 🡪 Crime/personal safety | None | Multilevel linear and logistic regression models accounting for clustering. | ***Main effects with TotalWalking(mins/wk):***  1. High volume [traffic] streets:  *0.25 mile:* b=1.27 (SE=0.5), p<.05 *0.5 mile:* b=1.83 (SE=0.61), p<.001 (Traffic/pedestrian safety **-*0.5; -*0.5**) 2. Medium volume [traffic] streets:  *0.25 mile:* b=0.73 (SE=0.6), p>.05 *0.5 mile:* b=1.15 (SE=0.89), p>.05 (Traffic/pedestrian safety **0*0.5; 0*0.5**) 3. Low volume [traffic] streets:  *0.25 mile:* b=-1.36 (SE=0.4), p<.001 *0.5 mile:* b=-1.93 (SE=0.48), p<.001 (Traffic/pedestrian safety **-*0.5; -*0.5**) 4. Sidewalk coverage: *0.25 mile:* b=0.16 (SE=0.17), p>.05 *0.5 mile:* b=0.32 (SE=0.21), p>.05 (Walk-friendly infrastructure **0*0.5; 0*0.5**) 5. Number of intersections: *0.25 mile:* b=0.36 (SE=0.27), p>.05 (Street connectivity **0*0.5**) *0.5 mile:* b=0.20 (SE=0.08), p<.05 (Street connectivity **+*0.5**) 6. Number of bus lines: *0.25 mile:* b=2.22 (SE=1.21), p>.05 (Public transport access/availability **0*0.5**) *0.5 mile:* b=1.86 (SE=0.70), p<.05 (Public transport access/availability **+*0.5**) 7. Number of commercial establishments: *0.25 mile:* b=0.25 (SE=0.06), p<.001 *0.5 mile:* b=0.07 (SE=0.02), p<.001 (Shops/commercial access/availability **+*0.5; +*0.5**) 8. Number of select establishments: *0.25 mile:* b=0.68 (SE=0.26), p<.05 (Destinations/services (overall/unspecific) access/availability **+*0.5**) *0.5 mile:* b=0.34 (SE=0.09), p<.001 (Destinations/services (overall/unspecific) access/availability **0*0.5**) 9. Distance to nearest park: b=-0.01 (SE=0.01), p<.05 (Parks/public open space access/availability **+**) 10. Neighbourhood-level problems:  b=-4.11, p=.050 (Crime/personal safety **+**)  11. Neighbourhood-level walking safety: b=not reported, p>.05 (Crime/personal safety **0**)  ***Main effects with TotalWalking(likelihood>0 mins/wk; Yes/No):*** 1. High volume streets:  *0.25 mile:* OR=not reported, p>.05 *0.5 mile:* OR=not reported, p>.05 (Traffic/pedestrian safety **0*0.5; 0*0.5**) 2. Medium volume streets:  *0.25 mile:* OR=not reported, p>.05 *0.5 mile:* OR=not reported, p>.05 (Traffic/pedestrian safety **0*0.5; 0*0.5**) 3. Low volume streets: *0.25 mile:* OR=not reported, p>.05 *0.5 mile:* OR=not reported, p>.05 (Traffic/pedestrian safety **0*0.5; 0*0.5**) 4. Sidewalk coverage: *0.25 mile:* OR=not reported, p>.05 *0.5 mile:* OR=not reported, p>.05 (Walk-friendly infrastructure **0*0.5; 0*0.5**) 5. Number of intersections: *0.25 mile:* OR=not reported), p>.05 *0.5 mile:* OR=not reported, p>.05 (Street connectivity **0*0.5; 0*0.5**) 6. Number of bus lines: *0.25 mile:* OR=not reported, p>.05 *0.5 mile:* OR=not reported, p>.05 (Public transport access/availability **0*0.5; 0*0.5**) 7. Number of commercial establishments: *0.25 mile:* OR=not reported, p>.05 *0.5 mile:* OR=not reported, p>.05 (Shops/commercial access/availability **0*0.5; 0*0.5**) 8. Number of select establishments: *0.25 mile:* OR=not reported, p>.05 *0.5 mile:* OR=not reported, p>.05 (Destinations/services (overall/unspecific) access/availability **0*0.5; 0*0.5**) 9. Distance to nearest park: OR=not reported, p>.05 (Parks/public space access/availability **0**) 10. Neighbourhood-level problems:  b=not reported, p>.05 (Crime/personal safety **0**)  11. Neighbourhood-level walking safety: b=not reported, p>.05 (Crime/personal safety **0**)  ***Main effects with BriskTotalWalking(mins/wk):*** 1. High volume [traffic] streets:  *0.25 mile:* b=1.11, p=.028 (Traffic/pedestrian safety **-*0.5**) *0.5 mile:* b=not reported, p>.05 (Traffic/pedestrian safety **0*0.5**) 2. Medium volume [traffic] streets:  *0.25 mile:* b=not reported, p>.05 *0.5 mile:* b=not reported, p>.05 (Traffic/pedestrian safety **0*0.5; 0*0.5**) 3. Low volume [traffic] streets:  *0.25 mile:* b=-0.85, p=0.42 (Traffic/pedestrian safety **-*0.5**) *0.5 mile:* b=not reported, p>.05 (Traffic/pedestrian safety **0*0.5**) 4. Sidewalk coverage: *0.25 mile:* b=not reported, p>.05 *0.5 mile:* b=not reported, p>.05 (Walk-friendly infrastructure **0*0.5; 0*0.5**) 5. Number of intersections: *0.25 mile:* b=not reported, p>.05 *0.5 mile:* b=not reported, p>.05  (Street connectivity **0*0.5; 0*0.5**) 6. Number of bus lines: *0.25 mile:* b=not reported, p>.05 *0.5 mile:* b=not reported, p>.05  (Public transport access/availability **0*0.5; 0*0.5**) 7. Number of commercial establishments: *0.25 mile:* b=not reported, p>.05 (Shops/commercial access/availability **0*0.5**) *0.5 mile:* b=0.04, p=.016 (Shops/commercial access/availability **+*0.5**) 8. Number of select establishments: *0.25 mile:* b=not reported, p>.05 (Destinations/services (overall/unspecific) access/availability **0*0.5**) *0.5 mile:* b=0.20, p=.033 (Destinations/services (overall/unspecific) access/availability **+*0.5**) 9. Distance to nearest park:  b=-0.02, p=.032 (Parks/public open space access/availability **+**) 10. Neighbourhood-level problems:  b=not reported, p>.05 (Crime/personal safety **0**)  11. Neighbourhood-level walking safety: b=not reported, p>.05 (Crime/personal safety **0**) | Buffer effects. Table 4—Model 2. *Note. Total walking (n=426), brisk walking (n=275)* |
| 85 | SMARTRAQ (Strategies for Metropolitan Atlanta’s Regional Transportation and Air Quality study) Frank et al. (2010) [130] | N=1970 (urban) 65+ years 56% female 30.4% response rate Community-dwellers Atlanta, USA | Cross-sectional Cluster: purposive Individuals: purposive Stratification: residential density (urbanisation), household size and income Neighbourhood definition: 1km buffer | Age, sex, education, living with others, income, car ownership, ethnicity | *Self-report [travel survey; unvalidated]:*  MVPA (150+ mins/wk; Yes/No) 🡪 Total MVPA *TotalMVPA(150+ mins/wk; Yes/No)* Walked at least once in 2 days 🡪 Total walking *TotalWalking(Once/2d; Yes/No)* | *Objective [County Level Tax Assessor’s data, regional land use data, street network data, census data; unvalidated]:*  1. Walkability – categorised as: low, medium and high walkability | None | Multilevel logistic regression | ***Main effect with TotalMVPA(150+ mins/wk; Yes/No):*** 1. Walkability (Ref: Low)—OR (95% CIs): Medium OR=1.13 (0.82; 1.54), High OR=1.08 (0.78; 1.48) (Walkability **0**)  ***Main effect with TotalWalking(Once/2d; Yes/No):*** 1. Walkability (Ref: Low)—OR (95% CIs): Medium OR=1.10 (0.59; 2.07), High OR=2.02 (1.13; 3.64) (Walkability **+**) | Table 3. |
| 86 | SNQLS (Senior Neighborhood Quality of Life Study) Kerr et al., 2011 [43] | N=147 (urban) Mean age: 80 years 71% female Response rate not reported Retirement village dwellers Baltimore, Palo Alto, San Diego, & Seattle USA | Validation study Cluster: purposive Individuals: convenience Stratification: participants/site (≤n=30) (none) Neighbourhood definition: retirement village | Clustering | *Objective [ActiGraph—validated; Freedson MVPA cutoff point—validated]:*  MVPA 🡪 Total MVPA *TotalMVPAFreedson (mins/d)* | *Objective [Audit of Physical Activity Resources for Seniors; validated]:*  1. Grassy area 🡪 Parks/public open space access/availability 2. Path intersections 🡪 Street connectivity 3. Path with moderate slope 🡪 No physical barriers to walking 4. Water features 🡪 Greenery and aesthetically pleasing scenery 5. Art/sculptures 🡪 Greenery and aesthetically pleasing scenery 6. Pleasant views 🡪 Greenery and aesthetically pleasing scenery 7. Hazardous path sections 🡪 No physical barriers to walking 8. Obstructions on path and lighting 🡪 No physical barriers to walking 9. ≤1 exterior light 🡪 Crime/personal safety 10. Putting green 🡪 Recreational facilities access/availability  11. Lawn bowling 🡪 Recreational facilities access/availability  12. Basketball hoop 🡪 Recreational facilities access/availability  13. Exercise stations 🡪 Recreational facilities access/availability  14. Tennis court 🡪 Recreational facilities access/availability  15. Swimming pool 🡪 Recreational facilities access/availability  16. Combined fitness aerobic classroom 🡪 Recreational facilities access/availability  17. Indoor pool 🡪 Recreational facilities access/availability  18. Exercise equipment not in designated room 🡪 Recreational facilities access/availability  19. Open lounges 🡪 Social recreational facilities access/availability  20. Dining room 🡪 Social recreational facilities access/availability  21. Kitchen 🡪 Social recreational facilities access/availability  22. Open areas/courtyards/patios 🡪 Parks/public open space access/availability 23. Bank 🡪 Government/finance services access/availability 24. Pharmacy 🡪 Shops/commercial access/availability 25. Hairdresser/beautician 🡪 Other service/institution access/availability 26. Chapel/religious services 🡪 Religious institution access/availability 27. Mail room 🡪 Other service/institution access/availability 28. Café/cafeteria 🡪 Food outlets access/availability 29. Gift shop 🡪 Shops/commercial access/availability 30. Snack shop 🡪 Shops/commercial access/availability 31. Laundry 🡪 Other service/institution access/availability 32. Medical/dental clinic 🡪 Health and aged care access/availability 33. Length of longest walking path 🡪 Walk-friendly infrastructure | None | Bivariate and Spearman correlations; mixed effects regression models adjusted for clustering. | ***Main effects with TotalMVPAFreedson(mins/d):*** 1. Grassy area: r=not reported, p>.05 (Parks/public open space access/availability **0**) 2. Path intersections: r=.165, p<.05 (Street connectivity **+**) 3. Path with moderate slope: r=.193, p<.05 (No physical barriers to walking **+**) 4. Water features: r=not reported, p>.05 (Greenery and aesthetically pleasing scenery **0**) 5. Art/sculptures: r=not reported, p>.05 (Greenery and aesthetically pleasing scenery **0**) 6. Pleasant views: r=not reported, p>.05 (Greenery and aesthetically pleasing scenery **0**) 7. Hazardous path sections: r=not reported, p>.05 (No physical barriers to walking **0**) 8. Obstructions on path: r=.165, p<.05 (No physical barriers to walking **-**) 9. ≤1 exterior light: r=.165, p<.05 (Crime/personal safety **+**) 10. Putting green: r=.138, p>.05 (Recreational facilities access/availability **0**) 11. Lawn bowling: r=.138, p>.05 (Recreational facilities access/availability **0**) 12. Basketball hoop: r=.138, p>.05 (Recreational facilities access/availability **0**) 13. Exercise stations: r=.206, p<.05 (Recreational facilities access/availability **+**) 14. Tennis court: r=not reported, p>.05 (Recreational facilities access/availability **+**) 15. Swimming pool: r=.138 p>.05 (Recreational facilities access/availability **0**) 16. Combined fitness aerobic classroom: r=.234 p<.01 (Recreational facilities access/availability **+**) 17. Indoor pool: r=not reported, p>.05  (Recreational facilities access/availability **0**) 18. Exercise equipment not in designated room: r=not reported, p>.05 (Recreational facilities access/availability **0**) 19. Open lounges: r=not reported, p>.05 (Social Recreational facilities access/availability **0**) 20. Dining room: r=-.103, p>.05 (Social recreational facilities access/availability **0**) 21. Kitchen: r=.228, p<.01 (Social recreational facilities access/availability **+**) 22. Open areas/courtyards/patios: r=not reported, p>.05 (Parks/public open space access/availability **0**) 23. Bank: r=not reported, p>.05 (Government/finance access/availability **0**) 24. Pharmacy: r=not reported, p>.05 (Shops/commercial access/availability **0**) 25. Hairdresser/beautician: r=-.086, p>.05 (Other service/institution access/availability **0**) 26. Chapel/religious services: r=not reported, p>.05 (Religious institution access/availability **0**) 27. Mail room: r=not reported, p>.05 (Other service/institution access/availability **0**) 28. Café/cafeteria: r=not reported, p>.05 (Food outlets access/availability **0**) 29. Gift shop: r=.008, p>.05 (Shops/commercial access/availability **0**) 30. Snack shop: r=not reported, p>.05 (Shops/commercial access/availability **0**) 31. Laundry: r=-.138, p>.05 (Other service/institution access/availability **0**) 32. Medical/dental clinic: r=not reported, p>.05 (Health and aged care access/availability **0**) 33. Length of longest walking path: r=.176, p<.05 (Walk-friendly infrastructure **+**) | Table 4. |
| 87 | SNQLS  Carlson et al., 2012 [44] | N=687 (urban)  66+ years; 53% women  21.4% response rate  Community dwellers  Seattle and Baltimore, USA | Cross-sectional  Cluster: purposive  Individuals: random  Stratification: walkability and SES  Neighbourhood definition: 500m street-network buffer (objective) and 15-20mins walk from home (perceived) | Age, sex, education, marital status, moths at address, number of people in the household, number of vehicles per adult | *Objective [ActiGraph accelerometer; validated; Freedson cutoff point; validated]:*  Total MVPA (mins/wk) 🡪 Total MVPA *TotalMVPAFreedson(mins/wk)* | *Objective [data from county-level tax assessor; GIS walkability index; unvalidated]:*  1. Parks and recreation 🡪 Parks/open space access/availability  *Perceived [NEWS questionnaire; validated]:*  2. Aesthetics 🡪 Greenery and aesthetically pleasing scenery  3. Walking facilities 🡪 Walk-friendly infrastructure  *Note:* Walkability not extracted as included in Bracy et al. with more appropriate statistical analyses and moderating effects yielding same-direction associations across values of moderator. | Social support,  Self-efficacy,  Barriers  n average (mean)=488  n high (+1SD)=115  n low (-1SD)=115 | General mixed models accounting for clustering | *Moderating effects:*  Walkability*social support p=.003  Stronger positive effects in those with higher social support  Walkability*self-efficacy p=.512  Nil effects in those with higher self-efficacy  Walkability*barriers p>.376  Nil effects in those with lower levels of barriers  ***Main and moderated effects with TotalMVPAFreedson(mins/wk):***  *Objective:*  1. Parks and recreation—b (95% CIs):  b=7.6 (-10.9; 26.0), p>.05 (Parks/public open space access/availability **0**)  *Perceived:*  2. Aesthetics:  b=3.0 (-5.3; 11.3), p>.05 (Greenery and aesthetically pleasing scenery **+*0.5; -*0.5**—interaction with barriers)  3. Walking facilities:  b=-3.5 (-11.9; 4.8), p>.05 (Walk-friendly infrastructure **0**) | Moderating effects. Walkability not reported here as it is included in Bracy et al. with more appropriate statistical analyses and moderating effects yielding same-direction associations across values of moderators. |
| 88 | SNQLS  Bracy et al., 2014 [46] | N=718 (urban)  66+ years; 53% women  21.4% response rate  Community dwellers  Seattle and Baltimore, USA | Cross-sectional  Cluster: purposive  Individuals: random  Stratification: walkability and SES  Neighbourhood definition: 500m street-network buffer (objective) and 15-20mins walk from home (perceived) | Age, sex, education, marital status, moths at address, number of people in the household, number of vehicles per adult | *Objective [ActiGraph accelerometer; validated; Freedson MVPA cutoff point; validated]:*  Total MVPA (mins/d) 🡪 Total MVPA *TotalMVPAFreedson(mins/wk)* | *Objective [Walkability index; validated]:*  1. Walkability 🡪 Walkability  *Perceived [NEWS questionnaire; validated]:*  2. Parks 🡪 Parks/public open space access/availability 3. Recreational facilities 🡪 Recreational facilities access/availability  *Notes:* Traffic safety, pedestrian safety, and crime safety not reported as included in Carlson et al. (2014). | Traffic safety, Pedestrian safety, Crime safety  n average (mean)=488  n high (+1SD)=115  n low (-1SD)=115 | General mixed models accounting for clustering; log transformed transport walking | *No significant moderating effects.*  ***Main and moderated effects with TotalMVPAFreedson(mins/wk):***  Objective environment:  1. Walkability—b (95% CIs):  b=6.03 (2.71; 9.34), p<.05 (Walkability **+**)  Perceived environment:  2. Parks: b=33.01 (15.76; 50.26), p<.05 (Parks/public open space access/availability **+**) 3. Recreational facilities: b=10.20 (-7.57; 27.98), p>.05 (Recreational facilities access/availability **0**) | There are multiple measures per environmental construct that need to be summed. Traffic, pedestrian and crime safety not reported as reported in Carlson et al. (2014). |
| 89 | SNQLS  Cain et al., 2014 [47] | N=367 (urban) Mean age: 75.0 years 51% female 21.4% response rate  Community dwellers  Seattle and Baltimore, USA | Cross-sectional Cluster: purposive Individuals: random Stratification: walkability and SES Neighbourhood definition: 0.25mile buffer | Age, sex, education, race, physical functioning, clustering of participants within block groups | *Objective [ActiGraph accelerometer—validated; Freedson MVPA cutoff point—validated]:*  Total MVPA 🡪 Total MVPAFreedson (mins/d) *TotalMVPAFreedson(mins/d)* | *Objective [Microscale Audit of Pedestrian Streetscapes; unvalidated]:*  1. Residential mix 🡪 Residential density 2. Shops 🡪 Shops/commercial access/availability 3. Restaurant 🡪 Food outlets access/availability 4. Institutional-service 🡪 Government/finance services access/availability 5. Government-service 🡪 Government/finance services access/availability 6. Public recreation 🡪 Recreational facilities access/availability  7. Private recreation 🡪 Recreational facilities access/availability  8. Transit stops 🡪 Public transport access/availability 9. Aesthetics and social characteristics 🡪 Greenery and aesthetically pleasing scenery 10. Curb quality 🡪 Pavement/footpath quality 11. Crossings/intersections / Impediments 🡪 No physical barriers to walking 12. Street segments / Buffer 🡪 Traffic/pedestrian safety 13. Bike infrastructure 🡪 Cycle-friendly infrastructure 14. Trees 🡪 Greenery and aesthetically pleasing scenery 15. Building aesthetics/design 🡪 Greenery and aesthetically pleasing scenery 16. Sidewalk 🡪 Walk-friendly infrastructure 17. Sidewalk obstructions/hazards 🡪 No physical barriers to walking 18. Slope 🡪 No physical barriers to walking 19. Intersection control 🡪 Traffic/pedestrian safety | None | Mixed linear regression | ***Main effects with TotalMVPAFreedson(mins/d):*** 1. Residential mix: t=2.269, p≤.05 (Residential density **+**) 2. Shops: t=1.312, p>.05  (Shops/commercial access/availability **0**) 3. Restaurant-entertainment: t=1.039, p>.05 (Food outlets access/availability **0**) 4. Institutional-service: t=0.421, p>.05 (Government/finance access/availability **0**) 5. Government-service: t=-0.875, p>.05 (Government/finance access/availability **0**) 6. Public recreation: t=-0.278, p>.05 (Recreational facilities access/availability **0**) 7. Private recreation: t=-1.216, p>.05 (Recreational facilities access/availability **0**) 8. Transit stops: t=0.969, p>.05 (Public transport access/availability **0**) 9. Aesthetics and social characteristics: t=0.057, p>.05 (Greenery and aesthetically pleasing scenery **0**) 10. Curb quality: t=2.662, p≤.01 (Pavement/footpath quality **+**) 11. Crossings/intersections / Impediments: t=-2.016, p ≤ .05 (No physical barriers to walking **+**) 12. Street segments / Buffer: t=1.007, p>.05 (Traffic/pedestrian safety **0**) 13. Bike infrastructure: t=0.292, p>.05 (Cycle-friendly infrastructure **0**) 14. Trees: t=1.450, p>.05 (Greenery and aesthetically pleasing scenery **0**) 15. Building aesthetics/design: t=0.400, p>.05 (Greenery and aesthetically pleasing scenery **0**) 16. Sidewalk: t=1.816, p>.05 (Walk-friendly infrastructure **0**) 17. Sidewalk obstruction/hazards: t=-1.814, p>.05 (No physical barriers to walking **0**) 18. Slope: t=-1.442, p>.05 (No physical barriers to walking **0**) 19. Intersection control: t=3.581, p≤.001 (Traffic/pedestrian safety **+**) | Table 5–Unadjusted. |
| 90 | SNQLS  Carlson et al., 2014 [45] | N=718 (urban)  66+ years; 53% women  21.4% response rate  Community dwellers  Seattle and Baltimore, USA | Cross-sectional  Cluster: purposive  Individuals: random  Stratification: walkability and SES  Neighbourhood definition: 15-20mins walk from home | Age, sex, education, marital status, moths at address, number of people in the household, number of vehicles per adult, walkability | *Objective [ActiGraph accelerometer; validated; Freedson cutoff point; validated]:* Total MVPA (mins/wk) 🡪 Total MVPA *TotalMVPA(mins/wk)* | *Perceived [NEWS questionnaire; validated]:*  1. Crime safety 🡪 Crime/personal safety 2. Pedestrian safety 🡪 Traffic/pedestrian safety 3. Traffic safety 🡪 Traffic/pedestrian safety | Gender; race/ethnicity; education; neighbourhood income  n average (mean)=488  n high (+1SD)=115  n low (-1SD)=115 | General mixed models accounting for clustering. | *Significant moderating effects for:* Crime safety*gender, p>.05 Crime safety*race/ethnicity, p>.05 Crime safety*education, p>.05 Crime safety*neighbourhood income, p>.05 Pedestrian safety*gender, p>.05 Pedestrian safety*race/ethnicity, p>.05 Pedestrian safety*education, p>.05 Pedestrian safety*neighbourhood income, p<.10 Traffic safety*gender, p>.05 Traffic safety*race/ethnicity, p>.05 Traffic safety*education, p<.10 Traffic safety*neighbourhood income, p>.05  ***Main effects with TotalMVPA(mins/wk):***  1. Crime safety—b (95% CIs):  b=0.4 (-9.4; 10.2), p>.05 (Crime/personal safety **0**)  2. Pedestrian safety:  b=5.0 (-4.6; 14.7), p>.05 (Traffic/pedestrian safety **0**) 3. Traffic safety: b=2.0 (-7.3; 11.3), p>.05 (Traffic/pedestrian safety **0**) | Table 4. |
| 91 | SNQLS Ding et al., 2014 [48] | N=861 (urban)  66+ years; 56% women  21.4% response rate  Community and retirement village dwellers  Seattle and Baltimore, USA | Cross-sectional with two assessments  Cluster: purposive  Individuals: random  Stratification: walkability and SES  Neighbourhood definition: 500m street-network buffer (objective) and 15-20mins walk from home (perceived) | Age, sex, education, ethnicity, study site, marital status, number of people in household, living situation, length of time in current address, medical conditions, mobility impairment | *Objective [ActiGraph accelerometer; validated; Freedson MVPA cutoff point: validated]:*  Counts/min 🡪 Total PA *TotalPA(cpm)*  MVPA (mins/d) 🡪 Total MVPA *TotalMVPAFreedson(mins/d)* | *Objective [GIS index; validated]:*  1. Walkability 🡪 Walkability 2. Parks and Recreational facilities access/availability (categorized) 🡪 Parks/public open space access/availability  *Perceived [NEWS questionnaire; validated]:*  3. Residential density 🡪 Residential density  4. Land use mix—access 🡪 Destinations/services (overall/unspecific) access/availability  5. Land use mix—diversity 🡪 Land-use mix—destination diversity  6. Street connectivity 🡪 Street connectivity  7. Walking or cycling infrastructures 🡪 Cycle/walk-friendly infrastructure  8. Neighbourhood aesthetics 🡪 Greenery and aesthetically pleasing scenery  9. Traffic safety 🡪 Traffic/pedestrian safety  10. Pedestrian safety structures 🡪 Traffic/pedestrian safety  11. Transit access 🡪 Public transport access/availability  12. Personal safety 🡪 Crime/personal safety | Driving status:  Driving (n=712),  Non-driving (n=149) | Mixed linear regression models | *No significant moderating effects.*  ***Main effects with TotalPA(cpm):*** Objective environment: 1. Walkability—b (95% CIs): *Drivers:* b=3.23 (0.61; 5.82), p<.05 *Non-drivers:* b=1.09 (-1.92; 4.10), p>.05 (Walkability **+*1**) 2. Parks and Recreational facilities access/availability : *Drivers:* 1 vs. 0 (Ref): b=10.80 (-6.66; 28.28), p>.05; 2+ vs. 0 (Ref): b=11.98 (-3.79; 27.20), p>.05 *Non-drivers:* 1 vs. 0 (Ref): b=14.76 (-22.84; 52.37), p>.05; 2+ vs. 0 (Ref): b=27.84 (-7.11; 62.79), p>.05 (Parks/public open space access/availability **0*1**)  Perceived environment: 3. Residential density: *Drivers:* b=1.49 (-7.10; 10.07), p>.05 *Non-drivers:* b=0.28 (-8.08; 8.64), p>.05 (Residential density **0*1**) 4. Land-use mix—access: *Drivers:* b=9.67 (-1.42; 20.76), p<.10 *Non-drivers:* b=9.15 (-8.02; 26.32), p>.05 (Destinations/services (overall/unspecific) access/availability **0*1**) 5. Land-use mix–diversity: *Drivers:* b=11.24 (2.88; 19.59), p<.01 *Non-drivers:* b=9.20 (-3.31; 21.72), p>.05 (Land-use mix—destination diversity **+*1**) 6. Street connectivity: *Drivers:* b=2.58 (-6.84; 12.00), p>.05 *Non-drivers:* b=-9.77 (-25.62; 6.07), p>.05 (Street connectivity **0*1**) 7. Walking or cycling infrastructures: *Drivers:* b=0.85 (-6.87; 8.58), p>.05 *Non-drivers:* b=0.68 (-13.36; 14.71), p>.05 (Cycle/Walk-friendly infrastructure **0*1**) 8. Neighbourhood aesthetics: *Drivers:* b=5.14 (-4.92; 15.21), p>.05 *Non-drivers:* b=-5.58 (-20.29; 9.12), p>.05 (Greenery and aesthetically pleasing scenery **0*1**) 9. Traffic safety: *Drivers:* b=-0.01 (-0.48; 0.50), p>.05 *Non-drivers:* b=-13.72 (-29.29; 1.72), p>.05 (Traffic/pedestrian safety **0*1**) 10. Pedestrian safety structures: *Drivers:* b=-3.01 (-17.34; 11.33), p>.05 *Non-drivers:* b=0.94 (-20.94; 22.82), p>.05 (Traffic/pedestrian safety **0*1**) 11. Transit access: *Drivers:* b=3.04 (-3.53; 9.60), p>.05 *Non-drivers:* b=2.26 (-9.08; 13.60), p>.05 (Public transport **0*1**) 12. Personal safety: *Drivers:* b=2.03 (-9.48; 13.22), p>.05 *Non-drivers:* b=-10.84 (-27.78; 6.10), p>.05 (Crime/personal safety **0*1**)  ***Main effects with TotalMVPAFreedson(mins/d):*** Objective environment: 1. Walkability: *Drivers:* b=0.72 (0.27; 1.16), p<.01 *Non-drivers:* b=0.06 (-0.37; 0.49), p>.05 (Walkability **+*1**) 2. Parks and Recreational facilities access/availability : *Drivers:* 1 vs. 0 (Ref): b=1.09 (-1.92; 4.10), p>.05; 2+ vs. 0 (Ref): b=2.36 (-0.31; 5.03), p>.05 *Non-drivers:* 1 vs. 0 (Ref): b=-0.90 (-6.26; 4.46), p>.05; 2+ vs. 0 (Ref): b=2.52 (-2.74; 7.22), p>.05 (Parks/public open space access/availability **0*1**)  Perceived environment: 3. Residential density: *Drivers:* b=0.77 (-0.71; 1.69), p>.05 *Non-drivers:* b=0.46 (-0.76; 1.68), p>.05 (Residential density **0*1**) 4. Land-use mix—access: *Drivers:* b=1.98 (0.07; 3.89), p<.05 *Non-drivers:* b=1.60 (-0.84; 4.05), p>.05 (Destinations/services (overall/unspecific) access/availability **+*1**) 5. Land-use mix—diversity: *Drivers:* b=2.53 (1.09; 3.96), p<.01 *Non-drivers:* b=1.87 (0.12; 3.61), p<.05 (Land-use mix—destination diversity **+*1**) 6. Street connectivity: *Drivers:* b=-0.66 (-2.28; 0.96), p>.05 (Street connectivity **0*0.86**) *Non-drivers:* b=-2.45 (-4.68; -0.21), p<.05 (Street connectivity **-*0.14**) 7. Walking or cycling infrastructures: *Drivers:* b=0.39 (-0.94; 1.72), p>.05 *Non-drivers:* b=0.77 (-1.23; 2.76), p>.05 (Cycle/Walk-friendly infrastructure **0*1**) 8. Neighbourhood aesthetics: *Drivers:* b=0.27 (-1.46; 2.01), p>.05 *Non-drivers:* b=-0.31 (-2.41; 1.79), p>.05 (Greenery and aesthetically pleasing scenery **0*1**) 9. Traffic safety: *Drivers:* b=-0.01 (-1.63; 1.62), p>.05 *Non-drivers:* b=-1.24 (-3.45; 0.98), p>.05 (Traffic/pedestrian safety **0*1**) 10. Pedestrian safety structures: *Drivers:* b=0.78 (-1.68; 3.25), p>.05 *Non-drivers:* b=0.13 (-3.00; 3.24), p>.05 (Traffic/pedestrian safety **0*1**) 11. Transit access: *Drivers:* b=1.05 (-0.08; 2.17), p<.10 *Non-drivers:* b=0.92 (-0.69; 2.53), p>.05 (Public transport **0*1**) 12. Personal safety: *Drivers:* b=-0.20 (-2.16; 1.75), p>.05 *Non-drivers:* b=0.49 (-1.94; 2.91), p>.05 (Crime/personal safety **0*1**) | Moderating effects. *Note. Multiple measures per environmental construct need to be summed.*  Table 3. |
| 92 | TILDA (The Irish Longitudinal Study on Ageing) McKee et al., 2015 [147] | N=596 (mixed) 65+ years 53% female Response rate not reported Community-dwellers *Dublin City or County and other locations,* Ireland | Cross-sectional Cluster: purposive Individuals: random Stratification: age, SES and geography (urbanisation) Neighbourhood definition: townland cluster (≈500-1180 addresses) (other participant delineation) | Age, sex, education, employment status, loneliness, membership of a non-church club, type of house, disability, time spent sitting, grip test, often troubled with pain, BMI, long-term illness, self-rated vision, self-rated hearing, number of regular medications, fallen last year, anxiety, quality of life, depression, mini-mental state examination, self-rated memory | *Self-report [IPAQ; validated]:*  METs/wk 🡪 Total PA *TotalPA(METs/wk)* | *Perceived [unnamed questionnaire; unvalidated]:*  1. Location of home 🡪 Urbanisation 2. Physical state of buildings in area 🡪 Greenery and aesthetically pleasing scenery 3. Vandalism in area Aesthetics and greenery 4. Litter in area 🡪 Greenery and aesthetically pleasing scenery | None | Multivariate linear regression | ***Main effects with TotalPA(METs/wk):*** 1. Location of home—b (95% CIs): b=not reported, p>.001 (Urbanisation **0**) 2. Physical state of buildings in the area: b=-0.024 (-0.060; 0.015), p =.246 (Greenery and aesthetically pleasing scenery **0**) 3. Vandalism in area: b=not reported, p>.001 (Greenery and aesthetically pleasing scenery **0**) 4. Litter in area: b=not reported, p>.001 (Greenery and aesthetically pleasing scenery **0**) | *Note. Bonferroni adjustment (α=.001).* Table 2. |
| 93 | TILDA Murtagh et al., 2015 [152] | N=4892 (mixed) 60-75+ years 53% female Response rate not reported Community-dwellers *Dublin City or County and other locations,* Ireland | Cross-sectional Cluster: purposive Individuals: random Stratification: age, SES and geography (urbanisation) Neighbourhood definition: townland cluster (≈500-1180 addresses) (other participant delineation) | Age, education, SES, living status, having children, employment status, car ownership, perceived overall health, perceived emotional health, falls in last year, fear of falling, activity limited by illness, caring for grandchildren, attendance to an Education course | *Self-report [IPAQ; validated]:*  Total MVPA (<150 mins/wk; Yes/No) 🡪 Total MVPA *TotalMVPA(<150 mins/wk; Yes/No)* | *Perceived [unnamed questionnaire; unvalidated]:*  1. Location 🡪 Urbanisation | Sex:  Males( n≈2280), Females (n≈2612) | Multivariate logistic regression | ***Moderated effects with TotalMVPA(<150 mins/wk; Yes/No):*** 1. Location—OR (95% CIs):  *Females:* OR=0.87 (0.84; 0.89), p<.05 (Urbanisation **+*0.53**) *Males:* OR=1.24 (1.20; 1.28), p<.05 (Urbanisation **-*0.47**) | Moderating effects. Table 3. |
| 94 | UAB (University of Alabama at Birmingham) Study of Aging Hannon et al.,2012 [134] | N=433 (mixed) 65+ years 50% female 45.7% response rate (original study) Community-dwellers Jefferson, Tuscaloosa, Hale, Pickens, and Bibb, USA | Cross-sectional Cluster: purposive Individuals: random Stratification: ethnicity/race (African-Americans) and urbanisation Neighbourhood definition: county level (objective); not reported (perceived) | Age, sex, education, homeownership, occupancy, median home value, length of residency, income, marital status, comorbidities | *Self-report [unnamed questionnaire; unvalidated]:*  Neighbourhood walking (blocks/d) 🡪 Total walking *TotalWalking(blocks/d)* | *Objective [Alabama Rural Health Association data; unvalidated]:*  1. Residence 🡪 Urbanisation  *Perceived [unnamed questionnaire; unvalidated]:*  2. Fear of being robbed or attacked 🡪 Crime/personal safety | None | Hierarchical linear regression | ***Main effects with TotalWalking(blocks/d):*** Objective environment: 1. Residence: b=-0.193, p<.01 (Urbanisation **-**)  Perceived environment: 2. Fear of being robbed or attacked: b=0.064, p>.05 (Crime/personal safety **0**) | Table 3—model 4. |
| 95 | VoisiNuAge study  Gauvin et al., 2012 [62] | N=521 (urban) 67+ years 53% female Response rate not reported Community-dwellers Montreal and Laval, Canada | Longitudinal Cluster: none Individuals: random Stratification: age and sex Neighbourhood definition: participant delineation (perceived); not defined (objective) | Age, sex, education, country of birth, marital status, family income, housing ownership | *Self-report [1 question from PASE questionnaire; validated]:*  Frequency of walking change over time? (days) 🡪 Total walking *TotalWalking(d/wk)* | *Objective [MEGAPHONE database; validated]:*  1. Accessibility to 16 services and amenities 🡪 Destinations/services (overall/unspecific) access/availability  *Perceived [unnamed questionnaire; validated]:*  2. Quality of walking environment and transportation services 🡪 Walk-friendly infrastructure 3. Neighbourhood amenities and services—key resources for older adults 🡪 Destinations/services (overall/unspecific) access/availability 4. Neighbourhood amenities and services within 5 min 🡪 Destinations/services (overall/unspecific) access/availability 5. Availability of a bus stop or subway within 5 min walk 🡪 Public transport access/availability | None | Ordinal growth curve analysis. Spatial autocorrelation examined to account for clustering. | ***Main effects with TotalWalking(d/wk):*** Objective: 1. Accessibility to 16 services and amenities (Ref: Furthest)—OR (95% CIs): *Closest:* OR=2.52 (1.42; 4.49), p<.001 *Close:* OR=1.86 (1.09; 3.18), p<.05 *Far:* OR=1.15 (0.69; 1.94), p>.05 (Destinations/services (overall/unspecific) access/availability **+**)  Perceived: 2. Quality of walking environment (Ref: Very easy): *Very/somewhat difficult:* OR=0.34 (0.14; 0.86), p<.05 *Somewhat easy:* OR=0.71 (0.42; 1.21), p>.05 (Walk-friendly infrastructure **+**) 3. Neighbourhood amenities and services (Ref: Higher): *Lower:* OR=0.96 (0.61; 1.53), p>.05 *Average:* OR=1.12 (0.69; 1.78), p>.05 (Destinations/services (overall/unspecific) access/availability **0**) 4. Neighbourhood amenities and services within 5 min walk (Ref: Most): *Fewest:* OR=1.05 (0.52; 2.13), p>.05 *Few:* OR=1.22 (0.67; 2.23), p>.05 *Average:* OR=0.86 (0.42; 1.64), p>.05 *Many:* OR=0.88 (0.50; 1.55), p>.05 (Destinations/services (overall/unspecific) access/availability **0**) 5. Availability of a bus stop or subway within 5 min walk: OR=not reported, p>.05 (Public transport access/availability **0**) | Table 3 – apart from #5 public transport that was not presented in Table 3 but measured, evidenced in Table 2. |
| 96 | VoisiNuAge study  Julien et al., 2015 [138] | N=519 (urban) 68+ years 53% female 58.6% response rate Community-dwellers Montreal and Laval, Canada | Cross-sectional Cluster: purposive Individuals: random Stratification: age and sex Neighbourhood definition: postal code | Age, sex, education, income, owning a car, transit stop | *Self-report [1 question from PASE questionnaire; unvalidated]:*  Frequency of walking (days) 🡪 Total walking *TotalWalking(d/wk)* | *Objective [MEGAPHONE database; validated]:*  1. Accessibility to services and amenities conducive to social participation 🡪 Social recreational facilities access/availability | None | Multiple mediation analyses | ***Direct effects with TotalWalking(d/wk):*** 1. Access to/availability of amenities/services: b=0.43 (SE=0.14), p<.05  (Social recreational facilities access/availability **+**) | Fig. 1. |
| 97 | Walk the Talk Hirsch et al., 2016 [135] | N=77 (urban) 65+ years 66% female 8% response rate Community-dwellers Vancouver, Canada | Cross-sectional Cluster: purposive Individuals: random Stratification: income and walkability Neighbourhood definition: variable (6 different types of buffers) | Age, sex, education, vehicle access | *Objective [ActiGraph accelerometer—validated; using Freedson cutoff points for LPA and MVPA—validated]:*  Total PA (min/d) 🡪 Total PA *TotalPA(mins/d)* Steps/d 🡪 Total walking *TotalWalking(steps/d)* Meeting recommended daily steps 🡪 Total walking *Total Walking(10,000+ steps/d; Yes/No)* | *Objective [Environics Analytics business data, North American Industry Classification System, ArcGIS; unvalidated]:*  1. Destination diversity (number of different destinations; 1 additional destination type) 🡪 Land-use mix—destination diversity 2. Ambulatory health care services destination density (10% difference)🡪 Health and aged care access/availability 3. Banks/credit unions destination density (10% difference)🡪 Government/finance access/availability 4. Community centre/neighbourhood house destination density (10% difference)🡪 Social recreational access/availability 5. Convenience stores destination density (10% difference)🡪 Shops/commercial access/availability 6. Entertainment destination density (10% difference)🡪 Social recreational facilities access/availability  7. Food stores destination density (10% difference)🡪 Shops/commercial access/availability 8. Gym and fitness centres destination density (10% difference)🡪 Recreational facilities access/availability  9. Library destination density (10% difference)🡪 Education facilities access/availability 10. Malls destination density (10% difference)🡪 Shops/commercial access/availability 11. Museum destination density (10% difference)🡪 Education facilities access/availability 12. Nature/parks/botanical gardens destination density (10% difference)🡪 Parks/public open space access/availability 13. Pharmacies/drug stores/personal care destination density (10% difference)🡪 Health and aged care access/availability 14. Religious organisations destination density (10% difference)🡪 Religious institution access/availability 15. Restaurants destination density (10% difference)🡪 Food outlets access/availability 16. Retail shopping destination density (10% difference)🡪 Shops/commercial access/availability 17. Services destination density (10% difference)🡪 Destinations/services (overall/unspecific) access/availability 18. Total densities (10% difference) 🡪 Destinations/services (unspecified)access/availability | None | Multiple linear and logistic regression models, no adjustment for clustering. | ***Main effects with TotalPA(mins/d):*** 1. Diversity (1 additional destination type)—b (95% CIs): *400m:* b=-0.97 (5.47; 3.53), p>.05 *800m:* b=3.03 (1.79; 7.86), p>.05 *All-mode activity space:* b=-7.24 (-18.76; 4.28), p>.05 *Pedestrian and bicycling activity space:* b=3.97 (-1.17; 9.12), p>.05 *SD ellipse:* b=-2.80 (-8.90; 3.29), p>.05 *Min. convex polygon:* b=-1.12 (-8.80; 6.55), p>.05 (Land-use mix—destination diversity **0*0.166; 0*0.166; 0*0.166; 0*0.166; 0*0.166; 0*0.17**) 2. Ambulatory health care services: *400m:* b=0.02 (-0.04; 0.07), p>.05 *800m:* b=0.02 (-0.06; 0.09), p>.05 *SD ellipse:* b=-0.05 (-0.21; 0.11), p>.05 *Min. convex polygon:* b=-0.02 (-0.24; 0.20), p>.05 *Daily path area:* b=0.06 (-0.83; 0.96), p>.05 *Daily path area (pedestrian/bike):* b=0.05 (-0.05; 0.15), p>.05 (Health and aged care access/availability **0*0.166; 0*0.166; 0*0.166; 0*0.166; 0*0.166; 0*0.17**) 3. Banks/credit unions: *400m:* b=0.01 (-0.07; 0.05), p>.05 *800m:* b=0.02 (-0.04; 0.08), p>.05 *SD ellipse:* b=-0.05 (-0.16; 0.07), p>.05 *Min. convex polygon:* b=0.05 (-0.09; 0.20), p>.05 *Daily path area:* b=0.21 (-0.82; 1.24), p>.05 (Government/finance services access/availability **0*0.166; 0*0.166; 0*0.166; 0*0.166; 0*0.17**) *Daily path area (pedestrian/bike):* b=0.08 (0.01; 0.14), p<.05 (Government/finance services access/availability **+*0.166**) 4. Community centre/neighbourhood house: *400m:* b=-0.01 (-0.07; 0.04), p>.05 *800m:* b=0.01 (-0.06; 0.07), p>.05 *SD ellipse:* b=0.00 (-0.15; 0.14), p>.05 *Min. convex polygon:* b=0.04 (-0.13; 0.21), p>.05 *Daily path area:* b=-0.02 (-0.26; 0.21), p>.05 *Daily path area (pedestrian/bike):* b=0.03 (-0.05; 0.11), p>.05 (Social recreational facilities access/availability **0*0.166; 0*0.166; 0*0.166; 0*0.166; 0*0.166; 0*0.17**) 5. Convenience stores: *400m:* b=0.00 (-0.07; 0.07), p>.05 *800m:* b=0.04 (-0.02; 0.10), p>.05 *SD ellipse:* b=-0.03 (-0.11; 0.04), p>.05 *Min. convex polygon:* b=-0.02 (-0.11; 0.08), p>.05 *Daily path area:* b=-0.02 (-0.10; 0.07), p>.05 *Daily path area (pedestrian/bike):* b=0.06 (0.00; 0.12), p<.10  (Shops/commercial access/availability **0*0.166; 0*0.166; 0*0.166; 0*0.166; 0*0.166; 0*0.17**) 6. Entertainment: *400m:* b=-0.01 (-0.09; 0.07), p>.05 *800m:* b=0.02 (-0.04; 0.07), p>.05 *SD ellipse:* b=-0.02 (-0.10; 0.07), p>.05 *Min. convex polygon:* b=0.00 (-0.11; 0.11), p>.05 *Daily path area:* b=0.04 (-0.10; 0.17), p>.05 *Daily path area (pedestrian/bike):* b=0.05 (-0.01; 0.11), p>.05  (Social recreational facilities access/availability **0*0.166; 0*0.166; 0*0.166; 0*0.166; 0*0.166; 0*0.17**) 7. Food stores: *400m:* b=-0.02 (-0.08; 0.04), p>.05 *800m:* b=0.08 (-0.01; 0.17), p>.05 *SD ellipse:* b=-0.06 (-0.20; 0.08), p>.05 *Min. convex polygon:* b=0.00 (-0.23; 0.23), p>.05 *Daily path area:* b=0.29 (-0.66; 1.24), p>.05 *Daily path area (pedestrian/bike):* b=0.04 (-0.04; 0.12), p>.05 (Shops/commercial access/availability **0*0.166; 0*0.166; 0*0.166; 0*0.166; 0*0.166; 0*0.17**) 8. Gym and fitness facilities: *400m:* b=-0.02 (-0.08; 0.03), p>.05 *800m:* b=0.01 (-0.06; 0.08), p>.05  *SD ellipse:* b=-0.06 (-0.18; 0.07), p>.05 *Min. convex polygon:* b=-0.05 (-0.23; 0.12), p>.05 *Daily path area:* b=-0.09 (-0.34; 0.15), p>.05 *Daily path area (pedestrian/bike):* b=0.02 (-0.04; 0.09), p>.05 (Recreational facilities access/availability **0*0.166; 0*0.166; 0*0.166; 0*0.166; 0*0.166; 0*0.17**) 9. Library: *400m:* b=0.01 (-0.07; 0.08), p>.05 *800m:* b=0.04 (-0.02; 0.10), p>.05 *SD ellipse:* b=0.03 (-0.04; 0.10), p>.05 *Min. convex polygon:* b=0.04 (-0.05; 0.12), p>.05 *Daily path area:* b=-0.02 (-0.12; 0.07), p>.05 (Education facilities access/availability **0*0.166; 0*0.166; 0*0.166; 0*0.166; 0*0.17**) *Daily path area (pedestrian/bike):* b=0.06 (0.00; 0.12), p<.05 (Education facilities access/availability **+*0.166**) 10. Malls: *400m:* b=0.05 (-0.14; 0.05), p>.05 *800m:* b=0.04 (-0.02; 0.10), p>.05 *SD ellipse:* b=-0.02 (-0.09; 0.04), p>.05 *Min. convex polygon:* b=-0.02 (-0.09; 0.06), p>.05 *Daily path area:* b=-0.02 (-0.10; 0.06), p>.05  *Daily path area (pedestrian/bike):* b=0.01 (-0.05; 0.07), p>.05 (Shops/commercial access/availability **0*0.166; 0*0.166; 0*0.166; 0*0.166; 0*0.166; 0*0.17**) 11. Museum: *400m:* b=-0.05 (-0.17; 0.06), p>.05 *800m:* b=-0.02 (-0.09; 0.06), p>.05 *SD ellipse:* b=-0.02 (-0.08; 0.05), p>.05 *Min. convex polygon:* b=-0.02 (-0.08; 0.05), p>.05 *Daily path area:* b=-0.03 (-0.10; 0.03), p>.05 *Daily path area (pedestrian/bike):* b=-0.02 (-0.09; 0.05), p>.05 (Education facilities access/availability **0*0.166; 0*0.166; 0*0.166; 0*0.166; 0*0.166; 0*0.17**) 12. Nature/parks/botanical gardens: *400m:* b=-0.03 (-0.12; 0.06), p>.05 *800m:* b=0.01 (-0.07; 0.09), p>.05 *SD ellipse:* b=-0.03 (-0.10; 0.03), p>.05 *Min. convex polygon:* b=-0.03 (-0.10; 0.04), p>.05 *Daily path area:* b=-0.06 (-0.12; 0.00), p<.10 *Daily path area (pedestrian/bike):* b=-0.04 (-0.12; 0.03), p>.05 (Parks/public open space access/availability **0*0.166; 0*0.166; 0*0.166; 0*0.166; 0*0.166; 0*0.17**) 13. Pharmacies/drug stores/personal care: *400m:* b=-0.04 (-0.09; 0.02), p>.05 *800m:* b=0.02 (-0.04; 0.09), p>.05 *SD ellipse:* b=-0.06 (-0.20; 0.08), p>.05 *Min. convex polygon:* b=-0.01 (-0.23; 0.22), p>.05 *Daily path area:* b=0.14 (-0.84; 1.11), p>.05 *Daily path area (pedestrian/bike):* b=0.02 (-0.07; 0.10), p>.05 (Health care and aged access/availability **0*0.166; 0*0.166; 0*0.166; 0*0.166; 0*0.166; 0*0.17**) 14. Religious organisations: *400m:* b=0.04 (-0.02; 0.10), p>.05 *800m:* b=0.00 (-0.09; 0.10), p>.05 *SD ellipse:* b=-0.02 (-0.13; 0.08), p>.05 *Min. convex polygon:* b=0.05 (-0.10; 0.19), p>.05 *Daily path area:* b=0.30 (-0.89; 1.50), p>.05 *Daily path area (pedestrian/bike):* b=-0.01 (-0.08; 0.07), p>.05 (Religious institution access/availability **0*0.166; 0*0.166; 0*0.166; 0*0.166; 0*0.166; 0*0.17**) 15. Restaurants: *400m:* b=0.00 (-0.06; 0.06), p>.05 *800m:* b=0.08 (-0.01; 0.18), p>.05 *SD ellipse:* b=-0.05 (-0.21; 0.11), p>.05 *Min. convex polygon:* b=0.01 (-0.21; 0.22), p>.05 *Daily path area:* b=0.16 (-0.68; 0.99), p>.05 *Daily path area (pedestrian/bike):* b=0.06 (-0.04; 0.16), p>.05 (Food outlets access/availability **0*0.166; 0*0.166; 0*0.166; 0*0.166; 0*0.166; 0*0.17**) 16. Retail shopping: *400m:* b=0.00 (-0.06; 0.06), p>.05 *800m:* b=0.00 (-0.12; 0.11), p>.05 *SD ellipse:* b=-0.06 (-0.22; 0.10), p>.05 *Min. convex polygon:* b=-0.01 (-0.23; 0.21), p>.05 *Daily path area:* b=-0.03 (-0.79; 0.73), p>.05 *Daily path area (pedestrian/bike):* b=0.01 (-0.08; 0.11), p>.05 (Shops/commercial access/availability **0*0.166; 0*0.166; 0*0.166; 0*0.166; 0*0.166; 0*0.17**) 17. Services: *400m:* b=-0.01 (-0.08; 0.05), p>.05 *800m:* b=0.11 (0.01; 0.21), p<.05 (Destinations/services (overall/unspecific) access/availability **+*0.166**) *SD ellipse:* b=-0.06 (-0.23; 0.10), p>.05 *Min. convex polygon:* b=-0.01 (-0.23; 0.22), p>.05 *Daily path area:* b=0.10 (-0.85; 1.05), p>.05 *Daily path area (pedestrian/bike):* b=0.03 (-0.08; 0.14), p>.05 (Destinations/services (overall/unspecific) access/availability **0*0.166; 0*0.166; 0*0.166; 0*0.166; 0*0.17**)  ***Main effects with TotalWalking(steps/d):*** 1. Diversity (1 additional destination type): *400m:* b=39.32 (-138.51; 217.15), p>.05 *800m:* b=127.07 (-63.17; 317.30), p>.05 *All-mode activity space:* b=-45.68 (-505.59; 414.23), p>.05 *Pedestrian and bicycling activity space:* b=243.34 (35.97; 450.70), p<.05 (Land-use mix—destination diversity **+*0.166**) *SD ellipse:* b=-56.58 (-298.42; 185.26), p>.05 *Min. convex polygon:* b=23.80 (-279.45; 327.06), p>.05 (Land-use mix—destination diversity **0*0.166; 0*0.166; 0*0.166; 0*0.166; 0*0.17**) 2. Ambulatory health care services: *400m:* b=1.19 (-1.10; 3.49), p>.05 *800m:* b=1.21 (-1.81; 4.24), p>.05 *SD ellipse:* b=0.18 (-6.13; 6.49), p>.05 *Min. convex polygon:* b=2.05 (-6.57; 10.68), p>.05 *Daily path area:* b=26.43 (-8.35; 61.22), p>.05 *Daily path area (pedestrian/bike):* b=3.72 (-0.35; 7.80), p<.10 (Health and aged care access/availability **0*0.166; 0*0.166; 0*0.166; 0*0.166; 0*0.166; 0*0.17**) 3. Banks/credit unions: *400m:* b=0.06 (-2.23; 2.34), p>.05 *800m:* b=0.81 (-1.57; 3.19), p>.05 *SD ellipse:* b=0.53 (-4.13; 5.19), p>.05 *Min. convex polygon:* b=1.41 (-4.34; 7.17), p>.05 *Daily path area:* b=37.16 (-2.66; 76.99), p<.10 (Government/finance services access/availability **0*0.166; 0*0.166; 0*0.166; 0*0.166; 0*0.17**) *Daily path area (pedestrian/bike):* b=3.10 (0.44; 5.75), p<.05 (Government/finance services access/availability **+*0.166**) 4. Community centre/neighbourhood house: *400m:* b=-0.06 (-2.29; 2.16), p>.05 *800m:* b=0.12 (-2.45; 2.69), p>.05 *SD ellipse:* b=-0.44 (-6.11; 5.24), p>.05 *Min. convex polygon:* b=1.59 (-5.20; 8.37), p>.05 *Daily path area:* b=1.50 (-7.70; 10.71), p>.05 *Daily path area (pedestrian/bike):* b=1.98 (-1.19; 5.15), p>.05 (Social recreational facilities access/availability **0*0.166; 0*0.166; 0*0.166; 0*0.166; 0*0.166; 0*0.17**) 5. Convenience stores: *400m:* b=0.79 (-2.05; 3.64), p>.05 *800m:* b=2.20 (-0.09; 4.49), p>.05 *SD ellipse:* b=-0.61 (-3.60; 2.38), p>.05 *Min. convex polygon:* b=0.91 (-2.84; 4.66), p>.05 *Daily path area:* b=0.50 (-2.87; 3.87), p>.05 (Shops/commercial access/availability **0*0.166; 0*0.166; 0*0.166; 0*0.166; 0*0.17**) *Daily path area (pedestrian/bike):* b=3.26 (0.79; 5.74), p<.05 (Shops/commercial access/availability **+*0.166**)  6. Entertainment: *400m:* b=0.68 (-2.47; 3.83), p>.05 *800m:* b=0.78 (-1.56; 3.13), p>.05 *SD ellipse:* b=1.32 (-2.01; 4.64), p>.05 *Min. convex polygon:* b=0.95 (-3.26; 5.17), p>.05 *Daily path area:* b=1.68 (95% CL=-3.59; 6.96), p>.05 *Daily path area (pedestrian/bike):* b=2.10 (95% CL=-0.44; 4.64), p>.05 (Social recreational facilities access/availability **0*0.166; 0*0.166; 0*0.166; 0*0.166; 0*0.166; 0*0.17**) 7. Food stores: *400m:* b=-0.20 (-2.52; 2.12), p>.05 *800m:* b=3.28 (-.41; 6.98), p<.10 *SD ellipse:* b=-1.69 (-7.26; 3.87), p>.05 *Min. convex polygon:* b=1.97 (-7.04; 10.97), p>.05 *Daily path area:* b=24.89 (-12.18; 61.96), p>.05 *Daily path area (pedestrian/bike):* b=2.14 (-1.20; 5.49), p>.05 (Shops/commercial access/availability **0*0.166; 0*0.166; 0*0.166; 0*0.166; 0*0.166; 0*0.17**) 8. Gym and fitness facilities: *400m:* b=0.56 (-1.60; 2.71), p>.05 *800m:* b=1.37 (-1.29; 4.03), p>.05 *SD ellipse:* b=-1.67 (-6.75; 3.41), p>.05 *Min. convex polygon:* b=-0.02 (-7.01; 6.98), p>.05 *Daily path area:* b=-0.24 (-9.99; 9.51), p>.05 *Daily path area (pedestrian/bike):* b=2.51 (-0.15; 5.16), p>.05 (Recreational facilities access/availability **0*0.166; 0*0.166; 0*0.166; 0*0.166; 0*0.166; 0*0.17**) 9. Library: *400m:* b=1.23 (-1.59; 4.06), p>.05 *800m:* b=1.56 (-0.81; 3.92), p>.05 *SD ellipse:* b=1.54 (-1.33; 4.41), p>.05 *Min. convex polygon:* b=0.39 (-3.09; 3.86), p>.05 *Daily path area:* b=-1.25 (-4.99; 2.50), p>.05 *Daily path area (pedestrian/bike):* b=2.25 (-0.25; 4.76), p<.10 (Education facilities access/availability **0*0.166; 0*0.166; 0*0.166; 0*0.166; 0*0.166; 0*0.17**) 10. Malls: *400m:* b=-1.40 (-5.22; 2.42), p>.05 *800m:* b=2.35 (-0.09; 4.78), p<.10 *SD ellipse:* b=-0.15 (-2.75; 2.45), p>.05 *Min. convex polygon:* b=1.27 (-1.70; 4.25), p>.05 *Daily path area:* b=1.50 (-1.74; 4.74), p>.05 *Daily path area (pedestrian/bike):* b=0.99 (-1.53; 3.51), p>.05 (Shops/commercial access/availability **0*0.166; 0*0.166; 0*0.166; 0*0.166; 0*0.166; 0*0.17**) 11. Museum: *400m:* b=-1.37 (-5.83; 3.09), p>.05 *800m:* b=-1.12 (-4.09; 1.86), p>.05 *SD ellipse:* b=-0.70 (-3.17; 1.76), p>.05 *Min. convex polygon:* b=-0.24 (-2.92; 2.45), p>.05 *Daily path area:* b=0.00 (-2.58; 2.57), p>.05 *Daily path area (pedestrian/bike):* b=1.45 (-1.48; 4.38), p>.05 (Education facilities access/availability **0*0.166; 0*0.166; 0*0.166; 0*0.166; 0*0.166; 0*0.17**) 12. Nature/parks/botanical gardens: *400m:* b=-1.33 (-4.85; 2.20), p>.05 *800m:* b=-0.28 (-3.31; 2.75), p>.05 *SD ellipse:* b=-0.76 (-3.45; 1.93), p>.05 *Min. convex polygon:* b=0.09 (-2.61; 2.78), p>.05 *Daily path area:* b=-1.01 (-3.41; 1.39), p>.05 *Daily path area (pedestrian/bike):* b=0.02 (-3.19; 3.23), p>.05 (Parks/public open space access/availability **0*0.166; 0*0.166; 0*0.166; 0*0.166; 0*0.166; 0*0.17**) 13. Pharmacies/drug stores/personal care: *400m:* b=0.13 (-1.98; 2.25), p>.05 *800m:* b=1.28 (-1.32; 3.88), p>.05 *SD ellipse:* b=-1.25 (-6.83; 4.32), p>.05 *Min. convex polygon:* b=2.27 (-6.71; 11.26), p>.05 *Daily path area:* b=27.94 (-10.01; 65.90), p>.05 *Daily path area (pedestrian/bike):* b=1.76 (-1.71; 5.23), p>.05 (Health and aged care access/availability **0*0.166; 0*0.166; 0*0.166; 0*0.166; 0*0.166; 0*0.17**) 14. Religious organisations: *400m:* b=0.39 (-1.98; 2.75), p>.05 *800m:* b=-1.33 (-5.23; 2.56), p>.05 *SD ellipse:* b=-1.59 (-5.83; 2.64), p>.05 *Min. convex polygon:* b=0.52 (-5.09; 6.13), p>.05 *Daily path area:* b=9.29 (-38.07; 56.64), p>.05 *Daily path area (pedestrian/bike):* b=-0.78 (-4.02; 2.45), p>.05 (Religious institution access/availability **0*0.166; 0*0.166; 0*0.166; 0*0.166; 0*0.166; 0*0.17**) 15. Restaurants: *400m:* b=0.74 (-1.63; 3.12), p>.05 *800m:* b=3.53 (-0.18; 7.24), p<.10 *SD ellipse:* b=-0.28 (-6.57; 6.01), p>.05 *Min. convex polygon:* b=2.36 (-6.15; 10.87), p>.05 *Daily path area:* b=22.21 (-10.47; 54.89), p>.05 *Daily path area (pedestrian/bike):* b=3.19 (-0.85; 7.24), p>.05 (Food outlets access/availability **0*0.166; 0*0.166; 0*0.166; 0*0.166; 0*0.166; 0*0.17**) 16. Retail shopping: *400m:* b=0.01 (-2.45; 2.47), p>.05 *800m:* b=-1.57 (-6.10; 2.96), p>.05  *SD ellipse:* b=-0.34 (95% CL=-6.66; 5.98), p>.05 *Min. convex polygon:* b=2.21 (-6.33; 10.75), p>.05 *Daily path area:* b=18.47 (-11.17; 48.11), p>.05 *Daily path area (pedestrian/bike):* b=0.76 (-3.16; 4.67), p>.05 (Shops/commercial access/availability **0*0.166; 0*0.166; 0*0.166; 0*0.166; 0*0.166; 0*0.17**) 17. Services: *400m:* b=1.09 (-1.43; 3.61), p>.05 *800m:* b=4.72 (0.78; 8.66), p<.05 (Destinations/services (overall/unspecific) access/availability **+*0.166**)  *SD ellipse:* b=-0.40 (-6.90; 6.10), p>.05 *Min. convex polygon:* b=1.97 (-6.87; 10.82), p>.05 *Daily path area:* b=25.32 (-11.72; 62.36), p>.05 *Daily path area (pedestrian/bike):* b=2.41 (-2.04; 6.86), p>.05 (Destinations/services (overall/unspecific) access/availability **0*0.166; 0*0.166; 0*0.166; 0*0.166; 0*0.17**)  ***Main effects with TotalWalking(10,000+ steps/d; Yes/No):*** 1. Diversity (1 additional destination type): *400m:* OR=1.03 (0.88; 1.20), p>.05 *800m:* OR=1.06 (0.90; 1.26), p>.05 *All-mode activity space:* OR=0.90 (0.62; 1.35), p>.05 *Pedestrian and bicycling activity space:* OR=1.21 (0.97; 1.59), p>.05 *SD ellipse:* OR=0.94 (0.78; 1.17), p>.05 *Min. convex polygon:* OR=1.00 (0.78; 1.39), p>.05 (Land-use mix—destination diversity **0*0.166; 0*0.166; 0*0.166; 0*0.166; 0*0.166; 0*0.17**) 18. Total densities (10% difference): *400m:* OR=1.00 (1.00; 1.02), p>.05 *800m:* OR=1.01 (1.00; 1.03), p>.05 *All-mode activity space:* OR=1.02 (0.99; 1.05), p>.05 *Pedestrian and bicycling activity space:* OR=1.003 (1.00; 1.03), p>.05 *SD ellipse:* OR=1.01 (1.00; 1.03), p>.05 *Min. convex polygon:* OR=1.02 (1.00; 1.04), p>.05 (Destinations/services (overall/unspecific) access/availability **0*0.166; 0*0.166; 0*0.166; 0*0.166; 0*0.166; 0*0.17**) |  |
| 98 | WHI (Women’s Health Initiative)  Perry et al., 2013 [155] | N=1038 (mixed) Mean age: 66 years 100% female Response rate not reported Community-dwellers Seattle, USA | Cross-sectional Cluster: purposive Individuals: convenience Stratification: sex (female), age, menopausal and urbanisation Neighbourhood definition: 1km buffer | Age, education, health status, ethnicity, previous vigorous exercise history, personal income, marital status, energy expenditure (non-walking), neighbourhood income | *Self-report [WHI questionnaire; reliable]:*  Walking (MET hr/wk) 🡪 Total walking *TotalWalking(MET hr/wk)* | *Objective [Walkable and Bikable Communities Index; unvalidated]:*  1. Walkability 🡪 Walkability | Interaction terms for: Walkability*age  Walkability*ethnicity: White (n≈987), Asian/Pacific Islander (n≈21), African-American (n≈10), American Indian/Alaskan Native (n≈10), Latina (n≈10)  Walkability*marital status: Married (n≈654), previously married/never (n≈384)  Walkability*median family income: <$10,000 (n≈21), $10k-$19,999 (n≈145), $20,000-$34,999 (n≈280), $35,000-$49,999 (n≈239), $50,000-$74,999 (n≈197), $75,000-$99,999 (n≈83), $100,000-$149,999 (n≈52), $150,000+ (n≈21)  Walkability*education level: Less than high school (n≈31), graduated high school (n≈135), some college (n≈467), graduated college (n≈135), >College (n≈270)  Walkability*prior exercise history: Very hard exercise 3 d/wk at 35 years old (n≈457) | Multivariate linear regression | *No significant moderating effects.* ***Main effect with TotalWalking(MET hr/wk):*** 1. Walkability—b (95% CIs): b=0.001 (0.017; 0.019), p=.948 (Walkability **0**) |  |
| 99 | WISER (Wellness Information Survey Evaluation Research) study de Melo 2013 [61] | N=88 (urban) Mean age: 71 years 55% female 82.6% response rate (at follow-up) Community-dwellers Winnipeg, Canada | Longitudinal Cluster: purposive Individuals: convenience Stratification: select neighbourhoods (none) Neighbourhood definition: participant delineation | Sex, physical function, chronic disease, BMI | *Objective [Digiwalker pedometer; validated]:*  Total change in step/d (increase; Yes/No) 🡪 Total walking *TotalWalking(change; increased steps/d; Yes/No)* | *Perceived [modified version of NEWS questionnaire; unvalidated]:* All factor analysis-derived variables:  1. Traffic safety 🡪 Traffic/pedestrian safety 2. Sidewalks 🡪 Walk-friendly infrastructure 3. Aesthetics 🡪 Greenery and aesthetically pleasing scenery 4. Walkability safety 🡪 Crime/personal safety | None | Multivariate logistic regression analysis | ***Main effects with TotalWalking(change); increased steps/d; Yes/No):*** 1. Traffic safety—OR (95% CIs): OR=0.46 (0.16; 1.30), p=.14  (Traffic/pedestrian safety **0**) 2. Sidewalks: OR=0.54 (0.18; 1.58), p=.26  (Walk-friendly infrastructure **0**) 3. Aesthetics: OR=1.75 (0.58; 5.32, p=.31  (Greenery and aesthetically pleasing scenery **0**) 4. Walkability safety: OR=7.24 (1.54; 33.92), p=.01 (Crime/personal safety **0**) | Table 9—Model 4. |
| 100 | ZHTS (Zhongshan Household Travel Survey) Zhang et al., 2014 [80] | N=4308 (urban) 60+ years (no mean) 29-48% female (two groups) 85.4% response rate Community-dwellers Zhongshan, China | Cross-sectional Cluster: purposive Individuals: random Stratification: Traffic analysis zone (urbanisation) Neighbourhood definition: Traffic analysis zone (administrative) | Age, sex, employment status, household size, household income, number of bikes, e-bikes, motorbikes, cars in household | *Self-report [ZHTS questionnaire; unvalidated]:*  Likelihood of walking 🡪 Total walking *TotalWalking(likelihood; Yes/No)* Frequency of walking (trips/d) 🡪 Total walking *TotalWalking(trips/d)*  Duration of total walking among walkers (mins/d) 🡪 Total walking *TotalWalking(mins/d)* | *Objective [ArcGIS—Zhongshan Municipal Bureau of Urban Planning; unvalidated]:*  1. Population density 🡪 Residential density 2. Access to commercial and service destinations 🡪 Shops/commercial access/availability 3. % greenspace 🡪 Greenery and aesthetically pleasing scenery 4. Land-use mix: diversity 🡪 Land-use mix—destination diversity 5. Sidewalk density 🡪 Walk-friendly infrastructure 6. Bus stop density 🡪 Public transport access/availability | None | Zero-inflated Poisson regression model | ***Main effects with TotalWalking(likelihood; Yes/No):*** 1. Population density: (Residential density **+**) 2. Access to commercial and service destinations: (Shops/commercial access/availability **0**) 3. % greenspace: (Greenery and aesthetically pleasing scenery **+**) 4. Land-use mix—diversity: (Land-use mix—destination diversity **0**) 5. Sidewalk density:  (Walk-friendly infrastructure **+**) 6. Bus stop density: (Public transport access/availability **+**)  ***Main effects with TotalWalking(trips/d):*** 1. Population density: Poisson: IRR=-0.008, p<.05 (Residential density **-**) 2. Access to commercial and service destinations: Poisson: IRR=0.004, p<.05 (Shops/commercial access/availability **+**) 3. % greenspace: Poisson: IRR=0.305, p<.05 (Greenery and aesthetically pleasing scenery **+**) 4. Land-use mix—diversity: Poisson: IRR=-0.360, p<.05 (Land-use mix—destination diversity **-**) 5. Sidewalk density: Poisson: IRR=0.034, p<.05  (Walk-friendly infrastructure **+**) 6. Bus stop density: Poisson: IRR=0.012, p<.05 (Public transport access/availability **+**)  ***Main effects with TotalWalking(mins/d):*** 1. Population density: Poisson: IRR=-0.005, p<.05 (Residential density **-**) 2. Access to commercial and service destinations: Poisson: IRR=0.002, p<.05 (Shops/commercial access/availability **+**) 3. % greenspace: Poisson: IRR=0.224, p<.05 (Greenery and aesthetically pleasing scenery **+**) 4. Land-use mix—diversity: Poisson: IRR=-0.041, p<.05 (Land-use mix—destination diversity **-**) 5. Sidewalk density: Poisson: IRR=0.016, p<.05 (Walk-friendly infrastructure **+**) 6. Bus stop density: Poisson: IRR=0.012, p<.05 (Public transport access/availability **+**) | Table 2. |
