# Supplementary material for: Built environmental correlates of older adults’ total physical activity and walking: a systematic review and meta-analysis
Source: Int J Behav Nutr Phys Act. 2017 Aug 7;14:103. doi: 10.1186/s12966-017-0558-z (PMC5547528; doi:10.1186/s12966-017-0558-z)
Supplement: Supplementary file 2 — Reviewed total physical activity articles (N = 100) – Quality assessment. (DOCX 106 kb) [file 12966_2017_558_MOESM2_ESM.docx]

**Table S3. Reviewed total physical activity articles (N=100) – Quality assessment**

| **#** | **Study name and authors** | **Study design [weight: cross-sectional = 0; longitudinal = 1; quasi-experimental = 2]** | **Stratification of recruitment sites by relevant environmental attributes [weight 1]** | **Adequate response rate (>60%) or shown to be representative of the population [weight 1]** | **Outcome measures shown to be reliable and valid [weight 1]** | **Adjustment for socio-demographic covariates (at least age, gender, and education considered) [weight 1]** | **Adjustment for self-selection [weight 1]** | **Appropriate analytical approach – accounting for clustering (if needed) [weight 1/3]** | **Appropriate analytical approach – accounting for distributional assumptions [weight 1/3]** | **Appropriate analytical approach –analyses conducted and presented correctly (e.g., formal testing of moderators; presentation of point estimates and *p*-values, 95% CIs) [weight 1/3]** | **Did not (inappropriately) categorise continuous environmental exposure [weight 1]** | **Total quality score (maximum of 9)** |
| --- | --- | --- | --- | --- | --- | --- | --- | --- | --- | --- | --- | --- |
| 1 | Active Living Study  Nathan et al., 2014 ^[1]^ | Cross-sectional | Y | N | Y | Y | Y | Y | Y | Y | Y | 6 |
| 2 | Active Living Study  Nathan et al., 2014 ^[2]^ | Cross-sectional | Y | N | Y | Y | N | Y | Y | Y | Y | 5 |
| 3 | Active Living Study  Nathan et al., 2014 ^[3]^ | Cross-sectional | Y | N | Y | Y | Y | Y | Y | Y | Y | 6 |
| 4 | AGES Hanibuchi et al., 2011 ^[4]^ | Cross-sectional | N | N | Y | Y | N | N | Y | N (significance of interaction effects were not provided) | Y | 4.33 |
| 5 | AIBL study Cerin et al., 2016 ^[5]^ | Cross-sectional | N | N | Y | Y | N | Y | Y | Y | Y | 4 |
| 6 | ALECS study Cerin et al., 2016 ^[6]^ | Cross-sectional | Y | Y | Y | Y | Y | Y | Y | Y | Y | 7 |
| 7 | Australian Time Use Survey 2006 Espinel et al., 2015 ^[7]^ | Cross-sectional | N | Y | N (PA diary; unvalidated) | Y | N | Y (not needed) | Y | Y | Y | 4 |
| 8 | Behavior Change Consortium Initiative – Rhode Island Trail King et al., 2006 ^[8]^ | Cross-sectional | N | N | Y | Y | N | Y | N | Y | Y | 4.67 |
| 9 | BEPAS Seniors Van Cauwenberg et al., 2016 ^[9]^ | Cross-sectional | Y | N | Y | Y | Y | Y | Y | Y | Y | 6 |
| 10 | BEPAS Seniors Van Holle et al., 2016 ^[10]^ | Cross-sectional | Y | N | Y | Y | N | Y | Y | Y | Y | 5 |
| 11 | British Regional Heart Study & British Women’s Heart Health Study Jefferis et al., 2014 ^[11]^ | Cross-sectional | Y | N | Y | N (education missing) | N | Y (included region in models) | Y | Y | N | 3 |
| 12 | Canada’s General Social Survey Time Use Spinney & Millward 2014 ^[12]^ | Cross-sectional | Y | Y (across many variables, albeit %female: 65.8, 63.2, 60.2, 58.8) | N | Y (in main logistic regression analysis) | N | Y | Y | Y | Y | 5 |
| 13 | CCHS 2008/2009 Winters et al., 2015 ^[13]^ | Cross-sectional | N | Y (albeit ↑% higher educated) | N | Y | N | N | Y | Y | Y | 3.67 |
| 14 | CHIS 2003 data Li et al., 2015 ^[14]^ | Cross-sectional | N | Y (albeit Asians oversampled) | N | Y | N | N | Y (observed overdispersion) | N (no formal test of moderation by ethnicity) | N (exposure variables categorised without justification) | 2.33 |
| 15 | CNDS Mendes de Leon et al., 2009 ^[15]^ | Cross-sectional | N | Y (61% female) | N | Y | N (years in neighbourhood) | Y | Y | Y | Y | 4 |
| 16 | DIY Streets Thompson et al., 2012 ^[16]^ | Quasi-experimental | Y (comparison streets) | N (large discrepancies across some demographics, e.g., ethnicity—2008: 24.5% vs. 11.1% not white British) | N | N | N | N | N (initial tests were non-parametric, then same variables fitted to a regression model without mention of, e.g., transformation) | N (missing b-values for multiple variables, no 95% CIs) | Y | 4 |
| 17 | Easy Steps to Health Merom et al., 2015 ^[17]^ | Cross-sectional | N | N (73% female; inactive participants only -- <120 min/wk) | Y | Y | N | Y | Y | Y | N | 3 |
| 18 | EPOSA – Dutch trial Timmermans et al., 2016 ^[18]^ | Cross-sectional | Y | Y (although urban (n=176; 71%) participants were oversampled vs. rural (n=53; 22%)) | Y | Y | N | N | Y | Y | Y | 6.67 |
| 19 | Great Britain older adults 1 (name assigned) Sugiyama & Ward Thompson 2007 ^[19]^ | Cross-sectional | Y | N | N | N | N | N | Y | N (no data reported related to chi-square test) | Y | 2.33 |
| 20 | Great Britain older adults 1 (name assigned) Sugiyama et al., 2009 ^[20]^ | Cross-sectional | Y | N | N | Y (sex was not associated with outcome, hence exclusion from final model) | N | N | Y | Y | N (all environmental exposures categorised without justification) | 2.67 |
| 21 | HAN Walking Study Satariano et al., 2010 ^[21]^ | Cross-sectional | Y | N (albeit 77% female; ↑% higher educated) | N | Y | N | Y (study site included in models) | Y | N (many missing point estimates and associated CIs and p-values) | N (no justification for categorising exposure variables) | 2.67 |
| 22 | Harvard Alumni Study Lee et al., 2009 ^[22]^ | Cross-sectional & longitudinal | Y (conveniently recruited, however, there was variability in urbanisation) | N | Y | Y | N | N | Y | Y | Y | 5.67 |
| 23 | Health and Retirement study Latham et al., 2015 ^[23]^ | Cross-sectional | N | Y (albeit 67% female; only those reporting mobility impairment included) | N | Y | N | Y | Y | Y | Y | 4 |
| 24 | Health and Wellbeing Surveillance System Nathan et al., 2012 ^[24]^ | Cross-sectional | Y | Y | Y | Y | N | Y (not needed) | Y | Y | Y | 6 |
| 25 | Health and Wellbeing Surveillance System Villanueva et al., 2014 ^[25]^ | Cross-sectional | Y | Y | Y | Y | N | Y (not needed) | Y | Y | Y (despite categorising walkability, the authors also reported the continuous variable) | 6 |
| 26 | Hong Kong Elderly Study  Cerin et al., 2013 ^[26]^ | Cross-sectional | Y | Y | Y | Y | N | Y | Y | Y | Y | 6 |
| 27 | Kasama Study Tsunoda et al., 2012 ^[27]^ | Cross-sectional | N | N (excluded those with difficulty walking) | Y | Y | N | Y (not needed) | Y | Y (no moderators considered) | N (categorised all exposure variables without justification) | 3 |
| 28 | KNHANES 2007/2008 Yeom et al., 2011 ^[28]^ | Cross-sectional | Y | Y | Y | N | N | Y | Y | Y | Y | 5 |
| 29 | LL-FDI study Morris et al., 2008 ^[29]^ | Cross-sectional | N | N | Y | N | N | Y | Y | N | Y | 2.67 |
| 30 | LL-FDI study Hall & McAuley, 2010 ^[30]^ | Cross-sectional | N | N | Y | N | N | Y (not needed) | Y | Y | Y | 3 |
| 31 | Malaysian National Health and Morbidity Survey III 2006 data Kaur et al., 2015 ^[31]^ | Cross-sectional | Y | Y | Y | Y | N | N | Y | Y | Y | 5.67 |
| 32 | Melbourne older adults study 1 (name assigned) Bird et al., 2009 ^[32]^ | Cross-sectional | N | N | Y | N | N | N | Y | Y | Y | 2.67 |
| 33 | Melbourne older adults study 1 (name assigned) Bird et al., 2010 ^[33]^ | Cross-sectional | N | N | Y (albeit translated version) | N | N | N | N | N | Y | 2 |
| 34 | MOBILIZE Boston study Procter-Gray et al., 2015 ^[34]^ | Cross-sectional | N | Y | N | Y | N | N | Y | Y | Y | 3.67 |
| 35 | Neighbourhoods and Physical Activity in Elderly Men Michael et al., 2010 ^[35]^ | Longitudinal | N | N | Y | Y | N | Y | Y | N (many missing values related to RR and 95% CI) | N (environmental exposures categorised without justification) | 3.67 |
| 36 | Netherlands Housing Survey (WoON) data Jongeneel-Grimen et al., 2013 ^[36]^ | Cross-sectional | Y | Y | N | Y | N | Y | Y | N (no formal testing of moderation in older adults) | Y | 4.67 |
| 37 | Netherlands Housing Survey (WoON) data Jongeneel-Grimen et al., 2014 ^[37]^ | Cross-sectional | Y | Y | N | Y | N | Y | Y | N (no formal testing of moderation in older adults) | Y | 4.67 |
| 38 | *No study name* Aird et al., 2015 ^[38]^ | Cross-sectional | Y | N (🡩 income in some; ↓income in others) | N | N | N | N | N (no mention of assessing normality; likely skewed based on mean and range reported) | Y | Y | 2.33 |
| 39 | *No study name* Arnadottir et al., 2009 ^[39]^ | Cross-sectional | Y | Y | Y | Y | N | N | Y | Y | Y | 5.67 |
| 40 | *No study name* Asawachaisuwikrom 2001 ^[40]^ | Cross-sectional | Y | N (↓ education – elementary school completion compulsory until 1978 (Smalley, 1994)) | Y | N | N | N (no adjustment for village cluster) | Y | Y | Y | 3.67 |
| 41 | *No study name* Baceviciene & Alisauskas 2013 ^[41]^ | Cross-sectional | N | N (61% female; 39% university-educated) | Y | N | N | Y (not needed) | Y | Y | Y | 3 |
| 42 | *No study name* Bocker et al., 2016 ^[42]^ | Cross-sectional | Y | N (63% female; underrepresentation of lower-educated) | N | Y | N | Y | Y | Y | Y | 4 |
| **43** | *No study name* Carvalho Sampaio et al., 2012 ^[43]^ | Cross-sectional | Y | N | N (questionnaire) | N | N | Y (not needed) | Y | N (no formal testing of moderators) | Y | 2.67 |
| 44 | *No study name* Chad et al., 2005 ^[44]^ | Cross-sectional | N | Y | Y | N | N | Y (not needed) | Y | N (no formal testing of moderators) | N (all environmental exposures were categorised without justification) | 2.67 |
| 45 | *No study name* Chaudhury et al., 2016 ^[45]^ | Cross-sectional | Y (population density and median household income) | N (6% response rate; 64% female; 44% degree-educated) | N | Y | N | N | Y | Y | N (categorised scale without justification) | 2.67 |
| 46 | *No study name* Chen et al.,2013 ^[46]^ | Cross-sectional | N | N | Y | N | N | Y | Y | Y | N | 2 |
| 47 | *No study name* de Melo et al., 2010 ^[47]^ | Cross-sectional | N | N (75% female + response rate not reported) | Y | Y | N | N | Y (negative binomial model fitted to skewed data) | Y | Y | 3.67 |
| 48 | *No study name* Gallagher et al., 2012 ^[48]^ | Cross-sectional | N | Y | Y | Y | N | Y (not needed) | N | Y | Y | 4.67 |
| 49 | *No study name* Gomez et al., 2010 ^[49]^ | Cross-sectional | Y | Y | N (adapted without validation) | Y | N | Y | Y | Y | N (categorisation of exposure variables unjustified) | 4 |
| 50 | *No study name* Grant-Savela et al., 2010 ^[50]^ | Cross-sectional | N | N (↑% higher educated) | N (adapted PASE questionnaire without validation) | N | N | Y (not needed) | Y | Y | Y | 3 |
| 51 | *No study name* Inoue et al., 2011 ^[51]^ | Cross-sectional | Y | Y | N | Y | N | Y | Y | N (no formal testing of moderation) | N (exposure variable dichotomised without justification) | 3.67 |
| 52 | *No study name* King et al., 2003 ^[52]^ | Cross-sectional | N | N | Y | N | N | N | Y | Y | Y | 2.67 |
| 53 | *No study name* Koh et al., 2015 ^[53]^ | Cross-sectional | N | N | N | Y | N | N | Y | Y | Y (even though table 1 reports “categorical” exposure variables, table 4 reports them as continuous…) | 2.67 |
| 54 | None Kolbe-Alexander et al., 2015 ^[54]^ | Cross-sectional | Y (SES) | N (78% female) | Y | N | N | N | Y | N (no formal testing of moderators) | Y | 3.33 |
| 55 | *No study name* Lee & Park, 2015 ^[55]^ | Cross-sectional | Y | Y | Y | Y | Y (attitude toward regular walking; PA self-efficacy; intention to walk regularly) | N | Y | N (no formal testing of moderator) | Y | 6.33 |
| 56 | *No study name* Lotfi & Koohsari, 2011 ^[56]^ | Cross-sectional | Y | N (no sociodemographic info reported) | N | N | N | N | N | N | Y | 2 |
| 57 | *No study name* Maisel et al., 2016 ^[57]^ | Cross-sectional | Y | N (74% female) | Y | Y | Y (overall neighbourhood satisfaction) | N | Y | N (no formal testing of moderators) | N | 4.33 |
| 58 | *No study name* Mowen et al., 2007 ^[58]^ | Cross-sectional | Y (SES) | N | N | N | N | Y | N | Y | Y | 2.67 |
| 59 | *No study name* Pelclova et al.,2012 ^[59]^ | Cross-sectional | N | N (88% female) | Y (albeit translated version) | N | N | N | Y | Y | N (exposure variables dichotomised without justification) | 1.67 |
| 60 | *No study name* Persson et al., 2011 ^[60]^ | Cross-sectional | N | Y (albeit 77% female) | N | Y | N | Y (borough lived included in model) | Y | N (majority of ORs and CIs missing) | N | 2.67 |
| 61 | *No study name* Salvador et al., 2010 ^[61]^ | Cross-sectional | Y (large SES inequality in area; probability proportional to size measures) | Y | Y | Y | N | N | Y | Y | Y | 5.67 |
| 62 | *No study name* Sewo Sampaio et al., 2013 ^[62]^ | Cross-sectional | Y | N | N | N | N | Y | Y | N (actual p-values not reported) | Y | 2.67 |
| 63 | *No study name* Shin et al., 2011 ^[63]^ | Cross-sectional | N | Y | N (modified version of CHAMPS--modifications not reported) | N | N | N | Y | N | Y | 2.33 |
| 64 | *No study name* Shores et al., 2009 ^[64]^ | Cross-sectional | N | N | N | N (education not in model) | N | Y | Y | Y | Y | 2 |
| 65 | *No study name* Tanaka et al., 2016 ^[65]^ | Cross-sectional | Y | Y | Y | Y | N | N | Y | Y | Y | 5.67 |
| 66 | *No study name* Towne Jr., 2016 ^[66]^ | Cross-sectional | N | N | N | N (education not in model) | N | N | Y | Y | N (both environmental exposures were categorised without justification) | 0.67 |
| 67 | *No study name* Wang & Lee 2010 ^[67]^ | Cross-sectional | N | N | N | Y | N | Y | Y | Y | Y | 3 |
| 68 | *No study name* Wilcox et al., 2003 ^[68]^ | Cross-sectional | N | N | Y | Y | Y (decisional balance of PA: pros vs. cons) | N | Y | Y | Y | 4.67 |
| 69 | NSW Falls Prevention Baseline Survey 2009 Macniven et al., 2014 ^[69]^ | Cross-sectional | N | Y (albeit 19% osteoporosis; 58% female; 56% suffering arthritis) | N | Y | Y (make time to be active) | N | Y | N (no formal test of moderation) | Y | 4.33 |
| 70 | NSW OPHS Lim & Taylor 2005 ^[70]^ | Cross-sectional | N | Y | N | Y | N | Y | Y | Y | N (categorised feel safe in neighbourhood variable without justification) | 3 |
| 71 | Nurses’ Health Study James et al., 2013 ^[71]^ | Cross-sectional | N | Y | Y | Y | N | Y | N | Y | Y | 4.67 |
| 72 | Nurses’ Health Study Troped et al., 2014 ^[72]^ | Cross-sectional | N | Y | Y | Y | N | Y | Y | Y | N (intersection and population density were categorised without justification) | 4 |
| 73 | Oslo Health Study Piro et al., 2006 ^[73]^ | Cross-sectional | N | N | N | Y | N | Y | Y | Y | N | 2 |
| 74 | Physical Activity Monitor 2002 Pan et al., 2009 [74] | Cross-sectional | N | N | Y | Y | Y (PA intention) | Y | Y | Y | Y | 5 |
| 75 | PACS (Physical Activity Cohort Scotland) McMurdo et al., 2012 ^[75]^ | Cross-sectional | Y | N | Y | N (education not added) | N | N | Y | Y | Y | 3.67 |
| 76 | PACS Sniehotta et al., 2013 ^[76]^ | Cross-sectional | Y | N | Y | N (education not added) | Y (intention) | N | Y | Y | Y | 4.67 |
| 77 | Project OPAL Davis et al., 2011 ^[77]^ | Cross-sectional | Y (amenity access and SES) | N (↑% higher educated) | Y | N | N | N (clustering at the clinical level not accounted for) | Y | Y | N | 2.67 |
| 78 | Project OPAL Fox et al., 2011 ^[78]^ | Cross-sectional | Y | N | Y | N | N | N | Y | Y | N (categorised distance to nearest shop without justification) | 2.67 |
| 79 | Project OPAL Thompson et al., 2011 ^[79]^ | Cross-sectional | Y | N (↑% higher educated) | Y | N | N | N | Y | Y | Y | 3.67 |
| 80 | Project RICE pilot Han et al., 2016 ^[80]^ | Cross-sectional | N | N | Y (albeit translated version) | Y | N | Y (not needed) | Y | Y | Y | 4 |
| 81 | SHAPE Li et al., 2005a ^[81]^ | Cross-sectional | Y | N | Y | N | N | Y | Y | Y | Y | 4 |
| 82 | SHAPE Li et al., 2005b ^[82]^ | Longitudinal | Y | N (64% female + inadequate response rate) | Y | N (age and sex not added) | N | Y | Y | Y | Y | 5 |
| 83 | SHAPE Michael et al., 2006 ^[83]^ | Cross-sectional | Y | N (67% female + inadequate response rate) | Y | Y | N | N | Y | N (many ORs missing, and all 95% CIs not reported) | N (perceived environmental exposures categorised without justification) | 3.33 |
| 84 | SHAPE Nagel et al., 2008 ^[84]^ | Cross-sectional | N | N (70% female + inadequate response rate) | Y | Y | N | Y | Y | Y | Y | 4 |
| 85 | SMARTRAQ Frank et al., 2010 ^[85]^ | Cross-sectional | Y | N | Y (travel survey) | Y | N | Y | Y | Y | N (categorisation from continuous walkability index) | 4 |
| 86 | SNQLS Kerr et al., 2011 ^[86]^ | Cross-sectional | Y | N (71% female) | Y | N | N | N | N (outcome likely skewed) | Y | Y | 3.33 |
| 87 | SNQLS  Carlson et al., 2012 ^[87]^ | Cross-sectional | Y | N (🡩 % Caucasians; higher education) | Y | Y | N | Y | N (outcome likely skewed) | N | N | 3.33 |
| 88 | SNQLS Bracy et al., 2014 ^[88]^ | Cross-sectional | Y | N (🡩 % Caucasians; higher education) | Y | Y | N | Y | N (outcome likely skewed) | Y | Y | 4.67 |
| 89 | SNQLS Cain et al., 2014 ^[89]^ | Cross-sectional | Y | N (↑% Caucasians; higher educated) | Y | Y | N | Y | N (outcome likely skewed) | N | Y | 4.33 |
| 90 | SNQLS Carlson et al., 2014 ^[90]^ | Cross-sectional | Y | N (🡩 % Caucasians; higher education) | Y | Y | N | Y | N (outcome likely skewed) | Y | Y | 4.67 |
| 91 | SNQLS Ding et al., 2013 ^[91]^ | Cross-sectional | Y | N (🡩 % Caucasians; higher education) | Y | Y | N | Y | N (outcome likely skewed) | Y | Y | 4.67 |
| 92 | TILDA McKee et al., 2015 ^[92]^ | Cross-sectional | Y | N | Y | Y | N | N | Y | Y | Y | 4.67 |
| 93 | TILDA Murtagh et al., 2015 ^[93]^ | Cross-sectional | Y | N | Y | Y | N | Y | Y | Y | Y | 5 |
| 94 | UAB Study of Aging Hannon et al., 2012 ^[94]^ | Cross-sectional | Y | N | N | Y | N | N | Y | Y | Y | 3.67 |
| 95 | VoisiNuAge Gauvin et al., 2012 ^[95]^ | Longitudinal | N | N (↑% higher educated and non-low income residents) | Y | Y | Y (proximity to friend or relative) | Y (examined spatial autocorrelation) | Y (categorisation of outcome variable justified) | Y | N (categorisation of exposure variables unjustified) | 4 |
| 96 | VoisiNuAge Julien et al., 2015 ^[96]^ | Cross-sectional | N | N | Y | N | N | Y | Y | Y | Y | 2.67 |
| 97 | Walk the Talk Hirsch et al., 2016 ^[97]^ | Cross-sectional | Y | N (low income older adults only=inadequate response rate) | Y | Y | N | N | Y | Y | Y | 4.67 |
| 98 | WHI Perry et al., 2013 ^[98]^ | Cross-sectional | N | N (participants keen to be a part of research + inadequate response rate) | Y (reliable) | Y | N | Y | N | Y | Y | 3.67 |
| 99 | WISER study De Melo 2013 ^[99]^ | Longitudinal | N | Y | Y | N | N | N | N (outcome variable poorly defined: increased steps vs. decreased steps—no specified amount reported) | Y | Y | 4.33 |
| 100 | ZHTS 2014 Zhang et al., 2014 ^[100]^ | Cross-sectional | N | Y | N | Y | Y (pro-walking) | N | Y (insignificant overdispersion reported, zero-inflated Poisson model adopted) | Y | N | 3.67 |

**References**

1. Nathan, A., L. Wood, and B. Giles-Corti, *Perceptions of the Built Environment and Associations With Walking Among Retirement Village Residents.* Environment and Behavior, 2014. **46**(1): p. 46-69.

2. Nathan, A., L. Wood, and B. Giles-Corti, *Examining correlates of self-reported and objectively measured physical activity among retirement village residents.* Australasian Journal on Ageing, 2014. **33**(4): p. 250-256.

3. Nathan, A., L. Wood, and B. Giles-Corti, *Exploring Socioecological Correlates of Active Living in Retirement Village Residents.* Journal of Aging & Physical Activity, 2014. **22**(1): p. 1-15.

4. Hanibuchi, T., et al., *Neighborhood built environment and physical activity of Japanese older adults: results from the Aichi Gerontological Evaluation Study (AGES).* BMC Public Health, 2011. **11**: p. 657.

5. Cerin, E., et al., *Associations of neighborhood environment with brain imaging outcomes in the AIBL cohort.* Alzheimers Dement, 2016.

6. Cerin, E., et al., *Associations of objectively-assessed neighborhood characteristics with older adults’ total physical activity and sedentary time in an ultra-dense urban environment: Findings from the ALECS study.* Health and Place, 2016. **42**: p. 1-10.

7. Espinel, P.T., et al., *Older adults' time in sedentary, light and moderate intensity activities and correlates: Application of Australian Time Use Survey.* Journal of Science and Medicine in Sport, 2015. **18**(2): p. 161-166.

8. King, A.C., et al., *Perceived environments as physical activity correlates and moderators of intervention in five studies.* American Journal of Health Promotion, 2006. **21**(1): p. 24-35.

9. Van Cauwenberg, J., et al., *Neighborhood walkability and health outcomes among older adults: The mediating role of physical activity.* Health Place, 2016. **37**: p. 16-25.

10. Van Holle, V., et al., *The Association between Belgian Older Adults' Physical Functioning and Physical Activity: What Is the Moderating Role of the Physical Environment?* PLoS One, 2016. **11**(2): p. e0148398.

11. Jefferis, B.J., et al., *Adherence to physical activity guidelines in older adults, using objectively measured physical activity in a population-based study.* BMC Public Health, 2014. **14**(1).

12. Spinney, J.E.L. and H. Millward, *Active Living Among Older Canadians: A Time-Use Perspective Over 3 Decades.* Journal of Aging and Physical Activity, 2014. **22**(1): p. 103-113.

13. Winters, M., et al., *Older adults' outdoor walking and the built environment: does income matter?* BMC Public Health, 2015. **15**(1): p. 1-8 8p.

14. Li, Y., D. Kao, and T.Q. Dinh, *Correlates of Neighborhood Environment With Walking Among Older Asian Americans.* Journal of Aging & Health, 2015. **27**(1): p. 17-34.

15. Mendes de Leon, C.F.C., K. A., et al., *Neighborhood Social Cohesion and Disorder in Relation to Walking in Community-Dwelling Older Adults A Multilevel Analysis.* Journal of Aging and Health, 2009. **21**(1): p. 155-171.

16. Thompson, C.W., et al., *Do changes to the local street environment alter behaviour and quality of life of older adults? the 'DIY Streets' intervention.* British Journal of Sports Medicine, 2012. **48**(13): p. 1059-1065.

17. Merom, D., et al., *Neighborhood walkability, fear and risk of falling and response to walking promotion: The Easy Steps to Health 12-month randomized controlled trial.* Prev Med Rep, 2015. **2**: p. 704-10.

18. Timmermans, E.J., et al., *The association of the neighbourhood built environment with objectively measured physical activity in older adults with and without lower limb osteoarthritis.* BMC Public Health, 2015. **15**: p. 710.

19. Sugiyama, T. and C.W. Thompson, *Older people's health, outdoor activity and supportiveness of neighbourhood environments.* Landscape and Urban Planning, 2007. **83**(2-3): p. 168-175.

20. Sugiyama, T., C.W. Thompson, and S. Alves, *Associations Between Neighborhood Open Space Attributes and Quality of Life for Older People in Britain.* Environment and Behavior, 2009. **41**(1): p. 3-21.

21. Satariano, W.A., et al., *Lower-Body Function, Neighborhoods, and Walking in an Older Population.* American Journal of Preventive Medicine, 2010. **38**(4): p. 419-428.

22. Lee, I.M., R. Ewing, and H.D. Sesso, *The Built Environment and Physical Activity Levels The Harvard Alumni Health Study.* American Journal of Preventive Medicine, 2009. **37**(4): p. 293-298.

23. Latham, K. and M.M. Williams, *Does Neighborhood Disorder Predict Recovery From Mobility Limitation? Findings From the Health and Retirement Study.* Journal of Aging and Health, 2015. **27**(8): p. 1415-1442.

24. Nathan, A., et al., *Access to commercial destinations within the neighbourhood and walking among Australian older adults.* Int J Behav Nutr Phys Act, 2012. **9**: p. 133.

25. Villanueva, K., et al., *The impact of neighborhood walkability on walking: Does it differ across adult life stage and does neighborhood buffer size matter?* Health & Place, 2014. **25**: p. 43-46.

26. Cerin, E., et al., *Socioeconomic Status, Neighborhood Characteristics, and Walking Within the Neighborhood Among Older Hong Kong Chinese.* Journal of Aging & Health, 2013. **25**(8): p. 1425-1444.

27. Tsunoda, K., et al., *Associations of physical activity with neighborhood environments and transportation modes in older Japanese adults.* Preventive Medicine, 2012. **55**(2): p. 113-118.

28. Yeom, H.A., D. Jung, and M. Choi, *Adherence to physical activity among older adults using a geographic information system: Korean national health and nutrition examinations survey IV.* Asian Nursing Research, 2011. **5**(2): p. 118-127.

29. Morris, K.S., E. McAuley, and R.W. Motl, *Self-efficacy and environmental correlates of physical activity among older women and women with multiple sclerosis.* Health Educ Res, 2008. **23**(4): p. 744-52.

30. Hall, K.S. and E. McAuley, *Individual, social environmental and physical environmental barriers to achieving 10 000 steps per day among older women.* Health Education Research, 2010. **25**(3): p. 478-488.

31. Kaur, J., et al., *Predictors of Physical Inactivity Among Elderly Malaysians: Recommendations for Policy Planning.* Asia-Pacific Journal of Public Health, 2015. **27**(3): p. 314-322.

32. Bird, S., et al., *Factors influencing the physical activity levels of older people from culturally-diverse communities: an Australian experience.* Ageing & Society, 2009. **29**: p. 1275-1294.

33. Bird, S.R., et al., *Factors affecting walking activity of older people from culturally diverse groups: An Australian experience.* Journal of Science and Medicine in Sport, 2010. **13**(4): p. 417-423.

34. Procter-Gray, E., et al., *Variations in Community Prevalence and Determinants of Recreational and Utilitarian Walking in Older Age.* J Aging Res, 2015. **2015**: p. 382703.

35. Michael, Y.L., et al., *Physical Activity Resources and Changes in Walking in a Cohort of Older Men.* American Journal of Public Health, 2010. **100**(4): p. 654-660.

36. Jongeneel-Grimen, B., et al., *Change in Neighborhood Traffic Safety: Does It Matter in Terms of Physical Activity?* Plos One, 2013. **8**(5).

37. Jongeneel-Grimen, B., et al., *The relationship between physical activity and the living environment: A multi-level analyses focusing on changes over time in environmental factors.* Health & Place, 2014. **26**: p. 149-160.

38. Aird, R.L. and L. Buys, *Active Aging: Exploration into Self-Ratings of "Being Active," Out-of-Home Physical Activity, and Participation among Older Australian Adults Living in Four Different Settings.* J Aging Res, 2015. **2015**: p. 501823.

39. Arnadottir, S.A., E.D. Gunnarsdottir, and L. Lundin-Olsson, *Are rural older Icelanders less physically active than those living in urban areas? A population-based study.* Scandinavian Journal of Public Health, 2009. **37**(4): p. 409-417.

40. Asawachaisuwikrom, W., *Predictors of physical activity among older Thai adults*, in *Faculty of the Graduate School*. 2001, University of Texas, at Austin: Texas, United States of America.

41. Baceviciene, M. and J. Alisauskas, *Perceived constraints on exercise in the group of the elderly: a pilot study.* Central European Journal of Medicine, 2013. **8**(5): p. 689-695.

42. Böcker, L., P. van Amen, and M. Helbich, *Elderly travel frequencies and transport mode choices in Greater Rotterdam, the Netherlands.* Transportation, 2016: p. 1-22.

43. Carvalho Sampaio, R.A., et al., *Urban-rural differences in physical performance and health status among older Japanese community-dwelling women.* Journal of Clinical Gerontology and Geriatrics, 2012. **3**(4): p. 127-131.

44. Chad, K.E., et al., *Profile of physical activity levels in community-dwelling older adults.* Medicine and Science in Sports and Exercise, 2005. **37**(10): p. 1774-1784.

45. Chaudhury, H., et al., *Neighbourhood environment and physical activity in older adults.* Social Science and Medicine, 2016. **149**: p. 104-113.

46. Chen, T.A., et al., *Features of perceived neighborhood environment associated with daily walking time or habitual exercise: Differences across gender, age, and employment status in a community-dwelling population of Japan.* Environmental Health and Preventive Medicine, 2013. **18**(5): p. 368-376.

47. de Melo, L.L., et al., *Personal factors, perceived environment, and objectively measured walking in old age.* Journal of Aging & Physical Activity, 2010. **18**(3): p. 280-292.

48. Gallagher, N.A., et al., *Influences on Neighborhood Walking in Older Adults.* Research in Gerontological Nursing, 2012. **5**(4): p. 238-250.

49. Gomez, L.F., et al., *Built Environment Attributes and Walking Patterns Among the Elderly Population in Bogota.* American Journal of Preventive Medicine, 2010. **38**(6): p. 592-599.

50. Grant-Savela, S.D., *Active Living Among Older Residents of a Rural Naturally Occurring Retirement Community.* Journal of Applied Gerontology, 2010. **29**(5): p. 531-553.

51. Inoue, S., et al., *Perceived Neighborhood Environment and Walking for Specific Purposes Among Elderly Japanese.* Journal of Epidemiology, 2011. **21**(6): p. 481-490.

52. King, W.C., et al., *The relationship between convenience of destinations and walking levels in older women.* American Journal of Health Promotion, 2003. **18**(1): p. 74-82.

53. Koh, P.P., B.W. Leow, and Y.D. Wong, *Mobility of the elderly in densely populated neighbourhoods in Singapore.* Sustainable Cities and Society, 2015. **14**(1): p. 126-132.

54. Kolbe-Alexander, T.L., et al., *The relationship between the built environment and habitual levels of physical activity in South African older adults: a pilot study.* BMC Public Health, 2015. **15**: p. 518.

55. Lee, H.S. and E.Y. Park, *Associations of Neighborhood Environment and Walking in Korean Elderly Women: A Comparison between Urban and Rural Dwellers.* Asian Women, 2015. **31**(4): p. 1-21.

56. Lotfi, S. and M.J. Koohsari, *Neighborhood Walkability in a City within a Developing Country.* Journal of Urban Planning and Development-Asce, 2011. **137**(4): p. 402-408.

57. Maisel, J.L., *Impact of Older Adults' Neighborhood Perceptions on Walking Behavior.* Journal of Aging and Physical Activity, 2016. **24**(2): p. 247-255.

58. Mowen, A., et al., *The role of park proximity and social support in shaping park visitation, physical activity, and perceived health among older adults.* J Phys Act Health, 2007. **4**(2): p. 167-79.

59. Pelclová, J., et al., *Neighborhood Environment and Walking for Transport and Recreation in Central European Older Adults.* Acta Universitatis Palackianae Olomucensis. Gymnica, 2012. **42**(4): p. 49-56.

60. Persson, A. and A. While, *Physical activity among older people and related factors.* Health Education Journal, 2012. **71**(2): p. 144-153.

61. Salvador, E.P., R.S. Reis, and A.A. Florindo, *Practice of walking and its association with perceived environment among elderly Brazilians living in a region of low socioeconomic level.* International Journal of Behavioral Nutrition and Physical Activity, 2010. **7**.

62. Sewo Sampaio, P.Y., E. Ito, and R.A. Carvalho Sampaio, *The association of activity and participation with quality of life between Japanese older adults living in rural and urban areas.* Journal of Clinical Gerontology and Geriatrics, 2013. **4**(2): p. 51-56.

63. Shin, W.-H., B.-S. Kweon, and W.-J. Shin, *The distance effects of environmental variables on older African American women's physical activity in Texas.* Landscape and Urban Planning, 2011. **103**(2): p. 217-229.

64. Shores, K.A., et al., *Extra-Individual Correlates of Physical Activity Attainment in Rural Older Adults.* Journal of Rural Health, 2009. **25**(2): p. 211-218.

65. Tanaka, T., et al., *Comparison of objective physical activity, muscle strength, and depression among community-dwelling older women living in sloped versus non-sloped environments.* Journal of Nutrition, Health and Aging, 2016: p. 520-524.

66. Towne, S.D., Jr., et al., *Using Walk Score and Neighborhood Perceptions to Assess Walking Among Middle-Aged and Older Adults.* J Community Health, 2016. **41**(5): p. 977-88.

67. Wang, Z. and C. Lee, *Site and neighborhood environments for walking among older adults.* Health & Place, 2010. **16**(6): p. 1268-1279.

68. Wilcox, S., et al., *Psychosocial and perceived environmental correlates of physical activity in rural and older african american and white women.* J Gerontol B Psychol Sci Soc Sci, 2003. **58**(6): p. P329-37.

69. Macniven, R., et al., *Barriers and Enablers to Physical Activity Among Older Australians Who Want to Increase Their Physical Activity Levels.* Journal of Physical Activity & Health, 2014. **11**(7): p. 1420-1429.

70. Lim, K. and L. Taylor, *Factors associated with physical activity among older people—a population-based study.* Preventive Medicine, 2005. **40**(1): p. 33-40.

71. James, P., et al., *Urban Sprawl, Physical Activity, and Body Mass Index: Nurses' Health Study and Nurses' Health Study 11.* American Journal of Public Health, 2013. **103**(2): p. 369-375.

72. Troped, P.J., et al., *Relationships Between the Built Environment and Walking and Weight Status Among Older Women in Three US States.* Journal of Aging and Physical Activity, 2014. **22**(1): p. 114-125.

73. Piro, F.N., O. Noess, and B. Claussen, *Physical activity among elderly people in a city population: the influence of neighbourhood level violence and self perceived safety.* Journal of Epidemiology and Community Health, 2006. **60**(7): p. 626-632.

74. Pan, S.Y., et al., *Individual, social, environmental, and physical environmental correlates with physical activity among Canadians: a cross-sectional study.* Bmc Public Health, 2009. **9**.

75. McMurdo, M.E.T., et al., *Social, Environmental and Psychological Factors Associated with Objective Physical Activity Levels in the Over 65s.* Plos One, 2012. **7**(2).

76. Sniehotta, F.F., et al., *Psychological theory in an interdisciplinary context: psychological, demographic, health-related, social, and environmental correlates of physical activity in a representative cohort of community-dwelling older adults.* International Journal of Behavioral Nutrition & Physical Activity, 2013. **10**(1): p. 106-116.

77. Davis, M.G., et al., *Getting out and about in older adults: the nature of daily trips and their association with objectively assessed physical activity.* International Journal of Behavioral Nutrition & Physical Activity, 2011. **8**: p. 9p.

78. Fox, K.R., et al., *Neighbourhood deprivation and physical activity in UK older adults.* Health & Place, 2011. **17**(2): p. 633-640.

79. Thompson, J.L., et al., *Food shopping habits, physical activity and health-related indicators among adults aged >= 70 years.* Public Health Nutrition, 2011. **14**(9): p. 1640-1649.

80. Han, B.H., et al., *Correlates of Physical Activity Among Middle-Aged and Older Korean Americans at Risk for Diabetes.* Journal of Nursing Scholarship, 2016. **48**(1): p. 48-57.

81. Li, F.Z., et al., *Multilevel modelling of built environment characteristics related to neighbourhood walking activity in older adults.* Journal of Epidemiology and Community Health, 2005. **59**(7): p. 558-564.

82. Li, F.Z., K.J. Fisher, and R.C. Brownson, *A multilevel analysis of change in neighborhood walking activity in older adults.* Journal of Aging and Physical Activity, 2005. **13**(2): p. 145-159.

83. Michael, Y., et al., *Measuring the influence of built neighborhood environments on walking in older adults.* Journal of Aging and Physical Activity, 2006. **14**(3): p. 302-312.

84. Nagel, C.L., et al., *The relation between neighborhood built environment and walking activity among older adults.* American Journal of Epidemiology, 2008. **168**(4): p. 461-468.

85. Frank, L., et al., *Healthy Aging and Where You Live: Community Design Relationships With Physical Activity and Body Weight in Older Americans.* Journal of Physical Activity & Health, 2010. **7**: p. S82-S90.

86. Kerr, J., et al., *Assessing health-related resources in senior living residences.* Journal of Aging Studies, 2011. **25**(3): p. 206-214.

87. Carlson, J.A., et al., *Interactions between psychosocial and built environment factors in explaining older adults' physical activity.* Preventive Medicine, 2012. **54**(1): p. 68-73.

88. Bracy, N.L., et al., *Is the relationship between the built environment and physical activity moderated by perceptions of crime and safety?* International Journal of Behavioral Nutrition and Physical Activity, 2014. **11**(1).

89. Cain, K.L., et al., *Contribution of streetscape audits to explanation of physical activity in four age groups based on the Microscale Audit of Pedestrian Streetscapes (MAPS).* Social Science & Medicine, 2014. **116**: p. 82-92.

90. Carlson, J.A., et al., *Sociodemographic Moderators of Relations of Neighborhood Safety to Physical Activity.* Medicine & Science in Sports & Exercise, 2014. **46**(8): p. 1554-1563.

91. Ding, D., et al., *Neighborhood Environment and Physical Activity Among Older Adults: Do the Relationships Differ by Driving Status?* Journal of Aging & Physical Activity, 2014. **22**(3): p. 421-431.

92. McKee, G., P.M. Kearney, and R.A. Kenny, *The factors associated with self-reported physical activity in older adults living in the community.* Age Ageing, 2015. **44**(4): p. 586-92.

93. Murtagh, E.M., et al., *Prevalence and correlates of physical inactivity in community-dwelling older adults in Ireland.* PLoS One, 2015. **10**(2): p. e0118293.

94. Hannon, L., 3rd, P. Sawyer, and R.M. Allman, *Housing, the Neighborhood Environment, and Physical Activity among Older African Americans.* J Health Dispar Res Pract, 2012. **5**(3): p. 27-41.

95. Gauvin, L., et al., *Living in a Well-Serviced Urban Area Is Associated With Maintenance of Frequent Walking Among Seniors in the VoisiNuAge Study.* Journals of Gerontology Series B-Psychological Sciences and Social Sciences, 2012. **67**(1): p. 76-88.

96. Julien, D., et al., *Transit use and walking as potential mediators of the association between accessibility to services and amenities and social participation among urban-dwelling older adults: Insights from the VoisiNuAge study.* Journal of Transport & Health, 2015. **2**(1): p. 35-43.

97. Hirsch, J.A., et al., *Destinations That Older Adults Experience Within Their GPS Activity Spaces: Relation to Objectively Measured Physical Activity.* Environment and Behavior, 2016. **48**(1): p. 55-77.

98. Perry, C.K., et al., *Does neighborhood walkability moderate the effects of intrapersonal characteristics on amount of walking in post-menopausal women?* Health & Place, 2013. **21**: p. 39-45.

99. de Melo, L.L., *Perceived neighbourhood environment and health-related outcomes among older adults*, in *Faculty of Graduate Studies*. 2013, University of Manitoba: Winnipeg, Canada.

100. Zhang, Y., et al., *The Built Environment and Walking Activity of the Elderly: An Empirical Analysis in the Zhongshan Metropolitan Area, China.* Sustainability, 2014. **6**(2): p. 1076-1092.
