# Supplementary material for: Built environmental correlates of older adults’ total physical activity and walking: a systematic review and meta-analysis
Source: Int J Behav Nutr Phys Act. 2017 Aug 7;14:103. doi: 10.1186/s12966-017-0558-z (PMC5547528; doi:10.1186/s12966-017-0558-z)
Supplement: Supplementary file 3 — Associations between built environmental attributes and older adults’ objectively measured walking. (DOCX 16 kb) [file 12966_2017_558_MOESM3_ESM.docx]

**Table S2. Associations between built environmental attributes and older adults' objectively measured walking**

| **Environmental attribute** | **Objective walking** | | | | |
| --- | --- | --- | --- | --- | --- |
|  | P | Ø | N | *p*_a_ | D_a_ |
| Walkability | 2 | 0 | 0 | .006 | P |
| Residential density/urbanisation | 0 | 4 | 0 | 1.00 | Ø |
| Street connectivity | 1 | 1 | 0 | .192 | Ø |
| *Access to/availability of services & destinations* | | | | | |
| Overall access to services & destinations | 1.63 | 4.37 | 0 | .123 | Ø |
| Land-use mix—destination diversity | 0.17 | 2.83 | 0 | .723 | Ø |
| Shops/commercial | 0.38 | 8.62 | 0 | .727 | Ø |
| Food outlets | 0 | 5 | 0 | 1.00 | Ø |
| Government/finance services | 0.17 | 0.83 | 0 | .426 | Ø |
| Education | 0 | 5 | 0 | 1.00 | Ø |
| Health & aged care | 0 | 2 | 0 | 1.00 | Ø |
| Religious | 0 | 3 | 0 | 1.00 | Ø |
| Public transport | 0 | 2 | 0 | 1.00 | Ø |
| Parks/public open space | 0.75 | 5.25 | 0 | .500 | Ø |
| Recreational facilities | 0 | 3 | 0 | 1.00 | Ø |
| Social recreational facilities | 0 | 4 | 0 | 1.00 | Ø |
| Other destinations |  |  |  |  |  |
| *Infrastructure & streetscape* | | | | | |
| Overall cycle/walk-friendly infrastructure | 0 | 2 | 0 | 1.00 | Ø |
| Walk-friendly infrastructure | 1 | 1 | 0 | .400 | Ø |
| Cycle-friendly infrastructure | 0 | 0 | 0 | - | - |
| No physical barriers to walking (e.g., hills) | 0 | 0 | 0 | - | - |
| Pavement/footpath quality | 0 | 0 | 0 | - | - |
| Street lighting | 0 | 0 | 0 | - | - |
| Greenery & aesthetically pleasing scenery | 0 | 3 | 0 | 1.00 | Ø |
| Pollution (air) | 0 | 0 | 0 | - | - |
| *Safety* | | | | | |
| Traffic/pedestrian safety | 1 | 1 | 0 | .234 | Ø |
| Crime/personal safety | 0 | 1 | 0 | 1.00 | Ø |
| *Notes:* P = positive association; Ø = nil association; N = negative association; p = *p*-value; D = direction of association supported by the data; subscript “a” = fully adjusted (for sample size and article quality). In bold font: statistically significant evidence of a directional association that has been sufficiently studied (i.e., ≥5 findings reported on specific combinations of environmental exposure and physical activity variables). | | | | | |
