# Supplementary material for: Built environmental correlates of older adults’ total physical activity and walking: a systematic review and meta-analysis
Source: Int J Behav Nutr Phys Act. 2017 Aug 7;14:103. doi: 10.1186/s12966-017-0558-z (PMC5547528; doi:10.1186/s12966-017-0558-z)
Supplement: Supplementary file 4 — Meta-analytic results of significance of associations between built environmental correlates of older adults’ PA by outcome and type of adjustment for article characteristics. (DOCX 19 kb) [file 12966_2017_558_MOESM4_ESM.docx]

**Table S4. Meta-analytic-results of significance of associations between built environmental correlates of older adults' PA by outcome and type of adjustment for article characteristics**

| **Environmental attribute** | **Total PA** | | | | | **Total walking** | | | | |  | **Objective total PA** | | | | | **Self-report total PA** | | | | |
| --- | --- | --- | --- | --- | --- | --- | --- | --- | --- | --- | --- | --- | --- | --- | --- | --- | --- | --- | --- | --- | --- |
|  | *p*_a_ | *p*_sa_ | *p*_q_ | *p*_u_ | Diff’ | *p*_a_ | *p*_sa_ | *p*_q_ | *p*_u_ | Diff’ |  | *p*_a_ | *p*_sa_ | *p*_q_ | *p*_u_ | Diff’ | *p*_a_ | *p*_sa_ | *p*_q_ | *p*_u_ | Diff’ |
| Walkability | <.001 | <.001 | <.001 | <.001 | No | .001 | <.001 | .004 | .001 | No |  | <.001 | <.001 | <.001 | <.001 | No | <.001 | <.001 | <.001 | <.001 | No |
| Residential density/urbanisation | .394 | .528 | .436 | .688 | No | .036 | .033 | .045 | **.055** | **Yes** |  | .377 | .413 | .392 | .460 | No | .240 | .361 | .260 | .463 | No |
| Street connectivity | .094 | **.042** | .061 | **.012** | **Yes** | .185 | .133 | .124 | **.044** | **Yes** |  | .262 | .137 | .215 | .077 | No | .215 | .151 | .159 | .068 | No |
| *Access to/availability of services & destinations* | | | | | | | | | | | | | | | | | | | | | |
| Overall access to services/destinations | <.001 | <.001 | <.001 | <.001 | No | .009 | .009 | .008 | .008 | No |  | .006 | .003 | .008 | .005 | No | .003 | .003 | .003 | .002 | No |
| Land-use mix—destination diversity | .148 | .202 | .119 | .120 | No | .439 | .381 | .506 | .535 | No |  | .019 | .021 | .025 | .045 | No | .884 | .882 | .983 | .793 | No |
| Shops/commercial | .006 | .001 | .012 | .008 | No | .001 | <.001 | .002 | .001 | No |  | .507 | .512 | .525 | .564 | No | .002 | <.001 | .003 | .003 | No |
| Food outlets | .932 | .899 | .995 | .898 | No | .873 | .840 | .990 | .799 | No |  | 1.00 | 1.00 | 1.00 | 1.00 | No | .884 | .859 | .992 | .842 | No |
| Government/finance services | .834 | .883 | .811 | .822 | No | 1.00 | 1.00 | 1.00 | 1.00 | No |  | .377 | .820 | .726 | .740 | No | 1.00 | 1.00 | 1.00 | 1.00 | No |
| Education | .765 | .805 | .774 | .821 | No | .826 | .822 | .833 | .839 | No |  | .818 | .884 | .818 | .861 | No | .845 | .842 | .861 | .880 | No |
| Health & aged care | .275 | .185 | .268 | .179 | No | .191 | .179 | .149 | .089 | No |  | .612 | .526 | .634 | .621 | No | .206 | .203 | .169 | .124 | No |
| Religious | 1.00 | 1.00 | 1.00 | 1.00 | No | 1.00 | 1.00 | 1.00 | 1.00 | No |  | 1.00 | 1.00 | 1.00 | 1.00 | No | 1.00 | 1.00 | 1.00 | 1.00 | No |
| Public transport | .013 | .008 | .019 | .019 | No | .011 | .012 | .015 | .023 | No |  | .520 | .512 | .509 | .562 | No | .006 | .007 | .007 | .011 | No |
| Parks/public open space | .002 | <.001 | .002 | .002 | No | .014 | .018 | .018 | .020 | No |  | .296 | .282 | .315 | .333 | No | .002 | .004 | .004 | .002 | No |
| Recreational facilities | <.001 | <.001 | <.001 | <.001 | No | .135 | .104 | .143 | .123 | No |  | .056 | .063 | **.050** | .051 | **Yes** | .002 | <.001 | .004 | <.001 | No |
| Social recreational facilities | .105 | **.050** | .123 | .092 | **Yes** | .413 | .258 | .448 | .342 | No |  | .118 | .066 | .136 | .111 | No | .432 | .296 | .473 | .407 | No |
| Other destinations | 1.00 | 1.00 | 1.00 | 1.00 | No | - | - | - | - | - |  | 1.00 | 1.00 | 1.00 | 1.00 | No | - | - | - | - | - |
| *Infrastructure & streetscape* | | | | | | | | | | | | | | | | | | | | | |
| Overall cycle/walk-friendly infrastructure | .612 | .569 | .579 | .502 | No | 1.00 | 1.00 | 1.00 | 1.00 | No |  | .529 | .441 | .492 | .382 | No | 1.00 | 1.00 | 1.00 | 1.00 | No |
| Walk-friendly infrastructure | .009 | .005 | .006 | .006 | No | .042 | .008 | **.057** | .019 | **Yes** |  | .031 | .025 | .026 | .016 | No | .059 | **.036** | .072 | .061 | **Yes** |
| Cycle-friendly infrastructure | 1.00 | 1.00 | 1.00 | 1.00 | No | 1.00 | 1.00 | 1.00 | 1.00 | No |  | 1.00 | 1.00 | 1.00 | 1.00 | No | 1.00 | 1.00 | 1.00 | 1.00 | No |
| No physical barriers to walking (e.g., hills) | .208 | .347 | .214 | .362 | No | .384 | .474 | .390 | .495 | No |  | .135 | .260 | .123 | .123 | No | .631 | .698 | .684 | .684 | No |
| Pavement/footpath quality | .155 | .269 | .142 | .215 | No | .169 | .203 | .148 | .138 | No |  | .050 | .050 | .050 | .050 | No | .384 | .617 | .355 | .514 | No |
| Street lighting | .059 | **.044** | .051 | **.032** | **Yes** | .042 | .024 | .034 | .017 | No |  | - | - | - | - | - | .059 | **.044** | .051 | **.032** | **Yes** |
| Greenery & aesthetically pleasing scenery | .004 | <.001 | .005 | <.001 | No | .002 | <.001 | .003 | <.001 | No |  | .741 | .682 | .712 | .619 | No | .001 | <.001 | .003 | <.001 | No |
| Pollution (air) | 1.00 | 1.00 | 1.00 | 1.00 | No | 1.00 | 1.00 | 1.00 | 1.00 | No |  | - | - | - | - | - | 1.00 | 1.00 | 1.00 | 1.00 | No |
| *Safety* | | | | | | | | | | | | | | | | | | | | | |
| Traffic/pedestrian safety | .463 | .332 | .429 | .256 | No | .705 | .500 | .680 | .443 | No |  | .408 | .370 | .381 | .304 | No | .737 | .564 | .711 | .495 | No |
| Crime/personal safety | <.001 | <.001 | <.001 | <.001 | No | .027 | .006 | .029 | .009 | No |  | .063 | **.042** | .067 | .051 | **Yes** | .001 | .001 | .001 | <.001 | No |
| *Notes:* *p* = *p* value; Diff’ = difference in conclusions between types of adjustment of article characteristics; subscript ‘a’ = fully adjusted (for sample size and article quality); subscript ‘sa’ = adjusted for sample size; subscript ‘q’ = adjusted for article quality; subscript ‘u’ = unadjusted. | | | | | | | | | | | | | | | | | | | | | |
