# Supplementary material for: Built environmental correlates of older adults’ total physical activity and walking: a systematic review and meta-analysis
Source: Int J Behav Nutr Phys Act. 2017 Aug 7;14:103. doi: 10.1186/s12966-017-0558-z (PMC5547528; doi:10.1186/s12966-017-0558-z)
Supplement: Supplementary file 5 — Overview of moderating effects examined in the association between environmental attributes and older adults’ total PA. (DOCX 214 kb) [file 12966_2017_558_MOESM5_ESM.docx]

**Table S5. Overview of moderating findings examined in the association between environmental attributes and older adults' total PA**

| **Moderator** | **Article (n) & reference** | **Environmental attribute** | **Total PA** | | | | **Moderating association/s on specific environmental attribute-PA combinations** |
| --- | --- | --- | --- | --- | --- | --- | --- |
| *Individual factors* |  |  | ∅^1^ | Article ref’ | Sig^1^ | Article ref’ | P (positive), Ø (nil), N (negative) |
| **Age** | 4 [1-4] | Walkability | 1 | [4] | 0 | - | - |
|  |  | Residential density/urbanisation | 2 | [3] | 0 | - | - |
|  |  | *Access to/availability of services & destinations* | |  |  |  |  |
|  |  | Land-use mix—destination diversity | 0 | - | 1 | [2] | 1. 65-79y (P), ≥80y (Ø) |
|  |  | Parks/public open space | 1 | [2] | 1 | [2] | 1. ≥80y (P), 65-79y (Ø) |
|  |  | Recreational facilities | 2 | [2] | 5 | [1, 2] | 1. 75.5y (P) & 80y (P) @ 400m, 69.3y (Ø) @ 400m ^[1]^; 2. Skating rink: 65-79y (P), ≥80y (Ø); 3. Swimming pool: ≥80y (P), 65-79y (Ø); 4. Tennis court: ≥80y (P), 65-79y (Ø); 5. Dance studio: ≥80y (P), 65-79y (Ø) ^[2]^ |
|  |  | *Infrastructure & streetscape* |  |  |  |  |  |
|  |  | Walk-friendly infrastructure | 1 | [2] | 1 | [2] | 1. 65-79y (N), ≥80y (Ø) |
|  |  | Cycle-friendly infrastructure | 1 | [2] | 0 | - | - |
|  |  | No physical barriers to walking (e.g., hills) | 1 | [2] | 0 | - | - |
|  |  | Street lighting | 1 | [2] | 0 | - | - |
|  |  | Greenery & aesthetically pleasing scenery | 2 | [2] | 0 | - | - |
|  |  | *Safety* |  |  |  |  |  |
|  |  | Traffic/pedestrian safety | 1 | [2] | 0 | - | - |
|  |  | Crime/personal safety | 1 | [2] | 2 | [2] | 1. Unattended dogs: ≥80y (N), 65-79y (Ø); 2. See active people: ≥80y (P), 65-79y (Ø) |
| **Sex** | 7 [5-11] | Residential density/urbanisation | 3 | [5, 8, 9] | 1 | [12] | 1. Men (N), Women (P) |
|  |  | Street connectivity | 4 | [5, 8, 10] | 1 | [5] | 1. Men @ 1000m (P), all other findings across sexes (Ø) |
|  |  | *Access to/availability of services & destinations* | |  |  |  |  |
|  |  | Overall services & destinations | 7 | [5, 7, 8, 10] | 2 | [6] | 1. Age appropriate facilities: Women (P), Men (Ø); 2. Somewhere nice to walk: Men (P), Women (Ø) |
|  |  | Land-use mix—destination diversity | 2 | [7] | 0 | - | - |
|  |  | Shops/commercial destinations | 5 | [7, 10] | 1 | [9] | 1. Women (P), Men (Ø) |
|  |  | Food outlets | 3 | [7, 10] | 0 | - | - |
|  |  | Government/finance services | 3 | [7, 10] | 0 | - | - |
|  |  | Education | 1 | [5] | 0 | - | - |
|  |  | Health & aged care | 3 | [7, 10] | 1 | [10] | 1. Walk time to health clinic: Women (P), Men (Ø) |
|  |  | Religious | 1 | [10] | 0 | - | - |
|  |  | Public transport | 4 | [6, 9, 10] | 0 | - | - |
|  |  | Parks/public open space | 4 | [5, 10] | 1 | [10] | 1. Meeting square present: Women (P), Men (Ø) |
|  |  | Recreational facilities | 4 | [9, 10] | 2 | [10] | 1. Walk time to soccer fields: Men (P), Women (Ø); 2. Soccer fields present: Men (P), Women (Ø) |
|  |  | Social recreational facilities | 4 | [7, 10] | 1 | [6] | 1. Social and leisure activities: Men (P), Women (Ø) |
|  |  | *Infrastructure & streetscape* |  |  |  |  |  |
|  |  | Walk-friendly infrastructure | 1 | [9] | 0 | - | - |
|  |  | Cycle-friendly infrastructure | 1 | [9] | 0 | - | - |
|  |  | No physical barriers to walking (e.g., hills) | 1 | [10] | 1 | [5] | 1. Land slope: Men @ 1000m (Ø), all other findings across sexes (N) |
|  |  | Sidewalk quality | 1 | [10] | 0 | - | - |
|  |  | Street lighting | 1 | [10] | 0 | - | - |
|  |  | Greenery & aesthetically pleasing scenery | 3 | [8-10] | 0 | - | - |
|  |  | Pollution (air) | 2 | [10] | 0 | - | - |
|  |  | *Safety* |  |  |  |  |  |
|  |  | Traffic/pedestrian safety | 4 | [9, 10, 13] | 0 | - | - |
|  |  | Crime/personal safety | 6 | [6, 9, 10, 13] | 3 | [9, 11] | 1. Men (P), Women (Ø) ^[9]^; 2. Walking alone at night: Women (N), Men (Ø); 3. Neighbourhood violence: Men (N), Women (Ø) ^[11]^ |
| **Health status/ functionality** | 7 [1, 4, 14-18] | Walkability | 2 | [4, 14] | 0 | - | - |
|  |  | Residential density/urbanisation | 2 | [16] | 0 | - | - |
|  |  | Street connectivity | 2 | [16] | 0 | - | - |
|  |  | *Access to/availability of services & destinations* | |  |  |  |  |
|  |  | Overall services & destinations | 1 1 | [15] [16] | 1 | [17] | 1. Mobility-limited (P), Non-mobility-limited (Ø) |
|  |  | Land-use mix—destination diversity | 2 | [16] | 0 | - | - |
|  |  | Shops/commercial destinations | 1 | [16] | 0 | - | - |
|  |  | Public transport | 1 | [15] | 0 | - | - |
|  |  | Parks/public open space | 2 | [1, 15] | 0 | - | - |
|  |  | Recreational facilities | 1 | [18] | 0 | - | - |
|  |  | Social recreational facilities | 1 | [1] | 0 | - | - |
|  |  | *Infrastructure & streetscape* |  |  |  |  |  |
|  |  | Walk-friendly infrastructure | 1 | [15] | 0 | - | - |
|  |  | Cycle-friendly infrastructure | 1 | [16] | 0 | - | - |
|  |  | No physical barriers to walking (e.g., hills) | 1 | [15] | 0 | - | - |
|  |  | Greenery & aesthetically pleasing scenery | 3 | [15, 16] | 0 | - | - |
|  |  | *Safety* |  |  |  |  |  |
|  |  | Traffic/pedestrian safety | 3 | [15, 16] | 0 | - | - |
|  |  | Crime/personal safety | 3 | [15, 16] | 0 | - | - |
| **Other sociodemographics (e.g., education, ethnicity)** | 6 [1, 4, 8, 13, 19, 20] | Walkability | 4 | [4] | 0 | - | - |
|  |  | Residential density/urbanisation | 1 | [8] | 0 | - | - |
|  |  | Street connectivity | 2 | [1, 8] | 0 | - | - |
|  |  | *Access to/availability of services & destinations* | |  |  |  |  |
|  |  | Overall services & destinations | 3 | [8] | 0 | - | - |
|  |  | Parks/public open space | 2 | [19, 20] | 1 | [19] | 1. Chinese (P), Korean (P), Japanese (N), Filipino (Ø), Vietnamese (Ø) |
|  |  | Recreational facilities | 0 | - | 1 | [1] | 1. Up to primary (P), Secondary or higher (Ø) @ 400m |
|  |  | *Infrastructure & streetscape* |  |  |  |  |  |
|  |  | Walk-friendly infrastructure | 1 | [20] | 0 | - | - |
|  |  | Street lighting | 1 | [20] | 0 | - | - |
|  |  | Greenery & aesthetically pleasing scenery | 1 | [8] | 0 | - | - |
|  |  | *Safety* |  |  |  |  |  |
|  |  | Traffic/pedestrian safety | 5 | [13, 20] | 0 | - | - |
|  |  | Crime/personal safety | 5 | [13, 19, 20] | 1 | [19] | 1. Filipino (P), all other findings across ethnicities (Ø) |
| **Psychosocial factors** | 3 [15, 21, 22] | Street connectivity | 3 | [21] | 0 | - | - |
|  |  | *Access to/availability of services & destinations* | |  |  |  |  |
|  |  | Overall services & destinations | 1 | [15] | 0 | - | - |
|  |  | Public transport | 1 | [15] | 0 | - | - |
|  |  | Parks/public open space | 4 | [15, 22] | 0 | - | - |
|  |  | *Infrastructure & streetscape* |  |  |  |  |  |
|  |  | Walk-friendly infrastructure | 4 | [15, 22] | 0 | - | - |
|  |  | No physical barriers to walking (e.g., hills) | 1 | [15] | 0 | - | - |
|  |  | Greenery & aesthetically pleasing scenery | 7 | [15, 21, 22] | 1 | [22] | 1. Few barriers (P), High barriers (N) |
|  |  | Traffic/pedestrian safety | 5 | [15, 21] | 0 | - | - |
|  |  | Crime/personal safety | 5 | [15, 21] | 0 | - | - |
| **Driving status/car ownership** | 2 [1, 23] | Walkability | 2 | [23] | 0 | - | - |
|  |  | Residential density/urbanisation | 2 | [23] | 0 | - | - |
|  |  | Street connectivity | 2 | [23] | 0 | - | - |
|  |  | *Access to/availability of services & destinations* | |  |  |  |  |
|  |  | Overall services & destinations | 2 | [23] | 0 | - | - |
|  |  | Land-use mix—destination diversity | 2 | [23] | 0 | - | - |
|  |  | Public transport | 3 | [1, 23] | 0 | - | - |
|  |  | Parks/public open space | 2 | [23] | 0 | - | - |
|  |  | Recreational facilities | 0 | - | 1 | [1] | 1. No car (P), Own car (Ø) @ 400m |
|  |  | *Infrastructure & streetscape* |  |  |  |  |  |
|  |  | Overall cycle/walk-friendly infrastructure | 2 | [23] | 0 | - | - |
|  |  | Greenery & aesthetically pleasing scenery | 2 | [23] | 0 | - | - |
|  |  | *Safety* |  |  |  |  |  |
|  |  | Traffic/pedestrian safety | 4 | [23] | 0 | - | - |
|  |  | Crime/personal safety | 2 | [23] | 0 | - | - |
| **Duration of residency** | 1 [5] | Residential density/urbanisation | 1 | [5] | 0 | - | - |
|  |  | Street connectivity | 1 | [5] | 1 | [5] | 1. <50y @ 1000m (P), ≥50y @ 250m (N), all other findings across length of residencies (Ø) |
|  |  | *Access to/availability of services & destinations* | |  |  |  |  |
|  |  | Overall services & destinations | 1 | [5] | 0 | - | - |
|  |  | Education | 1 | [5] | 0 | - | - |
|  |  | Parks/public open space | 1 | [5] | 0 | - | - |
|  |  | *Infrastructure & streetscape* |  |  |  |  |  |
|  |  | No physical barriers to walking (e.g., hills) | 0 | - | 1 | [5] | 1. ≥50y (N), <50y (Ø) |
| *Environmental factors* |  |  |  |  |  |  |  |
| **Area-level income/SES** | 5 [13, 24-27] | Walkability | 0 | - | 3 | [24, 27] | 1. Low income (P), High income (Ø); 2. Low income (P), High income (Ø) ^[24]^; 3. Low social vulnerability (P) ^[27]^ |
|  |  | Residential density/urbanisation | 2 | [26] | 0 | - | - |
|  |  | Street connectivity | 2 | [26] | 0 | - | - |
|  |  | *Access to/availability of services & destinations* | |  |  |  |  |
|  |  | Overall services & destinations | 2 | [26] | 0 | - | - |
|  |  | Land-use mix—destination diversity | 2 | [26] | 0 | - | - |
|  |  | Parks/public open space | 0 | - | 2 | [25] | 1. High SES (P), Low SES (Ø) @ 0.125 mile; 2. High SES (P), Low SES (Ø) @ 0.5 mile |
|  |  | *Infrastructure & streetscape* |  |  |  |  |  |
|  |  | Overall cycle/walk-friendly infrastructure | 2 | [26] | 0 | - | - |
|  |  | Greenery & aesthetically pleasing scenery | 2 | [26] | 0 | - | - |
|  |  | *Safety* |  |  |  |  |  |
|  |  | Traffic/pedestrian safety | 6 | [13, 26] | 0 | - | - |
|  |  | Crime/personal safety | 2 | [13, 26] | 1 | [26] | 1. Low SES (P), High SES (Ø) |
| **Residential density/ urbanisation** | 6 [28-33] | Residential density/urbanisation | 3 | [28, 29] | 1 | [32] | 1. Urban (P) |
|  |  | *Access to/availability of services & destinations* | |  |  |  |  |
|  |  | Overall services & destinations | 1 | [31] | 2 | [33] | 1. Total facilities: 90.1-95.1^th^ percentile (P), 95.1-100^th^ percentile (P), all other percentiles (Ø); 2. Services: 95.1-100^th^ percentile (P), all other percentiles (Ø) |
|  |  | Shops/commercial destinations | 2 | [30, 33] | 1 | [33] | 1. 90.1-95.1^th^ percentile (P), 95.1-100^th^ percentile (P), all other percentiles (Ø) |
|  |  | Food outlets | 0 | - | 2 | [33] | 1. Fast-food outlets: 0-20^th^ percentile (P), 90.1-95.1th percentile (P), 95.1-100^th^ percentile (P), all other percentiles (Ø); 2. Restaurants: 90.1-95.1^th^ percentile (P), 95.1-100^th^ percentile (P), all other percentiles (Ø) |
|  |  | Education | 0 | - | 1 | [33] | 1. 95.1-100^th^ percentile (P), all other percentiles (Ø) |
|  |  | Parks/public open space | 0 | - | 1 | [30] | 1. Rural (P), Urban (Ø) |
|  |  | Recreational facilities | 0 | - | 1 | [33] | 1. 90.1-95.1^th^ percentile (P), 95.1-100^th^ percentile (P), all other percentiles (Ø) |
|  |  | *Infrastructure & streetscape* |  |  |  |  |  |
|  |  | Street lighting | 1 | [30] | 0 | - | - |
|  |  | Greenery & aesthetically pleasing scenery | 0 | - | 1 | [30] | 1. Rural (P), Urban (Ø) |
|  |  | *Safety* |  |  |  |  |  |
|  |  | Traffic/pedestrian safety | 1 | [30] | 0 | - | - |
|  |  | Crime/personal safety | 0 | - | 2 | [30, 31] | 1. Urban (P), Rural (Ø) ^[30]^; 2. Rural (P), Suburban (Ø), Urban (Ø) ^[31]^ |
| **Intervention group/setting** | 2 [15, 34] | *Access to/availability of services & destinations* | |  |  |  |  |
|  |  | Overall services & destinations | 1 | [15] | 0 | - | - |
|  |  | Parks/public open space | 2 | [15, 34] | 0 | - | - |
|  |  | Public transport | 1 | [15] | 0 | - | - |
|  |  | *Infrastructure & streetscape* |  |  |  |  |  |
|  |  | Overall cycle/walk-friendly infrastructure | 0 | - | 1 | [34] | 1. Pre-intervention (Ø), Post-intervention (P) |
|  |  | Walk-friendly infrastructure | 1 | [15] | 0 | - | - |
|  |  | No physical barriers to walking (e.g., hills) | 1 | [15] | 1 | [34] | 1. Pre-intervention (P), Post-intervention (Ø) |
|  |  | Sidewalk quality | 1 | [34] | 0 | - | - |
|  |  | Greenery & aesthetically pleasing scenery | 2 | [15] | 0 | - | - |
|  |  | *Safety* |  |  |  |  |  |
|  |  | Traffic/pedestrian safety | 2 | [15] | 0 | - | - |
|  |  | Crime/personal safety | 2 | [15] | 1 | [34] | 1. 1. Pre-intervention (Ø), Post-intervention (P) |
| **Infrastructure & streetscape aspects (e.g., walkability, street connectivity)** | 3 [15, 35, 36] | *Access to/availability of services & destinations* | |  |  |  |  |
|  |  | Overall services & destinations | 1 | [15] | 0 | - | - |
|  |  | Public transport | 1 | [15] | 0 | - | - |
|  |  | Parks/public open space | 1 | [15] | 0 | - | - |
|  |  | Recreational facilities | 1 | [36] | 0 | - | - |
|  |  | *Infrastructure & streetscape* | | | | | |
|  |  | Walk-friendly infrastructure | 1 | [15] | 0 | - | - |
|  |  | No physical barriers to walking (e.g., hills) | 1 | [15] | 0 | - | - |
|  |  | Greenery & aesthetically pleasing scenery | 2 | [15] | 0 | - | - |
|  |  | *Safety* |  |  |  |  |  |
|  |  | Traffic/pedestrian safety | 5 | [15, 35, 36] | 0 | - | - |
|  |  | Crime/personal safety | 3 | [15, 35] | 0 | - | - |
| **Buffer size (e.g., 400m)** | 6 [1, 5, 25, 37-39] | Residential density/urbanisation | 5 | [1, 5] | 0 | - | - |
|  |  | Street connectivity | 9 | [5, 37, 38] | 3 | [5, 38] | 1. 250m + ≥50y (N), all other 250m-related findings (Ø); 2. 1000m + men (P) & 1000m + <50y (P), all other 1000m-related findings (Ø) ^[5]^; 3. 0.5 mile (P), 0.25 mile (Ø) ^[38]^ |
|  |  | *Access to/availability of services & destinations* | |  |  |  |  |
|  |  | Overall services & destinations | 7 | [5, 7, 38, 39] | 4 | [38, 39] | 1. 0.25 mile (P), 0.5 mile (Ø); 2. 0.5 mile (P), 0.25 mile (Ø) ^[38]^; 3. 800m = +ve, all other buffers = Ø; 4. 800m (P), all other findings across buffers (Ø) ^[39]^ |
|  |  | Land-use mix—destination diversity | 5 | [7, 37, 39] | 1 | [39] | 1. Pedestrian & bicycling activity space (P), all other findings across buffers (Ø) |
|  |  | Shops/commercial destinations | 14 | [1, 7, 37-39] | 2 | [38, 39] | 1. 0.5 mile (P), 0.25 mile (Ø) ^[38]^; 2. Daily path area (P), all other findings across buffers (Ø) ^[39]^ |
|  |  | Food outlets | 5 | [1, 7, 39] | 0 | - | - |
|  |  | Health & aged care | 6 | [7, 39] | 0 | - | - |
|  |  | Government/finance services | 3 | [1, 7] | 2 | [39] | 1. Daily path area (P) with total PA, all other buffers (Ø); 2. Daily path area (P) with total objective walking (P), all other findings across buffers (Ø) |
|  |  | Education | 7 | [5, 37, 39] | 1 | [39] | 1. Daily path area (P), all other findings across buffers (Ø) |
|  |  | Religious | 3 | [37, 39] | 0 | - | - |
|  |  | Public transport | 3 | [1, 38] | 1 | [38] | 1. 0.5 mile (P), 0.25 mile (Ø) |
|  |  | Parks/public open space | 9 | [1, 5, 37, 39] | 2 | [25] | 1. 0.125 mile + High SES (P), all other findings across buffers (Ø); 2. 0.5 mile + High SES (P), all other findings across buffers (Ø) |
|  |  | Recreational facilities | 3 | [25, 39] | 1 | [1] | 1. 400m (P), 1km (Ø) |
|  |  | Social recreational facilities | 6 | [1, 7, 39] | 1 | [7] | 1. 800m (P), 400m (Ø) |
|  |  | *Infrastructure & streetscape* |  |  |  |  |  |
|  |  | Walk-friendly infrastructure | 4 | [37, 38] | 0 | - | - |
|  |  | No physical barriers to walking (e.g., hills) | 0 | - | 3 | [5] | 1. 250m + Men (P), 250m + Women (P), 250m + Rural (N), all other 250m-related findings (Ø); 2. 500m + Men (N), 500m + Women (N), 500m + Rural (N), all other 500m-related findings (Ø); 3. 1000m + Women (N), 1000m + Rural (N), 1000m + ≥50y (N), all other 1000m-related findings (Ø) |
|  |  | Greenery & aesthetically pleasing scenery | 2 | [37] | 0 | - | - |
|  |  | *Safety* |  |  |  |  |  |
|  |  | Traffic/pedestrian safety | 9 | [38] | 0 | - | - |
| *Notes*: ∅ = Number of non-significant interactions/findings; S = Number of significant interactions/findings. ^1^Articles that examined two and/or three factor interactions are summarised under each type of moderator considered so the number of interactions are counted multiple times (e.g., [8]). | | | | | | | |

**References**

1. Cerin, E., et al., *Associations of objectively-assessed neighborhood characteristics with older adults’ total physical activity and sedentary time in an ultra-dense urban environment: Findings from the ALECS study.* Health and Place, 2016. **42**: p. 1-10.

2. Chad, K.E., et al., *Profile of physical activity levels in community-dwelling older adults.* Medicine and Science in Sports and Exercise, 2005. **37**(10): p. 1774-1784.

3. James, P., et al., *Urban Sprawl, Physical Activity, and Body Mass Index: Nurses' Health Study and Nurses' Health Study 11.* American Journal of Public Health, 2013. **103**(2): p. 369-375.

4. Perry, C.K., et al., *Does neighborhood walkability moderate the effects of intrapersonal characteristics on amount of walking in post-menopausal women?* Health & Place, 2013. **21**: p. 39-45.

5. Hanibuchi, T., et al., *Neighborhood built environment and physical activity of Japanese older adults: results from the Aichi Gerontological Evaluation Study (AGES).* BMC Public Health, 2011. **11**: p. 657.

6. Jefferis, B.J., et al., *Adherence to physical activity guidelines in older adults, using objectively measured physical activity in a population-based study.* BMC Public Health, 2014. **14**(1).

7. Nathan, A., et al., *Access to commercial destinations within the neighbourhood and walking among Australian older adults.* Int J Behav Nutr Phys Act, 2012. **9**: p. 133.

8. Chen, T.A., et al., *Features of perceived neighborhood environment associated with daily walking time or habitual exercise: Differences across gender, age, and employment status in a community-dwelling population of Japan.* Environmental Health and Preventive Medicine, 2013. **18**(5): p. 368-376.

9. Inoue, S., et al., *Perceived Neighborhood Environment and Walking for Specific Purposes Among Elderly Japanese.* Journal of Epidemiology, 2011. **21**(6): p. 481-490.

10. Salvador, E.P., R.S. Reis, and A.A. Florindo, *Practice of walking and its association with perceived environment among elderly Brazilians living in a region of low socioeconomic level.* International Journal of Behavioral Nutrition and Physical Activity, 2010. **7**.

11. Piro, F.N., O. Noess, and B. Claussen, *Physical activity among elderly people in a city population: the influence of neighbourhood level violence and self perceived safety.* Journal of Epidemiology and Community Health, 2006. **60**(7): p. 626-632.

12. Murtagh, E.M., et al., *Prevalence and correlates of physical inactivity in community-dwelling older adults in Ireland.* PLoS One, 2015. **10**(2): p. e0118293.

13. Carlson, J.A., et al., *Sociodemographic Moderators of Relations of Neighborhood Safety to Physical Activity.* Medicine & Science in Sports & Exercise, 2014. **46**(8): p. 1554-1563.

14. Cerin, E., et al., *Associations of neighborhood environment with brain imaging outcomes in the AIBL cohort.* Alzheimers Dement, 2016.

15. Merom, D., et al., *Neighborhood walkability, fear and risk of falling and response to walking promotion: The Easy Steps to Health 12-month randomized controlled trial.* Prev Med Rep, 2015. **2**: p. 704-10.

16. Satariano, W.A., et al., *Lower-Body Function, Neighborhoods, and Walking in an Older Population.* American Journal of Preventive Medicine, 2010. **38**(4): p. 419-428.

17. Gallagher, N.A., et al., *Influences on Neighborhood Walking in Older Adults.* Research in Gerontological Nursing, 2012. **5**(4): p. 238-250.

18. Macniven, R., et al., *Barriers and Enablers to Physical Activity Among Older Australians Who Want to Increase Their Physical Activity Levels.* Journal of Physical Activity & Health, 2014. **11**(7): p. 1420-1429.

19. Li, Y., D. Kao, and T.Q. Dinh, *Correlates of Neighborhood Environment With Walking Among Older Asian Americans.* Journal of Aging & Health, 2015. **27**(1): p. 17-34.

20. Wilcox, S., et al., *Psychosocial and perceived environmental correlates of physical activity in rural and older african american and white women.* J Gerontol B Psychol Sci Soc Sci, 2003. **58**(6): p. P329-37.

21. Sniehotta, F.F., et al., *Psychological theory in an interdisciplinary context: psychological, demographic, health-related, social, and environmental correlates of physical activity in a representative cohort of community-dwelling older adults.* International Journal of Behavioral Nutrition & Physical Activity, 2013. **10**(1): p. 106-116.

22. Carlson, J.A., et al., *Interactions between psychosocial and built environment factors in explaining older adults' physical activity.* Preventive Medicine, 2012. **54**(1): p. 68-73.

23. Ding, D., et al., *Neighborhood Environment and Physical Activity Among Older Adults: Do the Relationships Differ by Driving Status?* Journal of Aging & Physical Activity, 2014. **22**(3): p. 421-431.

24. Van Cauwenberg, J., et al., *Neighborhood walkability and health outcomes among older adults: The mediating role of physical activity.* Health Place, 2016. **37**: p. 16-25.

25. Michael, Y.L., et al., *Physical Activity Resources and Changes in Walking in a Cohort of Older Men.* American Journal of Public Health, 2010. **100**(4): p. 654-660.

26. Kolbe-Alexander, T.L., et al., *The relationship between the built environment and habitual levels of physical activity in South African older adults: a pilot study.* BMC Public Health, 2015. **15**: p. 518.

27. Lotfi, S. and M.J. Koohsari, *Neighborhood Walkability in a City within a Developing Country.* Journal of Urban Planning and Development-Asce, 2011. **137**(4): p. 402-408.

28. Sugiyama, T. and C.W. Thompson, *Older people's health, outdoor activity and supportiveness of neighbourhood environments.* Landscape and Urban Planning, 2007. **83**(2-3): p. 168-175.

29. Carvalho Sampaio, R.A., et al., *Urban-rural differences in physical performance and health status among older Japanese community-dwelling women.* Journal of Clinical Gerontology and Geriatrics, 2012. **3**(4): p. 127-131.

30. Lee, H.S. and E.Y. Park, *Associations of Neighborhood Environment and Walking in Korean Elderly Women: A Comparison between Urban and Rural Dwellers.* Asian Women, 2015. **31**(4): p. 1-21.

31. Maisel, J.L., *Impact of Older Adults' Neighborhood Perceptions on Walking Behavior.* Journal of Aging and Physical Activity, 2016. **24**(2): p. 247-255.

32. Sewo Sampaio, P.Y., E. Ito, and R.A. Carvalho Sampaio, *The association of activity and participation with quality of life between Japanese older adults living in rural and urban areas.* Journal of Clinical Gerontology and Geriatrics, 2013. **4**(2): p. 51-56.

33. Troped, P.J., et al., *Relationships Between the Built Environment and Walking and Weight Status Among Older Women in Three US States.* Journal of Aging and Physical Activity, 2014. **22**(1): p. 114-125.

34. Thompson, C.W., et al., *Do changes to the local street environment alter behaviour and quality of life of older adults? the 'DIY Streets' intervention.* British Journal of Sports Medicine, 2012. **48**(13): p. 1059-1065.

35. Bracy, N.L., et al., *Is the relationship between the built environment and physical activity moderated by perceptions of crime and safety?* International Journal of Behavioral Nutrition and Physical Activity, 2014. **11**(1).

36. Li, F.Z., et al., *Multilevel modelling of built environment characteristics related to neighbourhood walking activity in older adults.* Journal of Epidemiology and Community Health, 2005. **59**(7): p. 558-564.

37. Shin, W.-H., B.-S. Kweon, and W.-J. Shin, *The distance effects of environmental variables on older African American women's physical activity in Texas.* Landscape and Urban Planning, 2011. **103**(2): p. 217-229.

38. Nagel, C.L., et al., *The relation between neighborhood built environment and walking activity among older adults.* American Journal of Epidemiology, 2008. **168**(4): p. 461-468.

39. Hirsch, J.A., et al., *Destinations That Older Adults Experience Within Their GPS Activity Spaces: Relation to Objectively Measured Physical Activity.* Environment and Behavior, 2016. **48**(1): p. 55-77.
